# Polyphyly of the traditional family Flabellinidae affects a major group of Nudibranchia: aeolidacean taxonomic reassessment with descriptions of several new families, genera, and species (Mollusca, Gastropoda)

**DOI:** 10.3897/zookeys.717.21885

**Published:** 2017-11-30

**Authors:** Tatiana Korshunova, Alexander Martynov, Torkild Bakken, Jussi Evertsen, Karin Fletcher, I Wayan Mudianta, Hiroshi Saito, Kennet Lundin, Michael Schrödl, Bernard Picton

**Affiliations:** 1 Koltzov Institute of Developmental Biology, RAS, 26 Vavilova Str., 119334 Moscow, Russia; 2 Zoological Museum, Moscow State University, Bolshaya Nikitskaya Str. 6, 125009 Moscow, Russia; 3 NTNU University Museum, Norwegian University of Science and Technology, NO-7491 Trondheim, Norway; 4 Port Orchard, Washington 98366, USA; 5 Universitas Pendidikan Ganesha, Bali, 81116, Indonesia; 6 National Museum of Nature and Science, Amakubo 4-1-1, Tsukuba, Japan; 7 Gothenburg Natural History Museum, Box 7283, S-40235, Gothenburg, Sweden; 8 Gothenburg Global Biodiversity Centre, Box 461, S-40530, Gothenburg, Sweden; 9 Zoologische Staatssammlung München, Münchhausenstr. 21, D-81247 München, Germany; 10 Biozentrum Ludwig Maximilians University and GeoBio-Center LMU Munich, Germany; 11 National Museums Northern Ireland, Holywood, Northern Ireland, United Kingdom; 12 Queen’s University, Belfast, Northern Ireland, United Kingdom

**Keywords:** Integration of morphological and molecular data, molecular systematics, Mollusca, morphology, phylogenetics, taxonomic revision

## Abstract

The Flabellinidae, a heterogeneous assembly of supposedly plesiomorphic to very derived sea slug groups, have not yet been addressed by integrative studies. Here novel material of rarely seen Arctic taxa as well as North Atlantic, North and South Pacific, and tropical Indo-West Pacific flabellinid species is investigated morpho-anatomically and with multi-locus markers (partial COI, 16S rDNA, 28S rDNA and H3) which were generated and analysed in a comprehensive aeolid taxon sampling. It was found that the current family Flabellinidae is polyphyletic and its phylogeny and taxonomic patterns cannot be understood without considering members from all the Aeolidacean families and, based on a robust phylogenetic hypothesis, morpho-anatomical evolution of aeolids is more complex than suspected in earlier works and requires reclassification of the taxon. Morphological diversity of Flabellinidae is corroborated by molecular divergence rates and supports establishing three new families (Apataidae **fam. n.**, Flabellinopsidae **fam. n.**, Samlidae **fam. n.**), 16 new genera, 13 new species, and two new subspecies among the former Flabellinidae. Two families, namely Coryphellidae and Paracoryphellidae, are restored and traditional Flabellinidae is considerably restricted. The distinctness of the recently described family Unidentiidae is confirmed by both morphological and molecular data. Several species complexes among all ex-“Flabellinidae” lineages are recognised using both morphological and molecular data. The present study shows that Facelinidae and Aeolidiidae, together with traditional “Tergipedidae”, deeply divide traditional “Flabellinidae.” Diagnoses for all aeolidacean families are therefore provided and additionally two new non-flabellinid families (Abronicidae **fam. n.** and Murmaniidae **fam. n.**) within traditional tergipedids are established to accommodate molecular and morphological disparity. To address relationships and disparity, we propose a new family system for aeolids. Here the aeolidacean species are classified into at least 102 genera and 24 families. Operational rules for integration of morphological and molecular data for taxonomy are suggested.

## Introduction

Flabellinids are a large family of commonly occurring cnidosac-bearing nudibranchs, especially abundant and diverse in boreal and Arctic regions. Information on taxonomy of various flabellinids can be found in several reviews of opisthobranch regional faunas (e.g., Odhner 1907, 1922, 1939; Marcus and Marcus 1963; MacFarland 1966; Behrens 1980, 1991; Behrens and Hermosillo 2005; Gosliner 1980; Gosliner and Griffiths 1981; Thompson and Brown 1984; Picton and Morrow 1994; Rudman 1998; Schrödl 2003) as well as separate publications specially focused on the selected taxa of the family Flabellinidae (Kuzirian 1977, 1979; Picton 1980; García-Gómez 1986; Hirano and Thompson 1990; Gosliner and Willan 1991; Cervera et al. 1998; DaCosta et al. 2007; Millen and Hermosillo 2007; and many others). Currently, approximately 74 species are included in Flabellinidae. Within the family Flabellinidae, the genus-level taxonomy is confused; whereas a majority of the species (64) of this large family have been placed within the single genus *Flabellina* (Gosliner and Griffiths 1981), it is a questionable placement since *Flabellina* in the current sense encompasses morphologically different Arctic and tropical species (Wägele and Willan 2000). Moreover, despite the fact that many species have been listed under the genus name *Flabellina* Gray, 1833, several genera like the Arctic *Chlamylla* Bergh, 1899 and the Mediterranean *Calmella* Eliot, 1910 were not synonymised with *Flabellina*, despite the similarity of their distinguishing characters to those of some other species currently considered to belong to *Flabellina*. There are no complete revisions of the family Flabellinidae or novel integrative studies on this family combining both morphological and molecular data. Available reviews are restricted mostly to some warm water species based on a morphological approach (Gosliner and Kuzirian 1990; Millen and Hamann 2006). There are some molecular data on flabellinids scattered in more recent publications on other aeolidacean groups (e.g., Carmona et al. 2013) and a recent achievement in molecular and morphological studies on Mediterranean and NE Atlantic species (Furfaro et al. 2017), but there are no recent attempts to understand the broad-scope taxonomic diversity of traditional Flabellinidae and their major phylogenetic patterns.

The aim of this study is to use novel material from unique and rarely seen Arctic species as well as North Atlantic, North and South Pacific, and tropical Indo-West Pacific flabellinid species to investigate their anatomy and to test the phylogenetic relationships of the current Flabellinidae in a comprehensive molecular framework. It was found that the current concept of Flabellinidae is deeply polyphyletic and that its phylogeny and taxonomic diversity cannot be understood without considering members from the majority of families in Aeolidacea. Morphological data and molecular analyses presented here lay a foundation for a modern revision and reclassification of one of the largest subgroups of nudibranchs, the Aeolidacea.

## Materials and methods

### Collecting data

Material for this study was obtained from various expeditions and fieldwork, and included specimens belonging to the different taxa of the family Flabellinidae. All specimens were deposited in the Zoological Museum, Moscow Lomonosov State University (**ZMMU**), Norwegian University of Science and Technology (**NTNU**), University Museum Trondheim (**NTNU-VM**), National Museums Northern Ireland, Cultra, Belfast, Gothenburg Natural History Museum (**GNM**), and the Bavarian State Collection of Zoology, Munich (**ZSM**). Type specimens from the Natural History Museum of Denmark (**NHMD**) have been also investigated. The majority of specimens of the new Coryphellidae species were collected alive at Gulen Dive Resort (Norway, north of Bergen). Other flabellinid species used here for comparison have been collected in various locations in northern Eurasia, including Banyuls-sur-Mer, Vigo, Ireland, middle Norway, Spitsbergen, Barents Sea, White Sea, Kara Sea, Laptev Sea, Chuckchi Sea, Bering Strait, Commander Islands, Bering Sea, Japan Sea, the Pacific side of the Japanese Islands as well as South America, Vietnam, Indonesia, and the Pacific coast of the USA. All necessary permissions have been obtained during the above-mentioned collections. The Balinese nudibranch specimen was collected under the permit of Governor of Bali No. 070/4710/IV/BPMP/2016.

### Morphological analysis

The external morphology of specimens was studied under a stereomicroscope. For the description of internal features, we dissected both preserved and fresh specimens (when available) under the stereomicroscope. The buccal mass of each specimen was extracted and soaked in 10% sodium hypochlorite solution for 1–2 minutes to dissolve connective and muscle tissue, leaving only the radula and the jaws. The features of the jaws of each species were analysed under the stereomicroscope and scanning electron microscope, and then drawn. The coated radulae were examined and photographed using a scanning electron microscope (CamScan). The reproductive systems of different species were also examined and drawn using the stereomicroscope. In the description of reproductive characters, we consistently apply the terms “proximal receptaculum seminis” and “distal receptaculum seminis” (= bursa *sensu* e.g., Gosliner and Griffiths 1981 and other authors) since it was already clearly shown that according to their internal structure, both seminal reservoirs in aeolidacean nudibranchs are actually receptacula with sperm attached to the wall (Schmekel and Portmann 1982; Wägele and Willan 2000; Fischer et al. 2007).

### Molecular analysis

In total, 126 specimens were successfully sequenced for the mitochondrial genes cytochrome c oxidase subunit I (COI) and 16S rRNA, and the nuclear genes Histone 3 (H3) and 28S rRNA (C1–C2 domain). Additional sequences, including outgroup specimens, were obtained from GenBank (see Supplementary material 2: Table S1 for a full list of samples, localities, and voucher references). Small pieces of tissue were used for DNA extraction with Diatom DNA Prep 100 kit by Isogene Lab, according to the producer’s protocols. Extracted DNA was used as a template for the amplification of partial sequences of COI, 16S, H3 and 28S (see Suppl. material 3: Table S2 for primers). Polymerase chain reaction (PCR) amplifications were carried out in a 20-μL reaction volume, which included 4 μL of 5x Screen Mix (Eurogen Lab), 0.5 μL of each primer (10 μM stock), 1 μL of genomic DNA, and 14 μL of sterile water. The amplification of COI and 28S was performed with an initial denaturation for 1 min at 95 °C, followed by 35 cycles of 15 sec at 95 °C (denaturation), 15 sec at 45 °C (annealing temperature), and 30 sec at 72 °C, with a final extension of 7 min at 72 °C. The 16S amplification began with an initial denaturation for 1 min at 95 °C, followed by 40 cycles of 15 sec at 95 °C (denaturation), 15 sec at 52 °C (annealing temperature), and 30 sec at 72 °C, with a final extension of 7 min at 72 °C. Sequencing for both strands proceeded with the ABI PRISM BigDye Terminator v. 3.1. Sequencing reactions were analysed using an Applied Biosystems 3730 DNA Analyzer. Some COI sequences were produced at the Canadian Centre for DNA Barcoding (CCDB), using their automated systems for extraction, PCR, and sequencing.

Protein-coding sequences were translated into amino acids for confirmation of the alignment. All sequences were deposited in GenBank (Suppl. material 2: Table S1, highlighted in bold). Original data and publicly available sequences were aligned with the MUSCLE algorithm (Edgar 2004). Separate analyses were conducted for COI (657 bp), 16S (434 bp), H3 (327 bp) and 28S (327 bp). Gblocks 0.91b (Talavera and Castresana 2007) was applied to discard poorly aligned regions for the 16S data set and for the 28S data set (using less stringent options; in total, 11% and 5% of the positions were eliminated). An additional analysis was performed with all four concatenated markers (1745 bp). Evolutionary models for each data set were selected using MrModelTest 2.3 (Nylander et al. 2004) under the Akaike information criterion (Akaike 1974). The GTR + I + G model was chosen for COI, 16S, 28S, H3 and for the combined dataset. Two different phylogenetic methods, Bayesian inference (BI) and Maximum likelihood (ML) were used to infer evolutionary relationships. Bayesian estimation of posterior probability was performed in MrBayes 3.2 (Ronquist et al. 2012). Markov chains were sampled at intervals of 1000 generations. Analysis was started with random starting trees and 6 × 10^6^ generations. Maximum likelihood-based phylogeny inference was performed in RAxML 7.2.8 (Stamatakis et al. 2008) with bootstrap in 1000 pseudo-replications. Final phylogenetic tree images were rendered in FigTree 1.4.2. Nodes in phylogenetic trees with Bayesian posterior probability values ≥0.96 (pp) and bootstrap values ≥90% (bs) were considered well-supported, nodes with 0.90–0.95 and 80–89% accordingly were considered moderately supported (lower support values were considered not significant) (e.g., Furfaro et al. 2016). The program Mega7 (Kumar et al. 2016) was used to calculate the uncorrected p-distances between all the sequences and pairwise uncorrected p-distances within and between clades.

### Integration of morphological and molecular data (operational rules)

There is an extensive body of literature regarding the importance of an integrative approach which is targeted to employ both morphological and molecular data (e.g., Dayrat 2005; Schlick-Steiner et al. 2010; Papakostas et al. 2016; Korshunova et al. 2017a, b). In aeolidaceans clear preference is currently given to the molecular data and a classification is often constructed following the molecular phylogenetic trees (e.g., Carmona et al. 2013; Cella et al. 2016). Because there is no epistemological evidence that molecular data should have preference over morphological features (e.g., Mishler 1994; Giribet 2010; Bazsalovicsová et al. 2014; Jenner 2015; Anton et al. 2016), we have developed several operational rules: 1) Both morphological and molecular data should be utilised in the resulting classification; 2) Morphologically highly aberrant taxa (e.g., family- or genus-level) nested inside numerous taxa with disparate morphology should not be united with the rest of the related taxa but kept separate to highlight significant morphological differences; 3) Taxa for which molecular data persistently indicate the heterogeneous nature of a traditional taxon (e.g., family level) with apparently similar morphology (“para-” or “polyphyly”) should be separated into several taxa of the same rank; 4) Large-volume genera incorporating numerous species should be avoided because they considerably obscure both morphological and molecular diversity and do not properly allow the recognition of hidden diversity.

The foundation of the empirical rules outlined here is rooted in the following biological facts: i) Developmental genes (e.g., *Homeobox*, etc.) show a considerable level of conservatism over large phylogenetic distances that imply that similar morphological features may appear in taxa which are not closely related, ii) There is recent evidence on the importance not only of genetic but also epigenetic interactions, that implies that genes can be changed not just according to inferred molecular phylogenetic trees, iii) There is compelling recent evidence that evolutionary (phylogenetic) patterns of any groups of living organisms are extremely complicated and include numerous para- and polyphyletic events, iv) Therefore, in order to construct a classification which will reflect such complicated patterns in nature and not be constructed for merely logical or didactic purposes (e.g., “convenience”, “ease of use”, etc.) the resulting classification should be equally complex.

The separation of smaller classificatory groups/units also leads to increasing objectivity in taxonomy. Indeed, “objectivity” is a very complicated and not an equivocal term and cannot be applied to the taxonomic field without reservation, despite the assertions of some authors that there is an objective interpretation of the phylogeny and morphological characters. However, instead of clarification, lumping morphologically diverse genera and families leads to a decrease of objectivity since the decision as to which genus/family should be united and which should not is an extremely subjective process even if a molecular phylogeny is used as the primary justification. This subjectivity was clearly demonstrated by the recent molecular phylogeny of tergipedid aeolidacean nudibranchs (Cella et al. 2016), when some genera were united into super-lumping groups like *Tenellia*, whereas other closely related genera like *Tergipes* and *Rubramoena* were instead kept separate. The uniting of several previously clearly morphologically delineated families of aeolidaceans into the single family Fionidae (Cella et al. 2016) led, in turn, to the loss of any reliable morphological diagnostic features (see discussion in Korshunova et al. 2017a). The absence of the morphological synapomorphies for “*Tenellia*” in the sense of Cella et al. (2016) was also independently noted most recently in Goodheart et al. (2017). Thus, an interpretation of a phylogenetic framework itself is by no means an “objective process.” A putative hypothesis-driven modern taxonomic approach apparently based on clear testable hypothesis and reproducible methodological frameworks did not necessarily lead to objective decisions and classifications.

Taxonomic objectivity can be increased using consistent separation of small maximally coherent morphological and molecular taxonomic groups (taxa). It is very important to highlight that the separation of a small coherent group/unit does not specially imply a bias toward splitting in the classical taxonomic lumping/splitting dilemma. Instead, the necessity of splitting many traditional taxa is rather to conform to the molecular phylogenies which, in many cases, are also confirmed by morphological data reflecting the extremely complicated and mosaic pattern of natural evolutionary pathways which favour splitting, but at a new level supported in a modern and integrative way. Of course, there are many pitfalls in this method as well and it deserves wider discussion, but an objective truth is that apparently objective molecular-based methods do not imply a single resulting objective classification. Furthermore, since we still use a rigid binomial nomenclature and taxa hierarchy developed long prior to any phylogenetic and evolutionary conceptions as an unavoidable taxonomic rule, we should attempt to adapt an archaic system into extremely complicated phylogenetic patterns recently discovered in most of the organism groups. There is an immense body of literature on the relationship between taxonomy, phylogeny, and nomenclature (e.g., Simpson 1961; Hennig 1966; Ridley 1986; Queiroz and Gauthier 1992; Wägele 2005; Schuh and Brower 2009; Wiley and Lieberman 2011; Hubert and Hanner 2015; and many others). More recently a proposal regarding, for example, PhyloCode (Rieppel 2006; Queiroz 2006) was widely discussed, as well as other suggestions for linking molecular and traditional taxonomy (e.g., Galimberti et al 2012; Vences et al. 2013; Hedges 2013; Franz et al. 2015) but did not lead to any substantial changes to current taxonomic nomenclatural practice, which still only insignificantly differs from the Linnaean system. Our present study does not intend to review theoretical literature on this topic; however, without setting some operational rules/criteria, we cannot accommodate our results in a practical way.

These operational rules are consistently applied here (as far as possible) for the taxonomy of one of the largest and most complicated traditional families of nudibranchs, the Flabellinidae, and for the discussion of the general classification of one the major traditional subgroups of Nudibranchia, the Aeolidacea. These rules may help taxonomists with the actual integration of molecular and morphological data instead of a truly authoritative commonly held view that a taxonomist must just follow molecular phylogenetic patterns without any settled guidelines on how to convert the phylogenetic pattern into a taxonomic system and how to integrate, in many cases, considerable molecular and morphological disparity. The consistent application of the small coherent taxonomic groups concept may also help to resolve ongoing debates on the treatment of paraphyletic groups in taxonomy (e.g., Seifert et al. 2016; Ward et al. 2016) since on one hand, this concept targets avoidance of paraphyletic taxa to a maximal degree but on the other hand, it does not mask taxonomic diversity by uniting smaller monophyletic groups into larger, non-diagnosable units without support of morphological apomorphies (see also Korshunova et al. 2017a).

An independent support for the validity of our approach appeared in a recent study while our paper was under review (Zamora-Silva and Malaquias 2017). In that study, a revision of the traditional family Aglajidae was undertaken, and numerous new genera were proposed to accommodate complex phylogenetic patterns and morphological disparity. This aligns well with the principles of preferred separation of small, morphologically and molecularly coherent taxonomic groups/units proposed here. The most recent debates in amphibian taxonomy (Scherz et al. 2017) also support the small coherent unit taxonomic approach developed here.

## Results

### Molecular phylogeny

**Figure 1.**
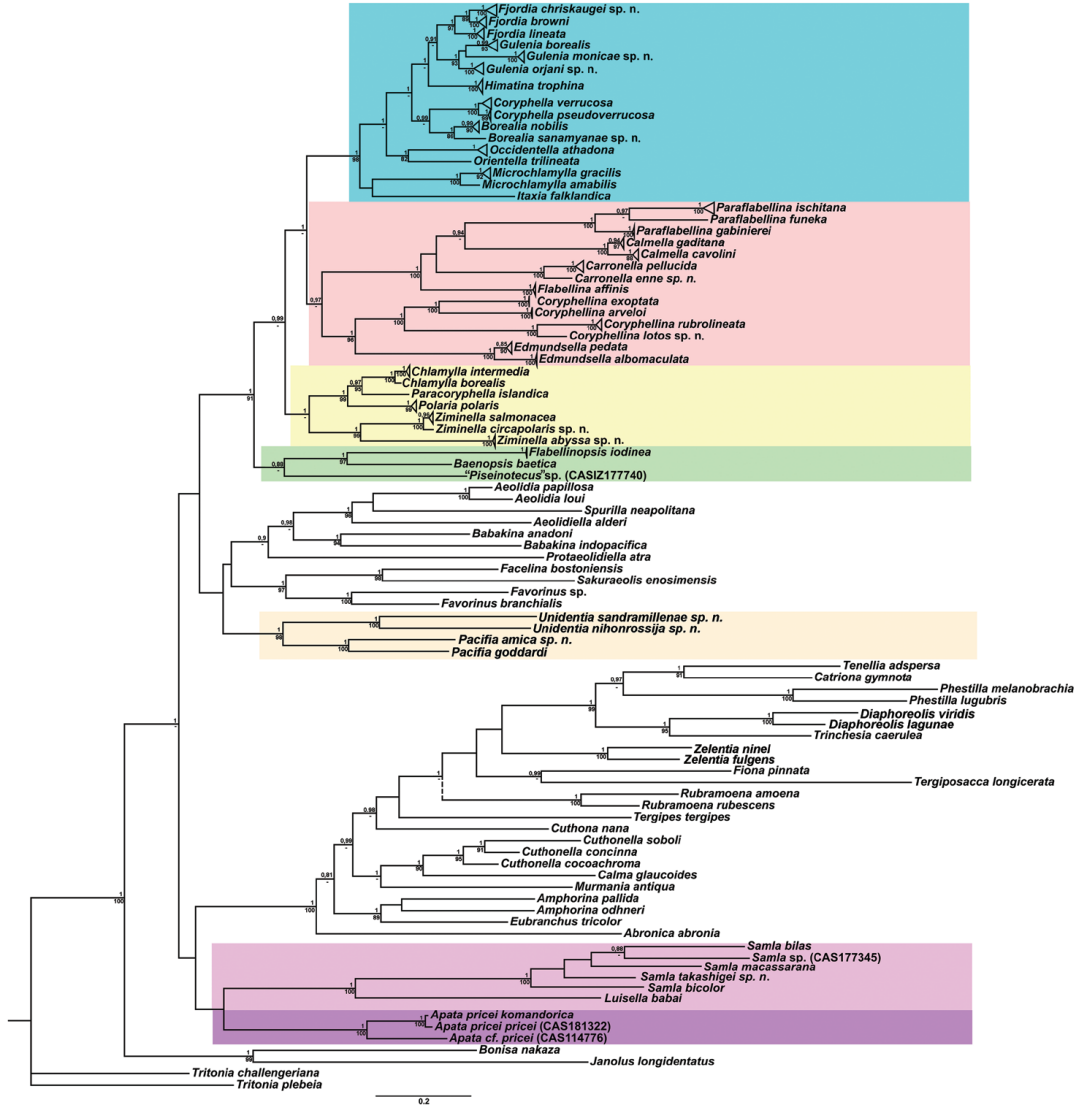
Phylogenetic tree of aeolidacean nudibranchs based on concatenated molecular data (COI + 16S + 28S + H3) represented by Bayesian Inference (BI). Numbers above branches represent posterior probabilities from Bayesian Inference. Numbers below branches indicate bootstrap values for Maximum Likelihood. Some branches are collapsed at species level. The “Flabellinidae” polyphyletic family complex is highlighted by different colours (see Fig. 2 for the family names).

**Figure 2.**
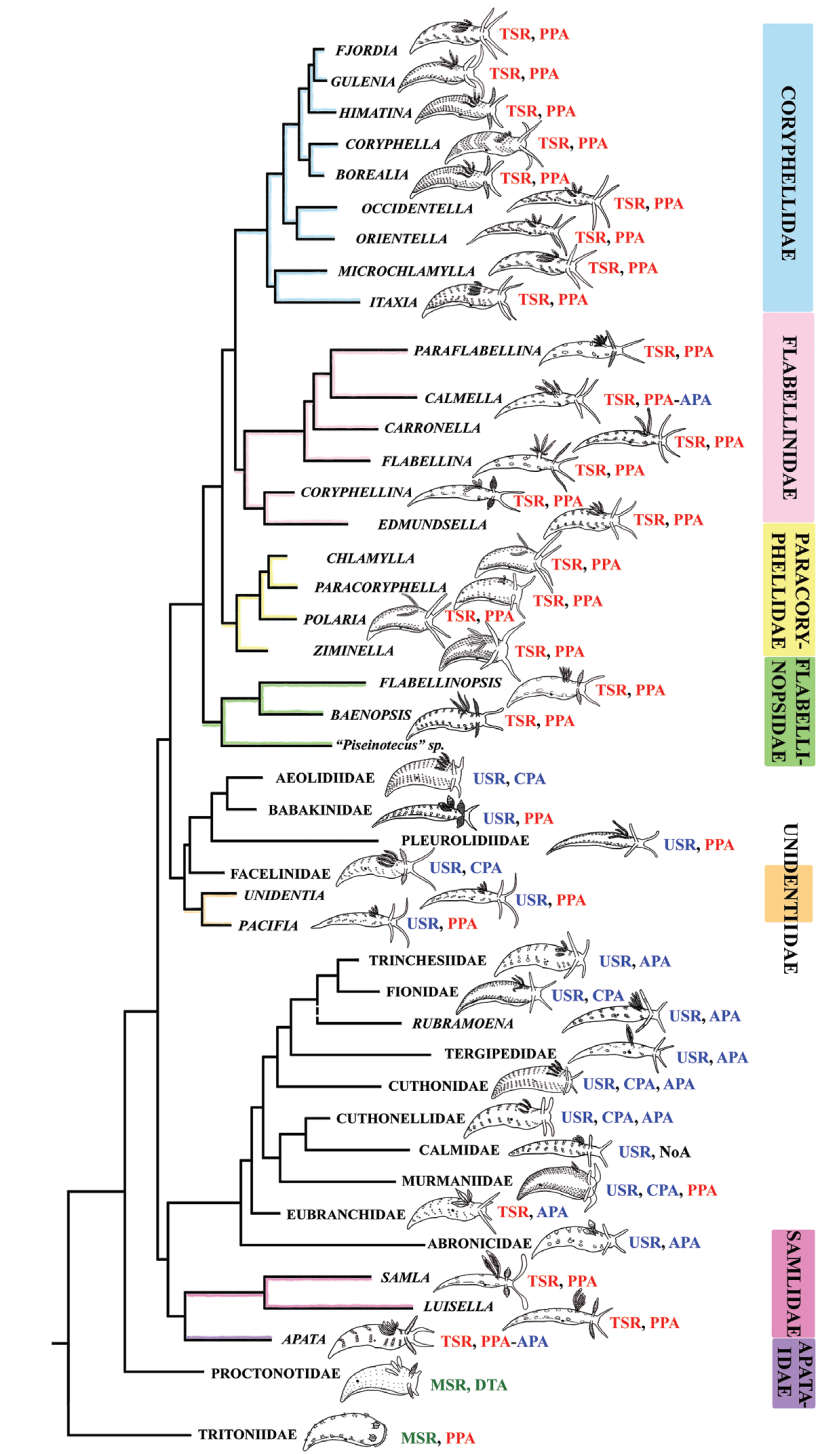
Phylogenetic tree of aeolids with integrated data on external morphology. The “Flabellinidae” polyphyletic family complex is highlighted by different colours; names of the family- and genus-level taxa are provided. Abbreviations: **CPA** cleioproctic anus **APA** acleioproctic anus **DTA** dorso-terminal anus **MSR** multiserial radula **PPA** pleuroproctic anus **TSR** triserial radula **USR** uniserial radula.

In this molecular study, 205 specimens were included, combining 404 novel sequences with 230 from GenBank. A total of 90 species was selected to represent all conventional flabellinid subgroups and all aeolid families with multi-locus data available. Bayesian Inference (BI) and Maximum Likelihood (ML) analyses based on the combined dataset for the mitochondrial genes COI and 16S, and the nuclear genes H3 and 28S yielded similar results (except the position of the *Rubramoena* clade, see Discussion for details) and revealed that the current family Flabellinidae is deeply polyphyletic (Figs 1–2). Highly supported and moderately supported nodes have been analysed (Fig. 1). Especially interesting is the fact that putatively highly derived *Flabellina* *sensu lato* are comprised of several definitely non-related groups in relation to other non-flabellinid groups such as Aeolidiidae, Facelinidae, Tergipedidae, and others (Fig. 2), thus rendering traditional Flabellinidae polyphyletic. The results of molecular phylogenetic analyses (BI and ML) support several family-level taxa.

The molecular phylogenetic analyses in combination with species delimitation analysis support the presence of several new species. Furthermore, a flabellinid species *Coryphella lineata* (Lovén, 1846), commonly considered to be a single species, is actually a highly heterogeneous group comprised of at least four species (three of which are new) and two new genera; species complexes were also discovered among many other lineages (Fig. 1).

### Taxonomy of the traditional family Flabellinidae

The family Flabellinidae is a morphologically very diverse assemblage and historically several genera have been created to encompass this species diversity (Bergh 1900a; Odhner 1907; Odhner 1968). The majority of the species were nevertheless separated within two major genera, *Flabellina* and *Coryphella*. The genus *Flabellina* was characterised by elevated stalk-like groups of cerata, whereas *Coryphella* was diagnosed as having non-elevated cerata inserted directly to the notum. Considering the extreme diversity within the family, a rigid classification based on just two character states (stalked vs. non-stalked cerata) was clearly inadequate and fails to acceptably place many intermediate taxa. For example, other genera with a continuous ample notal margin have been described, *Chlamylla* Bergh, 1886 and *Paracoryphella* Miller, 1971 (Bergh 1886, 1900a; Miller 1971) as well as several genera with the cerata on stalks or elevations, *Samla* Bergh, 1900, *Nossis* Bergh, 1902, *Tularia* Burn, 1966, *Flabellinopsis* MacFarland, 1966 (Bergh 1900b; Bergh 1902; Burn 1964, 1966; MacFarland 1966), but they were largely not incorporated into the broad-scale taxonomy of the family Flabellinidae. In 1981 when describing some new “intermediate” species a novel revision of the genus-level taxonomy of the family Flabellinidae was clearly needed; however, a decision was made to merge the overwhelming majority of flabellinid species under just the single oldest genus name, *Flabellina* Gray, 1833 (Gosliner and Griffiths 1981). This decision was justified thus: “Mayr (1969) suggested that a distinct morphological gap should exist between genera. The presence of intermediate forms with poor correlation of morphological characteristics suggests that maintenance of the generic separation of *Coryphella* and *Flabellina* is untenable.” (Gosliner and Griffiths 1981: 110). However, apart from this theoretical reasoning, which by no means represented any obligatory rule that any taxonomist should strictly follow and which also implies very subjective decisions, a thorough revision of the generic classification of the family Flabellinidae was absent and the majority of flabellinid species were merely listed without any detailed discussion under the name *Flabellina* (Gosliner and Griffiths 1981).

The present phylogenetic analysis of a broad selection of various Arctic and tropical flabellinid taxa reveals that fundamentally different flabellinid clades have been concealed under apparent “intermediate forms” (see below and Figs 1–2). One of the most remarkable results of the present analysis is that members of the traditional family “Flabellinidae” are phylogenetically extremely heterogeneous according to the molecular data, confirming and even extending earlier morphology-based assumptions (Wägele and Willan 2000; Martynov 2006a). The highly derived *Flabellina* *sensu stricto* are comprised of several non-related groups, and the inclusion of several disparate non-flabellinid families (Fig. 2) makes traditional Flabellinidae at least triply polyphyletic. Thus, the previously unchallenged decision to merge all diversity of the traditional family Flabellinidae has considerably masked not only the generic diversity within the traditional Flabellinidae (which is higher than currently recognised), but also Flabellinidae *sensu lato* actually embraced most other aeolid family-level taxa. This conclusion is foreshadowed by the anomalous position of *Flabellina babai* revealed by Carmona et al. (2013) and the trees presented by Furfaro et al. (2017) while our present study was under review.

Previous researchers who focused on flabellinids may have suspected that the taxon was paraphyletic; if so, they did not propose a satisfactory solution to incorporate those doubts. For example, while almost all flabellinid species were placed under the name *Flabellina* by Gosliner and Griffiths (1981), they did not consider several genera which already existed, e.g., *Chlamylla* and *Calmella*. As a result, while the genus *Chlamylla* was not synonymised with *Flabellina*, one species, *Coryphella orientalis* Volodchenko, 1941, was renamed as *Flabellina orientalis* (Volodchenko 1941) and a junior objective synonym *Coryphella barentsi* Derjugin & Gurjanova, 1926 was given a new name *Flabellina incognita* (Gosliner & Griffiths, 1981: 112). However, *Coryphella orientalis* Volodchenko, 1941 is actually a junior synonym of *Chlamylla atypica* (Volodchenko 1941; Martynov 2006a: 283) and *Flabellina incognita* is, according to the radular morphology and presence of an external penial collar, also a member of the genus *Chlamylla*. Thus, at least two species from the genus *Chlamylla* were listed under the genus name *Flabellina*, yet the genus *Chlamylla* was neither synonymised with *Flabellina* nor even mentioned in Gosliner and Griffiths (1981) or any subsequent papers. The same treatment was applied to some stalked/elevated cerata-bearing genera, e.g., *Calmella* and *Tularia*. The Australian and New Zealand genus and species *Tularia bractea* (Burn, 1966) which possesses all of the advanced flabellinid characters (triserial radula, cerata on raised elevations, reproductive system without penial gland) was also either not discussed or not included in the genus *Flabellina*. Therefore, a genus-level revision of the family is highly desirable. Several authors continued to use at least the genus name *Coryphella* as separate from *Flabellina* (Thompson and Brown 1984; Roginskaya 1987; Picton and Morrow 1994; Martynov 2006a; Martynov and Korshunova 2011); however, the very poorly defined *Flabellina* *sensu lato* dominates in common usage. Most importantly, this question is not just purely theoretical or redundant, but instead deeply affects the core of practical aeolid taxonomy. For example, assessment of the phylogenetic relationship of the flabellinid species complex *C. lineata* (Lovén, 1846), performed in the course of the present study, was impossible without a broad comparison between different flabellinid taxa.

Our molecular phylogeny thus shows that flabellinids with elevated/stalked cerata are clearly polyphyletic and cannot therefore be maintained within the same genus or even within the same family (Fig. 2). Actually, there are also several clades of flabellinids with continuous rows of cerata, with significant genetic gaps, that are now separated. The current practice of genus separation into many different groups is very far from the simplistic consideration that the presence of some “intermediate forms” is sufficient for synonymising genera. Instead, differences used for establishing the numerous new genera in a group in most recent papers (e.g., Bourguignon et al. 2016; Zamora-Silva and Malaquias 2017) shows that genera can be distinguished by subtle morphological characters. The dominant current classification of the family Flabellinidae is also very incompatible with the approach that is widely utilised in the family Facelinidae, one of the closest to the flabellinids, where numerous genera including monotypic ones are currently widely utilised (e.g., Millen and Hamann 1992; Rudman 1998; Hirano 1999; Millen and Hermosillo 2012; see Supplementary materials). Furthermore, there are recent proposals (e.g., Carmona et al. 2013, Knutson and Gosliner 2014), although not universally accepted, that COI divergence more than 11.1 ± 5.1% in molluscs is sufficient for separation of genus-level taxa. This implies broad limits of the molecular foundations of the genus-level taxa since divergence of more than ca. 5% may imply status of a separate genus for a particular species or a species complex. Thus, actual taxonomic practice of how many genera can be maintained in any group is not a field of universally accepted and clear rules, but instead a very complicated mixture of traditional authority and variously interpreted morphological and molecular data.

Given the great morphological and molecular diversity of the family Flabellinidae, independently arising *Flabellina*-like taxa with elevated/stalked cerata with very significant molecular divergence (Fig. 1) and similar significant divergence between different taxa with continuous cerata (Fig. 2), it is impossible to maintain only the traditional pair of taxa *Flabellina*-*Coryphella* but equally impossible to merge all flabellinid diversity into the single genus *Flabellina* and the single family Flabellinidae. The para- and polyphyletic nature of the genus *Coryphella* itself, after the exclusion of *Flabellina*, has already been noticed (Gosliner and Willan 1991). Not only molecular, but morphological differences within the family Flabellinidae are so significant that several species are formally still included under the generic name *Flabellina*, but actually far exceed the taxonomic diagnosis of the family Flabellinidae. For example, *Flabellina rubrolineata* (O’Donoghue, 1929) and several closely related species uniquely possess a putative triaulic reproductive system (Gosliner and Willan 1991). This character normally present in completely different dorid nudibranchs (Schmekel 1970, 1971), was originally described under the new genus *Coryphellina* O’Donoghue, 1929 but this is currently universally included within *Flabellina*. The recently described *Flabellina goddardi* Gosliner, 2010 has a uniserial radula, a very important character, never present in any other flabellinid taxa, which instead invariably possess a triserial radula (Gosliner 2010). There were preliminary data (González-Duarte et al. 2008) that another genus with uniserial radula, *Piseinotecus*, which is still traditionally placed within the separate family Piseinotecidae, is closely related to some *Flabellina* species. Most recently, Furfaro et al. (2017) confirmed this and have shown that in most of the NE Atlantic and Mediterranean species previously assigned to genus *Piseinotecus* the true triserial radula is present, and delicate lateral denticles were just overlooked in previous studies.

Thus, the only plausible alternative is a careful distinction of family-level groups and numerous genus-level taxa within the traditional family Flabellinidae employing both molecular and morphological evidence (Fig. 2). This approach is especially important when the number of morphologically difficult-to-distinguish species is continually growing and to attribute new species correctly requires increasingly narrower definitions of the genera used. Thus, having a morphologically disparate, large-volume genus we can never consistently describe many potential cryptic species (for usage of the term cryptic species see Korshunova et al. 2017b). Instead, narrowly-defined genera are more consistent with an approach to recognise potential species within morphologically homogeneous narrow genera. The theoretical and practical framework developed here is consistently applied below in detail for the Flabellinidae *s. l.* complex.

Importantly, although this study does not include molecular data on all species of traditional Flabellinidae, we present the largest taxon selection of traditional Flabellinidae and related groups (ranging from the North Pole to the tip of southern America through tropical regions) ever studied. Such an approach allows us to integrate morphological and molecular data and in most cases to suggest generic placement of species for which molecular data are not yet available.

#### Family Paracoryphellidae

Taxon classificationAnimaliaNudibranchiaParacoryphellidae

Miller, 1971, reinstated

##### Diagnosis.

Body wide. Notal edge present, well-defined, continuous. Cerata not stalked, in continuous numerous rows. Rhinophores smooth to wrinkled. Anus pleuroproctic under the notal edge. No distinct oral glands. Radula formula 1.1.1. Asymmetrically placed additional 1–3 rows of small reduced lateral teeth may be present. Rachidian teeth with strong cusp, never compressed by adjacent lateral denticles. Lateral teeth narrow or with attenuated process basally, usually denticulated. Commonly only single distal receptaculum seminis present. Vas deferens always long, with wide granulated or tubular prostate. External permanent penial collar present in some taxa. Penis elongated conical, internal or fully external, unarmed.

##### Genera included.

*Chlamylla* Bergh, 1886, *Paracoryphella* Miller, 1971, *Polaria* gen. n., *Ziminella* gen. n.

#### Chlamylla

Taxon classificationAnimaliaNudibranchiaFlabellinidae

Bergh, 1886

**Figure 3.**
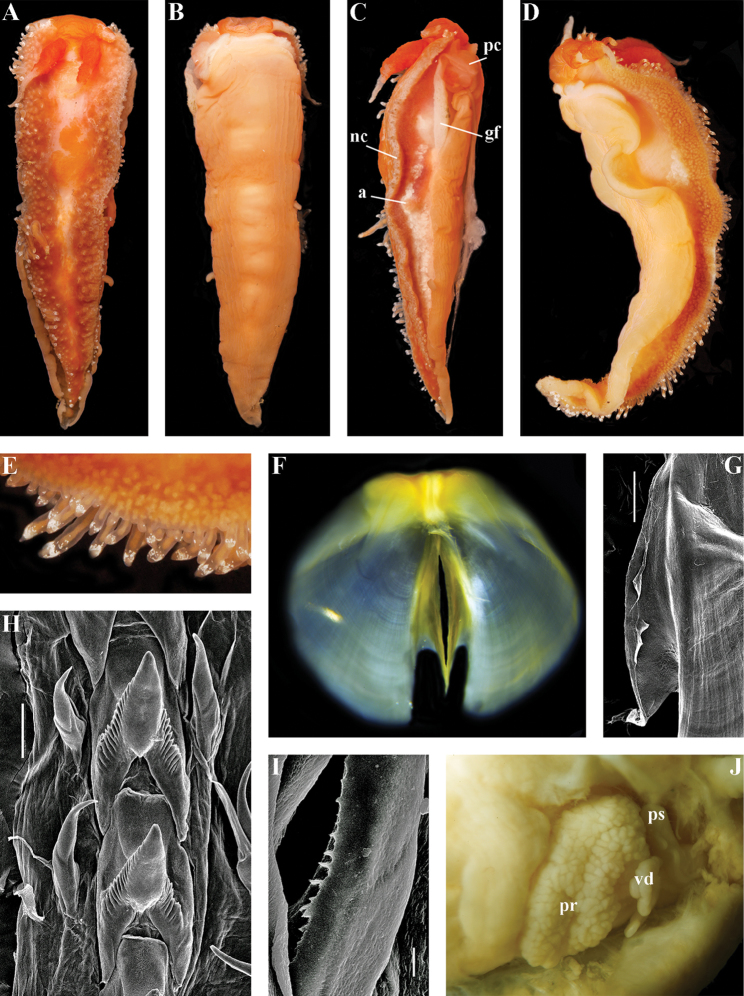
*Chlamylla borealis borealis* Bergh, 1899, stat.n. ZMMU Op-479. Kara Sea, living animal 46 mm in length: **A** dorsal view **B** ventral view **C** right lateral view **D** left lateral view **E** details of cerata and notal edge **F** jaws, frontal view, light microscopy **G** details of masticatory process of jaw, SEM **H** radular teeth, middle part, SEM **I** details of small denticles on lateral teeth, SEM **J** reproductive system, light microscopy. Abbreviations: **a** anus **gf** genital fold **nc** continuous notal edge **pc** penial collar (external) **pr** prostate **ps** penial sheath **vd** vas deferens (muscular part). Scale bars: **G** = 500 μm; **H** =100 μm; **I** = 10 μm. Photos of living specimens by O.L. Zimina, other photos and SEM images by A.V. Martynov.

**Figure 4.**
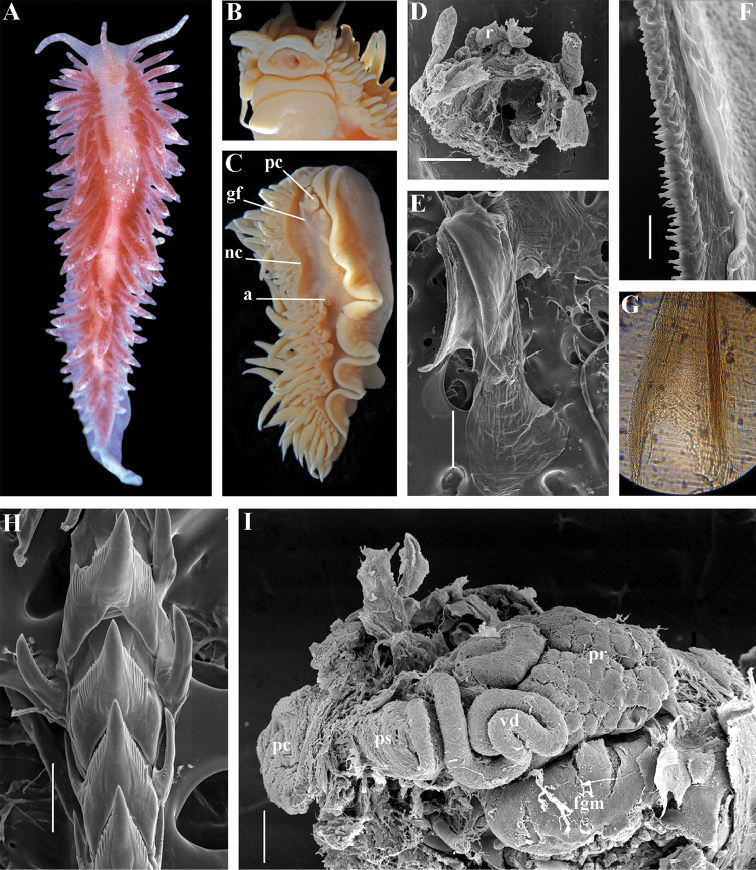
*Chlamylla borealis orientalis* (Volodchenko, 1941), comb. n. ZMMU Op-478. The Sea of Japan, Vostok Bay, 15 mm in length: **A** living animal, dorsal view **B** fixed animal, ventral view of anterior part **C** fixed animal, lateral view **D** dissected anterior part (pharynx removed) and rhinophores, SEM **E** jaw, SEM **F** details of masticatory process of jaw, SEM **G** details of masticatory process of jaw, light microscopy **H** radular teeth, posterior part, SEM **I** reproductive system, SEM. Abbreviations: **a** anus **gf** genital fold **fgm** female gland mass **nc** continuous notal edge **pc** penial collar (external) **pr** prostate **ps** penial sheath **r** rhinophores **vd** vas deferens (muscular part). Scale bars: **D** = 1 mm; **E, I** = 300 μm; **F** = 30 μm; **H** = 100 μm. Photos and SEM images by A.V. Martynov.

**Figure 5.**
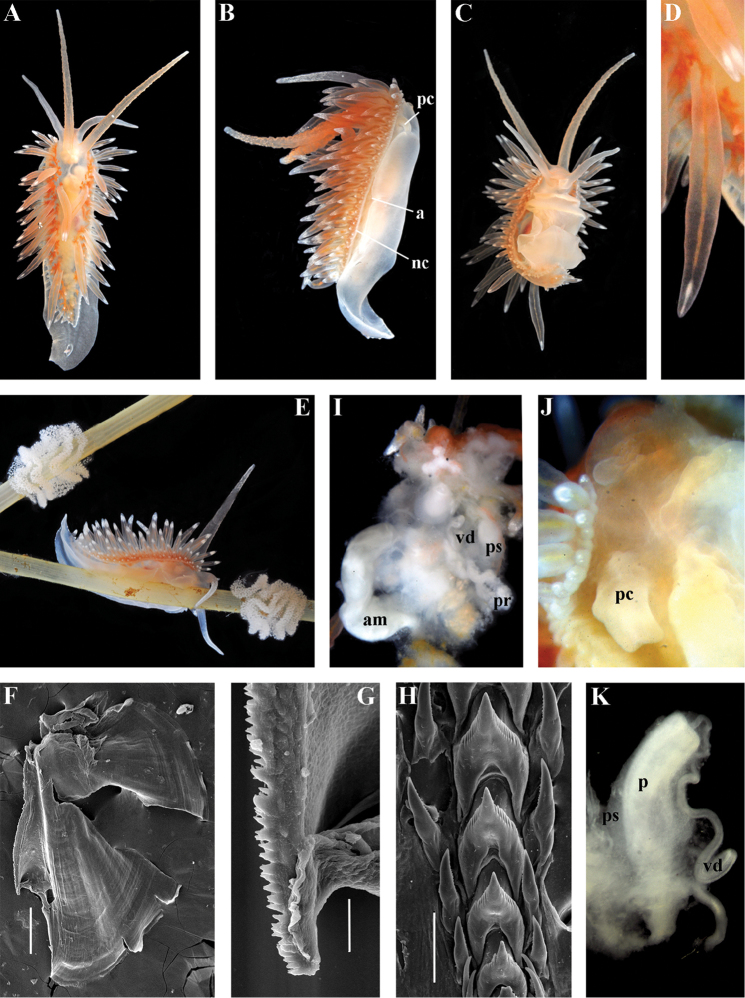
*Chlamylla intermedia* (Bergh, 1899). ZMMU Op-480. White Sea, Cape Kartesh, living animal 23 mm in length: **A** dorsal view **B** lateral view **C** ventral view **D** details of cerata **E** living animal and its egg masses on a *Tubularia* stem **F** jaw, SEM **G** details of masticatory process of jaw, SEM **H** radular teeth, posterior part, SEM **I** reproductive system **J** details of external penial collar **K** details of penis inside of penial sheath. Abbreviations: **a** anus **am** ampulla **nc** continuous notal edge **p** penis **pc** penial collar (external) **pr** prostate **ps** penial sheath **vd** vas deferens (muscular part). Scale bars: **F** = 300 μm; **G** = 30 μm; **H** = 100 μm. Photos and SEM images by T.A. Korshunova, A.V. Martynov.

**Figure 6.**
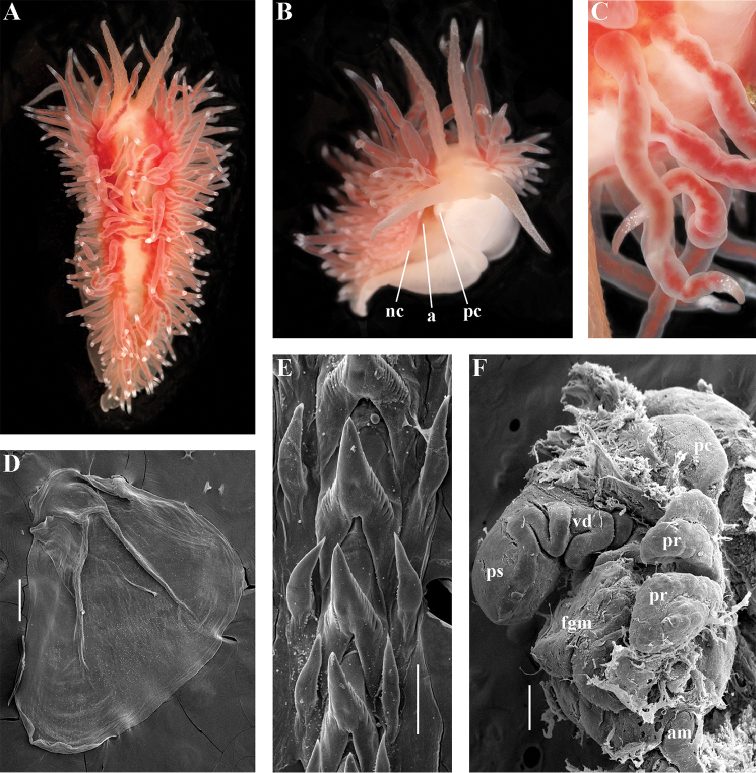
*Chlamylla intermedia* (Bergh, 1899). ZMMU Op-481. Laptev Sea, living specimen 15 mm in length: **A** dorsal view **B** latero-ventral view **C** details of cerata **D** jaw, SEM **E** radular teeth, posterior part, SEM **F** reproductive system, SEM. Abbreviations: **a** anus **am** ampulla **fgm** female gland mass **nc** continuous notal edge **pc** penial collar (external) **pr** prostate **ps** penial sheath **vd** vas deferens (muscular part). Scale bars: **D** = 300 μm; **E** = 100 μm; **F** = 300 μm. Photos of living specimens by O.L. Zimina, SEM images by A.V. Martynov.

**Figure 7.**
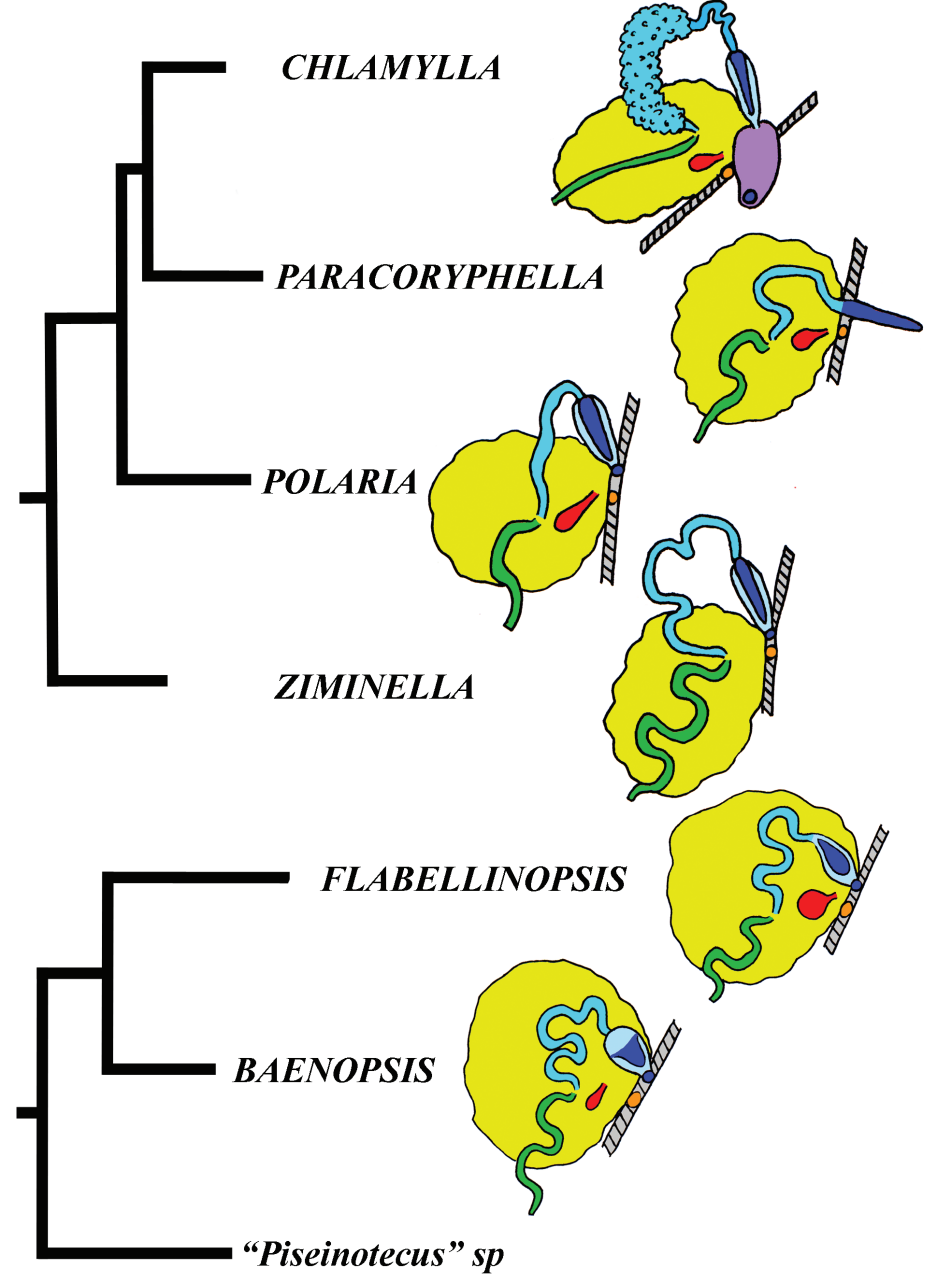
Schematic outline of the reproductive systems of the taxa of the families Paracoryphellidae and Flabellinopsidae integrated with molecular phylogenetic data. Colour indication of reproductive system characters: ampulla – green; body wall – gray; distal receptaculum seminis – red; female gland mass – yellow; female genital opening – orange; penis and male genital opening – dark blue; penial external collar – lilac; penial sheath – pale blue; prostate and prostatic vas deferens – turquoise.

Figs 2 , 3 , 4 , 5 , 6 , 7

##### Type species.

*Chlamylla borealis* Bergh, 1886

##### Diagnosis.

Body wide. Notal edge present, well-defined, continuous. Cerata not stalked, continuous. Rhinophores smooth to wrinkled, longer than oral tentacles. Anterior foot corners absent. Anus pleuroproctic under the notal edge. Rachidian teeth with strong denticulated cusp; lateral denticles not clearly delineated from cusp. Lateral teeth weakly denticulated to smooth without attenuated process basally. Single distal receptaculum seminis. Wide granulated prostate. Thin, long vas deferens clearly separated from prostate. External permanent penial collar. Penis elongated conical, internal.

##### Species included.

*Chlamylla borealis borealis* Bergh, 1886, stat. n. (= *Gonieolis atypica* Bergh, 1899, syn. n.) (Fig. 3) (original description in Bergh 1886, 1899, 1900a), *Ch. borealis orientalis* (Volodchenko, 1941), comb. n. (Fig. 4) (original description in Volodchenko 1941), *Chlamylla intermedia* (Bergh, 1899), comb. n. (Figs 5, 6) (original description in Bergh 1899, 1900a).

##### Remarks.

Bergh (1886) described the genus *Chlamylla* based on the species *Ch. borealis* with a continuous notum, wide granulated prostate, and aberrant radula (lateral teeth with very broad bases, rachidian teeth completely lacking denticles and having instead a pair of unusual long processes (Bergh 1886: Taf. 1, fig. 14). The true identity of the type species *Ch. borealis* with our extensive recent paracoryphellid specimens from the Arctic is a complicated question. Such radular features are completely unknown in the group of the Arctic Paracoryphellidae usually assigned to the genus *Chlamylla* (Roginskaya 1987; Martynov 2006a) Figs 1, 3H, 4H, 5H, 6E). However, the general shape of the body, shape of the jaws, presence of a wide granulated prostate, and penial collar in *Ch. borealis* agree with specimens that are currently assigned to the species *Ch. atypica* and *Ch. intermedia*. Bergh in the same work (1886) described the radula of another species “*Goniaeolis typica*” in detail (Bergh 1886: Taf. 3, fig. 14) as a normal triserial radula. However, Bergh (1886) also noted that the radula of *Ch. borealis* was in a poor condition. This may imply that either Bergh studied an abnormal specimen or had damaged the radula in some way during preparation. For this study we specially investigated the holotype of *Ch. borealis* (NHMD GAS-2055). It is dry and heavily dissected, but a separate cut-off of the external penial collar is left; the penis and damaged jaws are preserved. Comparison of available information from the holotype of *Ch. borealis* with figures from the original description (Bergh 1886: Taf. 1, fig. 22) confirms the presence of an external penial collar with caudal genital fold. According to our data only two paracoryphellid species with a complex folded penial collar are known from Arctic seas and are currently identified as *Chlamylla atypica* and *Ch. intermedia* (Figs 3, 4C, 4I, 5J, 6F, 7). Morphologically these taxa differ from the rest of the traditional flabellinids, forming a distinct compact clade according to the present molecular analysis (Figs 1, 2), which demonstrates significant molecular divergence (more than 11%) from other members of the family Paracoryphellidae. Based on the details in the original description of *Chlamylla* and type material, we can conclude, under supposition that the radula was malformed or wrongly processed, that the type species *Ch. borealis* belongs to the same genus as known paracoryphellid species from the Arctic, currently identified as *Ch. atypica* and *Ch. intermedia*. Given that *Ch. borealis* was never found again, but inhabited the same region our other samples, it is highly likely that *Ch. borealis* is actually conspecific with one of the two currently known *Chlamylla* species. One of these species, *Ch. intermedia* (Bergh, 1899) possesses an external penial collar but without any traces of a caudal genital fold (Fig. 5J), whereas *Ch. borealis*, according to both its original description (Bergh 1886: Taf. 1, fig. 22a) and our novel information from the holotype, possesses a short but evident caudal genital fold. Thus, we can conclude that *Ch. intermedia* cannot be a synonym of *Ch. borealis*. Another species, *Ch. atypica* which was described under the genus *Goniaeolis* from the Davis Strait, Greenland (Bergh 1899, 1900a) readily differs from other *Chlamylla* species by the presence of a special long external genital fold towards the anal opening (Bergh 1899, 1900a: Tab 4, fig. 6) (Fig. 3). Because *Ch. borealis* also possesses a genital fold, and given a high similarity of prostate patterns between our specimens previously identified as *Ch. atypica* (Fig. 3J) and the figure of the reproductive system with the characteristically bent prostate as in *Ch. borealis* in Bergh’s original description of *Ch. borealis* (Bergh 1886, Taf. 1, fig. 21) we therefore conclude that *Ch. atypica* is most likely is a junior synonym of *Ch. borealis*. The differences between length of the genital fold of the holotype of *Ch. atypica* (also investigated in the present study, NHMD GAS-2090) and *Ch. borealis* is possibly due to considerable differences in the length of holotypes of *Ch. borealis* and *Ch. atypica* (the former is nearly two times shorter than the latter). Smaller specimens previously identified as *Ch. atypica* *s. l.* may possess a considerably shorter genital fold, especially in the preserved state (Fig. 4C). Therefore, in order to preserve current usage of the genus *Chlamylla* that has already appeared in a number of publications on Russian nudibranch fauna, we therefore synonymise here the species *Ch. atypica* with *Ch. borealis*.

The Japan Sea specimens are consistent with the Arctic specimens in the presence of the external genital fold, but due to minor differences in the radula and also a very large geographic gap we consider it as a subspecies *Chlamylla borealis orientalis* (Volodchenko, 1941), comb. n. (Fig. 4). *Chlamylla borealis orientalis* is locally abundant during winter in Northern Japan (Hirano 1997, as *Ch. atypica*) and in the Russian part of the Sea of Japan (Martynov, unpublished course work (1991) as *Ch. atypica*; Martynov and Korshunova 2011; present study). Several specimens of other *Chlamylla* without such a fold from the Arctic seas (distributed at least from the Barents Sea to Laptev Sea) have no significant molecular differences between them (Figs 1, 2) and have consistent morphology (Figs 5–6) and clearly belong to the same species. The oldest name for this *Chlamylla* without a genital fold is *Goniaeolis intermedia* Bergh, 1899 also from the Davis Strait, Greenland. The lateral teeth of *Goniaeolis intermedia* with a broad base and slightly attenuated lateral processes (Bergh 1899: tab 4, fig. 16) are similar to our material from the Arctic seas (Figs 5H, 6E). However, *G. intermedia* lacked denticles on the lateral teeth. The Arctic specimens show small, sometimes almost diminishing denticles (Fig. 6E). Such denticles are not always clearly evident. Possibly this species reaches at least the Bering Strait and potentially may enter the coldest shelf waters of the NW Pacific (i.e., Bering and Okhotsk Sea, Martynov 2006a). *Coryphella barentsi* Derjugin, 1924 (Derjugin 1924a, b, preoccupied by *Coryphella barentsi* Vayssière, 1913 (Vayssière 1913, see Gosliner and Griffiths 1981 suggesting a replacement name) is a possible synonym of *Ch. intermedia* (Bergh, 1899).

#### Paracoryphella

Taxon classificationAnimaliaNudibranchiaParacoryphellidae

Miller, 1971

**Figure 8.**
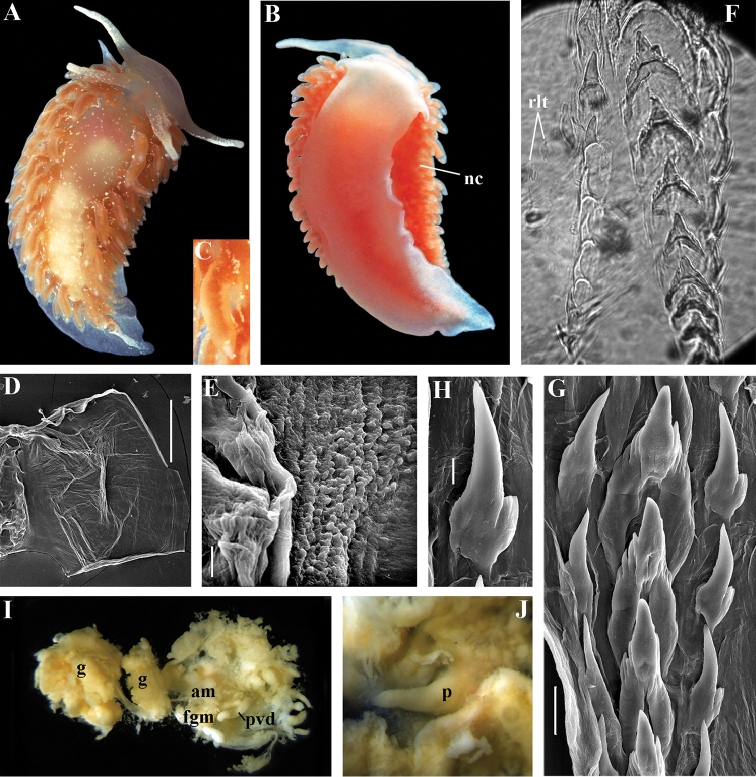
*Paracoryphella ignicrystalla* sp. n. ZMMU Op-490. The Sea of Japan, Vostok Bay, living specimen 11.5 mm in fixed length: **A** dorsal view **B** ventral view **C** details of cerata **D** jaw, SEM **E** details of masticatory process of jaw, SEM **F** radular teeth, posterior part, showing second rudimentary row of lateral teeth (**rlt**), light microscopy **G** radular teeth, posterior part, SEM **H** lateral tooth, close up, SEM **I** dissected anterior part showing pharynx and reproductive system **J** ventral anterior part of fixed specimen showing external non-retractable penis (**p**). Abbreviations: **am** ampulla **fgm** female gland mass **g** gonad **nc** continuous notal edge **p** penis **pvd** prostatic vas deferens **rlt** rudimentary lateral teeth (second additional rudimentary row of lateral teeth); Scale bars: **D** = 100 μm; **E, G** = 30 μm; **H** = 10 μm. Photos and SEM images by A.V. Martynov.

**Figure 9.**
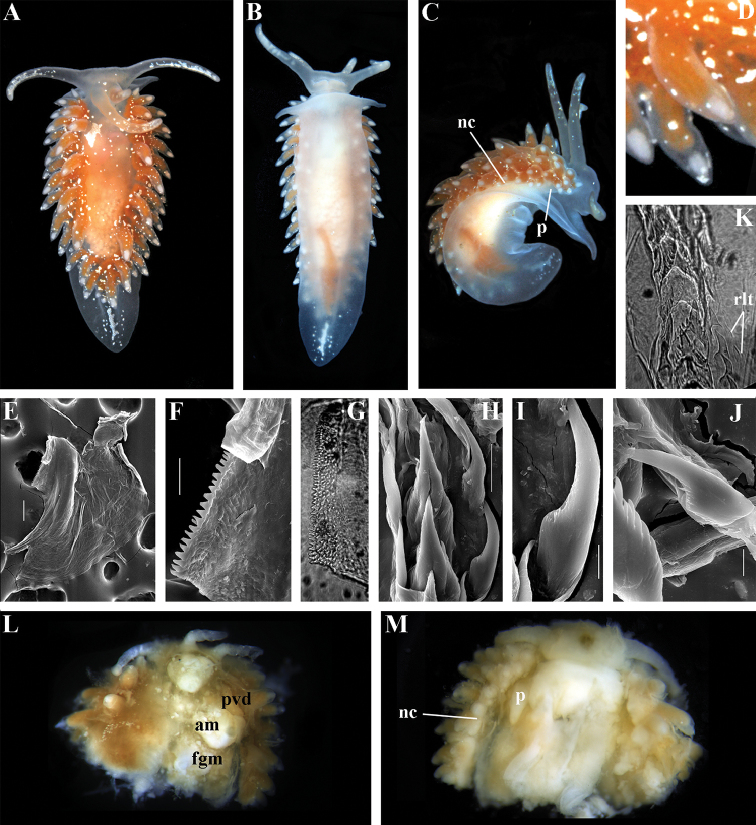
*Paracoryphella islandica* (Odhner, 1937). ZMMU Op-534. Barents Sea, Dalne-Zelentskaya Bay, living specimen 12 mm in fixed length: **A** dorsal view **B** ventral view **C** lateral view **D** same, details of cerata **E** jaw, SEM **F** details of masticatory process of jaw, SEM **G** details of masticatory process of jaw, light microscopy **H** radular teeth, posterior part, SEM **I** lateral tooth, close up, SEM **J** rachidian and lateral teeth, details, SEM **K** radular teeth, middle part, showing second rudimentary row of lateral teeth (rlt), light microscopy **L** dissected anterior part showing pharynx and reproductive system **M** ventral anterior part of fixed specimen showing external non-retractable penis (p). Abbreviations: **am** ampulla **fgm** female gland mass **nc** continuous notal edge **p** penis **pvd** prostatic bas deferens **rlt** rudimentary lateral teeth (second additional rudimentary row of lateral teeth) . Scale bars: **E** = 100 μm; **F, H** = 30 μm; **I, J** = 10 μm. Photos and SEM images by T.A. Korshunova, A.V. Martynov.

**Figure. 10.**
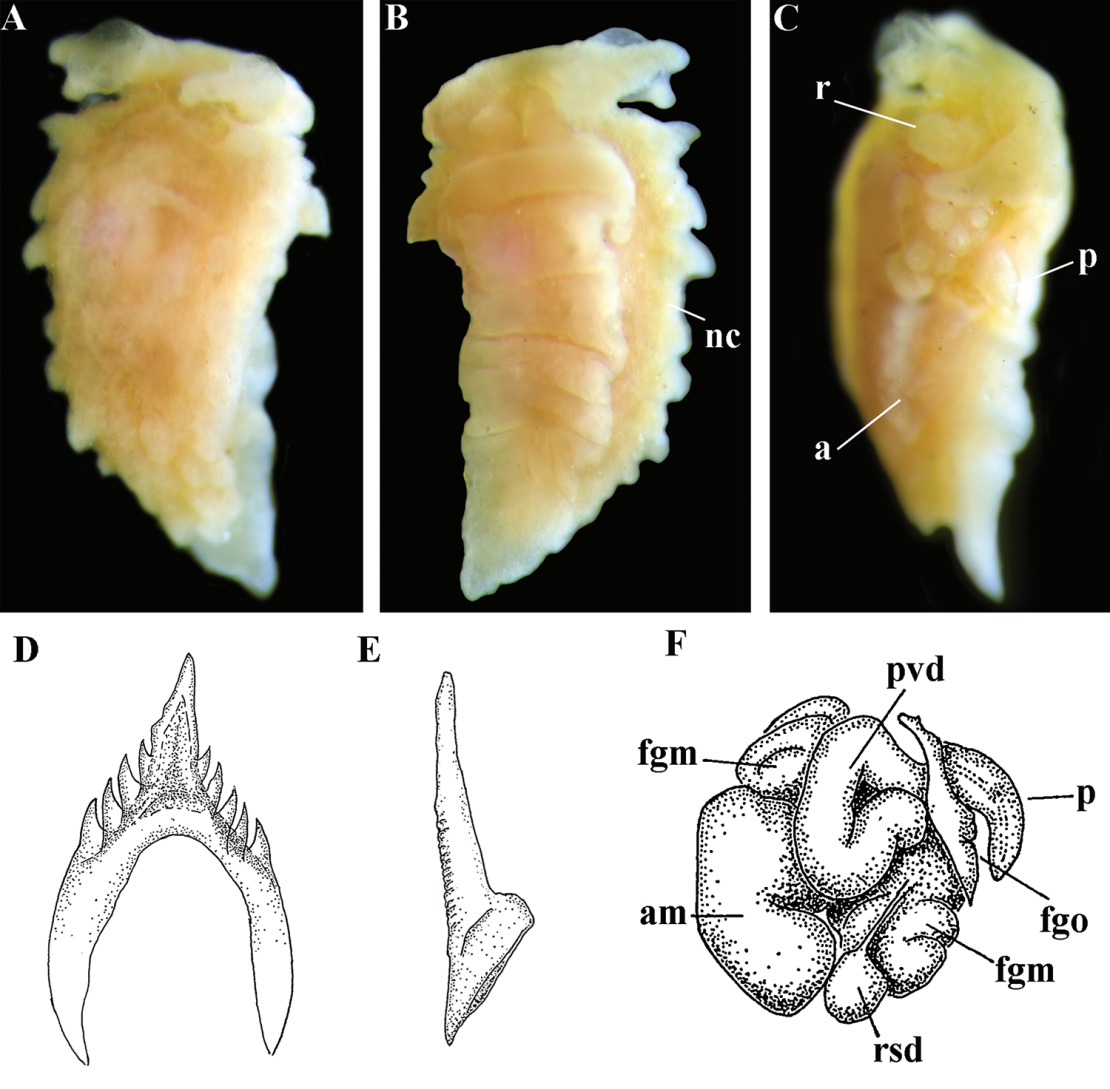
*Paracoryphella parva* (Hadfield, 1963), comb. n. Holotype NHMD-91476, 2 mm length: **A** fixed animal, dorsal view **B** fixed animal, ventral view of anterior part **C** fixed animal, lateral view **D** rachidian radular teeth **E** lateral teeth **F** reproductive system. Abbreviations: **a** anus **am** ampulla **fgm** female gland mass **fgo** female genital opening **nc** continuous notal edge **p** penis **pvd** prostatic vas deferens **r** rhinophores **rsd** distal receptaculum seminis. Photos by A.V. Martynov. Drawings **D, E, F** from original description of *P. parva* from Hadfield (1963).

Figs 2 , 7 , 8 , 9 , 10

##### Type species.

*Coryphella islandica* Odhner, 1937

##### Diagnosis.

Body wide. Notal edge present, well-defined, continuous. Cerata not stalked, continuous. Rhinophores smooth to wrinkled, shorter than or similar in size to oral tentacles. Anterior foot corners present. Anus pleuroproctic under the notal edge. Rachidian teeth with strong cusp; lateral denticles not clearly delineated from cusp. Lateral teeth weakly denticulated without attenuated process basally. Reduced additional rows of of small lateral teeth may present. Single distal receptaculum seminis. Long vas deferens without separate granulated prostate. Penis not internal, permanently attached externally.

##### Species included.

*Paracoryphella ignicrystalla* sp. n. (Fig. 8), *P. islandica* (Odhner, 1937) (Fig. 9) (original description in Odhner 1937), *Paracoryphella parva* (Hadfield, 1963), comb. n. (original description in Hadfield 1963) (Fig. 10).

##### Remarks.

The genus *Paracoryphella* and the family Paracoryphellidae were initially proposed by Miller (1971) because of the putative presence of a second asymmetrical row of lateral teeth in the original description of the species “*Coryphella*” *islandica* (see Odhner 1937). However, the latter character is not very evident compared to the true unique feature of the genus *Paracoryphella*, a non-retractable, permanently external penis, which is attached directly to the body wall and does not possesses any penial sheath (Figs 8J, 9M, 10C). Importantly, all three known species of this genus invariably possess this feature. This character is unique not only within traditional flabellinids, but also within the majority of Aeolidacea. Only a single species of the family Notaeolidiidae also has such an external penis (Wägele 1990). Furthermore, several members of the notaspid family Pleurobranchidae with an internal shell (Martynov and Schrödl 2008) and very basal Acteonidae with an external solid shell also possess an external penis. Therefore, this character within the genus *Paracoryphella* may be either a basal plesiomorphy, or an ontogenetic reversion to the basal plesiomorphy. Molecular data shows that in either case it occurs within one of the most basal clades of the traditional flabellinids. Additionally, analysis of the light microscopy images of the radula of both the type species *P. islandica* and the new species confirm the possible presence of 1–3 very reduced, asymmetrically placed, additional rows of lateral teeth (Figs 8F, 9K). During preparation for the SEM study these apparently reduced additional teeth became fully indistinguishable. Their correspondence to the normal lateral teeth needs to be further investigated.

*Coryphella parva*, only known from its original description from Swedish waters (Hadfield 1963), was described as having a permanent external penis and therefore is included here in the genus *Paracoryphella*. Here, for the first time, we have studied type material of *C. parva* from the Natural History Museum of Denmark (NHMD-91476) (Fig. 10) and confirmed that it possesses a non-retractable external penis (Fig. 10C) and other external features that align with the diagnosis of the genus *Paracoryphella*. The drawings of the radula (Fig. 10D, E) and reproductive systems (Fig. 10F) in the original description of *P. parva* in Hadfield (1963) are also very consistent with two other species of the genus *Paracoryphella*. Remarkably, even though the length of living specimens of *P. parva* do not exceed 3.5 mm (fixed not more than 2 mm, Fig. 10A, B), the animals at that size were fully mature and produced egg masses (Hadfield 1963). Because both *P. ignicrystalla* sp. n. and the type species of the genus *P. islandica* reach mature size in specimens at least three times larger than *P. parva*, the latter species thus is clearly a separate one and may represent an example of a partial paedomorphosis. According to the molecular phylogenetic analysis, the genus *Paracoryphella* is the sister of the *Chlamylla* clade (Figs 1, 2, 7). We retain the genus *Paracoryphella* as separate because of unique morphological characteristics including a permanent external penis and also the apparent presence of additional rudimentary lateral teeth rows.

#### Paracoryphella ignicrystalla sp. n.

Taxon classificationAnimaliaNudibranchiaParacoryphellidae

http://zoobank.org/3C94E2E9-C880-40F0-9557-3B39C339B7C0

Fig. 8

##### Type material.

Holotype, ZMMU Op-490, 11.5 mm long (fixed), The Sea of Japan, Vostok Bay, intertidal, 17.03.1994, coll. A.V. Martynov. 1 paratype, ZMMU Op-491, 5 mm long (fixed, dissected), The Sea of Japan, Vostok Bay, intertidal, 14.03.1994, coll. A.V. Martynov. 1 paratype, ZMMU Op-492, 12 mm long (fixed, dissected), The Sea of Japan, Vostok Bay, intertidal, 18.02.1990, coll. A.V. Martynov.

##### Type locality.

The Sea of Japan, Vostok Bay.

##### Etymology.

From *igni* (= fire, Latin) and *crystallum* (= ice, rock crystal, Latin), in reference to the double combination of peculiar morphological and ecological features: short flame-like cerata with icy speckles on dorsum and peculiar environmental characteristics of the type locality which combines icy sea water temperatures (down to -2 °C) in winter and warm subtropical conditions in summer (water temperature up to +26 °C) as an allusion to the George R. R. Martin “A Song of Ice and Fire” novels.

##### Diagnosis.

Continuous notal edge, colour translucent white with scattered opaque white dots, cerata orange-brown to reddish-brown, rachidian tooth with up to 12 denticles not clearly delineated from relatively low central cusp, lateral teeth with few distinct basal denticles, distal receptaculum seminis, penis not internal, permanently attached externally.

##### Description.

*External morphology*. Body wide. Foot and tail wide, anterior foot corners short. Oral tentacles long. Rhinophores ca. 1.5 times shorter than oral tentacles, smooth to slightly wrinkled. Dorsal cerata fusiform, relatively short, continuously attached to well-defined uninterrupted notal edge without forming clusters. Apices of cerata pointed. Notum narrow but distinct throughout both lateral sides of body. Digestive gland diverticulum fills significant volume of the cerata. Anal opening on right side below notal edge close to middle body part. Reproductive openings lateral and non-retractable penis below second ceratal row. Tail short and pointed, extending only a short distance beyond last cerata.

*Colour* (Fig. 8A). Background colour translucent white. Digestive gland diverticula orange-brown to reddish-brown. Small opaque white spots cover the entire dorsum, commonly on ceratal bases and less on cerata. Rhinophores and oral tentacles similar in colour to body; apical parts covered with opaque white pigment. Apical parts of cerata without opaque cap of white pigment.

*Jaws* (Fig. 8E). Masticatory process more than one-third as long as jaw body. Edge of masticatory processes bears ca. 40–50 denticles that continue to form several reduced rows of denticles on the body of the masticatory processes.

*Radula* (Fig. 8F, H). Radula formula: 10–12 × 1.1.1(2–3). Rachidian tooth elongate-triangular with strong non-compressed cusp of nealy 1/3 of the tooth length (Fig. 8G). Rachidian tooth bears up to 15 well-defined separated (but adpressed towards the cusp) long lateral denticles. Cusp is not clearly delineated from the adjacent first lateral denticles. Lateral teeth (Fig. 8F, H) narrowly triangular with peculiar widened base and few indistinct denticles on internal edge. There are one to three rudimentary additional lateral teeth on the right side only.

*Reproductive system* (Fig. 8I, J). Diaulic. Hermaphroditic duct leads to strong convoluted ampulla of about two whorls. Vas deferens is relatively long, no distinct prostate. No penial sheath. Penis is attached to the external body wall, vas deferens enters the base of penis from the internal side. Oviduct connects through insemination duct into female gland complex. Vagina short and indistinct. Distal receptaculum seminis.

##### Ecology.

Stony intertidal to 5–6 m. Feeds on athecate solitary hydroids. This species is locally abundant. Egg mass is white to pinkish narrow cord. Reproduction period from December to April. Development is about one month. The larva is a planktotrophic veliger with spiral shell.

##### Distribution.

Northwest part of the Sea of Japan.

##### Remarks.

*Paracoryphella ignicrystalla* sp. n. clearly differs from the type species of the genus *P. islandica* (Fig. 9K, H) in having considerably shorter cusps of the rachidian teeth (Fig. 8F, G). We have not yet obtained molecular data for this Sea of Japan *Paracoryphella*, but regard the morphological differences sufficient to warrant a new species.

#### Polaria gen. n.

Taxon classificationAnimaliaNudibranchiaParacoryphellidae

http://zoobank.org/716D049A-DF2B-489C-ABC8-B76B1E830073

Figs 2 , 7 , 11

##### Type species.

*Coryphella polaris* Volodchenko, 1946

##### Etymology.

After the northern Polar region, the predominant area of distribution of this genus.

##### Diagnosis.

Body wide. Notal edge present, well-defined, continuous. Cerata not stalked, continuous. Rhinophores smooth to wrinkled, longer than oral tentacles. Anterior foot corners present. Anus pleuroproctic under the notal edge. Rachidian teeth with strong smooth cusp and distinct denticles. Lateral teeth strongly denticulated with considerably attenuated process basally. Single distal receptaculum seminis. Long vas deferens without separate granulated prostate. No penial collar. Penis elongated conical.

##### Species included.

**Figure 11.**
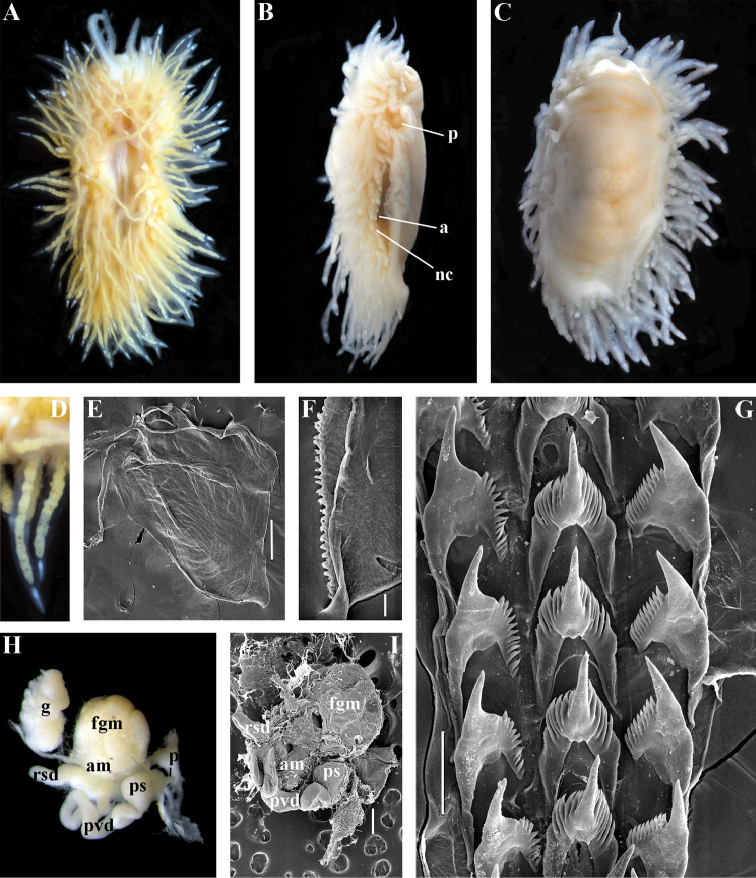
*Polaria polaris* (Volodchenko, 1946), comb. n. ZMMU Op-519. Laptev Sea, living specimen 12 mm in length: **A** dorsal view **B** latero-ventral view **C** ventral view **D** details of cerata **E** jaw, SEM **F** details of masticatory process of jaw, SEM **G** radular teeth, posterior part, SEM **H** reproductive system, light microscopy **I** reproductive system, SEM. Abbreviations: **a** anus **am** ampulla **rsd** distal receptaculum seminis **fgm** female gland mass; **g** gonad; **nc** continuous notal edge **p** penis **ps** penial sheath **pvd** prostatic vas deferens Scale bars: **E, I** = 300 μm; **F** = 30 μm; **G** = 100 μm. Photos of living specimens by O.L. Zimina, other photos and SEM images by A.V. Martynov.

*Polaria polaris* (Volodchenko, 1946), comb. n. (Figs 7, 11) (original description in Volodchenko 1946).

##### Remarks.

The type and single species of the genus *Polaria*, *Coryphella polaris* is the only available valid name (Volodchenko 1946) for the species *Goniaeolis typica* M. Sars, 1861 as incorrectly identified by Bergh (1886), Odhner (1907), and Roginskaya (1987, 1997). True *Goniaeolis typica* possesses a triserial radula but has no digestive gland branches or cnidosacs in the cerata and the shape of radular teeth is very different from any traditional flabellinid (M. Sars 1861; G.O. Sars 1872; Odhner 1922). The name *Coryphella polaris* Voldochenko, 1946 was therefore resurrected to avoid this misidentification (Martynov 2006a). The genus *Polaria* forms a separate clade within Paracoryphellidae according to the molecular phylogenetic analysis (Figs 1, 2). By combination of relatively long vas deferens without distinct prostate (Figs 7, 10H, I), retractable penis without external collar, and lateral teeth with strongly attenuated process (Fig. 10G), the genus *Polaria* morphologically differs from all other genera of the family Paracoryphellidae.

#### Ziminella gen. n.

Taxon classificationAnimaliaNudibranchiaParacoryphellidae

http://zoobank.org/62A50536-3E18-4A03-8FD2-B27D43357628

**Figure 12.**
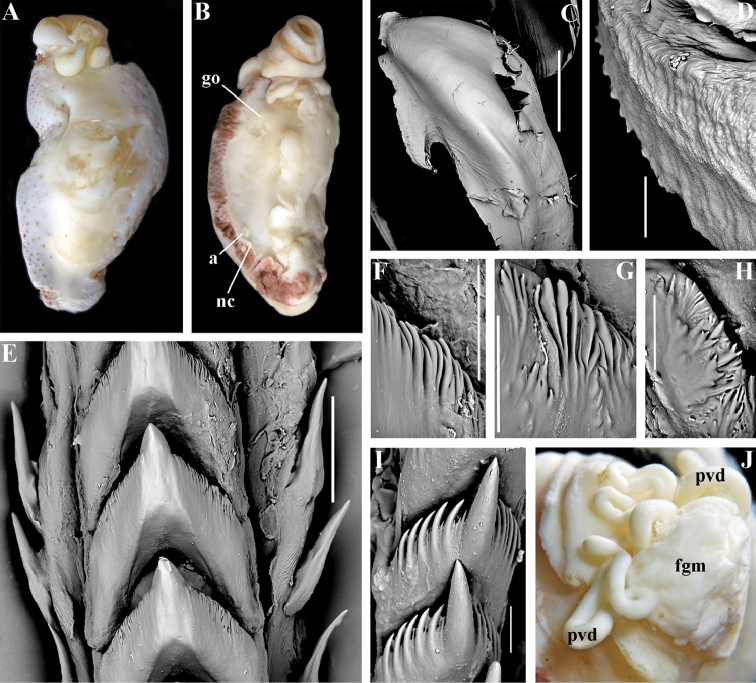
*Ziminella abyssa* sp. n.: **A** preserved holotype (Sea of Japan, ZSM Mol-20100647, 2676 m), dorsal view, 19 mm in fixed length **B** same, latero-ventral view **C** jaw, SEM ZMMU Op-259, depth 3460 m **D** details of masticatory process of jaws **E** radular teeth, posterior part, SEM ZMMU Op-250, 24 mm length, 3300 m **F–H** details of three central teeth with additional or highly aberrant lateral denticles ZMMU Op-248, 12 mm length, 3560 m **I** rachidian tooth of juvenile specimen 2 mm length showing additional lateral denticles ZMMU Op-264, 1525 m **J** reproductive system, light microscopy ZSM Mol-20100644, 3213 m. Abbreviations: **a** anus **fgm** female gland mass **go** genital opening **nc** continuous notal edge **pvd** prostatic vas deferens. Scale bars: **C** = 1 mm; **E** = 50 μm; **F, G, H** = 300 μm. Photos and SEM images by A.V. Martynov.

**Figure 13.**
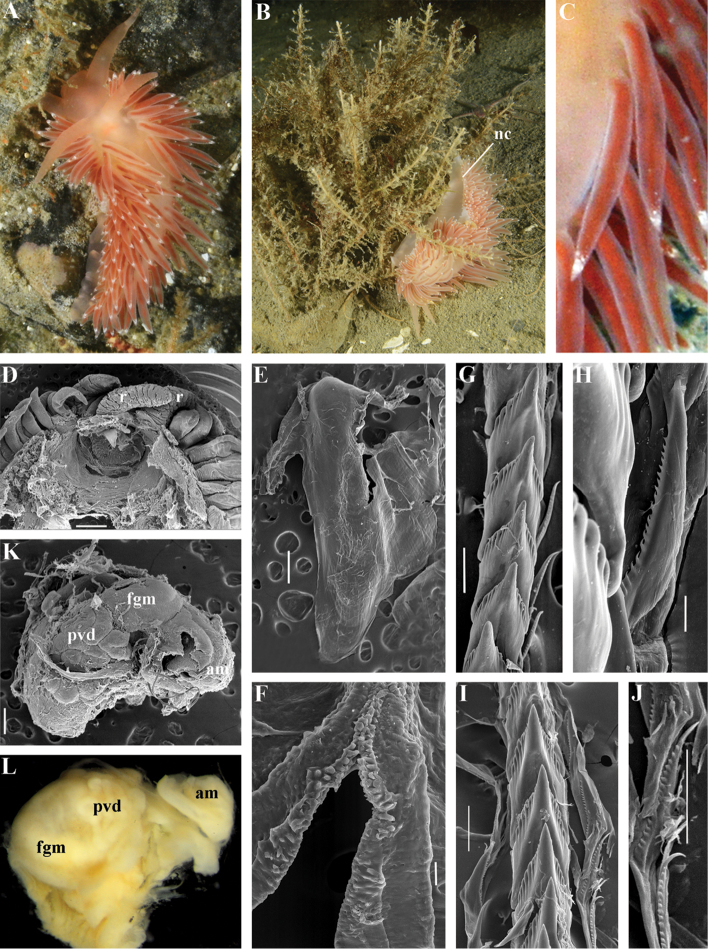
*Ziminella circapolaris* sp. n.: Barents Sea, Franz-Josef Land: **A** living animal, dorsal view ZMMU Op-482 **B** living animal on a hydroid colony ZMMU Op-483, 12 mm in fixed length **C** same, details of cerata **D** dissected anterior part (pharynx removed) and rhinophores, SEM **E** jaw, SEM **F** details of masticatory process of jaw, SEM **G** radular teeth, posterior part, SEM ZMMU Op-482 **H** lateral tooth, close up, SEM ZMMU Op-482 **I** radular teeth, posterior part, SEM ZMMU Op-483 **J** lateral tooth, close up, SEM ZMMU Op-483 **K** reproductive system, SEM ZMMU Op-483 **L** reproductive system, light microscopy. Abbreviations: **am** ampulla **fgm** female gland mass **nc** continuous notal edge **pvd** prostatic vas deferens **r** rhinophores. Scale bars: **D** = 1 mm; **E, K** = 300 μm; **F, H** = 30 μm; **G, I, J** = 100 μm. Photos of living specimens by O.V. Savinkin, other photos and SEM images by A.V. Martynov.

**Figure 14.**
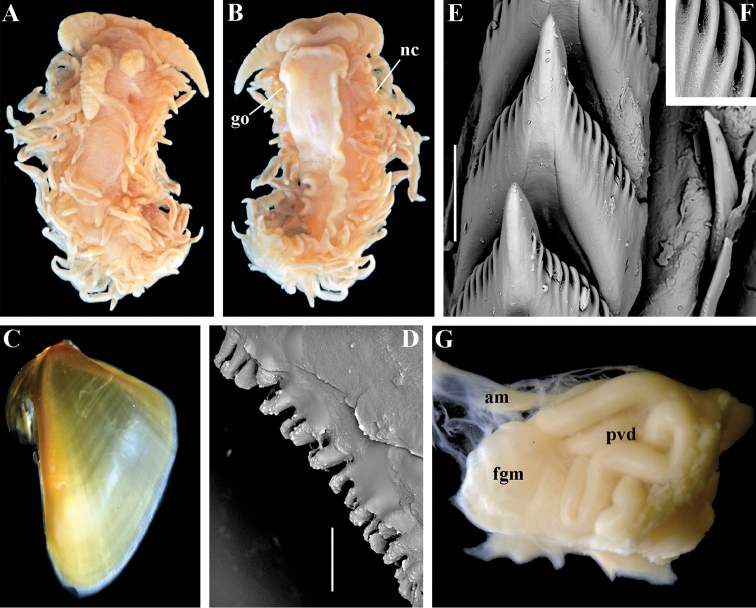
*Ziminella japonica* (Volodchenko, 1941), comb. n.: **A** ZMMU Op-261, (Sea of Japan, 470–528 m) dorsal view, 8.3 mm in fixed length **B** same, ventral view **C** jaw, light microsocopy **D** details of masticatory process of jaws, SEM **E** radular teeth, posterior part **F** details of rachidian radular tooth **G** reproductive system, light microscopy. Abbreviations: **am** ampulla **fgm** female gland mass **go** genital opening **nc** continuous notal edge **pvd** prostatic vas deferens. Scale bars: **D** = 30 μm; **E** = 100 μm. Photos and SEM images by A.V. Martynov.

**Figure 15.**
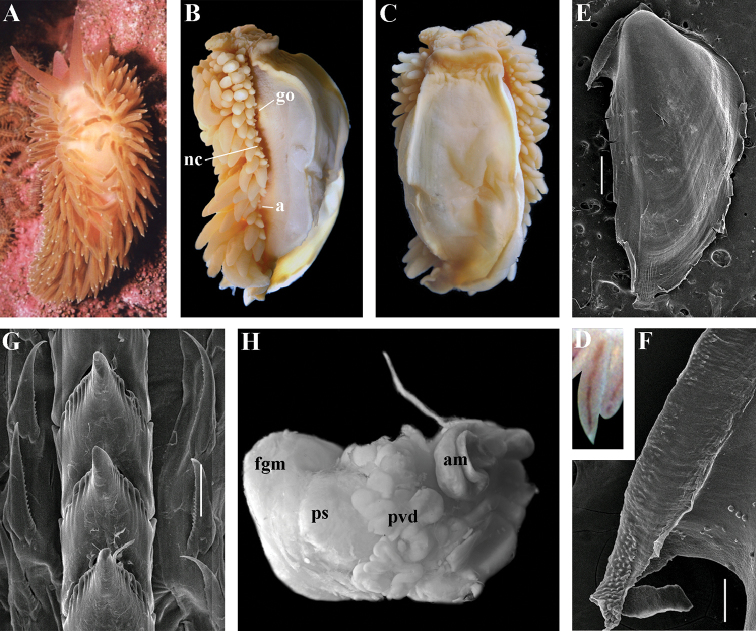
*Ziminella salmonacea* (Cothouy, 1839), comb. n.: **A** living animal (Spitzbergen), dorsal view **B** preserved animal, lateral view ZMMU Op-265, 19 mm preserved **C** same, ventral view **D** details of cerata **E** jaw, SEM **F** details of masticatory process of jaw, SEM **G** radular teeth, posterior part, SEM **H** reproductive system, light microscopy. Abbreviations: **a** anus **am** ampulla **fgm** female gland mass **go** genital opening **nc** continuous notal edge **ps** penial sheath **pvd** prostatic vas deferens. Scale bars: **E** = 300 μm; **F** = 30 μm; **G** bar = 100 μm. Photos of living specimens by B. Gulliksen, other photos and SEM images by A.V. Martynov.

Figs 2 , 12 , 13 , 14 , 15

##### Type species.

*Eolis salmonacea* Couthouy, 1838

##### Etymology.

In honour of Olga Zimina, scientist at Murmansk Marine Biology Institute; she made a considerable contribution in collecting Arctic paracoryphellid species for this study.

##### Diagnosis.

Body wide. Notal edge present, well-defined, continuous. Cerata not stalked, continuous. Rhinophores smooth to wrinkled, similar in size to oral tentacles. Anterior foot corners present. Anus pleuroproctic under the notal edge. Rachidian teeth with strong denticulated cusp; lateral denticles not clearly delineated from cusp. Lateral teeth weakly denticulated to smooth without attenuated process basally, significantly smaller than rachidian teeth. Receptaculum seminis not evident. Long vas deferens without separate granulated prostate. No penial collar. Penis folded or elongated conical.

##### Species included.

*Ziminella abyssa* sp. n. (Fig. 12), *Ziminella circapolaris* sp. n. (Fig. 13), *Ziminella japonica* (Volodchenko, 1941), comb. n. (Fig. 14) (original description in Volodchenko 1941; lectotype designated in Martynov 2013), *Ziminella salmonacea* (Couthouy, 1838), comb. n. (Fig. 15) (original description in Couthouy 1838; redescription in Kuzirian 1979).

##### Remarks.

*Ziminella* considerably differs morphologically from the genus *Chlamylla* by the absence of a granulated prostate and external penial collar (Fig. 7) and by the shape of the radular teeth, from the genus *Paracoryphella* by the presence of a penial sheath and by the shape of the radular teeth, from the genus *Polaria* by the absence of a very long, conspicuous distal receptaculum seminis and by the shape of the radular teeth. On the molecular tree, both species of the genus *Ziminella* place as a well-separated clade within the family Paracoryphellidae (Figs 1, 2). Four species currently known within the genus *Ziminella* are well-separated by the penial morphology (*Z. salmonacea* possesses a slightly folded penis, whereas *Z. abyssa* and *Z. japonica* have an entire conical penis; for discussion see Martynov 2013). Molecular data (Figs 1, 2) published here for the first time show that three species are placed together within a larger clade.

#### Ziminella abyssa sp. n.

Taxon classificationAnimaliaNudibranchiaParacoryphellidae

http://zoobank.org/B22A7B85-F09B-4604-BE8C-6D5B560ABBDD

Fig. 12

##### Type material.

Holotype, ZSM Mol-20100647, 19 mm long (fixed), R/V ‘‘Akademik Lavrentyev”, sta. B5–10, 24.08.2010, The Sea of Japan, depth 2676 m. 8 paratypes, ZMMU Op-248, up to 20 mm long (fixed), R/V ‘‘Vityaz’’, sta. 6657, 15.06.1972, The Sea of Japan, depth 3560 m. 9 paratypes, ZMMU Op-249, up to 24 mm long (fixed), R/V ‘‘Vityaz’’, sta.7462, 29.05.1976, The Sea of Japan, 41°19.8'N, 131°15.2'E, depth 3290 m. 57 paratypes, ZMMU Op-250, up to 25 mm long (fixed), R/V ‘‘Vityaz’’, sta. 7463, 29.05.1976, The Sea of Japan, 40°45.0'N, 131°16.0'E, depth 3300 m. 1 paratype, ZMMU Op-252, 9 mm long (fixed), R/V ‘‘Vityaz’’, sta. 7476, 06.06.1976, The Sea of Japan, 40°39.5'N, 132°32.8'E, depth 3425 m. 7 paratypes, ZMMU Op-253, up to 15 mm long (fixed), R/V ‘‘Vityaz’’, sta. 7479, 07.06.1976, The Sea of Japan, 39°09.1'N, 131°03.2'E,, depth 3120 m. 2 paratypes, ZMMU Op-255, up to 14 mm long (fixed), R/V ‘‘Vityaz’’, sta. 7492, 12.06.1976, The Sea of Japan, 38°21.8'N, 134°49.5'E, depth 3020–3030 m. 2 paratypes, ZMMU Op-256, up to 19 mm long (fixed), R/V ‘‘Vityaz’’, sta. 7494, 14.06.1976, The Sea of Japan, 38°48.0'N, 136°04.6'E, depth 2740 m. Two paratypes, ZMMU Op-257, 15 mm long (fixed), R/V ‘‘Vityaz’’, sta. 7496, 01.07.1976, The Sea of Japan, 40°36.60'N, 139°00.002'E, depth 3340 m. 48 paratypes, ZMMU Op-258, up to 21 mm long (fixed), R/V ‘‘Vityaz’’, sta. 7517, The Sea of Japan, 42°28.2'N, 138°20.9'E, depth 3620 m. 6 paratypes, ZMMU Op-259, up to 18 mm long (fixed), R/V ‘‘Vityaz’’, sta. 7519, 02.06.1976, The Sea of Japan, 41°28.8'N, 136°06.1'E, depth 3460 m. 11 paratypes, ZMMU Op-264, up to 12 mm long (fixed), R/V ‘‘Akademik Lavrentyev’’(SoJaBio), sta. A3–11, 14.06.2010, The Sea of Japan, 44°47.6338'N, 137°15.3182'E, depth 1494–1525 m.

##### Type locality.

The Sea of Japan.

##### Etymology.

From *abyssum* (= depth, Latin) in reference to abyssal habitat of the new species, one of the deepest among aeolidacean nudibranchs.

##### Diagnosis.

Continuous notal edge, lateral branches of digestive gland (ceratal basis) dark violet, rachidian tooth with up to 30 (and more) fold-like or fork-shaped denticles clearly delineated from central cusp, lateral teeth with few denticles on teeth edge, receptaculum seminis not evident, penis elongate conical.

##### Description.

*External morphology* (Fig. 12A, B). Body wide. Foot and tail wide, anterior foot corners short. Rhinophores similar in size to oral tentacles, smooth to slightly wrinkled. Dorsal cerata elongate, thin, continuously attached to well-defined uninterrupted notal edge without forming clusters. Apices of cerata pointed. Notum narrow but distinct throughout both lateral sides of body. Digestive gland diverticulum fills significant volume of the cerata. Anal opening on right side below notal edge close to middle body part. Reproductive openings on right side. Tail long and pointed, extending only short distance beyond last cerata

*Colour*. Background colour translucent milky white. Digestive gland diverticula probably reddish. Rhinophores light orange at the bases and pale on most of the rest of the length. Lateral branches of digestive gland (that shine through lateral dorsum sides) characteristically dark violet (estimated from a field colour photograph).

*Jaws* (Fig. 12C, D). Jaws broad, yellowish in colour. Masticatory processes of jaws covered with several compound denticles.

*Radula* (Fig. 12E–H). Radula formula up to 40 × 1.1.1 (in adult specimen 20–24 mm in length). Rachidian tooth with strongly protracted non-compressed cusp of ca. 1/3 of the tooth length (Fig. 12E). Rachidian tooth bears up to 30 (and more) special, fold-like or fork-like lateral denticles, which are sometimes greatly disordered. Larger denticles typically intermingled with the smaller ones. Cusp is clearly delineated from the adjacent first lateral denticles. Lateral teeth (Fig. 12F–H) narrowly triangular with few small denticles on internal edge.

*Reproductive system* (Fig. 12J). Diaulic. Hermaphroditic duct leads to long, relatively narrow convoluted ampulla of several whorls. Vas deferens extremely long, without distinct prostate. Penial sheath elongated. Penis entire, conical. Oviduct connects through insemination duct into female gland complex. No distal or proximal receptaculum seminis detected.

##### Ecology.

Deep sea basins at 1494–3620 m depth, soft bottom. This species is most common in the deepest parts of the Sea of Japan at about 3000 m. Upper bathymetric limit needs to be refined. Feeds on sea anemones of the family Edwardsiidae.

##### Distribution.

Central deepest basins of the Sea of Japan.

##### Remarks.

By presence of an entire copulative organ *Z. abyssa* sp. n. is similar to *Z. japonica* (Volodchenko, 1941), but clearly differs in the pattern of the rachidian radular teeth. While *Z. japonica* has regular lateral denticles of the rachidian teeth, all similar in size, no more 20 in number even in very large specimens (30 mm), *Z. abyssa* sp. n. has highly irregular lateral denticles of the rachidian teeth, different in size, fold- and fork-shape (Fig. 12F, G), sometimes even placed disorderly (Fig. 12H), up to at least 30 (and more) in number even in specimens which are much smaller than in the previous species with specimens reaching 20–24 mm in length. Remarkably, the irregularity of the rachidian teeth in *Z. abyssa* sp. n. persists even in very small juveniles (2 mm), in which larger denticles already intermingle with the smaller ones (Fig. 12I). The morphological differences between these two species agree with the considerable bathymetric differences: *Z. japonica* is a shelf and upper bathyal species (ca. 100–500 m), whereas *Z. abyssa* sp. n. inhabits predominantly the deepest (approximately 3000 m) basins of the Sea of Japan.

#### Ziminella circapolaris sp. n.

Taxon classificationAnimaliaNudibranchiaParacoryphellidae

http://zoobank.org/E376DC16-2736-4109-B09F-81B77B326A0B

Fig. 13

##### Type material.

Holotype, ZMMU Op-598, 35 mm long (fixed), Arctic Ocean, Franz Josef Land, Wiltona Island, 23.08.2013, depth 18–25 m, collected by O.V. Savinkin. 1 paratype, ZMMU Op-482, 14 mm long (fixed), Arctic Ocean, Franz Josef Land, Northbrook Island, 26.08.2013, depth 18–23 m, collected by O.V. Savinkin. 1 Paratype, ZMMU Op-483, 12 mm long (preserved), Arctic Ocean, Franz Josef Land, Pioneer Island, 17.08.2013, depth 21–23 m, collected by O.V. Savinkin.

##### Type locality.

Franz Josef Land.

##### Etymology.

From *circa* (= near, Latin) and *polaris* (= polar, Latin) in reference to the proximity of the habitat of the new species (Franz Josef Land) to the North Pole.

##### Diagnosis.

Continuous notal edge, colour yellowish, cerata reddish-brown, few apical white dots, rachidian tooth with up to ten denticles clearly delineated from central cusp, lateral teeth with numerous denticles (up to 24) which cover whole edges of lateral teeth, receptaculum seminis not evident, penis folded.

##### Description.

*External morpholog*y. Body wide. Foot and tail wide, anterior foot corners short. Rhinophores similar in size to oral tentacles, smooth to slightly wrinkled. Dorsal cerata elongate, thick, continuously attached to well-defined uninterrupted notal edge without forming clusters. Notum narrow but distinct throughout both lateral sides of body. Digestive gland diverticulum fills significant volume of cerata. Anal opening on right side below notal edge close to middle body part. Reproductive openings on right side. Tail long and pointed, extending only a short distance beyond last cerata.

*Colour* (Fig. 13A, B). Background colour yellowish-white. Digestive gland diverticula reddish-brown. Rhinophores light orange-yellowish. Lateral branches of digestive gland (that shine through lateral dorsum sides) not distinct.

*Jaws* (Fig. 13E, F). Jaws broad, yellowish in colour. Masticatory processes of jaws covered with several simple denticles.

*Radula* (Fig. 13G–J). Radula formula up 26 × 1.1.1 (specimens 20–24 mm in length). Rachidian tooth elongate with strongly protracted, pointed, non-compressed cusp of almost 1/3 of tooth length (Fig. 13G, I). Rachidian tooth bears five to ten lateral denticles. Larger denticles not intermingled with smaller ones. Cusp clearly delineated from the adjacent first lateral denticles. Lateral teeth (Fig. 13H, J) narrowly triangular with 19–24 denticles on internal edge.

*Reproductive system* (Fig. 13K, L). Diaulic. Hermaphroditic duct leads to long, relatively narrow convoluted ampulla of several whorls. Vas deferens long, without distinct prostate. Penial sheath elongated. Penis folded. Oviduct connects through insemination duct into female gland complex. No distal or proximal receptaculum seminis detected.

##### Ecology.

Soft bottom with stones 18 to 23 m.

##### Distribution.

Franz Josef Land.

##### Remarks.

According to the molecular phylogenetic analysis *Ziminella circapolaris* sp. n. forms a separate sister clade to *Z. salmonacea* (Fig. 1). Specimens from the West Atlantic North American coast (type locality of *Z. salmonacea*) and Spitzbergen belong to *Z. salmonacea*, according to molecular data, whereas specimens from Franz Josef Land belong to the separate species *Z. circapolaris* sp. n. This is concordant with a considerable level of endemism of the nudibranch fauna of Franz Josef Land recently demonstrated for other nudibranch groups (Martynov and Korshunova 2017). Morphological analysis reveals differences in the denticulation of the lateral teeth between *Z. salmonacea* and *Z. circapolaris* sp. n.: in the first species denticulation on the lateral teeth tends to be restricted to the first half of the length of lateral teeth or the teeth can be completely smooth, whereas in the new species denticulation runs up to the very end of the lateral teeth, and the teeth are always denticulated. Kuzirian (1979) studied the radulae of 15 specimens of *Z. salmonacea* from near the type locality and all of them have considerably smoother lateral teeth than *Z. circapolaris* sp. n.

#### Family Baenopsis fam. n.

Taxon classificationAnimaliaNudibranchiaFlabellinopsidae

http://zoobank.org/F3E7E3B7-E77F-484A-B629-FFDDA9FDCD59

##### Diagnosis.

Body relatively wide. Notal edge discontinuous. Cerata in separate clusters on broad extensions. Rhinophores perfoliated or granulated. Anus pleuroproctic under reduced notal edge. No distinct oral glands. Radula formula 1.1.1. Rachidian teeth usually with cusp compressed by adjacent lateral denticles. Lateral teeth narrow or with attenuated process basally, denticulated or smooth. Single distal receptaculum seminis. Vas deferens long, with or without distinct prostate. External permanent penial collar absent. Penis elongated conical, internal unarmed.

##### Genera included.

*Baenopsis* gen. n., *Flabellinopsis* MacFarland, 1966.

##### Remarks.

One of the unexpected results of the present molecular analysis is the most basal position of the species *Flabellina iodinea* (Cooper, 1863) in relation to the families Paracoryphellidae, Coryphellidae, and Flabellinidae (Figs 1, 2). This is prima facie not very consistent with the morphological data since *F. iodinea* is similar to the members of the family Flabellinidae *s. str.* as it possesses a medium-sized body with cerata on lateral extensions. However, this may be a case of morphological convergence since *F. iodinea*'s relatively broad, flap-like lateral modifications of the notal edge and absence of distinct oral glands is different from the majority of the Flabellinidae *s. str.* Furthermore, the vas deferens in *F. iodinea* is considerably longer than in the taxa of the Flabellinidae *s. str.* On all trees obtained, *Flabellina iodinea* invariably appears most basally and separate from all other families. Therefore, in this case our results speak for preference of the molecular data in revealing the very separate position of this particular taxon. It is most likely that the *Flabellina*-like external appearance has evolved in *F. iodinea* independently from Flabellinidae *s. str.* by parallel modifications of a continuous ancestral notal edge into flap-like ceratal structures. We therefore resurrect the genus *Flabellinopsis* MacFarland, 1966, which was previously proposed for the species *Aeolis* (*Phidiana*?) *iodinea* Cooper, 1863. On the present molecular tree, the NE Pacific *Flabellinopsis iodinea* clustered together with a NE Atlantic species, “*Flabellina*” *baetica* Garcia-Gomez, 1984, in the same clade (Figs 1, 2, 7). The NE Atlantic species has ceratal and reproductive morphology somewhat similar to the genus *Flabellinopsis* but possesses very peculiar folded and granulated rhinophores and smooth lateral teeth (Garcia-Gomez 1984). Therefore, rather than uniting it into the genus *Flabellinopsis* making that morphologically not very consistent, we propose a separate new genus for *Flabellina baetica* (see below). The diversity of the family Flabellinopsidae fam. n. is more considerable than currently understood. On our tree, an undetermined species of “*Piseinotecus*” sp. appeared as possibly related to this family (Fig. 1). Furthermore, Furfaro et al. (2017) have shown that “*Piseinotecus*” *soussi* Tamsouri, Carmona, Moukrim and Cervera, 2014 (original description in Tamsouri et al. 2014) has appeared basally to “*Flabellina*” *baetica* and *Facelina quatrefagesi* (Vayssière, 1888). Further investigations need to be made on these taxa.

#### Baenopsis gen. n.

Taxon classificationAnimaliaNudibranchiaFlabellinopsidae

http://zoobank.org/7C0E306D-0BC4-4286-814B-983D01598489

Figs 2 , 7

##### Type species.

*Flabellina baetica* Garcia-Gomez, 1984

##### Etymology.

The genus name is derived from the type species name, “*F.*” *baetica*, originally named after one of the main historical provinces of Spain.

##### Diagnosis.

Body relatively wide. Notal ridge discontinuous. Cerata in separate clusters on broad lateral extensions. Rhinophores with ridges and granules, similar in size to oral tentacles. Anterior foot corners present. Anus pleuroproctic. Rachidian teeth with narrow compressed cusp and distinct denticles. Lateral teeth smooth with attenuated process basally. Single distal receptaculum seminis. Long vas deferens with distinct prostate. Penis conical (?).

##### Species included.

*Baenopsis baetica* (Garcia-Gomez, 1984), comb. n. (original description in Garcia-Gomez 1984).

#### Flabellinopsis

Taxon classificationAnimaliaNudibranchiaFlabellinopsidae

MacFarland, 1966, reinstated

Figs 2 , 7

##### Type species.

*Aeolis* (*Phidiana*?) *iodinea* Cooper, 1863.

##### Diagnosis.

Body relatively wide. Notal ridge discontinuous. Cerata in separate clusters on broad flaps. Rhinophores perfoliated, shorter than oral tentacles. Anterior foot corners present. Anus pleuroproctic. Rachidian teeth with narrow compressed cusp and distinct denticles. Lateral teeth denticulated with attenuated process basally. Single distal receptaculum seminis. Long vas deferens without distinct prostate. Penis bluntly conical.

##### Species included.

*Flabellinopsis iodinea* (Cooper, 1863) (original description in Cooper 1863; detailed redescriptions in MacFarland 1966 and Marcus and Marcus 1967).

#### Family Coryphellidae

Taxon classificationAnimaliaNudibranchiaCoryphellidae

Bergh, 1889, reinstated

##### Diagnosis.

Body wide to narrow. Notal edge reduced continuous, discontinuous, or fully reduced. Cerata not stalked, in continuous or discontinuous numerous rows. Rhinophores smooth, wrinkled, rarely annulated or perfoliated. Anus pleuroproctic under the reduced notal edge. Distinct oral glands commonly absent. Radula formula 1.1.1. Rachidian teeth usually with strong cusp, only rarely compressed by adjacent lateral denticles. Lateral teeth narrow or with attenuated process basally, always denticulated. Commonly both distal and proximal receptaculum seminis present. Vas deferens usually short, rarely long, with indistinct prostate. External permanent penial collar absent. Penis in many cases broad to disk-shaped, more rarely elongated conical, always internal, unarmed.

##### Genera included.

*Borealia* gen. n., *Coryphella* Gray, 1850, *Fjordia* gen. n., *Gulenia* gen. n., *Himatina* Thiele, 1931, *Itaxia* gen. n., *Microchlamylla* gen. n., *Occidentella* gen. n., *Orientella* gen. n.

##### Remarks.

The molecular analysis showed the presence of a well-supported (PP = 1, BS = 94) big clade (Figs 1, 2) which encompassed the majority of the traditional *Coryphella* (originally suggested by Gray 1850) including type species *C. verrucosa*, and also *C. lineata*, *C. nobilis* and *C. gracilis*. This clade is clearly distinct from the other traditional Flabellinidae as well as traditional basal Coryphellidae now placed in the separate family Paracoryphellidae (see above). However, even in the restricted sense *Coryphella* remains extremely heterogeneous by morphology of the notal ridge (continuous to discontinuous) and reproductive system anatomy (relatively long as in *C. gracilis* and *C. amabilis*, S-shaped in the type species *C. verrucosa*, or very short as in *C. lineata* or *C. browni*; *C. athadona* instead possesses an aberrant penial gland distal part of the prostate). The penis usually appears as a wide lobe in many species but may also be narrow and conical as in *C. gracilis* and *C. nobilis*). Thus, a further separation of more narrow genera within the reinstated family Coryphellidae is clearly necessary. The molecular analysis based on broad sampling of many Coryphellidae across both northern and southern hemispheres supports such a decision since it resulted in the formation of many well-supported smaller clades within the family Coryphellidae (Fig. 2). For example, the morphologically quite different *C. athadona* and *C. trilineata* stand apart and cluster more basally from the majority of the traditional *Coryphella*. Presence within the traditional genus *Coryphella* of wide bodied taxa like *C. nobilis*, *C. trophina* with continuous notal ridges, and the complete separateness (along with paraphyly) of the genus *Flabellina* implies that transformation of the continuous well-defined ancestral notal ridge into derived stalks or elevations has occurred several times independently within the family Flabellinidae. It is notable that wide bodied (new and resurrected) genera with a continuous notal edge, namely *Borealia*, *Itaxia* and *Himatina*, hold the most basal positions within various smaller clades of the family Coryphellidae (Fig. 2) suggesting that the common ancestor of all Coryphellidae was wide-bodied with a well-defined notal edge, as in the more basally placed members of the family Paracoryphellidae. The minimum uncorrected p-distances for the partial COI gene between the species of family Coryphellidae are given in Table 1. The minimum uncorrected p-distances between all genera range 10.1–18.3%. The lowest distances occurred between *Himatina trophina* and *Fjordia chriskaugei* (10.1%). Whereas the maximal uncorrected p-distances for the COI fragments between *Fjordia browni* and *Fjordia lineata* reach 6.4%, between *Fjordia lineata* and *Fjordia chriskaugei* sp. n. they reach 6.1%. Intra-genera variability reached significantly smaller values than variability between genera. The biggest distances occurred between *Itaxia falklandica* and other genera of family Coryphellidae (range 12.0–18.3%). Such considerable molecular divergences between taxa of the family Coryphellidae (Table 1) support the reliability of a multi-genera approach consistently applied in this study.

**Table 1.** Minimum uncorrected p-distances (%) between representatives of the genera of the family Coryphellidae.

	*Fjordia chriskaugei*	*Gulenia monicae*	*Himatina trophina*	*Coryphella verrucosa*	*Borealia nobilis*	*Occidentella athadona*	*Orientella trilineata*	*Microchlamylla gracilis*	*Itaxia falklandica*
***Fjordia chriskaugei***	-	11.9%	10.1%	12.5%	12.5%	11.7%	11.0%	13.2%	16.9%
***Gulenia monicae***	11.9%	-	12.8%	15.4%	12.0%	15.4%	15.7%	15.8%	17.5%
***Himatina trophina***	10.1%	12.8%	-	12.0%	11.1%	12.5%	11.4%	12.9%	12.0%
***Coryphella verrucosa***	12.5%	15.4%	12.0%	-	11.0%	13.1%	14.6%	14.2%	17.2%
***Borealia nobilis***	12.5%	12.0%	11.1%	11.0%	-	12.6%	12.0%	14.0%	17.4%
***Occidentella athadona***	11.7%	15.4%	12.5%	13.1%	12.6%	-	10.7%	14.1%	15.2%
***Orientella trilineata***	11.0%	15.7%	11.4%	14.6%	12.0%	10.7%	-	13.4%	18.3%
***Microchlamylla gracilis***	13.2%	15.8%	12.9%	14.2%	14.0%	14.1%	13.4%	-	17.2%
***Itaxia falklandica***	16.9%	17.5%	12.0%	14.2%	17.4%	15.2%	18.3%	17.2%	-

#### Borealia gen. n.

Taxon classificationAnimaliaNudibranchiaFlabellinopsidae

http://zoobank.org/1D19234E-39D5-441B-A608-5CCE63A9A93B

Figs 16 , 17

##### Type species.

*Coryphella nobilis* Verrill, 1880

##### Etymology.

After *boreo* (north in Latin) because of the amphiboreal distribution of the two species included.

##### Diagnosis.

Body wide. Notal ridge present, reduced, continuous. Cerata in continuous rows. Rhinophores wrinkled. Anterior foot corners present. Rachidian teeth with compressed narrow cusp and distinct denticles. Lateral teeth denticulated with attenuated process basally. Separated distal and proximal receptaculum seminis. Moderately long vas deferens expands to narrow penial sheath. Penis narrow, tubular.

##### Species.

**Figure 16.**
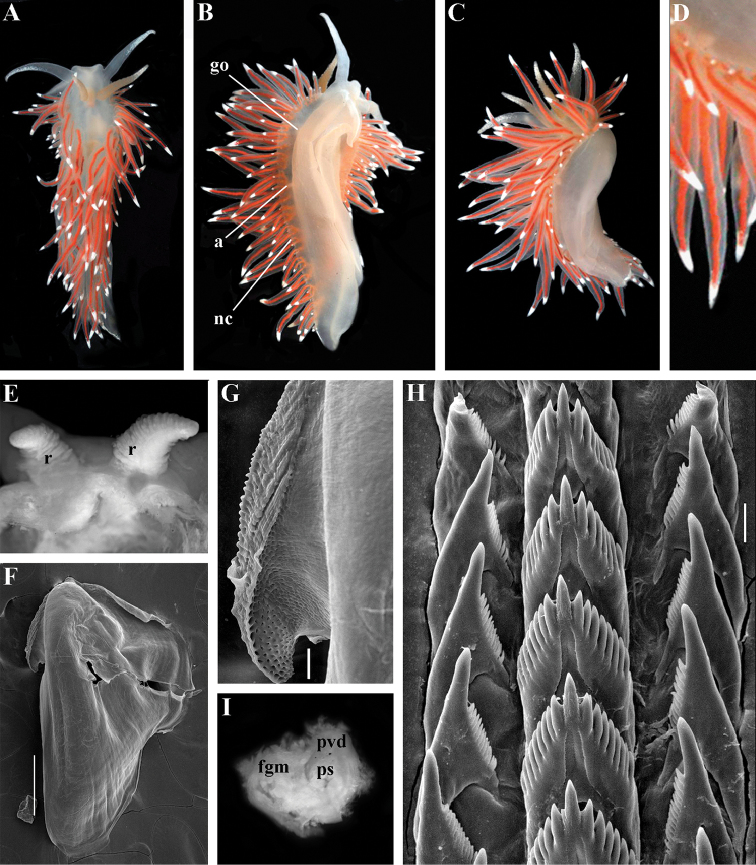
*Borealia nobilis* (Verrill, 1880), comb. n. ZMMU Op-510. White Sea, Cape Kartesh, 33 mm in length (live): **A** living animal, dorsal view **B** same, ventral view **C** same, lateral view **D** same, details of cerata **E** dissected anterior part (pharynx removed) and rhinophores, light microscopy **F** jaw, SEM **G** details of masticatory process of jaw, SEM **H** radular teeth, posterior part, SEM **I** reproductive system (non mature), light microscopy. Abbreviations: **a** anus **fgm** female gland mass **go** genital opening **nc** continuous notal edge **ps** penial sheath **pvd** prostatic vas deferens **r** rhinophores. Scale bars: **F** = 300 μm; **G, H** = 30 μm. Photos and SEM images by T.A. Korshunova, A.V. Martynov.

**Figure 17.**
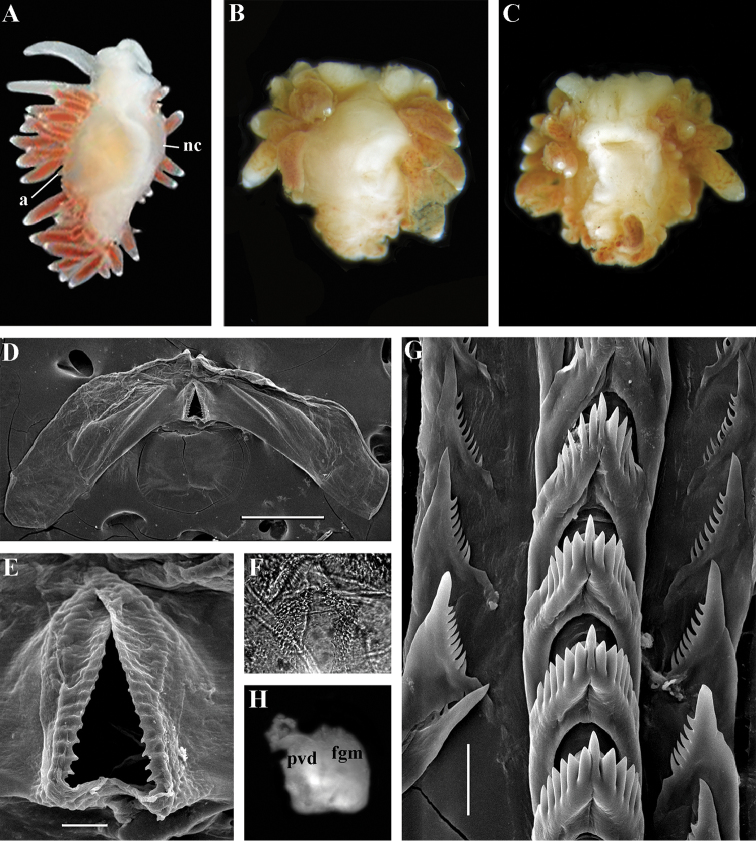
*Borealia sanamyanae* sp. n. ZMMU Op-518. Kurile Islands, Matua Island, living specimen 5 mm in length: **A** latero-ventral view **B** dorsal view (fixed) **C** ventral view (fixed) **D** jaw, SEM **E** details of masticatory process of jaw, SEM **F** details of masticatory process of jaw, light microscopy **G** radular teeth, posterior part **H** reproductive system (non-mature), light microscopy. Abbreviations: **a** anus **fgm** female gland mass **nc** continuous notal edge **pvd** prostatic vas deferens. Scale bars: **D** = 300 μm; **E, G** = 30 μm. Photos of living specimens by N.P. Sanamyan, other photos and SEM images by A.V. Martynov.

*Borealia nobilis* (Verrill, 1880), comb. n. (Fig. 16) (original description in Verril, 1880, detailed redescripton in Kuzirian 1977), *Borealia sanamyanae* sp. n. (Fig. 17).

##### Remarks.

The genus *Borealia* is clearly distinguished from any other Coryphellidae by a combination of continuous notal edge, long tubular penis, and compressed cusp of the rachidian radular teeth. In this study we discovered a closely related but clearly distinct (according to molecular data) new species of the genus *Borealia* from the North Pacific which forms separate sister clade to *B. nobilis* (Fig. 1).

#### Borealia sanamyanae sp. n.

Taxon classificationAnimaliaNudibranchiaCoryphellidae

http://zoobank.org/FB7C4260-5517-4449-9681-AB5168B0A200

Fig. 17

##### Type material.

Holotype, ZMMU Op-518, 5 mm long (fixed), Middle Kurile Islands, Matua Island, Cape Klyuv, 25.08.2016, depth 17 m, coll. N.P. Sanamyan.

##### Type locality.

North West Pacific, Middle Kurile Islands.

##### Etymology.

In honour of Nadezhda Sanamyan, marine biologist from Kamchatka. She has made a considerable contribution in collecting North West Pacific nudibranchs.

##### Diagnosis.

Continuous notal edge, background colour translucent white, digestive gland diverticula dark-red to pinkish, apical parts of cerata with white pigment, radula consists of more than 24 teeth, rachidian tooth with up to seven distinct denticles adpressed to central cusp, lateral teeth with few denticles on teeth edge.

##### Description.

*External morphology* (Fig. 17A–C). Body relatively wide. Foot and tail moderate, anterior foot corners relatively short. Rhinophores similar in size to oral tentacles, slightly wrinkled, robust. Dorsal cerata fusiform, long, continuously attached to rudimentary but uninterrupted notal edge without forming clusters. Apices of cerata pointed. Notum narrow but distinct throughout both lateral sides of body. Digestive gland diverticulum fills significant volume of cerata. Anal opening on right side below notal edge in first half of body but closer to middle. Reproductive openings lateral, below notal edge around middle part of body.

*Colour* (Fig. 17A). Background colour translucent white. Digestive gland diverticula dark-red to pinkish. Apical parts of cerata with opaque cap of white pigment.

*Jaws* (Fig. 17D–F). Masticatory process more than one-third as long as jaw body. Edge of masticatory processes bears ca. 20 denticles that continue to form several reduced rows of denticles on body of masticatory processes.

*Radula* (Fig. 17G). Radula formula: 24 × 1.1.1. Rachidian tooth elongate-triangular with small narrow cusp. Rachidian tooth bears 5-7 well-defined separated lateral denticles. Cusp is adpressed by adjacent first lateral denticles. Lateral teeth broadly triangular with obtuse and distinctly attenuated posteriorly outer process and between six and nine sharp long denticles on internal edge.

*Reproductive system* (Fig. 17H). The available material contains only a non-mature reproductive system, which do not allow recognition of characters in detail.

##### Ecology.

Shallow waters, stony and rocky habitats.

##### Distribution.

Northwest Pacific.

##### Remarks.

According to the molecular phylogenetic analysis *Borealia sanamyanae* sp. n. forms a separate sister clade to *B. nobilis* (Fig. 1). The following morphological characters distinguish *B. sanamyanae* sp. n. from *B. nobilis*: larger number of radular rows (max up to 22 in 40–50 mm length *B. nobilis* (Kuzirian 1979); our specimens from the White and Barents Seas are about 20–30 mm in length and have max 20 rows, whereas a ten times smaller 5-mm specimen of *B. sanamyanae* sp. n. has 24 rows), and the cusp of the central tooth in *B. nobilis*, though relatively low, is almost not adpressed by the adjacent lateral denticles (Fig. 16H), whereas in *B. sanamyanae* sp. n. it is considerably adpressed (Fig. 17G).

#### Coryphella

Taxon classificationAnimaliaNudibranchiaCoryphellidae

Gray, 1850, restricted

**Figure 18.**
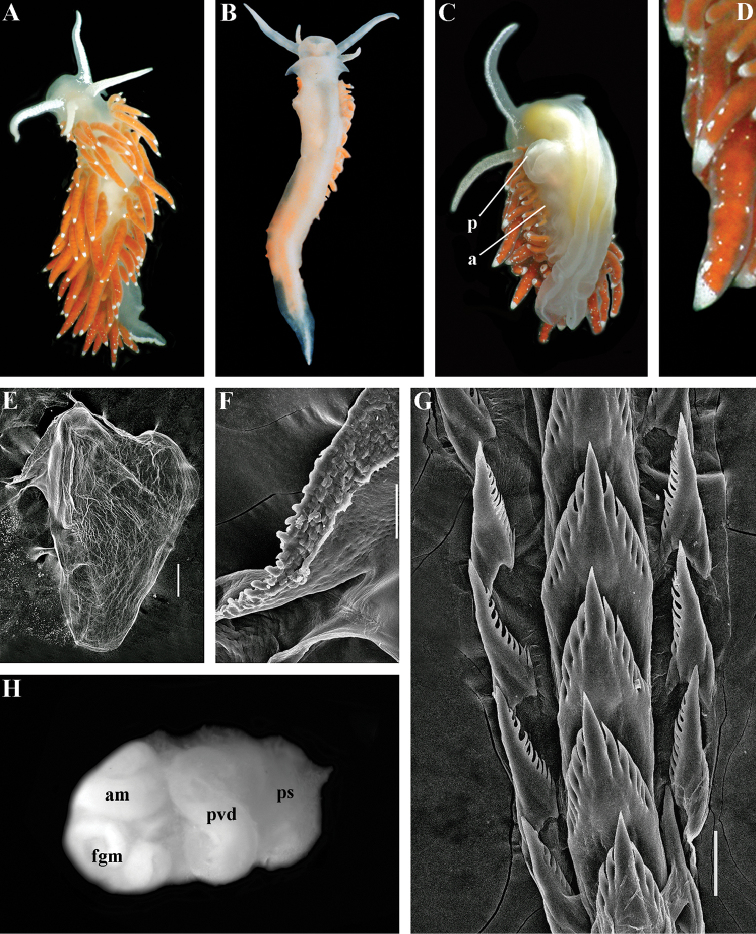
*Coryphella pseudoverrucosa* Martynov et al., 2015. ZMMU Op- 528. NW Pacific, Kamchatka, Avachinskaya guba, living specimen 12.5 mm in length (fixed): **A** dorsal view **B** ventral view **C** lateral view **D** details of cerata **E** jaw, SEM **F** details of masticatory process of jaw, SEM **G** radular teeth, posterior part, SEM **H** reproductive system, light microscopy. Abbreviations: **a** anus **am** ampulla **fgm** female gland mass **p** penis **ps** penial sheath **pvd** prostatic vas deferens. Scale bars: **E** = 300 μm; **F** 30 μm; **G** = 100 μm. Photos and SEM images by T.A. Korshunova, A.V. Martynov.

**Figure 19.**
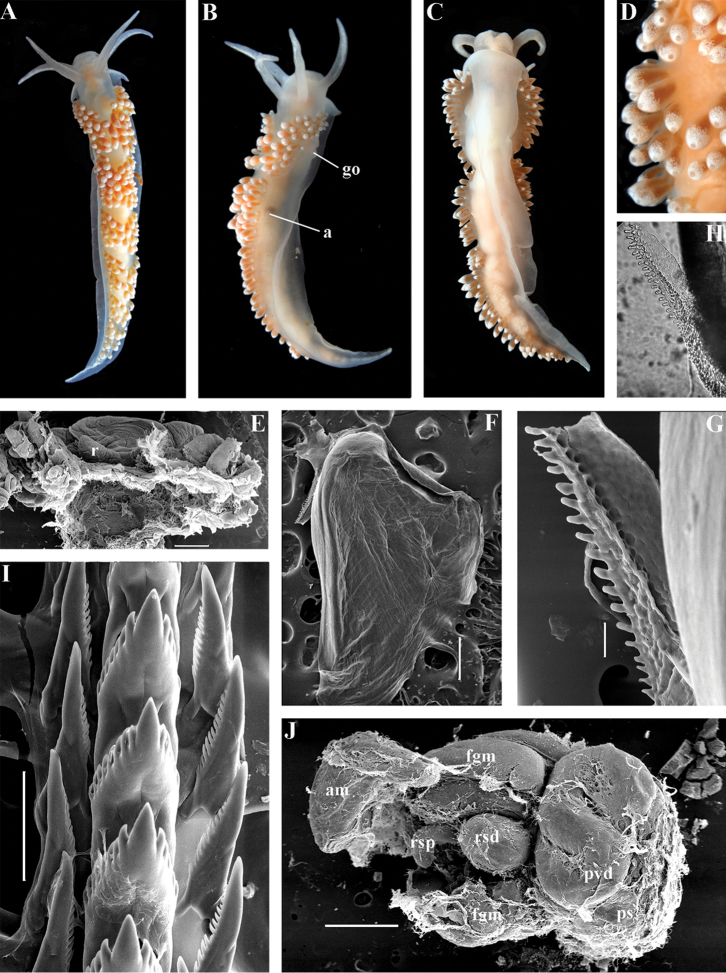
*Coryphella verrucosa* (M. Sars, 1829), short cerata (typical) morphotype. ZMMU Op-539. Norwegian Sea, Gulen Dive Center, living specimen 38 mm in length: **A** dorsal view **B** lateral view **C** right ventral view **D** details of cerata **E** dissected anterior part (pharynx removed) and rhinophores, SEM **F** jaw, SEM **G** details of masticatory process of jaw, SEM **H** details of masticatory process of jaw, light microscopy **I** radular teeth, posterior part, SEM **J** reproductive system, SEM. Abbreviations: **a** anus **am** ampulla **rsd** distal receptaculum seminis **fgm** female gland mass **go** genital opening **ps** penial sheath **pvd** prostatic vas deferens **r** rhinophores **rsp** proximal receptaculum seminis. Scale bars: **E, J** = 1 mm; **F** = 300 μm; **G** = 30 μm **I** = 100 μm. Photos and SEM images by T.A. Korshunova, A.V. Martynov.

**Figure 20.**
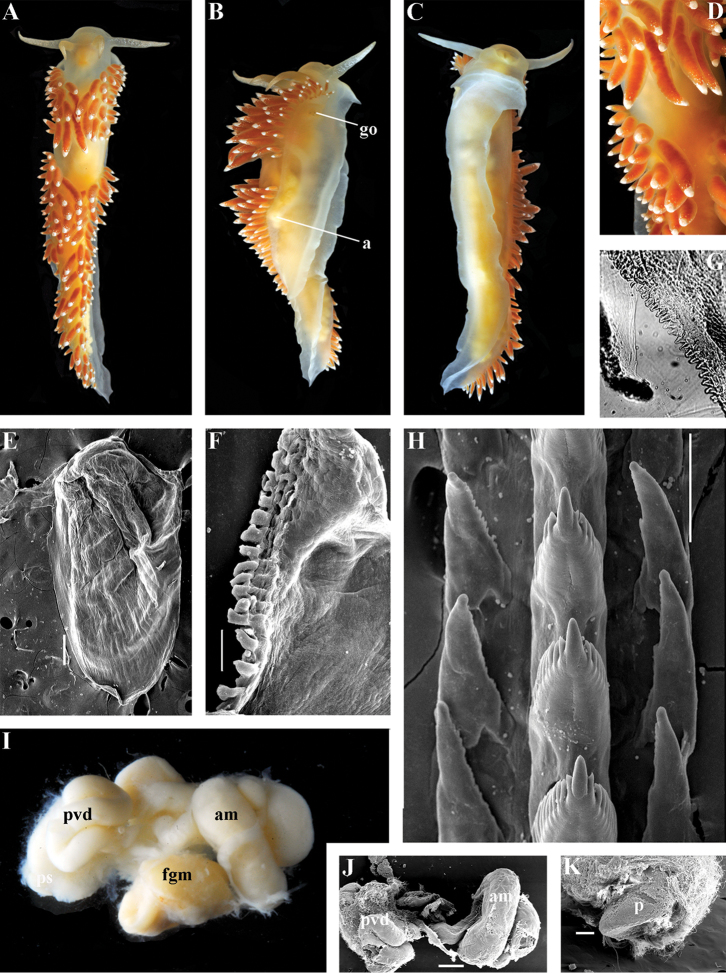
*Coryphella verrucosa* (M. Sars, 1829), long cerata (“rufibranchialis”) morphotype. ZMMU Op-521, Barents Sea, Oskara Bay, living specimen 42 mm in length (live): **A** dorsal view **B** lateral view **C** ventral view **D** details of cerata **E** jaw, SEM **F** details of masticatory process of jaw, SEM **G** details of masticatory process of jaw, light microscopy **H** radular teeth, posterior part, SEM **I** reproductive system, light microscopy **J** reproductive system, SEM **K** penis partially everted, SEM. Abbreviations: **a** anus **am** ampulla **fgm** female gland mass **go** genital opening **p** penis **pvd** prostatic vas deferens. Scale bars: **E** bar = 300 μm; **F, K** = 30 μm; **H** = 100 μm; **J** = 1 mm. Photos and SEM images by T.A. Korshunova, A.V. Martynov.

**Figure 21.**
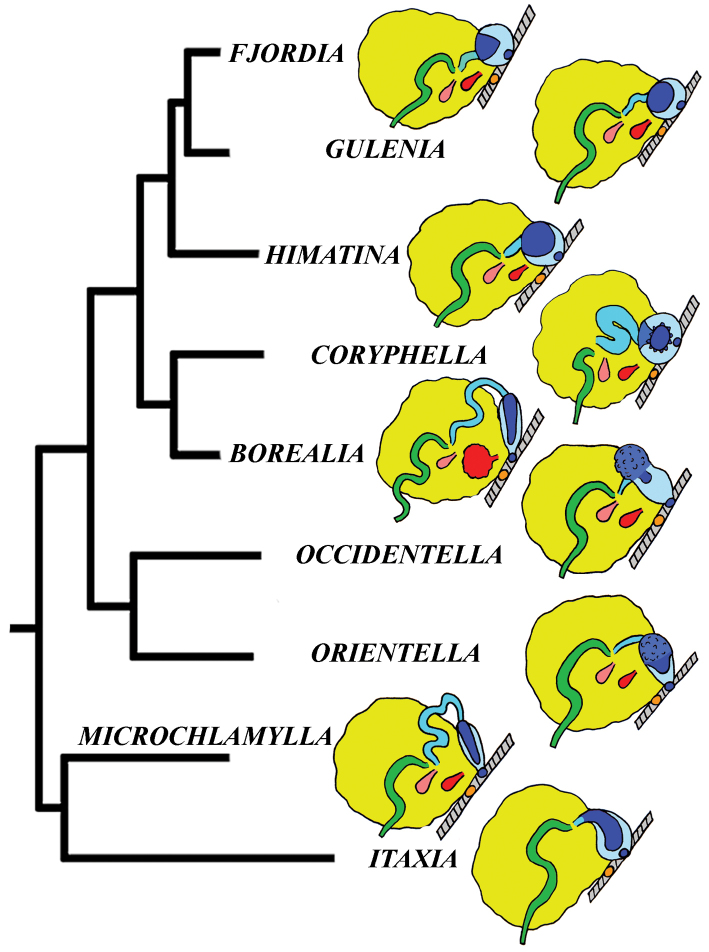
Schematic outline of the reproductive systems of the taxa of the families Coryphellidae integrated with molecular phylogenetic data. Colour indication of reproductive system characters: ampulla – green; body wall – gray; distal receptaculum seminis – red; female gland mass – yellow; female genital opening – orange; penis and male genital opening – dark blue; penial sheath – pale blue; prostatic vas deferens – turquoise; proximal distal receptaculum seminis – pink.

Figs 2 , 18 , 19 , 20 , 21

##### Type species.

*Eolidia verrucosa* M. Sars, 1829

##### Diagnosis.

Body narrow. Notal ridge completely reduced. Cerata in several groups. Rhinophores smooth with small tubercles. Anterior foot corners present. Rachidian teeth with non-compressed cusp and distinct denticles. Lateral teeth denticulated without attenuated process basally. Separated distal and proximal receptaculum seminis. S-shaped thick prostatic vas deferens. Penis disk-shaped with numerous small triangular processes at the disk edge.

##### Species included.

*Coryphella pseudoverrucosa* Martynov, Sanamyan, Korshunova, 2015 (Fig. 18) (original description in Martynov et al. 2015), *Coryphella verrucosa* (M. Sars, 1829) (Figs 19, 20) (original description in M. Sars 1829).

##### Remarks.

In the restricted sense the genus *Coryphella* Gray, 1850 represents a well-defined unit of narrow-bodied coryphellids with completely reduced notal edge and characteristic thick S-shaped prostatic vas deferens (Figs 18H, 19J, 20I, 21). Two morphologically similar species, the North Atlantic type species of the genus *C. verrucosa* and the North Pacific *C. pseudoverrucosa*, are clearly distinguished both morphologically (particularly the shape of the rachidian teeth of the radula) and according to our molecular analysis (Fig. 1).

#### Fjordia gen. n.

Taxon classificationAnimaliaNudibranchiaCoryphellidae

http://zoobank.org/79C027AE-BB13-4C3D-B88F-31504263704D

**Figure 22.**
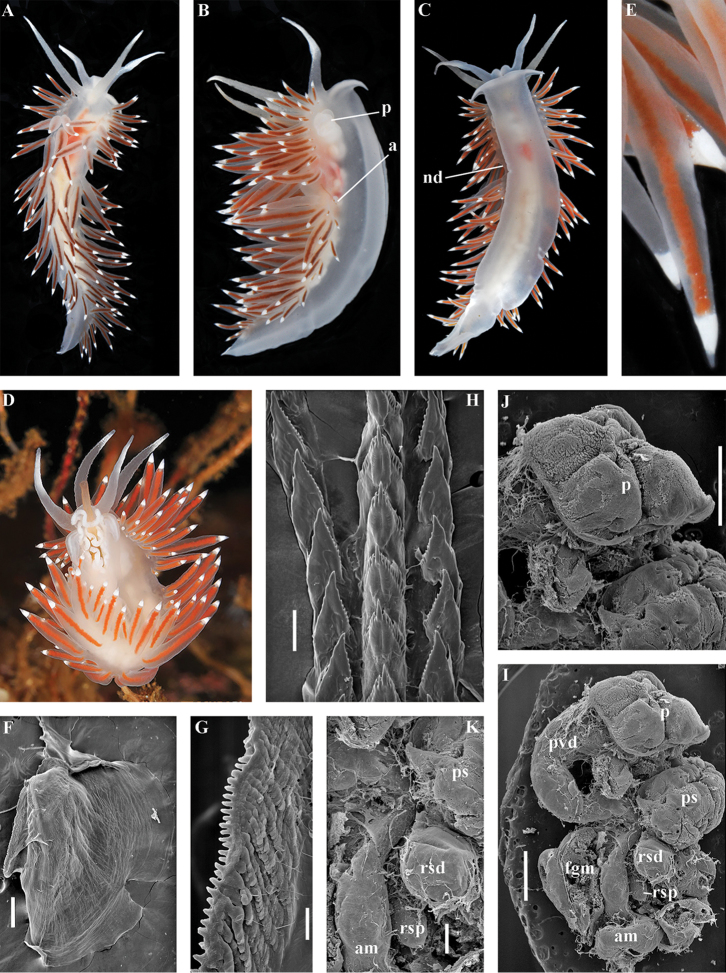
*Fjordia browni* (Picton, 1980), comb. n. Norwegian Sea, Gulen Dive Center. ZMMU Op-413, living specimen 48 mm in length: **A** dorsal view **B** lateral view **C** ventral view **D** living specimen on hydroid colony **E** details of cerata **F** jaw, SEM **G** details of masticatory process of jaw, SEM **H** radular teeth, posterior part, SEM **I** reproductive system, SEM **J** penis, SEM **K** seminal reservoirs. Abbreviations: **a** anus **am** ampulla **rsd** distal receptaculum seminis **fgm** female gland mass **nd** discontinuous notal edge **p** penis **ps** penial sheath **pvd** prostatic vas deferens **rsp** proximal receptaculum seminis. Scale bars: **F, J, K** = 300 μm; **G** = 30 μm; **H** = 100 μm; **I** = 1 mm. Photos of living specimens by T.A. Korshunova (**A–C**), and C. Skauge (**D**), SEM images by A.V. Martynov.

**Figure 23.**
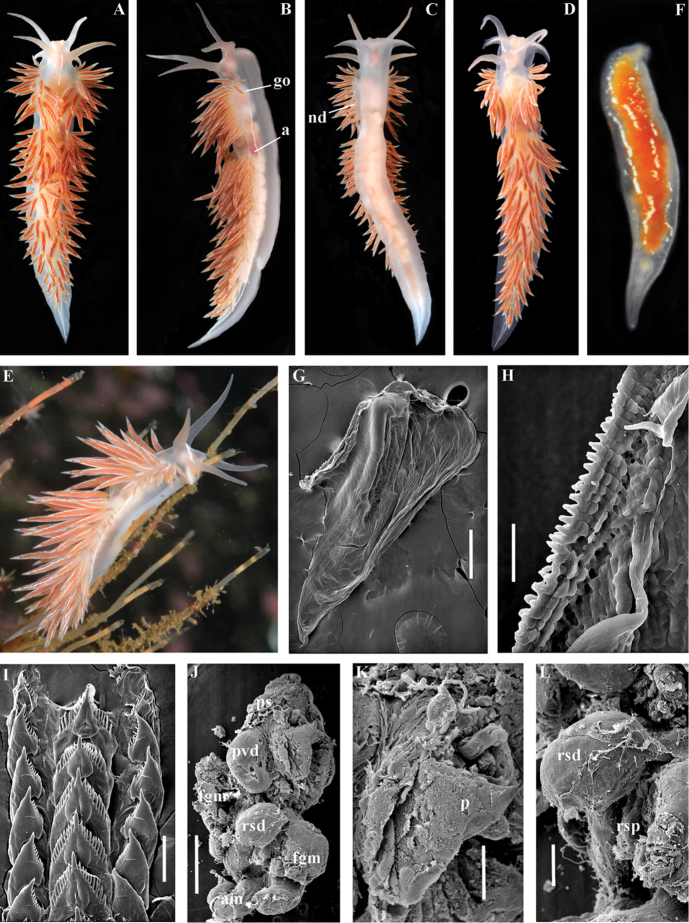
*Fjordia chriskaugei* sp. n. **A–C, F** Holotype ZMMU Op-477, living specimen 45 mm length, Norwegian Sea, Gulen Dive Center: **A** dorsal view **B** lateral view **C** ventral view **F** same, details of cerata **D** Paratype ZMMU Op- 412, living specimen 42 mm length, dorsal view, Gulen **E** living specimen on a hydroid colony, ZMMU Op-499, Ireland **G–L** Paratype ZMMU Op-405, details of living specimen 30 mm length, Gulen **G** jaw, SEM **H** details of masticatory process of jaw **I** radular teeth, posterior part, SEM **J** reproductive system, SEM **K** penis, SEM **L** seminal reservoirs, SEM. Abbreviations: **a** anus **am** ampulla **rsd** distal receptaculum seminis **fgm** female gland mass **go** genital opening **nd** discontinuous notal edge **p** penis **ps** penial sheath **pvd** prostatic vas deferens **rsp** proximal receptaculum seminis. Scale bars: **G, K, L** = 300 μm; **H** = 30 μm; **I** = 100 μm; **J** = 1 mm. Photos of living specimens by T.A. Korshunova (**A–D**), and B. Picton (**E**), SEM images by A.V. Martynov.

**Figure 24.**
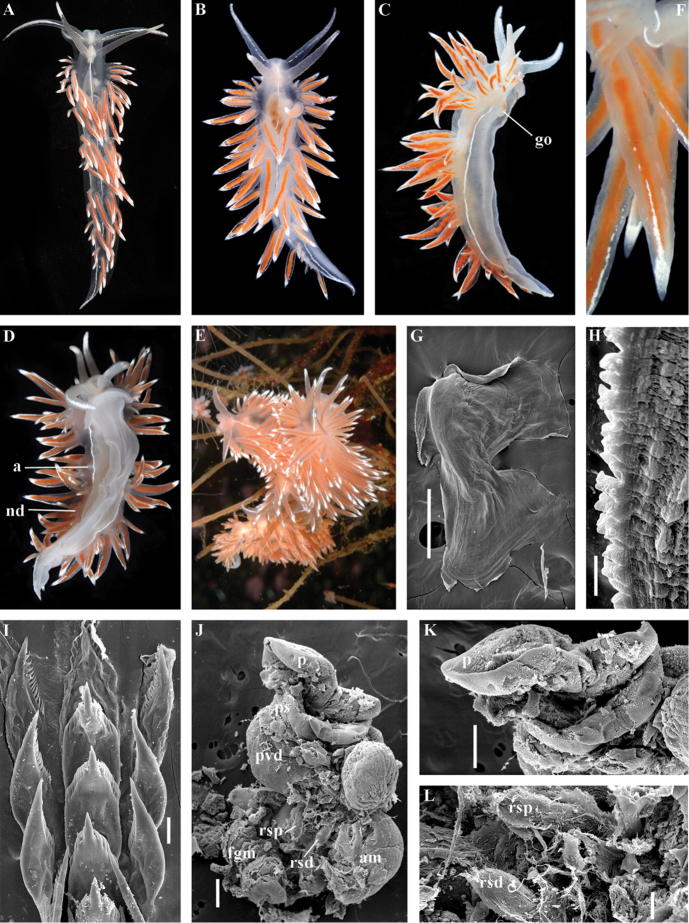
*Fjordia lineata* (Lovén, 1846), comb. n. **A** neotype 11 mm length (fixed), dorsal view (live), NTNU-VM-72483, Idefjorden, Sverige **B** living specimen 21 mm length dorsal view (ZMMU Op-507) **F** same, details of cerata **C** ZMMU Op-508, lateral view **D** same, ventral view **E** living specimens (not collected) on a hydroid colony **G** jaw, SEM (ZMMU Op-507) **H** details of masticatory process of jaw, SEM (ZMMU Op-507) **I** radular teeth, posterior part, SEM (ZMMU Op-508) **J** reproductive system, SEM, scale bar = 1 mm (ZMMU Op-507) **K** penis, SEM (ZMMU Op-507) **L** seminal reservoirs (ZMMU Op-507). Abbreviations: **a** anus **am** ampulla **rsd** distal receptaculum seminis **fgm** female gland mass **go** genital opening **p** penis **ps** penial sheath **pvd** prostatic vas deferens **rsp** proximal receptaculum seminis. Scale bars: **G, I, K** = 300 μm **H** = 10 μm; **J** = 1 mm; **L** = 100 μm. Photos of living specimens by T.A. Korshunova (**A, C, D**), and B. Picton (**B, E**), SEM images by A.V. Martynov.

Figs 21 , 22 , 23 , 24

##### Type species.

*Aeolis lineata* Lovén, 1846.

##### Etymology.

After the Norwegian word “fjord” because of the type locality of the Oslofjord and also this is a very common species at Gulen at the mouth of the Sognefjord, where many of the studied materials come from.

##### Diagnosis.

Body narrow. Notal edge present, moderately reduced, discontinuous. Cerata in several groups. Rhinophores smooth, similar in length or shorter than oral tentacles. Anterior foot corners present. Rachidian teeth with non-compressed cusp and distinct denticles. Lateral teeth denticulated with attenuated process basally. Separated distal and proximal receptaculum seminis. Vas deferens very short, expanding to a broad penial sheath. Penis broad, lobe-shaped.

##### Species included.

*Fjordia browni* (Picton, 1980), comb. n. (original description in Picton 1980) (Fig. 22), *Fjordia* (?) *capensis* (Thiele, 1925), comb. n. (original description in Thiele 1925), *Fjordia lineata* (Lovén, 1846), comb. n. (original description in Lovén 1846), *Fjordia chriskaugei* sp. n., *Fjordia* (?) *insolita* (Garcia-Gomez & Cervera, 1989), comb. n. (original description in Garcia-Gomez and Cervera 1989).

##### Remarks.

**Figure 25.**
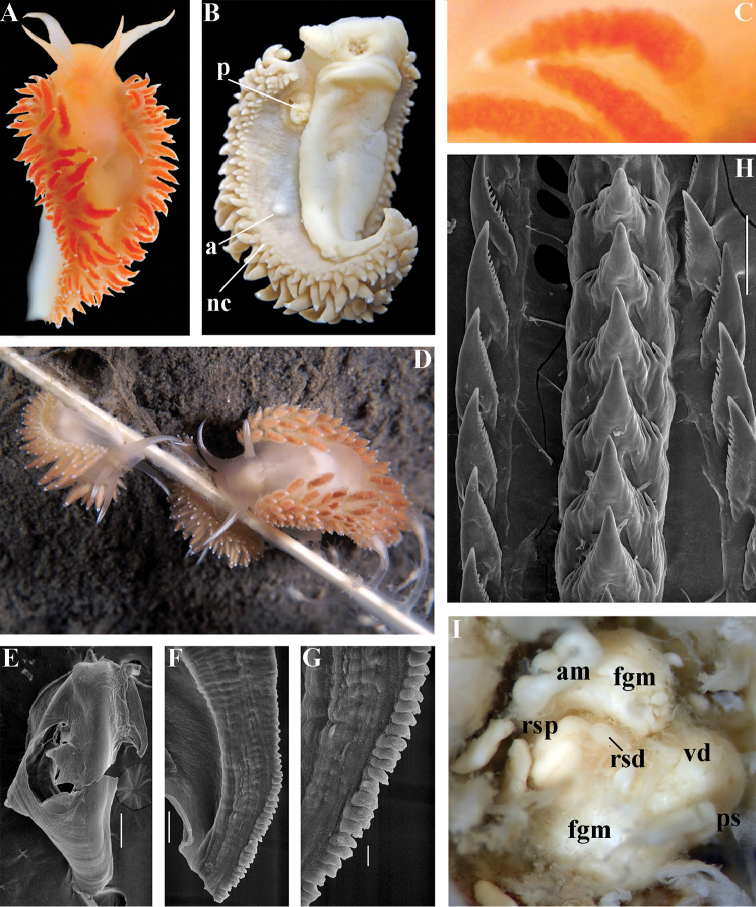
*Gulenia borealis* (Odhner, 1922), comb. n. GNM Gastropoda 9417, 11 mm length (fixed), Norway (**A–C, E–I**), Sweden (**D**): **A** dorsal view (live) **B** ventro-lateral view (fixed) **C** details of cerata **D** living animals in situ on *Funiculia* (Pennatulacea) **E** jaw, SEM **F**, **G** details of masticatory process of jaw, SEM **H** radular teeth, posterior part **I** reproductive system. Abbreviations: **a** anus **am** ampulla **fgm** female gland mass **nc** continuous notal edge **p** penis **ps** penial sheath **rsd** distal receptaculum seminis **rsp** proximal receptaculum seminis **vd** vas deferens (muscular part). Scale bars: **E** = 300 μm; **F** = 30 μm; **G** = 10 μm; **H** = 100 μm. Photos of living specimens by T. Cedhagen (**A**), and K. Malmberg (**D**), SEM images by A.V. Martynov.

According to combined morphological and molecular evidence, the traditional species *Coryphella lineata* was found to be a very heterogeneous species complex. This complex includes at least five species, which are clearly separated into two major clades according to molecular analysis (Figs 1, 2) and by the morphological data (presence or absence of continuous notal edge). According to these data two new genera are proposed for the former *C. lineata* complex. First is the group of narrow-bodied coryphellids with discontinuous notal edge which included the proper *C. lineata*, *C. browni*, and a new species (see below); it is named as the new genus *Fjordia* gen. n. The second group with a continuous notal edge includes two new species and the previously enigmatic species *Coryphella borealis* Odhner, 1922 (not to be confused with *Chlamylla borealis* Bergh, 1886, see above), which represents specimens wrongly identified as *Coryphella gracilis* in a barcoding project by Barco et al. (2016) and which have been fully investigated for the first time in the present studies (Fig. 25) including both morphological and molecular study (Figs 1, 2). Our sequences of real *C. borealis* are identical to the above-mentioned wrongly identified *C. gracilis* from GenBank. Real *C. gracilis* has very different morphological features and this species is recovered in a completely different clade in our molecular tree (Figs 1, 2). These two clades, clearly delineated by morphology and molecular analysis are therefore named *Fjordia* gen. n. (with discontinuous notal edge) and *Gulenia* gen. n. (with continuous notal edge).

In this study it is also confirmed for the first time that the enigmatic *C. borealis* Odhner, 1922 belongs to the genus *Gulenia* according to integrative morphological and molecular studies (Figs 1, 2, 25). Both new genera share a similar reproductive system with a very short vas deferens and wide penis (Fig. 21), that imply a common ancestry, confirmed by molecular data (Figs 1, 2). *Fjordia browni* (Fig. 22) unlike other species of the genus *Fjordia* does not possess both dorsal and lateral white lines, but the presence of rudimentary notal edges, shape of the radular teeth, short vas deferens, and broad penial lobe are consistent with *F. lineata*. Molecular results place this species as sister to *Fjordia chriskaugei* sp. n. and in a clade where other members have dorsal and lateral white lines. All studied specimens from the Gulen region demonstrate a high degree of uniformity both in body shape and colour. Originally described from the UK where specimens were significantly smaller than exemplars from Gulen, nevertheless it keeps all essential diagnostic characters; discontinuous notal ridge, complete absence of the white lines, and radular pattern. The specimens from Gulen measured up to 100 mm in length. According to the morphological data, we included two more species (the South African *Coryphella capensis* and western Mediterranean *Flabellina insolita*) in the genus *Fjordia*, but further molecular data are necessary to confirm this.

#### Fjordia chriskaugei sp. n.

Taxon classificationAnimaliaNudibranchiaCoryphellidae

http://zoobank.org/EA48D174-03B1-4B8C-A6BB-4833CAB0FABE

Fig. 23

Coryphella lineata (auctt.)
Flabellina lineata *sensu*, part.: 88.
Non Aeolis lineataLovén 1846: 8.

##### Type material.

Holotype, ZMMU Op-477, 45 mm long (live), Norway, entrance of the Sognefjord, Gulen Dive Resort, 19.03.2015, depth 20 m, coll. T.A. Korshunova, A.V. Martynov. 1 paratype, ZMMU Op-402, 28 mm long (live), Norway, entrance of the Sognefjord, Gulen Dive Resort, 18.03.2015, depth 10–20 m, coll. T.A. Korshunova, A.V. Martynov. 1 paratype, ZMMU Op-404, 28 mm long (live), Norway, entrance of the Sognefjord, Gulen Dive Resort, 16.03.2015, depth 20 m, coll. T.A. Korshunova, A.V. Martynov. 1 paratype, ZMMU Op-405, 30 mm long (live), Norway, entrance of the Sognefjord, Gulen Dive Resort, 16.03.2015, depth 20 m, coll. T.A. Korshunova, A.V. Martynov. 1 paratype, ZMMU Op-412, 42 mm long (live), Norway, entrance of the Sognefjord, Gulen Dive Resort, 17.03.2015, depth 10–20 m, coll. T.A. Korshunova, A.V. Martynov. 1 paratype, ZMMU Op-499, Achill Island, Co. Mayo, Ireland, 06.04.2015, coll. B.E. Picton.

##### Type locality.

Gulen Dive Resort, Norway.

##### Etymology.

In honour of Christian Skauge (Gulen Dive Resort and Scubapixel), the organiser of the “Nudibranch Safari” and our great friend, who first noticed the heterogeneity of the traditional *C. lineata* in the field in Norway, including photographic records.

##### Diagnosis.

Discontinuous notal edge, background colour translucent white, digestive gland diverticula pink, orange-brown to reddish-brown, apical parts of cerata with white pigment, usually one to several punctuated fine white lines run along dorsal face of cerata, thin opaque white lines on dorsal and lateral sides, rachidian tooth with up to 10 distinct denticles delineate from central cusp, lateral teeth with up to eleven denticles on teeth edge, penis is a broad lobe.

##### Description.

*External morphology* (Fig. 23A–E). Body relatively narrow. Foot and tail moderate, anterior foot corners long. Oral tentacles long. Rhinophores ca. 1.5 times longer than oral tentacles, slightly wrinkled. Dorsal cerata finger-shaped to fusiform, forming several clusters along dorsal edges. Apices of cerata gradually pointed, with elongate cnidosac. Distinct notal edge remains mostly below cerata clusters. Digestive gland diverticulum fills significant volume of cerata. Anal opening on right side below second large cluster of cerata. Reproductive openings lateral, below first anterior cluster of cerata.

*Colour* (Fig. 23A, D). Background colour translucent white. Digestive gland diverticula pale pink, orange-brown to reddish-brown depending on locality and diet. Pinkish oral tube, oesophagus, hindgut, and gonads shine through dorsal sides. Thin opaque zigzag white line runs along middle of whole dorsum from head to tail. Similar single thin median line runs on both lateral sides of the body. Rhinophores background colour similar to body; along dorsal side of rhinophores runs a thin white line; apical white pigment absent. Dorsal sides of oral tentacles covered with thin opaque white line. One to several punctuated fine white lines run along dorsal face of cerata, sometimes absent. Apical parts of cerata without any opaque cap of white pigment.

*Jaws* (Fig. 23G, H). Masticatory process more than one-third as long as jaw body. Edge of masticatory processes bears ca. 60 denticles that continue to form several reduced rows of denticles on body of masticatory processes.

*Radula* (Fig. 23I). Radula formula: 13 × 1.1.1. Rachidian tooth elongate-triangular with short narrow cusp of less than 1/3 of tooth length (Fig. 23I). Rachidian tooth bears between seven and ten well-defined separated long lateral denticles not adpressed towards the cusp. Cusp delineated from adjacent first lateral denticles. Lateral teeth (Fig. 23I) broadly triangular with obtuse and considerably attenuated posteriorly outer process and between eight and eleven sharp long denticles on internal edge.

*Reproductive system* (Fig. 23J–L). Diaulic. Hermaphroditic duct leads to convoluted ampulla of about two whorls. Vas deferens very short, attached to dorsal side of penial sheath, no distinct prostate. Penial sheath large, wide. Penis is broad lobe (Fig. 23L). Oviduct connects through insemination duct into female gland complex. Vagina short and indistinct. Proximal receptaculum seminis large, oval, swollen. Distal receptaculum seminis present.

##### Ecology.

Associates with *Tubularia* colonies usually at depth 20–40 m. Attacks and feeds on polyps of *Tubularia indivisa* L., 1758. Juveniles probably start to feed on *Eudendrium* spp. or other smaller athecate hydroids. This species is abundant in some localities. Egg mass narrow cord; forms irregular, compressed pink or off-white spirals. Reproduction period from February to June; larva planktotrophic veliger with oval shell.

##### Distribution.

Northeast Atlantic, including Ireland, Great Britain (Scotland, England, Wales), Norway.

##### Remarks.

*Fjordia chriskaugei* sp. n. is distinguished morphologically from *F. lineata* (Lovén, 1846) *s. str.* (Fig. 24) by faint dotted lines on the dorsal surface of the cerata, the complete absence of the apical white cap, by rhinophores longer than oral tentacles, and by the distinct cusp of the rachidian teeth which is placed above the adjacent lateral denticles. Molecular analysis corroborates the morphological data (see Discussion) showing *F. chriskaugei* sp. n. as a species distinct from *F. lineata* *s. str.* (Fig. 1). Thompson and Brown (1984, pl. 27) and Picton and Morrow (1994, p. 95) illustrate this species as *Coryphella lineata* and their descriptions are a composite of the two species. The majority of specimens demonstrate significant uniformity in the body shape and colour. In some specimens from Ireland the irregular white lines on the cerata can be completely absent.

#### Fjordia lineata

Taxon classificationAnimaliaNudibranchiaCoryphellidae

(Lovén, 1846) comb. n.

Fig. 24

Aeolis lineata Lovén, 1846: 8.
Coryphella lineata : Thompson and Brown 1984 (pars.)
Flabellina lineata *sensu*Malmberg and Lundin 2015 (pars.): 88

##### Type material.

Original type material lost. Neotype, NTNU-VM-72483, 11 mm long (fixed) is designated here from the Svarte Jan, Idefjorden, Sweden, 06.06.2015, depth 22 m, coll. Mats Larsson, close to Lovén’s original type locality.

##### Material.

One specimen, ZMMU Op-403, 19 mm long (live), Norway, entrance of the Sognefjord, Gulen Dive Resort, 18.03.2015, depth 10–20 m, coll. Jørn Ari, Tina Malmgren. One specimen, ZMMU Op-406, 27 mm long (live), Norway, entrance of the Sognefjord, Gulen Dive Resort, 17.03.2015, depth 10–20 m, coll. T.A. Korshunova, A.V. Martynov. One specimen, ZMMU Op-506, 34 mm long (live), Norway, entrance of the Sognefjord, Gulen Dive Resort, 17.03.2015, depth 20 m, coll. T.A. Korshunova, A.V. Martynov. One specimen, ZMMU Op-506, 37 mm long (live), Norway, entrance of the Sognefjord, Gulen Dive Resort, 17.03.2015, 20 m depth, coll. T.A. Korshunova, A.V. Martynov. One specimen, ZMMU Op-507, 21 mm long (live), Norway, entrance of the Sognefjord, Gulen Dive Resort, 01.03.2014, depth 20 m depth, coll. T.A. Korshunova, A.V. Martynov. One specimen, ZMMU Op-508, 21 mm long (live), Norway, entrance of the Sognefjord, Gulen Dive Resort, 17.03.2015, 20 m depth, coll. T.A. Korshunova, A.V. Martynov.

##### Type locality.

Oslofjord, Norway.

##### Diagnosis.

Discontinuous notal edge, background colour translucent white, digestive gland diverticula pink, orange-brown to reddish-brown, apical parts of cerata with white pigment, along dorsal face of cerata runs thin straight or slightly curved line, thin opaque white lines on dorsal and lateral sides, rachidian tooth with up to seven distinct denticles delineate from central cusp, lateral teeth with up to 14 denticles on teeth edge, penis is a broad lobe.

##### Description.

*External morphology* (Fig. 24A–E). Body elongate, graceful, laterally compressed. Foot narrow, tail moderate, anterior foot corners long. Oral tentacles long. Rhinophores long and slightly wrinkled, similar in length to oral tentacles. Dorsal cerata finger-shaped forming two to four indistinct clusters. Apices of cerata pointed. Distinct notum remains only below cerata clusters. Digestive gland diverticulum fills significant volume of the cerata. Anal opening on right side of body between first and second pair of dorsolateral processes. Reproductive openings lateral, below first pair of dorsolateral cerata on right side.

*Colour* (Fig. 24A). Background colour translucent white. Digestive gland diverticula orange-brown to salmon to bright red to dark brown, almost black when feeding on *Bougainvillea*. Thin continuous opaque white line runs over middle of dorsum from head to tail. Similar single thin median line runs on both lateral sides of the body. Rhinophores similar in colour to body; apical parts covered with faint opaque white pigment. Dorsal sides of oral tentacles covered with thin opaque white line. Along dorsal face of cerata runs thin straight or slightly curved line. Apical parts of cerata covered with opaque oblique ring of white pigment which connects to median cerata line. Cnidosac obscured by white pigment ring. Scattered small white dots over cerata absent.

*Jaws* (Fig. 24G, H). Masticatory process more than one-third as long as jaw body. Edge of masticatory processes bears ca. 70 denticles that continue to form several reduced rows of denticles on body of masticatory processes.

*Radula* (Fig. 24I). Radula formula: 17 × 1.1.1. Rachidian tooth horseshoe-shaped without evident cusp (Fig. 24I). Rachidian tooth bears six or seven well-defined separated long lateral denticles. Cusp indistinct and sunken below adjacent first lateral denticles. Lateral teeth (Fig. 24I) narrowly triangular with sharpened and attenuated outer process and between eleven and fourteen sharp long denticles on internal edge.

*Reproductive system* (Fig. 24J–L). Diaulic. Hermaphroditic duct leads to convoluted ampulla of about two whorls. Vas deferens very short, no distinct prostate. Penial sheath large, wide. Penis is broad lobe (Fig. 24K). Oviduct connects through insemination duct into female gland complex. Vagina short and indistinct. Proximal receptaculum seminis large, oval, swollen. Distal receptaculum seminis present.

##### Ecology.

Associates with the *Tubularia indivisa* and *Ectopleura larynx* colonies usually at depths of 10–40 m. Attacks and feeds on polyps of *Tubularia, Ectopleura, Eudendrium*, *Bougainvillea* and other athecate hydroids. Juveniles probably start to feed on species with smaller polyps. This species is abundant in some localities. Egg mass is irregular compressed spiral cord. Reproduction period from February to June; the larva is a planktotrophic veliger with an oval shell.

##### Distribution.

Confirmed specimens of *F. lineata* Northeast Atlantic, including Ireland, Great Britain, Norway (Oslofjord to Lofoten Islands) (Odhner 1939; present study), Sweden (Bohuslän region) (Malmberg and Lundin 2015) western Mediterranean (Thompson and Brown 1984, Trainito and Doneddu 2014).

##### Remarks.

*Coryphella lineata* in the traditional sense (e.g., Odhner 1939; Thompson and Brown 1984; Evertsen and Bakken 2005) is a genera complex (see below) according to both morphological and molecular data (Fig. 1). Lovén’s (1846) original description of *Aeolis lineata* is short and uninformative. In order to maintain the current usage of the specific name *Fjordia lineata* (Lovén, 1846) a neotype is designated here (Fig. 24). The neotype was collected in shallow waters in the Idefjord on the south-eastern coast of Sweden, in the vicinity of the original type locality in the Bohuslän region (Lovén 1846). All studied specimens shared the characteristic for this species of an expanded white pigment ring at the ceratal tip which is continued as a thin white line down the ceratal body. This feature persists both in subadult and fully mature specimens. The latter differ from subadults in having a broader body and pinkish body colour.

#### Gulenia gen. n.

Taxon classificationAnimaliaNudibranchiaCoryphellidae

http://zoobank.org/CF1E550C-9D6B-40BF-A82F-B9E15E1336D9

**Figure 26.**
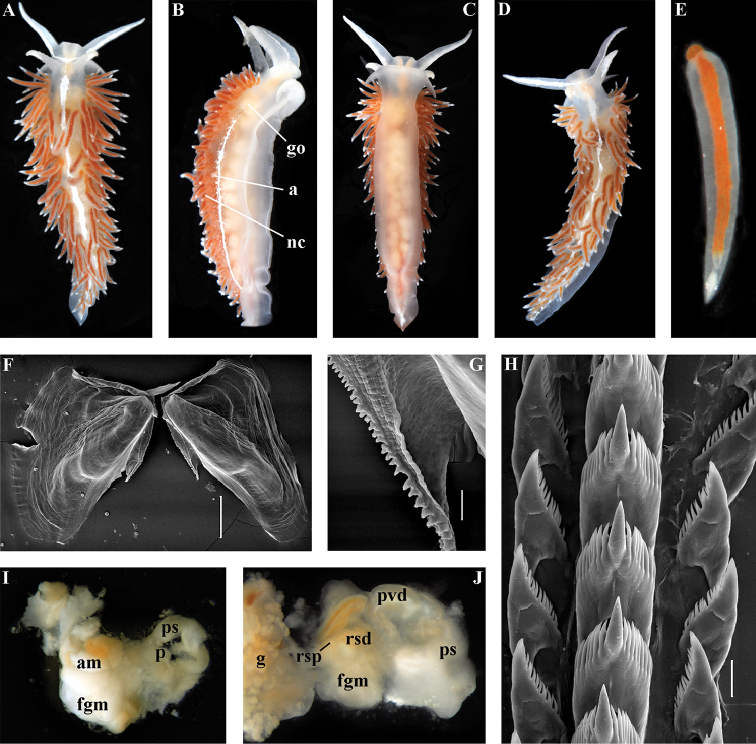
*Gulenia monicae* sp. n. Norwegian Sea, Gulen Dive Center. Holotype ZMMU Op-466, living specimen 34 mm length: **A** dorsal view **B** lateral view **C** ventral view; Paratype ZMMU Op-411, living specimen 24.5 mm length **D** dorsal view **E** details of cerata **F** jaw, SEM **G** details of masticatory process of jaw, SEM **H** radular teeth, posterior part, SEM **I J** reproductive system, light microscopy. Abbreviations: **a** anus **am** ampulla **fgm** female gland mass **g** gonad **go** genital opening **nc** continuous notal edge **p** penis **ps** penial sheath **pvd** prostatic vas deferens **rsd** distal receptaculum seminis **rsp** proximal receptaculum seminis. Scale bars: **F** = 300 μm; **G, H** = 30 μm. Photos and SEM images by T.A. Korshunova, A.V. Martynov.

**Figure 27.**
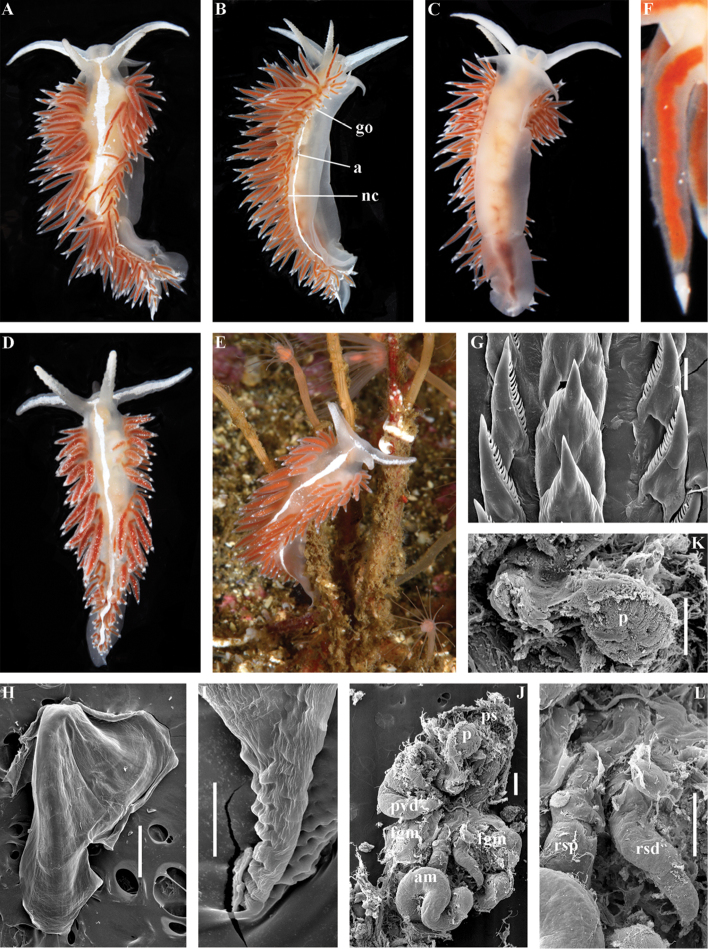
*Gulenia orjani* sp. n. Norwegian Sea, Gulen Dive Center. Holotype NTNU-VM-72482, living specimen 22 mm length: **A** dorsal view **B** lateral view **C** ventral view **F** details of cerata **D** Paratype ZMMU Op-538, living specimen 19 mm length, dorsal view **E** living specimens (not collected) on a hydroid colony **G** radular teeth, posterior part, SEM ZMMU Op-407 **H** jaw, SEM ZMMU Op-407 **I** details of masticatory process of jaw, SEM ZMMU Op-407 **J** reproductive system, SEM ZMMU Op-409 **K** penis, SEM ZMMU Op-409 **L** seminal reservoirs ZMMU Op-409. Abbreviations: **a** anus **am** ampulla **fgm** female gland mass **go** genital opening **nc** continuous notal edge **p** penis **ps** penial sheath **pvd** prostatic vas deferens **rsd** distal receptaculum seminis **rsp** proximal receptaculum seminis. Scale bars: **G** = 30 μm; **H, J, K, L** = 300 μm; **I** =30 μm. Photos and SEM images by T.A. Korshunova, A.V. Martynov.

Figs 21 , 25 , 26 , 27

##### Type species.

*Gulenia orjani* gen. et sp. n.

##### Etymology.

After Gulen Dive Resort (Norway) from where many of the studied materials come.

##### Diagnosis.

Body moderately wide. Notal ridge present, reduced, continuous. Cerata in continuous rows. Rhinophores smooth to wrinkled. Anterior foot corners present. Rachidian teeth with non-compressed broad cusp and distinct denticles. Lateral teeth denticulated with attenuated process basally. Distal and proximal receptaculum seminis. Vas deferens very short, expands to broad penial sheath. Penis broad, lobe shaped.

##### Species included.

*Gulenia borealis* (Odhner, 1922), comb. n. (Fig. 25), *Gulenia monicae* sp. n. (Fig. 26), *Gulenia orjani* sp. n. (Fig. 27).

##### Remarks.

See above under the genus *Fjordia*.

#### Gulenia monicae sp. n.

Taxon classificationAnimaliaNudibranchiaCoryphellidae

http://zoobank.org/47CAD149-1896-40C4-B94D-8F50C3C7640A

Fig. 26

Flabellina lineata *sensu*Malmberg and Lundin 2015 (pars.): 88.
Non Aeolis lineata Lovén, 1846: 8.

##### Type material.

Holotype, ZMMU Op-466, 34 mm long (live), Norway, entrance of the Sognefjord, Gulen Dive Resort, 17.03.2015, depth 29–30 m, coll. T.A. Korshunova, A.V. Martynov. 1 paratype, ZMMU Op-408, 23.5 mm long (live), Norway, entrance of the Sognefjord, Gulen Dive Resort, 17.03.2015, depth 20 m, coll. T.A. Korshunova, A.V. Martynov. 1 paratype, ZMMU Op-411, 24.5 mm long (live), Norway, entrance of the Sognefjord, Gulen Dive Resort, 17.03.2015, depth 10–20 m, coll. T.A. Korshunova, A.V. Martynov. 1 paratype, ZMMU Op-475, 25 mm long (live), Norway, entrance of the Sognefjord, Gulen Dive Resort, 02.04.2016, depth 20–25 m, coll. T.A. Korshunova, A.V. Martynov. 1 paratype, ZMMU Op-475, 31 mm long (live), Norway, entrance of the Sognefjord, Gulen Dive Resort, 02.04.2016, 25–30 m depth, coll. T.A. Korshunova, A.V. Martynov.

##### Type locality.

Gulen Dive Resort, Norway.

##### Etymology.

After Monica Bakkeli, proprietor of the Gulen Dive Resort, who significantly helped in organizing the scientific meetings and collecting activities in Gulen.

##### Diagnosis.

Continuous notal edge, background colour translucent white, digestive gland in cerata orange-brown to reddish-brown, apical parts of cerata without white pigment, usually small white spots scattered on dorsal face of cerata, thick opaque white lines on dorsal and lateral sides, rachidian tooth with up to nine distinct denticles, not delineated from central cusp, lateral teeth with up to 18 denticles on teeth edge, penis is broad lobe.

##### Description.

*External morphology* (Fig. 26A–E). Body relatively narrow. Foot and tail moderate, anterior foot corners long. Oral tentacles long and robust. Rhinophores ca. 1.5 times shorter than oral tentacles, slightly wrinkled, robust. Dorsal cerata fusiform, short, continuously attached to rudimentary but uninterrupted notal edge without forming clusters. Apices of cerata pointed. Notum narrow but distinct throughout both lateral sides of body. Digestive gland diverticulum fills significant volume of the cerata. Anal opening on right side below notal edge in first half of body but closer to middle. Reproductive openings lateral, below notal edges around middle part of body. Tail short and pointed, extending only short distance beyond last cerata.

*Colour* (Fig. 26A). Background colour translucent white. Digestive gland diverticula orange-brown to reddish-brown. Thick opaque white line runs along middle of whole dorsum from head to tail, varying in width and often spreading as lateral lobes amongst ceratal bases. Similar single thick median line runs on both lateral sides of body. Rhinophores similar in colour to body; apical parts covered with opaque white pigment. Dorsal sides of oral tentacles covered with thick opaque white line. No line along dorsal face of cerata, instead small white dots and speckles scattered over ceratal surfaces. Apical parts of cerata without opaque cap of white pigment.

*Jaws* (Fig. 26F, G). Masticatory process more than one-third as long as jaw body. Edge of masticatory processes bears about 60–80 denticles that continue to form several reduced rows of denticles on the body of the masticatory processes.

*Radula* (Fig. 26H). Radula formula: 16–17 × 1.1.1. Rachidian tooth elongate-triangular with very strong cusp of nearly 1/3 of the tooth length (Fig. 26H). Rachidian tooth bears eight or nine well-defined separated (but adpressed towards the cusp) long lateral denticles. Cusp delineated from the adjacent first lateral denticles. Lateral teeth (Fig. 26H) narrowly triangular with obtuse and distinctly attenuated posteriorly outer process and between 13 and 18 sharp long denticles on internal edge.

*Reproductive system* (Fig. 26I, J). Diaulic. Hermaphroditic duct leads to convoluted ampulla of about two whorls. Vas deferens very short, not attached to dorsal side of the penial sheath, no distinct prostate. Penial sheath large, wide. Penis is broad lobe. Oviduct connects through insemination duct into female gland complex. Vagina short and indistinct. Proximal and distal receptaculum seminis placed relatively close to each other (Fig. 26J).

##### Ecology.

Associates with the *Tubularia* colonies usually at depth 20–30 m. Feeds on small athecate hydroids and *Eudendrium* spp. which grow on the stalks of the *Tubularia* and the adjacent rock surfaces. This species is locally abundant. Egg mass is a narrow spiral cord. Reproduction period from February to May; the larva is a planktotrophic veliger with oval shell.

##### Distribution.

In the Northeast Atlantic has been found only in Norway (present study) and possibly in Sweden.

##### Remarks.

According to the present molecular phylogenetic analysis *Gulenia monicae* sp. n. forms a separate clade within the genus *Gulenia*, sister to *G. orjani* sp. n. The genetic distance of the mitochondrial barcode marker (COI) separates sympatric *G. monicae* sp. n. with high genetic divergence (11.3%). The mean COI p-distance value within the *G. monicae* sp. n. clade (5 specimens) is 0.45% and within the *G. orjani* sp. n. clade (14 specimens) is 0.41%. While this species shows a considerable minimal COI p-distance (10.97 ± 1.2%) compared to *G. orjani* sp. n., morphologically it is difficult to distinguish. *G. monicae* sp. n. can be tentatively distinguished externally from *G. orjani* sp. n. by a narrower body and shorter cerata.

#### Gulenia orjani sp. n.

Taxon classificationAnimaliaNudibranchiaCoryphellidae

http://zoobank.org/20E4709D-8ACE-49E5-8696-16508FE37953

Fig. 27

Flabellina lineata *sensu*Malmberg and Lundin 2015 (pars.): 88.
Non Aeolis lineata Lovén, 1846: 8.

##### Type material.

Holotype, NTNU-VM-72482, 22 mm long (live), Norway, entrance of the Sognefjord, Gulen Dive Resort, 19.03.2015, depth 20–30 m, coll. T.A. Korshunova, A.V. Martynov. 1 paratype, ZMMU Op-407, 30 mm long (live), Norway, entrance of the Sognefjord, Gulen Dive Resort, 18.03.2015, depth 20 m, coll. T.A. Korshunova, A.V. Martynov. 1 paratype, ZMMU Op-409, 27 mm long (live), Norway, entrance of the Sognefjord, Gulen Dive Resort, 18.03.2015, depth 20–30 m, coll. T.A. Korshunova, A.V. Martynov. 1 paratype, ZMMU Op-410, 22 mm long (live), Norway, entrance of the Sognefjord, Gulen Dive Resort, 18.03.2015, depth 20–30 m depth, coll. T.A. Korshunova, A.V. Martynov. 1 paratype, ZMMU Op-467, 35 mm long (live), Norway, entrance of the Sognefjord, Gulen Dive Resort, 02.04.2016, depth 10–15 m, coll. T.A. Korshunova, K. Malmberg, A.V. Martynov. 1 specimen, ZMMU Op-468, 19 mm long (live), Norway, entrance of the Sognefjord, Gulen Dive Resort, depth 20–25 m depth, 03.04.2016, coll. T.A. Korshunova, A.V. Martynov. 1 paratype, ZMMU Op-469, 33 mm long (live), Norway, entrance of the Sognefjord, Gulen Dive Resort, 02.04.2016, depth 20–25 m, coll. T.A. Korshunova, A.V. Martynov. 1 paratype, ZMMU Op-470, 21 mm long (live), Norway, entrance of the Sognefjord, Gulen Dive Resort, 03.04.2016, depth 25–30 m, coll. T.A. Korshunova, A.V. Martynov. 1 paratype, ZMMU Op-471, 19.5 mm long (live), Norway, entrance of the Sognefjord, Gulen Dive Resort, 05.04.2016, depth 20–30 m, coll. T.A. Korshunova, A.V. Martynov. 1 paratype, ZMMU Op-472, 22.5 mm long (live), Norway, entrance of the Sognefjord, Gulen Dive Resort, 07.04.2016, depth 20–30 m, coll. T.A. Korshunova, B. Picton. 1 paratype, ZMMU Op-473, 32 mm long (live), Norway, entrance of the Sognefjord, Gulen Dive Resort, depth 20–25 m, 03.04.2016, coll. T.A. Korshunova, A.V. Martynov. 1 paratype, ZMMU Op-474, 32 mm long (live), Norway, entrance of the Sognefjord, Gulen Dive Resort, depth 20–25 m, 03.04.2016, coll. T.A. Korshunova, A.V. Martynov. 1 paratype, ZMMU Op-538, 19 mm long (live), Norway, entrance of the Sognefjord, Gulen Dive Resort, 19.03.2015, depth 20 m depth, coll. T.A. Korshunova, A.V. Martynov.

##### Type locality.

Gulen Dive Resort.

##### Etymology.

After Ørjan Sandnes, proprietor of the Gulen Dive Resort where the majority of the material for this study has been collected, who has been immensely supportive of this work.

##### Diagnosis.

Continuous notal edge, background colour translucent white, digestive gland in cerata orange-brown to reddish-brown, sometimes almost blackish, apical parts of cerata without white pigment, usually small white spots scattered on dorsal face of cerata, thick opaque white lines on dorsal and lateral sides, rachidian tooth with up to nine distinct denticles, not delineated from rachidian cusp, lateral teeth with up to 18 denticles on teeth edge, penis is a broad lobe.

##### Description.

*External morphology* (Fig. 27A–E). Body relatively wide. Foot and tail moderate, anterior foot corners long. Oral tentacles long and robust. Rhinophores ca. 1.5 times shorter than oral tentacles, slightly wrinkled, robust. Dorsal cerata fusiform, long, continuously attached to rudimentary but uninterrupted notal edge without forming clusters. Apices of cerata pointed. Notum narrow but distinct throughout both lateral sides of body. Digestive gland diverticulum fills significant volume of cerata. Anal opening on right side below notal edge in first half of body but closer to middle. Reproductive openings lateral, below notal edge around middle part of body. Tail short and pointed, extending only short distance beyond last cerata.

*Colour* (Fig. 27A). Background colour translucent white. Digestive gland diverticula orange-brown to reddish-brown, sometimes dark brown to blackish. Thick opaque white line runs down middle of whole dorsum from head to tail, varying in width and often spreading as lateral lobes amongst ceratal bases. Similar single thick median line runs on both lateral sides of the body. Rhinophores similar in colour to body; apical parts covered with opaque white pigment. Dorsal sides of oral tentacles covered with thick opaque white line. No line along dorsal face of cerata, instead small white dots and speckles scattered over the surfaces of cerata (Fig. 27F). Apical parts of cerata without opaque cap of white pigment.

*Jaws* (Fig. 27H, I). Masticatory process more than one-third as long as jaw body. Edge of masticatory processes bears ca. 40–50 denticles that continue to form several reduced rows of denticles on body of the masticatory processes.

*Radula* (Fig. 27G). Radula formula: 16–19 × 1.1.1. Rachidian tooth elongate-triangular with very strong cusp of around 1/3 of the tooth length. Rachidian tooth bears eight or nine well-defined separated (but adpressed towards the cusp) long lateral denticles. Cusp strongly delineated from adjacent first lateral denticles. Lateral teeth narrowly triangular with obtuse and distinctly attenuated posteriorly outer process and between 13 and 18 sharp long denticles on internal edge.

*Reproductive system* (Fig. 27J–L). Diaulic. Hermaphroditic duct leads to convoluted ampulla of about two whorls. Vas deferens very short, not attached to dorsal side of penial sheath, no distinct prostate. Penial sheath large, wide. Penis is a broad lobe (Fig. 27K). Oviduct connects through insemination duct into female gland complex. Vagina short and indistinct. Proximal and distal receptaculum seminis placed close to each other and similar in size (Fig. 27L).

##### Ecology.

Associates with the *Tubularia* colonies usually at depth 20–30 m. Feeds on small athecate hydroids growing on the *Tubularia* stems and the adjacent rock as well as on *Eudendrium* species. Egg mass is a narrow cord. Reproduction period from February to April; the larva is a planktotrophic veliger with oval shell.

##### Distribution.

In the Northeast Atlantic has been found only in Norway (present study) and possibly in Sweden.

##### Remarks.

*Gulenia orjani* sp. n. is readily distinguished morphologically from other superficially similar taxa of the family Coryphellidae (e.g., *Fjordia lineata, F. chriskaugei*) as well as from the majority of the North Atlantic flabellinids by a combination of continuous notal edge with cerata not in clusters, a broad dorsal medial white line, and strong cusp of the rachidian radular tooth. Another distinctive feature of *G. orjani* sp. n. is absence of the white line on the dorsal surface of the cerata, which is substituted often by scattered white dots. The continuous notal edge resembles that in the genus *Chlamylla*; the latter, however, is very different in having a distinct granulose prostate and complicated external penial collar. Two North Atlantic genera and species of the family Coryphellidae also possess a continuous notal edge, namely *Gulenia borealis* and *Borealia nobilis*. However, unlike *G. orjani* both these taxa do not possess dorsal and lateral continuous white lines and differ considerably in radular and reproductive morphology. Molecular analysis corroborates the morphological data (see Discussion). Colour may vary significantly. The reddish colour of the digestive diverticulum in the cerata is most common; however, specimens with light brown and even almost black diverticulum may occur. The medial white line also may vary to a considerable degree - including specimens with broad and narrow lines. White pigment spots on the cerata may be dense or almost absent. For differences from *G. monicae* sp. n. see above.

#### Himatina

Taxon classificationAnimaliaNudibranchiaCoryphellidae

Thiele, 1931

Figs 21 , 28

##### Type species.

*Himatella trophina* Bergh, 1894

##### Diagnosis.

Body moderately wide. Notal ridge present, reduced, continuous. Cerata in continuous rows. Rhinophores perfoliated. Anterior foot corners present. Rachidian teeth with moderately compressed narrow cusp and distinct denticles. Lateral teeth denticulated with attenuated process basally. Distal and proximal receptaculum seminis. Short vas deferens expands to broad penial sheath. Penis broad, discoid.

##### Species included.

**Figure 28.**
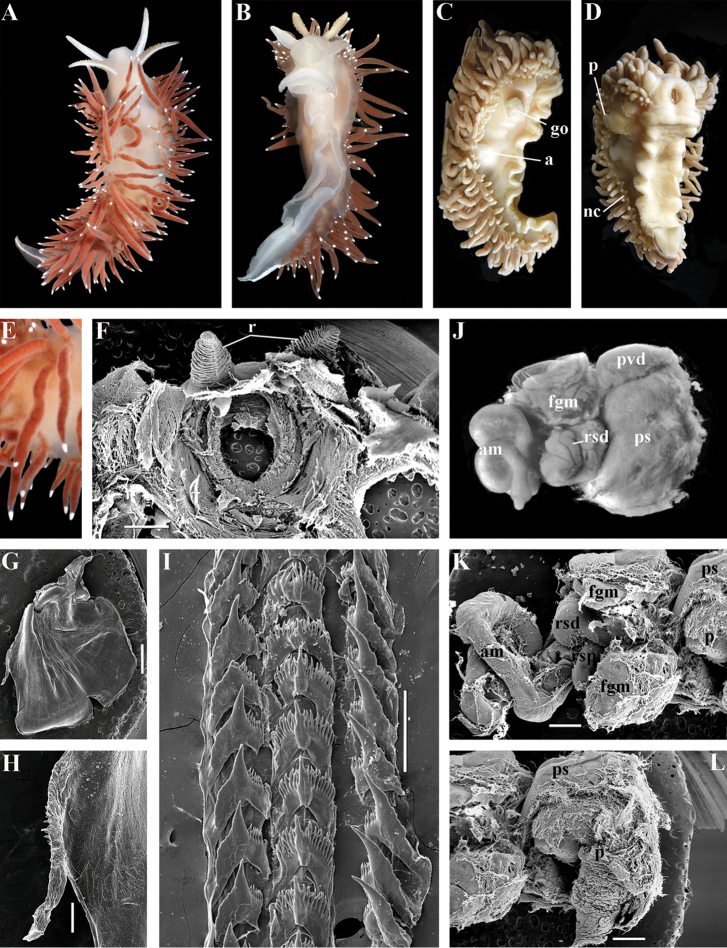
*Himatina trophina* (Bergh, 1894). North West Pacific, Kamchatka. ZMMU Op-29, living specimen 32 mm length: **A** dorsal view **B** ventral view ZMMU Op-532, fixed specimen 27 mm length **C** lateral view **D** ventral view **E** details of cerata ZMMU Op-29 **F** dissected anterior part (pharynx removed) and rhinophores, SEM **G** jaw, SEM **H** details of masticatory process of jaw SEM **I** radular teeth, posterior part, SEM **J** reproductive system, light microscopy **K** reproductive system SEM **L** penis, SEM. Abbreviations: **a** anus **am** ampulla **fgm** female gland mass **go** genital opening **nc** continuous notal edge **p** penis **ps** penial sheath **r** rhinophores **rsd** distal receptaculum seminis **rsp** proximal receptaculum seminis. Scale bars: **G, K, L** = 1 mm; **H** = 30 μm; **I** =10 μm. Photos and SEM images by T.A. Korshunova, A.V. Martynov.

*Himatina trophina* (Bergh, 1894) (Fig. 28) (original description in Bergh 1894).

##### Remarks.

The genus *Himatina* Thiele, 1931 was established as a replacement (Thiele 1931) for a single species *Himatella trophina* Bergh, 1894 and possesses a unique combination of morphological and molecular characteristics (Figs 1, 2). *Himatina trophina* is a wide-bodied coryphellid with continuous notal edge, perfoliated rhinophores and very short vas deferens and broad penis (Fig. 21). According to our molecular phylogenetic analysis *Himatina* forms a separate clade basally to the genera *Fjordia* and *Gulenia*. These morphological and molecular data clearly delineate *Himatina* from all known Atlantic and Pacific coryphellids.

#### Itaxia gen. n.

Taxon classificationAnimaliaNudibranchiaCoryphellidae

http://zoobank.org/843ACE1A-FFBC-4790-A1C5-0C97EB076F3A

Figs 21 , 29

##### Type species.

*Coryphella falklandica* Eliot, 1907

##### Etymology.

After *itax* (meaning “south” in Yagán (Yaghan), a nearly extinct language) because Yagáns are regarded as the southernmost peoples in the world who traditionally inhabited the very end of South America thus are close to the range of the only known coryphellid nudibranchs from the southern sub-Antarctic waters.

##### Diagnosis.

Body moderately wide. Notal ridge present, reduced, continuous. Cerata in continuous rows. Rhinophores wrinkled. Anterior foot corners present. Rachidian teeth with non-compressed broad cusp and distinct denticles. Lateral teeth denticulated without attenuated process basally. Receptaculum seminis unknown. Vas deferens very short, thick. Penis broad, lobe-shaped.

##### Species included.

**Figure 29.**
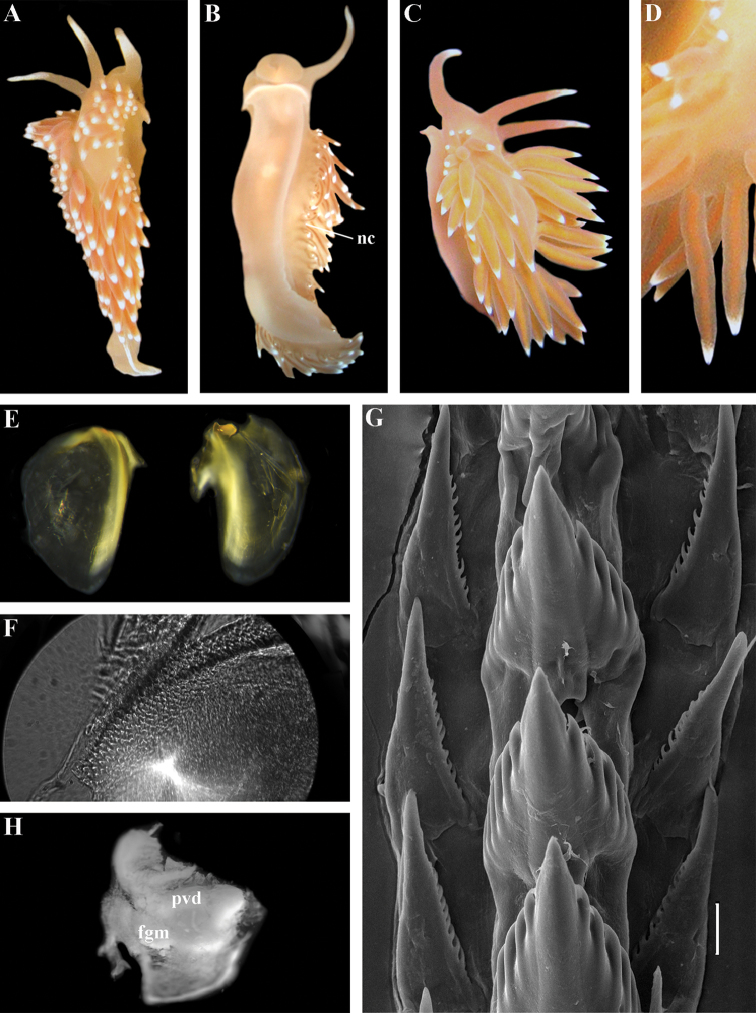
*Itaxia falklandica* (Eliot, 1907), comb. n. South Pacific, Chile, Canal Artilleria, ZSM Mol-20070592, specimen 12 mm length (fixed): **A** dorsal view (live) **B** ventral view (live) **C** lateral view (live) **D** details of cerata (live) **E** jaws, light microscopy **F** details of masticatory process of jaw, light microscopy **G** radular teeth, posterior part, SEM **H** reproductive system (non mature), light microscopy. Abbreviations: **fgm** female gland mass **nc** continuous notal edge **pvd** prostatic vas deferens. Scale bars: **G** = 30 μm. Photos of living specimens by M. Schrödl, other photos and SEM images by A.V. Martynov.

*Itaxia falklandica* (Eliot, 1907), comb. n. (Fig. 29) (original description in Eliot 1907, morphological data in Odhner 1944; Marcus 1959, Schrödl 2003; neotype from southern Chile (Bahía Mansa) designated by Schrödl 2003).

##### Remarks.

**Figure 30.**
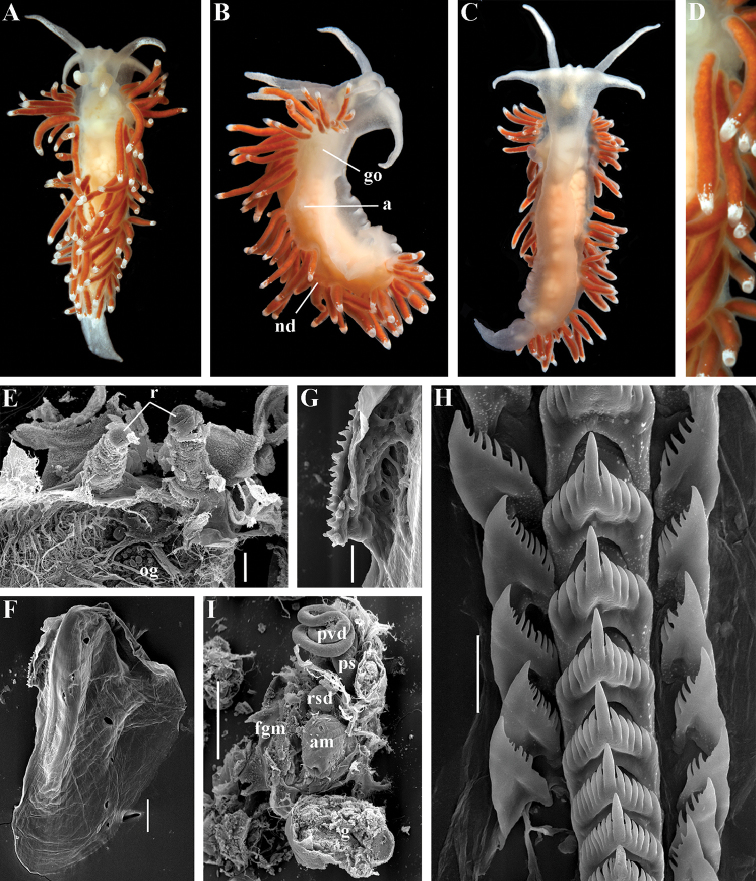
*Microchlamylla gracilis gracilis* (Alder & Hancock, 1844), comb. n. Norwegian Sea, Gulen Dive Center. ZMMU Op-502, living specimen 23 mm length: **A** dorsal view **B** lateral view **C** ventral view **D** details of cerata **E** dissected anterior part (pharynx removed) and rhinophores, SEM **F** jaw, SEM **G** details of masticatory process of jaw, SEM **H** radular teeth, posterior part, SEM **I** reproductive system, SEM. Abbreviations: **a** anus **am** ampulla **fgm** female gland mass **go** genital opening **nd** discontinuous notal edge **og** oral glands **ps** penial sheath **pvd** prostatic vas deferens **r** rhinophores **rsd** distal receptaculum seminis. Scale bars: **E, I** = 1 mm; **F** = 300 μm; **G, H** = 30 μm. Photos and SEM images by T.A. Korshunova, A.V. Martynov.

Externally the single included species *Itaxia falklandica* is similar to the species of the genus *Borealia*, with a moderately wide body and continuous notal margin; however, the reproductive system with short vas deferens and broad penis more closely resembles the genera *Gulenia* and *Fjordia*. In the present study, molecular data for *I. falklandica* was obtained for the first time from the specimens from southern Chile. Most unexpectedly, our molecular analysis places *Itaxia falklandica* as sister to the genus *Microchlamylla* (see below) which differs considerably from *Itaxia* by the presence of a discontinuous notal edge and a remarkable reproductive system with several loops of thin vas deferens without a distinct prostate and a small narrow penis (Figs 21, 30I).

#### Microchlamylla gen. n.

Taxon classificationAnimaliaNudibranchiaCoryphellidae

http://zoobank.org/8AF1E2E8-36E7-487B-975C-568A39FCDDC8

Figs 21 , 30 , 31

##### Type species.

*Eolis gracilis* Alder & Hancock, 1844

##### Etymology.

After *micro*- and *chlamylla*; in reference to the unusually long vas deferens of this genus typical for the genus *Chlamylla* of the family Paracoryphellidae, but smaller body size and discontinuous notal edge, common in the family Coryphellidae.

##### Diagnosis.

Body narrow. Notal edge present, moderately reduced, discontinuous, formed by a distinct series of lateral pieces. Cerata in several groups. Rhinophores smooth, similar in length or shorter than oral tentacles. Anterior foot corners present. Rachidian teeth with relatively wide cusp and distinct denticles. Lateral teeth denticulated without attenuated process basally. Separated distal and proximal receptaculum seminis. Long convoluted thin vas deferens expands distally to narrow penial sheath. Penis narrow, conical.

##### Species included.

**Figure 31.**
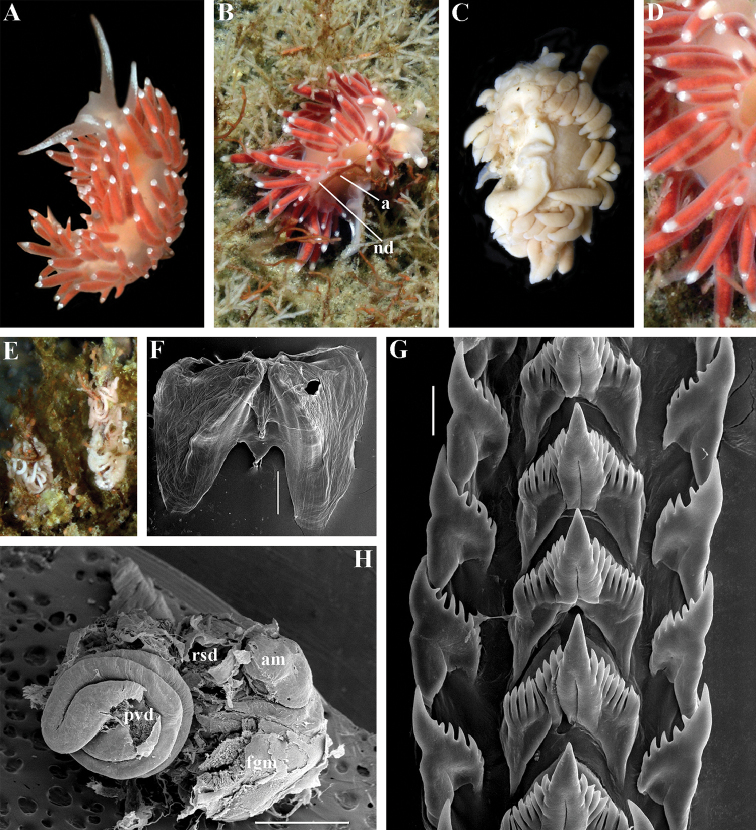
*Microchlamylla gracilis zfi* subsp. n. Arctic, Franz Josef Land. ZMMU Op-501, specimen 11 mm length (fixed): **A** dorsal view (live) **B** lateral view (live) **C** ventral view (fixed) **D** details of cerata **E** egg masses on hydroids **F** jaws, SEM **G** radular teeth, posterior part, SEM **H** reproductive system. Abbreviations: **a** anus **am** ampulla **fgm** female gland mass **nd** discontinuous notal edge **pvd** prostatic vas deferens **rsd** distal receptaculum seminis. Scale bars: **F** = 300 μm; **G** = 30 μm; **H** = 1 mm. Photos of living specimens and eggs by O.V. Savinkin, other photos and SEM images by A.V. Martynov

*Microchlamylla gracilis gracilis* (Alder & Hancock, 1844), comb. n. (Fig. 30) (original description in Alder and Hancock 1844), *Microchlamylla gracilis zfi* subsp. n. (Fig. 31), *Microchlamylla amabilis* (Hirano & Kuzirian, 1991), comb. n. (original description in Hirano and Kuzirian 1991).

##### Remarks.

The genus *Microchlamylla* gen. n. is clearly delineated from all known taxa of the family Coryphellidae by a combination of narrow body with a discontinuous notal edge, smooth rhinophores, and long narrow vas deferens which forms several loops (Fig. 21). The molecular phylogenetic analysis places *Microchlamylla* gen. n. in a clade very distinct from the rest of the coryphellids (Figs 1, 2) with the only relatively close genus being *Itaxia* gen. n. The genus *Microchlamylla* gen. n. is comprised of two species which are morphologically quite similar but molecularly well delineated - the North Atlantic *M. gracilis* and North Pacific *M. amabilis*.

#### Microchlamylla gracilis zfi subsp. n.

Taxon classificationAnimaliaNudibranchiaCoryphellidae

http://zoobank.org/435F800F-4A07-4583-844B-C1DD6D9D458C

Fig. 31

##### Type material.

Holotype. ZMMU Op-501, 11 mm long (fixed), Franz Josef Land, Northbrook Island, 26.08.2013, depth 20 m, coll. O.V. Savinkin.

##### Etymology.

After common acronym in Russian “ZFI” (Zemlya Franza Iosifa) for Franz Josef Land.

##### Type locality.

Franz Josef Land.

##### Diagnosis.

Discontinuous notal edge, background colour translucent white, digestive gland in cerata dark red, apical parts of cerata with white pigment, rachidian tooth with up to ten distinct denticles, delineated from central cusp, lateral teeth with up to eleven denticles on teeth edge, penis conical.

##### Description.

*External morphology* (Fig. 31A–D). Body relatively narrow. Foot and tail moderate, anterior foot corners long. Oral tentacles long. Rhinophores ca. 1.5 times longer than oral tentacles, slightly wrinkled. Dorsal cerata finger-shaped to fusiform, forming several clusters along dorsal edges. Apices of cerata gradually pointed, with elongate cnidosac. Distinct notal edge remains mostly below cerata clusters. Digestive gland diverticulum fills significant volume of the cerata. Anal opening on right side below second large cluster cerata. Reproductive openings lateral, below first anterior cluster of cerata.

*Colour* (Fig. 31A, B, D). Background colour translucent white. Digestive gland diverticula dark red. Rhinophores background colour similar to body; along dorsal side of rhinophores runs thin white line. Dorsal sides of oral tentacles covered with thin opaque white line. Apical parts of cerata with opaque cap of white pigment.

*Jaws* (Fig. 31F). Masticatory process more than one-third as long as jaw body. Edge of masticatory processes bears several denticles that continue to form several reduced rows of denticles on the body of the masticatory processes.

*Radula* (Fig. 31G). Radula formula: 21 × 1.1.1. Rachidian tooth elongate-triangular with very broad cusp up to 1/2 of the tooth length, with a peculiar notch at the base. Rachidian tooth bears between seven and ten well-defined lateral denticles not adpressed towards the cusp. Some denticles form clusters, outermost denticles are considerably enlarged. Cusp is delineated from the adjacent first lateral denticles. Lateral teeth (Fig. 31G) broadly triangular with obtuse and attenuated posteriorly outer process and between five and eleven sharp denticles of various lengths on the internal edge.

*Reproductive system* (Fig. 31H). Diaulic. Hermaphroditic duct leads to swollen short ampulla. Vas deferens is long, broad, comprising at least three whorls, no distinct prostate. Penial sheath is narrow. Oviduct connects through insemination duct into female gland complex. Vagina short and indistinct. Receptaculum seminis oval, swollen. Distal receptaculum seminis present.

##### Ecology.

Associates with hydroid colonies usually at depths around 20 m. Apart from the holotype, several more specimens of this new subspecies can be traced from the original in situ photographs, but they were not collected. The egg mass is narrow cord that forms irregular, compressed pink or off-white spirals (Fig. 31E). Reproduction recorded in August.

##### Distribution.

So far known only from the northernmost locality, Franz Joseph Land Archipelago.

##### Remarks.

*Microchlamylla gracilis zfi* subsp. n. differs considerably from *Microchlamylla gracilis gracilis* by a peculiarly enlarged base with a prominent notch of the cusp of the rachidian radular teeth (compare Fig. 30H and Fig. 31G). In *M. gracilis gracilis* such a character was never reported before, either in literature (e.g., Odhner 1939; Thompson and Brown 1984) or according to our study of the specimens from geographically very distant regions, including the White Sea and southwest Norway. The lateral denticles of the rachidian teeth of *M. gracilis zfi* subsp. n. demonstrate considerable differences from *M. gracilis gracilis* in the presence of clustered denticles and very enlarged outermost lateral denticles (compare Fig. 30H and Fig. 31G). In addition, the vas deferens is much broader in *M. gracilis zfi* subsp. n. and differs considerably from *M. gracilis gracilis* (compare Fig. 30I and Fig. 31H). Thus, morphologically *M. gracilis zfi* subsp. n. is well-distinguished from the nominative subspecies; however, according to the molecular data, these two taxa are similar (maximal COI p-distance is 0.62% whereas mean COI p-distance value within the *M. gracilis* clade is 0.56%) and therefore we here consider their status to be at the subspecies level. More study is clearly necessary in this case. *Microchlamylla gracilis* *s. l.* was never reported prior to this study from Franz Josef Land and the presence of a separate taxon in this region can be explained also by isolation of this Archipelago from the main North Atlantic streams. This agrees with other our findings; namely, the presence of a new species of paracoryphellid *Ziminella circapolaris* sp. n. at Franz Josef Land (this study) and a new species of the onchidoridid genus *Adalaria* (Martynov and Korshunova 2017).

#### Occidentella gen. n.

Taxon classificationAnimaliaNudibranchiaCoryphellidae

http://zoobank.org/3C99AC04-613F-4495-92B1-F2593AC70074

Figs 21 , 32

##### Type species.

*Coryphella athadona* Bergh, 1875.

##### Etymology.

After the Western Pacific (from *occidens* = “west” in Latin), where the type species predominantly occurs.

##### Diagnosis.

Body narrow. Notal ridge completely reduced. Cerata in several groups. Rhinophores smooth. Anterior foot corners absent. Rachidian teeth with non-compressed cusp and distinct denticles. Lateral teeth denticulated without attenuated process basally. Distal and proximal receptaculum seminis. Vas deferens very short, expanding into broad penial sheath with an additional glandular formation. Penis small, amorphous.

##### Species included.

**Figure 32.**
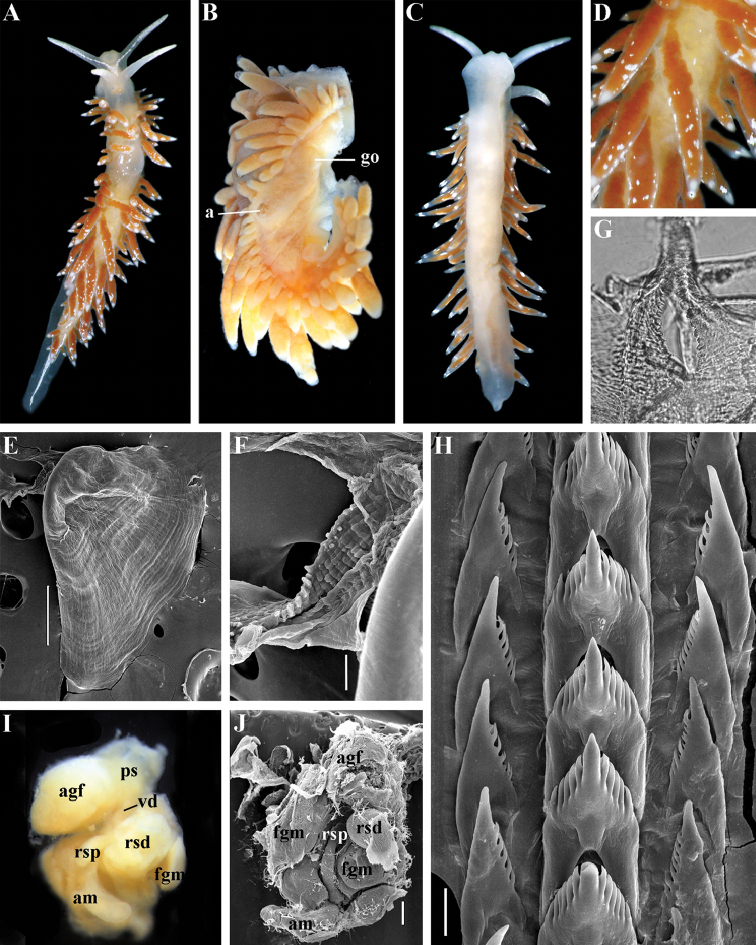
*Occidentella athadona* (Bergh, 1875), comb. n. North West Pacific, Kamchatka. ZMMU Op-498, living animal 19 mm length: **A** dorsal view **B** lateral view (fixed) **C** ventral view **D** details of cerata **E** jaw, SEM, scale bar = 1 mm **F** details of masticatory process of jaw, SEM **G** details of masticatory process of jaw, light microscopy **H** radular teeth, posterior part, SEM **I** reproductive system, light microscopy **J** reproductive system, SEM. Abbreviations: **a** anus **agf** additional glandular formation (modified prostate), **am** ampulla **fgm** female gland mass **go** genital opening **ps** penial sheath **rsd** distal receptaculum seminis **rsp** proximal receptaculum seminis **vd** vas deferens (very rudimentary, rapidly transits to greatly enlarged modified prostate (**agf**). Scale bars: **E** = 1 mm; **F, H** = 30 μm; **J** = 300 μm. Photos and SEM images by T.A. Korshunova, A.V. Martynov.

*Occidentella athadona* (Bergh, 1875), comb. n. (Fig. 32) (original description in Bergh 1875; redescription in Baba 1987a)

##### Remarks.

The genus *Occidentella* with only a single known species *O. athadona* has unique external and internal morphological features compared to all described Coryphellidae taxa: a rounded anterior part of the foot (no foot corners) and a highly developed glandular part of vas deferens which is similar to a true penial gland in some other families (e.g., Tergipedidae and Eubranchidae) (Fig. 21). Millen and Hermosillo (2007) indicated that some other taxa, e.g., *Coryphella trilineata* possess in a some degree a similar glandular structure; however, the penial complex in *C. trilineata* is still similar (see MacFarland 1966) to other coryphellids and not so obviously separated from the penial sheath as in *O. athadona*. By other features, e.g., presence of annulated rhinophores and anterior foot corners, *C. trilineata* differs considerably from *O. athadona*. Possibly in agreement with data on the similarity of a glandular formation in the penis, *C. trilineata* is placed within the same clade with *O. athadona* according to our molecular analysis (Figs 1, 2). However, this result may also be because we do not have molecular data for other coryphellids which are morphologically similar to *C. trilineata* from the Eastern Pacific (e.g., *C. cooperi* (Cockerell, 1901), *Flabellina fogata* Millen & Hermosillo, 2007) and *C. trilineata* together with these other species will probably be further separated from *O. athadona*. However, because it is morphologically very different from all other coryphellid taxa, *C. athadona* deserves its own genus. This is consistent with one of the general rules of integration of morphological and molecular data which were outlined above (see Materials and methods section), when a morphologically strongly aberrant taxon should not be merged with a phylogenetically relatively close taxon. This implies that if we merge the morphologically strongly aberrant *C. athadona* with the morphologically different but phylogenetically possibly relatively close *C. trilineata* we will considerably mask the natural diversity and will therefore make further classification much more difficult. If more species are discovered within these species complexes, we will need to compare them with morphologically very disparate taxa instead of making a comparison within narrow morphological and molecular units. According to these principles, the species *Coryphella trilineata* O’Donoghue, 1921 is separated here into another new genus (see below), within which we tentatively include at least two more Eastern Pacific species *C. cooperi* and *Flabellina fogata* Millen & Hermosillo, 2007 with similar external characters (rhinophores annulated in two species and warted in one species) and reproductive features (glandular formation at the base of penis).

#### Orientella gen. n.

Taxon classificationAnimaliaNudibranchiaCoryphellidae

http://zoobank.org/BA1C449B-CB08-4D96-946F-FBA1CCF52FF1

Fig. 21

##### Type species.

*Coryphella trilineata* O’Donoghue, 1921.

##### Etymology.

After *orientis* (Latin, east), in reference to the predominantly Eastern Pacific range of the type species *C. trilineata* and further species included in the genus.

##### Diagnosis.

Body narrow. Notal ridge discontinuous. Cerata in several groups. Rhinophores annulated or tuberculated. Anterior foot corners present. Rachidian teeth with non-compressed cusp and distinct denticles. Lateral teeth denticulated without attenuated process basally. Distal and proximal receptaculum seminis. Vas deferens very short, expands into broad penial sheath with an additional glandular formation. Penis discoid with small papillae.

##### Species included.

*Orientella* (?) *cooperi* (Cockerell, 1901), comb. n. (original description in Cockerell 1901), *Orientella* (?) *fogata* (Millen & Hermosillo, 2007), comb. n. (original description in Millen and Hermosillo 2007), *Orientella trilineata* (O’Donoghue, 1921), comb. n. (original description in O’Donoghue 1921). Probably this clade and genus includes more Eastern Pacific coryphellid species, since several undescribed species have already been reported from Chile (Schrödl 2003).

#### Family Coryphellidae incertae sedis

Taxon classificationAnimaliaNudibranchiaCoryphellidae

##### Species included.

*Coryphella abei* Baba, 1987 (original description in Baba 1987b), *Coryphella verta* Marcus, 1970 (original description in Marcus 1970, redescription in Millen and Hamann 2006).

#### Family Flabellinidae

Taxon classificationAnimaliaNudibranchiaFlabellinidae

Bergh, 1889, restricted

##### Diagnosis.

Body commonly narrow. Notal edge discontinuous or fully reduced. Cerata in separate clusters, on elevations or distinct stalks. Rhinophores smooth, annulated or papillated. Anus mixed (pleuroproctic in higher acleioproctic position) or pleuroproctic under the reduced notal edge. Distinct oral glands present, commonly penetrate below anterior cerata. Radula formula 1.1.1. Rachidian teeth usually compressed by adjacent lateral denticles. Lateral teeth with attenuated process basally, usually denticulated, rarely smooth. Number and position of receptaculum seminis variable: two separate ones, or double proximal and single distal, or double distal one, in few cases proximal receptaculum not evident. Vas deferens usually long, with indistinct prostate. External permanent penial collar absent. Penis usually elongated conical, narrow, always internal unarmed.

##### Genera included.

*Calmella* Eliot, 1910, *Carronella* gen. n., *Coryphellina* O’Donoghue, 1929, *Edmundsella* gen. n., *Flabellina* Gray, 1833 in Griffith and Pidgeon, 1833–1834, *Paraflabellina* gen. n., *Piseinotecus* (?) Marcus, 1955.

##### Remarks.

The name Flabellinidae has been used for a long time to encompass all the diversity of aeolidaceans with a triseriate radula and without a clearly defined supplementary (or penial) gland in the male part of reproductive system. However, according to our present molecular phylogenetic analysis (Figs 1, 2), the former Flabellinidae form several distinct clades. Remarkably, some of them appear to be closely related to traditional Tergipedidae or Facelinidae thus making any notion that traditional Flabellinidae can be saved as a monophyletic unit highly unlikely.

Therefore, following the phylogenetic pattern discovered from the DNA analysis (Fig. 1) and consolidated morphological data (Fig. 2) we consistently utilise a differential approach, and propose several family level groups within traditional “Flabellinidae”. The super-cluster of the majority of traditional Flabellinidae holds a central position within this group and is apparently monophyletic (Fig. 2). It is, however, comprised of three clearly defined clades, which roughly correspond to the traditional genera *Chlamylla*, *Coryphella*, and *Flabellina*. However, a decision to maintain these phylogenetic superclusters just at generic levels will considerably obscure the complicated evolutionary patterns discovered from the molecular analysis (Fig. 1) which must be consistently reflected in a classification. Importantly, every large phylogenetic cluster has clear morphological trends. For example, the reinstated family Paracoryphellidae is characterised by the invariable presence of a wide body and continuous notal edge, elongated penis, and presence of complicated external penial collars or external permanent penis in two genera, and also invariably an uncompressed cusp of the rachidian teeth. The reinstated family Coryphellidae contains mostly taxa with a narrow body and discontinuous notal edge, but importantly there are several genera not directly related to other coryphellids (e.g., *Borealia*, *Himatina*, *Gulenia*, *Itaxia*, see above) that possess a continuous notal edge, which are all basal to various clades of narrow-bodied coryphellids. Internally most of the genera of the family Coryphellidae possess a non-compressed cusp of the rachidian teeth, short vas deferens, and broad penis always without an external penial collar. In turn, the considerably restricted family Flabellinidae *s. str.* includes taxa which usually have various ceratal elevations, up to highly ramified stalks in the genus *Flabellina* *s. str.* The family Flabellinidae in the restricted sense also includes such clear morphological trends as an almost invariably compressed cusp of the rachidian teeth, lateral teeth with attenuated bases, commonly large oral glands and usually a relatively long vas deferens and elongated penis (see below).

The minimum uncorrected p-distances of the mitochondrial barcode marker (COI) between the species of family Flabellinidae are given in Table 2. The minimum uncorrected p-distances between all genera range from 13.1 to 20.2%. The lowest distances occurred between *Coryphellina rubrolineata* and *Edmundsella pedata* (13.1%). Such considerable molecular divergence between taxa of the family Flabellinidae supports the reliability of the multi-genera approach consistently applied in this study.

**Table 2.** Minimum uncorrected p-distances (%) between representatives of the genera of the family Flabellinidae.

	*Paraflabellina funeka*	*Calmella cavolini*	*Carronella pellucida*	*Flabellina affinis*	*Coryphellina rubrolineata*	*Edmundsella pedata*
***Paraflabellina funeka***	-	18.0%	20.2%	16.5%	19.8%	19.1%
***Calmella cavolini***	18.0%	-	17.6%	17.4%	17.3%	17.3%
***Carronella pellucida***	20.2%	17.6%	-	17.0%	17.9%	16.2%
***Flabellina affinis***	16.5%	17.4%	17.0%	-	16.6%	15.0%
***Coryphellina rubrolineata***	19.8%	17.3%	17.9%	16.6%	-	13.1%
***Edmundsella pedata***	19.1%	17.3%	16.2%	15.0%	13.1%	-

#### Calmella

Taxon classificationAnimaliaNudibranchiaFlabellinidae

Eliot, 1910

Figs 33 , 34

##### Type species.

*Eolidia cavolini* Vérany, 1846.

##### Diagnosis.

Body narrow. Notal edge completely absent. Cerata on short stalks in several groups. Rhinophores smooth, larger than oral tentacles or similar in size. Anterior foot corners present. Anus mixed: pleuroproctic shifted towards dorsal acleioproctic position. Distinct oral glands. Rachidian teeth with narrow compressed cusp and distinct denticles. Lateral teeth weakly denticulated to smooth with attenuated process basally. Single distal receptaculum seminis. Moderately long prostatic non-granulated vas deferens. Penis conical.

##### Species included.

**Figure 33.**
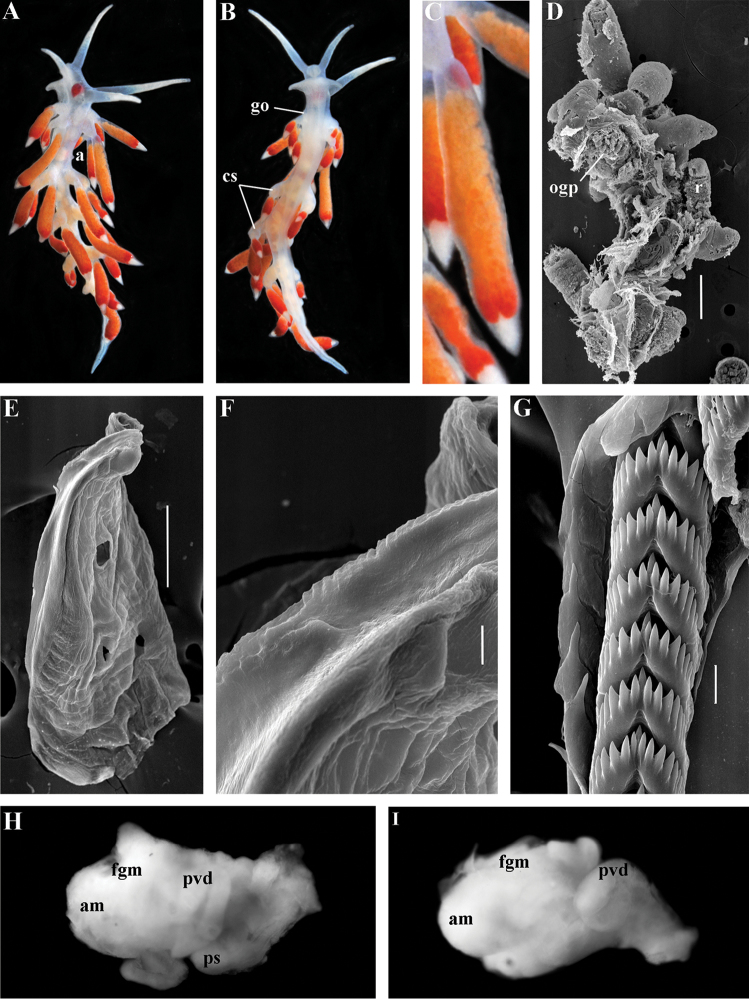
*Calmella cavolini* (Vérany, 1846). Mediterranean Sea, Banyuls-sur-Mer. ZMMU Op-485, living specimen 12 mm in length: **A** dorsal view **B** ventral view **C** details of cerata **D** dissected anterior part (pharynx removed) and rhinophores **E** jaw, SEM **F** details of masticatory process of jaw, SEM **G** radular teeth, posterior part, SEM **H I** reproductive system, light microscopy. Abbreviations: **a** anus **am** ampulla **cs** ceratal stalks **fgm** female gland mass **go** genital opening **ogp** oral gland penetrating into basis of cerata **r** rhinophores **ps** penial sheath **pvd** prostatic vas deferens. Scale bars: **D, E** = 300 μm; **F, G** = 10 μm. Photos and SEM images by T.A. Korshunova, A.V. Martynov.

*Calmella cavolini* (Vérany, 1846) (Fig. 33) (original description in Vérany 1846, detailed description in Schmekel and Portmann 1982), *Calmella gaditana* (Cervera, García-Gómez & García, 1987), comb. n. (original description in Cervera et al. 1987, = “*Flabellina*” *confusa* González-Duarte et al. 2008, original description in González-Duarte et al. 2008; for discussion see Furfaro et al. 2017), *Calmella bandeli* Marcus, 1976 (original description in Marcus 1976).

##### Remarks.

The genus *Calmella* was established by Eliot (1910) and since that time it has never been synonymised with *Flabellina*, until the most recent Furfaro et al. (2017) study. According to the present phylogenetic analysis *Calmella* is valid, related to the true *Flabellina* *s. str.* (Fig. 2) and differs morphologically regarding its much less ramified and raised ceratal stalks and presence of smooth rhinophores. See remarks also under the genus *Piseinotecus*.

#### Carronella gen. n.

Taxon classificationAnimaliaNudibranchiaFlabellinidae

http://zoobank.org/3B991AB9-A639-4974-AC7C-B3D600F6E9D1

Figs 34 , 35 , 36

##### Type species.

*Eolis pellucida* Alder & Hancock, 1843.

##### Etymology.

After Loch Carron, a sea loch on the west of the Scottish Highlands, where numerous specimens of *Eolis pellucida* have been observed over many years, feeding on *Eudendrium arbusculum* growing on flame shell (*Limaria hians*) reefs.

##### Diagnosis.

Body relatively wide. Notal ridge present, reduced, discontinuous, in several indistinct pieces which continue to low elevations of cerata, in several groups. Rhinophores smooth, similar in size to oral tentacles. Anterior foot corners present. Anus pleuroproctic. Distinct oral glands. Rachidian teeth with narrow compressed cusp and distinct denticles. Lateral teeth smooth with attenuated process basally. Distal and proximal receptaculum seminis. Moderately long vas deferens without distinct prostate. Penis conical.

##### Species included.

*Carronella enne* sp. n., *Carronella pellucida* (Alder & Hancock, 1843), comb. n. (Fig. 36) (original description in Alder and Hancock 1843; original description of radula in Alder and Hancock 1845–1855).

##### Remarks.

The genus *Carronella* gen. n. differs from other genera of the family Flabellinidae *s. str.* by the absence of distinct ceratal elevations or stalks and by the presence of smooth lateral denticles. The absence of distinct elevations or stalks represents a plesiomorphic feature, but according to the molecular phylogenetic analysis *Carronella* appears in a quite derived position (Figs 1, 2). This is therefore one more interesting case of discrepancy between morphological and molecular data that needs to be further investigated.

#### Carronella enne sp. n.

Taxon classificationAnimaliaNudibranchiaFlabellinidae

http://zoobank.org/E7E79BB2-941A-4580-9219-B9D7DE8FDEC3

Fig. 35

##### Type material.

Holotype, ZMMU Op-526, 5 mm long, North Atlantic W of Ireland, 48.7089'N, 10.5607'W, 11.06.2014, depth 1131 m, collected by Enrico Schwabe.

##### Type locality.

North Atlantic west of Ireland.

##### Etymology.

In honour of our friend, the malacologist Enrico (Enne) Schwabe (Bavarian State Collection of Zoology, Munich).

##### Diagnosis.

Mostly reduced notal edge, forming several clusters, background colour translucent white, digestive gland in cerata dull reddish, apical parts of cerata without white pigment, rachidian tooth with up to ten distinct denticles, adpressed to central cusp, lateral teeth smooth, penis conical.

##### Description.

*External morphology* (Fig. 35A–E). Body relatively wide. Foot and tail narrow, anterior foot corners long. Rhinophores ca. 1.5 times longer than oral tentacles, slightly wrinkled. Dorsal cerata finger-shaped to fusiform, forming several clusters along dorsal edges. Apices of cerata gradually pointed, with elongate cnidosac. Distinct notal edge remains mostly below ceratal clusters and forms slight elevations. Digestive gland diverticulum fills significant volume of the cerata. Anal opening pleuroproctic on right side in between first and second large ceratal clusters. Reproductive openings lateral, below first anterior cluster of cerata.

*Colour* (Fig. 35A). Background colour translucent white. Digestive gland diverticula dull reddish. There are no opaque white lines on the body. Rhinophore background colour similar to body; a thin white line runs along dorsal side of rhinophores runs; apical white pigment absent. Apical parts of cerata with opaque cap of white pigment.

*Jaws* (Fig. 35G, H). Masticatory process more than one-third as long as jaw body. Edge of masticatory processes bears ca. 30 denticles that continue to form several reduced rows of denticles on body of masticatory processes.

*Radula* (Fig. 35I). Radula formula: 24 × 1.1.1. Rachidian tooth elongate-triangular with short narrow cusp of less than 1/3 of the tooth length (Fig. 35I). Rachidian tooth bears between eight and ten well-defined separated long lateral denticles strongly adpressed towards the cusp. Cusp is compressed by adjacent first lateral denticles. Lateral teeth (Fig. 35I) broadly triangular with obtuse and very attenuated posteriorly outer process, completely smooth.

*Reproductive system* (Fig. 35J). Diaulic. Hermaphroditic duct leads to convoluted ampulla of about two whorls. Vas deferens moderately long, no distinct prostate. Penial sheath small. Penis narrow conical. Oviduct connects through insemination duct into female gland complex. Vagina short and indistinct. Proximal receptaculum seminis oval. Distal receptaculum seminis present, small.

##### Ecology.

Deep sea species (deeper than 1000 m).

##### Distribution.

Northeast Atlantic, off Ireland.

##### Remarks.

According to the molecular phylogenetic analysis *Carronella enne* sp. n. forms a separate sister clade to *Carronella pellucida* (Fig. 1). Morphological analysis reveals differences in proportions of lateral teeth in *C. enne* sp. n. The lateral teeth of *C. enne* sp. n. are more elongated (Fig. 35I) than in *C. pellucida* (Fig. 36H).

#### Coryphellina

Taxon classificationAnimaliaNudibranchiaFlabellinidae

O’Donoghue, 1929

**Figure 34.**
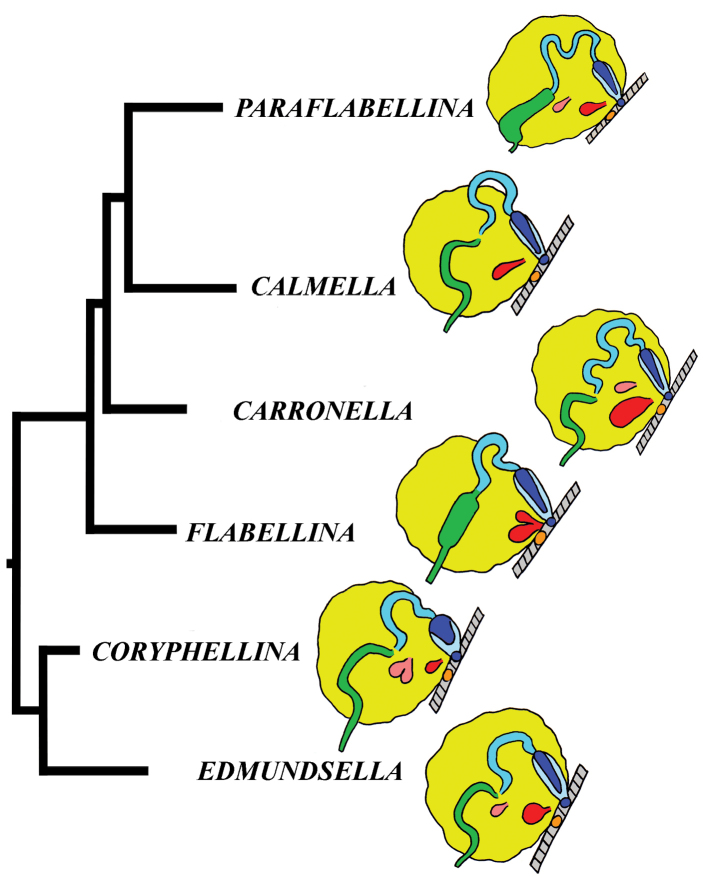
Schematic outline of the reproductive systems of the taxa of the family Flabellinidae integrated with molecular phylogenetic data. Colour indication of reproductive system characters: ampulla – green; body wall – gray; distal receptaculum seminis – red; female gland mass – yellow; female genital opening – orange; penis and male genital opening – dark blue; penial sheath – pale blue; prostatic vas deferens – turquoise; proximal receptaculum seminis – pink.

Figs 34 , 37 , 38 , 39

= Nossis Bergh, 1902; non Nossis Kinberg, 1865 (senior homonym).

##### Type species.

*Coryphellina rubrolineata* O’Donoghue, 1929.

##### Diagnosis.

Body narrow. Notal ridge present, reduced, discontinuous, in several indistinct pieces. Cerata on low elevations, in several groups. Rhinophores similar in length or shorter than oral tentacles, densely papillated. Anterior foot corners present. Anus pleuroproctic. Distinct oral glands. Rachidian teeth with narrow compressed cusp and distinct denticles. Lateral teeth denticulated with attenuated process basally. Proximal receptaculum seminis usually bilobed. Distal receptaculum seminis present. Short prostatic, not granulated vas deferens. Penis conical to bulbous.

##### Species included.

*Coryphellina albomarginata* (Miller, 1971) comb. n. (original description in Miller 1971), *Coryphellina arveloi* (Ortea & Espinosa, 1998), comb. n. (original description in Ortea and Espinosa 1998), *Coryphellina* (?) *delicata* (Gosliner & Willan, 1991), comb. n. (original description in Gosliner and Willan 1991), *Coryphellina exoptata* (Gosliner & Willan, 1991), comb. n. (Fig. 37) (original description in Gosliner and Willan 1991), *Coryphellina* (?) *hamanni* (Gosliner, 1994), comb. n. (original description in Gosliner 1994), *Coryphellina indica* (Bergh, 1902) comb. n. (original description in Bergh 1902), *Coryphellina lotos* sp. n. (Fig. 38), *Coryphellina marcusorum* (Gosliner & Kuzirian, 1990), comb. n. (original description in Gosliner and Kuzirian 1990), *Coryphellina* (?) *poenicia* (Burn, 1957), comb. n. (original description in Burn 1957), *Coryphellina rubrolineata* O’Donoghue, 1929 (Fig. 39) (original description in O’Donoghue 1929), *Coryphellina* (?) *westralis* (Burn, 1964), comb. n. (original description in Burn 1964).

##### Remarks.

The genus *Coryphellina* is well delineated from all Flabellinidae *s. str.* taxa. Morphologically most of *Coryphellina* species are characterised by papillated rhinophores and a peculiar bilobed receptaculum seminis. Externally *Coryphellina* has a discontinuous but still distinct notal edge which forms only slightly raised ceratal elevations (compared to the compound stalks in the true *Flabellina* and *Calmella*). These morphological characteristics are consistent with the molecular phylogenetic analysis where all *Coryphellina* place in a compact clade (Fig. 2). However, the type species *Coryphellina rubrolineata* forms a separate subclade within other *Coryphellina*. This may be consistent with previous information (Gosliner and Willan 1991) that *C. rubrolineata* has a “triaulic” reproductive system, which is unusual for most aeolidaceans. Since the fine precise relation between postampullar ducts and ducts of seminal reservoirs has not been studied in detail in the majority of traditional Flabellinidae and also because putative triauly in *C. rubrolineata* is different from well-defined “typical” triauly of dorid nudibranchs, we consider all flabellinids with papillated rhinophores and a bilobed receptaculum seminis as belonging to the same genus *Coryphellina*.

The genus *Nossis* Bergh, 1902 was established for a type species of *N. indica* from the Gulf of Siam (Bergh 1902). When he established the genus *Coryphellina* O’Donoghue (1929) did not mention the previously described genus *Nossis* but later a close relationship between *Nossis* and *Coryphellina* was noticed (e.g., Nordsieck 1972; McDonald 2009) and the type species of the genus *Coryphellina* has been treated under the name *Nossis rubrolineata*. However, the genus name *Nossis* Bergh, 1902 for nudibranchs was preoccupied by the name *Nossis* Kinberg, 1865 which was established for a polychaete (Kinberg 1865). Therefore these two names are homonyms, and the junior one should therefore be replaced with the available name *Coryphellina* O’Donoghue, 1929. In the present study we have investigated two syntypes of *Nossis indica* Bergh, 1902 (NHMD GAS-2008) and confirm that their external characters are concordant with the genus *Coryphellina*. We also find in both syntypes of *Nossis indica* densely papillated rhinophores essentially similar to those in the genus *Coryphellina*, contrary to the description of rhinophores of *N. indica* in the first description of Bergh (1902: 211) as bearing “leaves” that instead imply a perfoliate condition (Burn 1964). Perhaps, Bergh recognized the rhinophoral papillae but just described it as “leaves”. In addition, Burn (1964) also described another species of *Nossis, N. westralis*, and mentioned the genera *Nossis* and *Coryphellina*, but did not synonymise them; instead he highlighted their differences most likely because of the description of *N. indica* in Bergh (1902) regarding the rhinophoral leaves (instead of rhinophoral papillae). Accordingly, the species *Nossis indica* is transferred here to the genus *Coryphellina*, but its exact relationship to other common Indo-West Pacific *Coryphellina* such as *C. rubrolineata* and *C. exoptata* remains to be investigated. We are not excluding the possibility that *C. indica* is the same or a closely related species to the type species of *Coryphellina*, *C. rubrolineata*. We also tentatively assign *Nossis westralis* as a species of the genus *Coryphellina* but it needs to be further studied. The proposed family Nossidae Odhner, 1968 thus is a junior synonym of Flabellinidae *s. str.* Odhner (Odhner 1968) also proposed a few more genera within the family "Nossidae"; no descriptions were provided for these genera and they are *nomina nuda* according to article 13. 1 (ICZN 1999).

#### Coryphellina lotos sp. n.

Taxon classificationAnimaliaNudibranchiaFlabellinidae

http://zoobank.org/B89F0CA9-BBF3-4B3C-9946-B3696F194C6D

Fig. 38

##### Type material.

Holotype. ZMMU Op-515, 15.5 mm long (live), Japan, Pacific coast of Honshu, Osezaki, 10–15 m depth, 10.09.2016, coll. T.A. Korshunova, A.V. Martynov. 2 paratypes, ZMMU Op-516, up to 17 mm long (live), same locality and dates as holotype.

##### Type locality.

Osezaki, Pacific coast of Honshu, Japan.

##### Diagnosis.

Considerably reduced notal edge, forming several clusters, background colour light violet, digestive gland in cerata light brownish, subapical parts of cerata reddish lilac, apical parts of cerata without white pigment, rachidian tooth with up to seven distinct denticles, adpressed to rachidian cusp, lateral teeth with up to nine denticles, penis conical.

##### Etymology.

After the lotus flower in reference to the similarity of the colour of the new species to the common colour of the lotus flower.

##### Description.

*External morphology* (Fig. 38A–E). Body relatively narrow. Foot and tail narrow, anterior foot corners long. Rhinophores ca. 1.5 times shorter than oral tentacles, strongly papillate. Dorsal cerata finger-shaped to fusiform, forming several slightly elevated clusters along dorsal edges. Apices of cerata gradually pointed, with elongate cnidosac. Distinct notal edge remains mostly below cerata clusters. Digestive gland diverticulum fills significant volume of the cerata. Anal opening pleuroproctic on right side below second large ceratal clusters. Reproductive openings lateral, below first anterior cluster of cerata.

*Colour* (Fig. 38A). Background colour light violet. Digestive gland diverticula light brownish. Rhinophore background colour similar to body. Oral tentacles lilac (except for translucent tips). Subapical parts of cerata and rhinophores brighter reddish lilac. Apical parts of cerata without opaque cap of white pigment.

*Jaws* (Fig. 38G, H). Masticatory process more than one-third as long as jaw body. Edge of masticatory processes bears ca. 35 sharp denticles that continue to form at least two rows of denticles on body of masticatory processes.

*Radula* (Fig. 38I). Radula formula: 26 × 1.1.1 (31 mm). Rachidian tooth elongate-triangular with short narrow cusp of less than 1/3 of the tooth length (Fig. 38I). Rachidian tooth bears between four and seven well-defined separated long lateral denticles strongly adpressed towards cusp. Cusp is compressed by adjacent first lateral denticles. Lateral teeth (Fig. 38I) broadly triangular with obtuse and very attenuated posteriorly outer process, bear seven to nine sharp denticles.

*Reproductive system* (Fig. 38J). Diaulic. Hermaphroditic duct leads to convoluted ampulla of about two whorls. Vas deferens is relatively long, no distinct prostate. Penial sheath is large, wide. Penis is conical. Oviduct connects through insemination duct into female gland complex. Vagina short and indistinct. Receptaculum seminis large, oval, bilobed. Distal receptaculum seminis present, small.

##### Ecology.

Shallow water species, stony and rocky habitats.

##### Distribution.

Pacific side of middle Japan (Honshu).

##### Remarks.

The three specimens of *Coryphellina lotos* sp. n. demonstrate uniformity in the body shape and colour and are readily distinguished from any previously described *Coryphellina* species, including *C. rubrolineata*, which is sister to *C. lotos* sp. n. according to the molecular phylogenetic analysis (Fig. 1).

#### Edmundsella gen. n.

Taxon classificationAnimaliaNudibranchiaFlabellinidae

http://zoobank.org/180B02BB-5A88-4E8B-BF90-B79304FB6625

Figs 34 , 40

##### Type species.

*Doris pedata* Montagu, 1815.

##### Etymology.

In honour of distinguished opisthobranch taxonomist Malcolm Edmunds (UK) who died in January 2017.

##### Diagnosis.

Body narrow. Notal edge reduced. Cerata on low elevations, in several groups. Rhinophores wrinkled, similar in length to or shorter than oral tentacles. Anterior foot corners present. Anus pleuroproctic. Distinct oral glands. Rachidian teeth with narrow compressed cusp and distinct denticles. Lateral teeth denticulated with attenuated process basally. Separated distal and proximal receptaculum seminis. Short prostatic, non-granulated vas deferens. Penis conical to bulbous.

##### Species included.

*Edmundsella albomaculata* (Pola et al., 2014), comb. n. (original description in Pola et al. 2014), *Edmundsella pedata* (Montagu, 1815), comb. n. (Fig. 40) (original description in Montagu 1815), *Edmundsella* (?) *vansyoci* (Gosliner, 1994), comb. n. (original description in Gosliner 1994).

##### Remarks.

The genus *Edmundsella* gen .n. clearly differs morphologically from other flabellinid genera by the presence of smooth rhinophores, non-stalked cerata on low elevations, and a relatively short vas deferens. Molecular distances between *Edmundsella* gen. n. and other genera of the family Flabellinidae are significant (13%–19%; see Table 1).

#### Flabellina

Taxon classificationAnimaliaNudibranchiaFlabellinidae

Gray, 1833 in Griffith & Pidgeon, 1833–1834

Figs 2 , 34

Non Flabellina*sensu*Gosliner and Griffiths (1981) and auctt.
Non Flabellina d'Orbigny, 1839 (junior homonym, Foraminifera, replaced by Neoflabellina Bartenstein, 1948)

##### Type species.

*Dorisaffinis Gmelin*, 1791.

##### Diagnosis.

Body narrow. Notal ridge completely absent. Cerata on high compound stalks. Rhinophores with ring lamellae, shorter than oral tentacles. Anterior foot corners present. Anus pleuroproctic. Distinct oral glands. Rachidian teeth with narrow compressed cusp and distinct denticles. Lateral teeth denticulated with attenuated process basally. Two closely placed distal seminal receptacles. Long vas deferens without distinct prostate. Penis elongated conical.

##### Species included.

*Flabellina affinis* (Gmelin, 1791) (original description in Gmelin 1791; detailed redescription in Schulze and Wägele 1998).

##### Remarks.

After the restrictions imposed at the family level, the genus *Flabellina* (to which morphologically and molecularly extremely disparate taxa were formerly assigned) is thus returned to its original definition (Gray 1833 in Griffith and Pidgeon 1833–1834). It is characterised by very ramified tall ceratal stalks and a relatively long vas deferens. In our molecular analysis, the type species of the genus *Flabellina affinis* was placed basally and separate from the morphologically similar *F. funeka* and *F. ischitana*. Further complicating this case is the position of the genus *Calmella* which may be more closely related to *F. funeka* and *F. ischitana* rather than to *F. affinis*. Thus, to keep the genus *Flabellina* with the inclusion of *F. funeka* and *F. ishitana* is not fully consistent with the molecular data. Therefore, the clade that contains “*F.*” *funeka*, “*F.*” *ischitana* and “*Piseinotecus*” *gabienerei* must be separated in another new genus, *Paraflabellina* gen. n. (see below).

#### Paraflabellina gen. n.

Taxon classificationAnimaliaNudibranchiaFlabellinidae

http://zoobank.org/11399BB9-BF00-4A6E-B374-7193C2EA4C98

Figs 2 , 34

##### Type species.

*Flabellina ischitana* Hirano & Thompson, 1990.

##### Etymology.

From Ancient Greek *παρα* (= beside, adjacent to) and *Flabellina* in reference to the external similarity of the type species *F. ischitana* to *Flabellina* *s. str.*, but disparate molecular results that render the genus *Flabellina* *s. l.* paraphyletic in relation to the genus *Calmella* if *F. ischitana* is included within *Flabellina*.

##### Diagnosis.

Body narrow. Notal ridge completely absent. Cerata on high compound stalks. Rhinophores with ring lamellae, shorter than oral tentacles. Anterior foot corners present. Anus pleuroproctic. Rachidian teeth with narrow compressed cusp and distinct denticles. Lateral teeth denticulated with attenuated process basally. Proximal and distal receptaculum seminis. Long vas deferens without distinct prostate. Penis conical.

##### Species included.

*Paraflabellina funeka* (Gosliner & Griffiths, 1981), comb. n. (original description in Gosliner and Griffiths 1981), *Paraflabellina gabinierei* (Vicente, 1975), comb. n. (original description in Vicente 1975; additional data in Schmekel 1980), *Paraflabellina ischitana* (Hirano & Thompson, 1990) (original description in Hirano and Thompson 1990; redescription in Cervera et al. 1998), *Paraflabellina* (?) *rubromaxillarubromaxilla* (Edmunds, 2015), comb. n. (original description in Edmunds 2015).

#### Piseinotecus

Taxon classificationAnimaliaNudibranchiaFlabellinidae

Er. Marcus, 1955

Figs 2 , 34

##### Type species.

*Piseinotecus divae* Er. Marcus, 1955.

##### Diagnosis.

Body narrow. Notal ridge fully reduced. Cerata on low compound stalks. Rhinophores smooth, similar in size to oral tentacles. Anterior foot corners present. Anus pleuroproctic. Rachidian teeth with non-compressed cusp and distinct denticles. Lateral teeth absent. Proximal receptaculum seminis. Short vas deferens with distinct prostate. Penis conical.

##### Species included.

*Piseinotecus divae* Er. Marcus, 1955 (original description in Marcus 1955), *Piseinotecus* (?) *sphaeriferus* (Schmekel, 1965) (original description in Schmekel 1965).

##### Remarks.

The genus *Piseinotecus* is commonly considered to belong to a separate family Piseinotecidae Edmunds, 1970 because of the presence of a uniserial radula instead of the triserial radula common for traditional Flabellinidae. However, external features, including the presence of compound ceratal stalks in the type species of the genus *Piseinotecus*, *P. divae*, and the other species (e.g., *P. sphaeriferus*) suggest a relationship to *Flabellina*. Preliminary data suggest that this genus is deeply nested within the traditional family Flabellinidae (Gosliner et al. 2007; Tamsouri et al. 2014). This is confirmed by our analysis (Figs 1, 2) and therefore we have considered *Piseinotecus* a genus within the family Flabellinidae *s. str.*, after removal of the families Coryphellidae, Paracoryphellidae, and others. While this paper was under review an analysis was published (Furfaro et al. 2017) that at least some species previously considered within the genus *Piseinotecus* (i.e., “*P.*” *gabinierei* and “*P.*” *gaditanus*) actually possess a triserial radula and thus must be firmly included into the family Flabellinidae in its restricted sense. There are more undescribed species of the putative genus *Piseinotecus* that appear within some clades which are not closely related, e.g., Flabellinopsidae fam. n. (see above) (Fig. 2). Because of that and also since there are no molecular data for the type species *P. divae*, the validity and monophyly of this genus still needs to be confirmed. Therefore, until molecular data on the type species *P. divae* becomes available, we consider the genus *Piseinotecus* only tentatively as belonging to the family Flabellinidae *s. str.* According to the first description (Marcus 1955) *P. divae* has no distinct oral glands but has rather strong cusp of rachidian tooth. These features contradict to the diagnosis of the family Flabellinidae *s. str.* but still need to be confirmed by novel material. Since the Mediterranean “*P.*” *gabinierei* is closely related to *Paraflabellina ishitana* (Fig. 1) we include “*P.*” *gabinierei* into the genus *Paraflabellina* (see above). Another species “*P.*” *gaditanus* is nested within *Calmella* clade and therefore assigned to that genus. Whether the triserial radula has been overlooked in the type species *P. divae* or not is currently unclear. Therefore *Piseinotecus* is not synonymised in the present study with any existing genera, but awaits assignment until more data becomes available. In addition, based on the morphological data we do not include species without ceratal stalks in the genus *Piseinotecus* or species with more disparate ceratal and radular morphology, such as “*Piseinotecus*” *gonja* Edmunds, 1970 and “*Piseinotecus*” *minipapilla* Edmunds, 2015 (Edmunds 1970, 2015).

#### Family Flabellinidae incertae sedis

Taxon classificationAnimaliaNudibranchiaFlabellinidae

##### Species included.

*Flabellina alternata* Ortea & Espinosa, 1998 (original description in Ortea and Espinosa 1998), *Flabellina bertschi* Gosliner & Kuzirian, 1990 (original description in Gosliner and Kuzirian 1990), *Flabellina dana* Millen & Hamann, 2006 (original description in Millen and Hamann 2006), *Flabellina dushia* (Marcus & Marcus, 1963) (original description in Marcus and Marcus 1963, redescription in Millen and Hamann 2006), *Flabellina engeli* Marcus & Marcus, 1968 (original description in Marcus and Marcus 1968), *Flabellina engeli lucianae* DaCosta, Cunha, Simone, Schrödl, 2007 (original description in DaCosta et al. 2007), *Flabellina evelinae* Edmunds, 1989 (original description in Edmunds 1989), *Flabellina ilidioi* Calado, Ortea & Caballer, 2005 (original description in Calado et al. 2005), *Flabellina llerae* Ortea, 1989 (original description in Ortea 1989).

##### Remarks.

Some of these species are insufficiently described (e.g., *Flabellina alternata* Ortea & Espinosa, 1998, *Flabellina llerae* Ortea, 1989) whilst some others have been described in detail (e.g., *Flabellina engeli lucianae* DaCosta, Cunha, Simone & Schrödl, 2007), but in the absence of the molecular data in this case, we prefer not to assign these species into particular genera. Most likely, more new genera need to be created for some of these species.

#### Family Unidentiidae

Taxon classificationAnimaliaNudibranchiaUnidentiidae

Millen & Hermosillo, 2012

##### Diagnosis.

Body narrow. Notum fully reduced. Cerata in separate clusters, on distinct elongated elevations. Rhinophores smooth. Anus pleuroproctic or mixed (pleuroproctic in higher acleiproctic position). Distinct oral glands present. Radula formula 0.1.0. Rachidian teeth with non-compressed cusp. Lateral teeth always absent. Number and position of receptaculum seminis variable: two separate ones, or double or single proximal ones. Vas deferens moderately long, with distinct prostate. External permanent penial collar absent. Adjacent penial gland present or absent. Penis conical, armed or unarmed, always internal.

##### Genera included.

*Pacifia* gen. n., *Unidentia* Millen & Hermosillo, 2012.

##### Remarks.

This is a very interesting case which shows the polyphyletic nature of the traditional Flabellinidae. In 2010 a species with uniseriate radula and unusual supplementary penial gland was added to the traditional genus *Flabellina* (Gosliner 2010) despite the fact that the outlined characters directly contradict such a decision. The present molecular phylogenetic analysis fully confirms this species to be extremely disparate from the majority of Flabellinidae *s. str.* as suggested by its morphology. *Flabellina goddardi* and a new closely related species from the Pacific coast of Japan (see below) were clustered together in the same clade which was placed basally to the family Facelinidae and not clustering with any other Flabellinidae (Figs 1, 2). Importantly, this taxon is invariably placed basally to Facelinidae in any variants of the obtained trees and not nested within the family Flabellinidae *s. str.* as was suggested by Gosliner (2010: 630). Remarkably, two other new species of the genus *Unidentia* from Japan and from Indonesia also possess a uniserial radula and pleuroproctic anus in a higher acleioproctic position. This challenging taxon, with a new genus and two species, is confirmed for the first time in the present study as belonging to the family Unidentiidae Millen & Hermosillo, 2012.

Currently, many researchers are actively debating phylogenetic relationships and higher taxonomy of one of the most diverse nudibranch groups, Aeolidacea (e.g., Carmona et al. 2013; Padula et al. 2014; Kienberger et al. 2016; Korshunova et al. 2017a; Korshunova et al. 2017c). However, until recently no molecular analysis of aeolidacean nudibranchs has included the family Unidentiidae. When Gosliner (2010) described *F. goddardi* he only mentioned a preliminary molecular phylogeny, but no trees or molecular analyses have been presented since his publication. In addition, Gosliner (2010: 630) has highlighted that his data “strongly suggest that *F. goddardi*, despite having a uniseriate radula, is most closely related to other species of *Flabellina*”. However, the first molecular analysis of this group presented here has clearly and robustly shown that the family Unidentiidae (including the newly described *Pacifia amica* gen. n. et sp. n. and *Pacifia goddardi* comb. n.) is not related to any real *Flabellina*, but instead to the family Facelinidae. Since all members of the Facelinidae have a uniserial radula (i.e., as in Unidentiidae) in strong contrast to a triserial radula in traditional Flabellinidae, the phylogenetic relationship of Unidentiidae to Facelinidae (Fig. 1) uncovered in the present study is in much greater agreement with the morphological data on radula, thus confirming the general integrative agenda for taxonomy (Dayrat 2005; Korshunova et al. 2017b).

The morphological cladistic analysis (Millen and Hermosillo 2012) also placed the uniserial family Unidentiidae as a basal group to the triserial Flabellinidae, but included including some uniserial groups like *Piseinotecus* and *Babakina*. However, most recently (Furfaro et al. 2017) it was shown, in remarkable agreement with our molecular phylogenetic study, that some species placed in *Piseinotecus* actually possess a triserial radula and are closely related to the true Flabellinidae, and that delicate lateral teeth were previously simply unrecognised. Since on the morphological cladogram the genus *Piseinotecus* was clustered basally to the also uniserial genus *Babakina* (the family Babakinidae) it may thus not reflect the real phylogenetic placement. In our phylogenetic analysis, the genus *Babakina* is not related to any Flabellinidae or Unidentiidae, but instead placed basally to the family Aeolidiidae (Fig. 1).

Thus, this first molecular data and molecular analysis of the family Unidentiidae clearly shows that previous morphological estimations of the *F. goddardi* as a proper flabellinid (Gosliner 2010) (despite the uniserial radula) or a group closely related to the traditional family Flabellinidae (Millen and Hermosillo 2012) were incorrect. However, Millen and Hermosillo (2012) prophetically separated *Unidentia* into an independent family that is now fully confirmed by our molecular analysis. Our new data also confirm the reliability of the detailed morphological analysis on an integrative basis (Korshunova et al. 2017a) since striking differences of the uniserial *F. goddardi* and *Unidentia* with the triserial traditional Flabellinidae, previously considered as just an independent case of reduction of lateral teeth, (e.g., according to the cladogram in Millen and Hermosillo 2012) is actually an indication of phylogenetic relationship with the uniserial traditional Facelinidae. The retaining of a pleuroproctic anus, or pleuroproctic in a higher acleioproctic position in Unidentiidae, can be considered therefore as a plesiomorphic feature (see Korshunova et al. 2017c for a discussion of the plesiomorphic pleuroproctic position in Nudibranchia).

The present new findings and the first molecular phylogenetic analysis involving members of the family Unidentiidae thus not only has particular taxonomic importance but also makes important contributions for the understanding of the phylogeny of the whole Nudibranchia group and for the general discussion about the plausibility of using morphological data for phylogenetic analysis and high-level systematics.

In the combined phylogenetic tree, all Unidentiidae species clustered in a highly supported clade together (PP = 1, BS = 98) that is dramatically separated from the Flabellinidae clade. All *Unidentia* species clustered together (PP = 1, BS = 100) in a maximum-supported clade that is sister to the maximum-supported (PP = 1, BS = 100) clade with the species of *Pacifia* gen. n.

The family Unidentiidae originally was incorrectly spelled as Unidentiidae (Millen and Hermosillo 2012). The name is based on *Unidentia* with the stem therefore being Unidenti-, so the original name Unidentiidae should be corrected to Unidentiidae with the same author and date (ICZN 1999, article 29.3).

#### Pacifia gen. n.

Taxon classificationAnimaliaNudibranchiaUnidentiidae

http://zoobank.org/CFB27003-57DF-4AE5-A6D4-268AC434DDF7

##### Type species.

*Pacifia amica* sp. n.

##### Etymology.

After the Pacific Ocean and also in reference to another meaning of the word “pacific” = “peaceful” and “amica” = “friend”, “friendly” because this paper represents a friendship-based collaboration between American, British, German, Japanese, Indonesian, Norwegian, Russian, and Swedish colleagues, as opposed to recent antagonistic political relationships.

##### Diagnosis.

Body narrow. Notum fully reduced. Cerata on elevations. Rhinophores smooth. Anterior foot corners present. Anus pleuroproctic. Rachidian teeth with narrow non-compressed cusp covers with small denticles; lateral denticles distinct. Lateral teeth absent. Proximal and distal receptaculum seminis. Moderately long vas deferens with thickened non-granulated prostate. No external penial collar. Penis conical, unarmed. Adjacent penial gland may be present.

##### Species included.

*Pacifia amica* sp. n., *P. goddardi* (Gosliner, 2010), comb. n.

##### Remarks.

From *Unidentia*, the only currently known genus of the family Unidentiidae, *Pacifia* gen. n. is readily distinguished by the presence of a distal receptaculum seminis, absence of the penial stylet and considerable molecular distance. Despite differing in some morphological characteristics from *P. amica* sp. n. (including the presence in *F. goddardi* Gosliner, 2010 of distinct ceratal elevations and an accessory gland near the penis, and also by the absence of distinct denticles on the cusp of the rachidian teeth throughout the whole radula) *Flabellina goddardi* Gosliner, 2010, forms a sister molecular clade to *Pacifia amica* sp. n. and also shares with the latter the presence of a distal stalked receptaculum seminis. We therefore include *F. goddardi* in the new genus *Pacifia* as a new combination *Pacifia goddardi* (Gosliner, 2010), comb. n. Presence of an accessory gland near the penis in *P. goddardi* needs in further confirmation.

#### Pacifia amica sp. n.

Taxon classificationAnimaliaNudibranchiaUnidentiidae

http://zoobank.org/FA6D85E0-022E-4807-A93F-3BD4F790A5BD

Figs 41 , 45

##### Type material.

Holotype, ZMMU Op-614, 6.5 mm long (live), NE Pacific, Sullivan Point, Rich Passage, 25.01.2017, depth 15.2 m, stones, collector Karin Fletcher. 1 paratype, ZMMU Op-615, same locality and collector as holotype.

##### Type locality.

NE Pacific, Rich Passage.

##### Etymology.

See under the genus description above.

##### Diagnosis.

Four to five ceratal clusters, colour translucent white with opaque white mid-dorsal line, cerata orange red to light pink, rachidian tooth with up to six denticles, central cusp bears small denticles, distinct distal receptaculum seminis, no accessory penial gland, penis unarmed.

##### Description.

*External morphology* (Fig. 41A–B). Body elongate, 6.5 mm in length (live). Rhinophores similar in size to oral tentacles, smooth. Dorsal cerata finger-shaped to fusiform, forming four to five ceratal clusters along dorsal edges. Apices of cerata gradually pointed, with elongate cnidosac. Notal edge discontinuous. Digestive gland diverticulum fills significant volume of the cerata. Anal opening pleuroproctic towards acleioproctic position on right side below second large ceratal clusters. Reproductive openings lateral, below first anterior cluster of cerata.

*Colour* (Fig. 41A–B). General colour translucent white. Oral tentacles with opaque white lines, which meet on middle of head and continue through middle part of body to the tail. Digestive gland in the cerata orange-red to light pink; scattered small spots on cerata, ceratal tip transparent (Fig. 41C).

*Jaws* (Fig. 41D–E). Jaws broadly-triangular, masticatory borders with weak tubercles.

*Radula* (Fig. 41F–H) Radula formula 28 × 0.1.0. Rachidian tooth with up to six distinct denticles. Rachidian cusp bears small denticles at the base (Fig. 41H). No lateral teeth.

*Reproductive system* (Fig. 41I). Diaulic. Ampulla wide, folded twice (Fig. 41I, am). Prostate thick (Fig. 41I, pr). Vas deferens short (Fig. 41I, vd), penial sheath elongate (Fig. 41I, ps), conical penis unarmed. Distal seminal receptaculum long, with distinct stalk and oval reservoir (Fig. 41I, rsd). Proximal receptaculum seminis oval.

##### Biology.

Subtidal, on stones with hydroids (Fig. 41B).

##### Distribution.

Presently found only at a Port Orchard locality in Washington State, NE Pacific.

##### Remarks.

See Remarks under the new genus *Pacifia* above.

#### Unidentia

Taxon classificationAnimaliaNudibranchiaUnidentiidae

Millen & Hermosillo, 2012

Figs 42 , 43

##### Type species.

*Unidentia angelvaldesi* Millen & Hermosillo, 2012.

##### Diagnosis.

Body narrow. Notum fully reduced. Cerata on distinct elonagate elevations. Rhinophores smooth. Anterior foot corners present. Anus mixed (pleuroproctic in higher cleiproctic position) or pleuroproctic. Rachidian teeth with narrow non-compressed cusp and distinct denticles. Lateral teeth absent. Proximal double or single receptaculum seminis. Distal receptaculum seminis absent. Moderately long vas deferens with widened prostatic part. Penis conical, with hollow stylet. Adjacent penial absent.

##### Species included.

*Unidentia angelvaldesi* Millen & Hermosillo, 2012 (first description in Millen and Hermosillo 2012), *U. nihonrossija* sp. n. (Fig. 42), *U. sandramillenae* sp. n. (Fig. 43).

#### Unidentia nihonrossija sp. n.

Taxon classificationAnimaliaNudibranchiaUnidentiidae

http://zoobank.org/08E34FC9-6AAF-4F4D-A1DB-3CDCC4B77BBD

Fig. 42

##### Type material.

Holotype. ZMMU Op-517, 4.5 mm long (preserved), Japan, Osezaki, depth 10–15 m, 11.09.2016, coll. T.A. Korshunova, A.V. Martynov.

##### Type locality.

Osezaki, Japan.

##### Etymology.

After *nihon* (“日本” (=“にほん”), meaning “Japan” in Japanese) and *rossija* (“Россия” meaning “Russia” in Russian) in reference to the international group of Russian and Japanese scientists who organised the field work in Japan, during which this unique representative of the family Unidentiidae was collected.

##### Diagnosis.

Five to seven ceratal clusters, background colour whitish with dispersed light violet middorsal line, cerata orange to reddish orange, subapical parts of cerata with broad opaque white line, apical parts of cerata with white pigment, rachidian tooth with up to six denticles, central cusp bears small denticles, distinct distal receptaculum seminis, no accessory penial gland, penis with hollow stylet.

##### Description.

*External morphology* (Fig. 42A–E). Body relatively narrow. Foot and tail narrow, anterior foot corners long. Rhinophores ca. 1.5–2 times shorter than oral tentacles, smooth. Dorsal cerata finger-shaped to fusiform, forming several distinctly elongate elevations with ceratal clusters along dorsal edges. Apices of cerata gradually pointed, with elongate cnidosac. Distinct notal edge absent. Digestive gland diverticulum fills significant volume of the cerata. Anal opening pleuroproctic on right side below second large ceratal clusters. Reproductive openings lateral, below first anterior cluster of cerata.

*Colour* (Fig. 42A). Background colour whitish. Digestive gland diverticula reddish orange. Rhinophores and oral tentacles light violet. A narrow dispersed light violet band runs from base of oral tentacles through middle part of dorsal side. Subapical parts of cerata with broad opaque white line. Apical parts of cerata without opaque cap of white pigment.

*Jaws* (Fig. 42G, H). Masticatory process more than one-third as long as jaw body. Edge of masticatory processes bears several rows of ridge-shaped denticles.

*Radula* (Fig. 42I). Radula formula: 39 × 0.1.0. Rachidian tooth elongate-triangular with short broad cusp. Rachidian tooth bears four to seven well-defined separated long lateral denticles strongly adpressed towards cusp (Fig. 42I). Cusp is not compressed by adjacent first lateral denticles. Lateral teeth absent.

*Reproductive system* (Fig. 42J). Diaulic. Hermaphroditic duct leads to convoluted ampulla of about two whorls. Vas deferens relatively long, no distinct prostate. Penial sheath narrow. Penis narrow conical, armed with curved hollow stylet. Oviduct connects through insemination duct into female gland complex. Vagina short and indistinct. Double proximal receptaculum seminis. Supplementary (“penial”) gland absent.

##### Ecology.

Shallow waters, stony habitats.

##### Distribution.

Pacific side of middle Japan (Honshu).

##### Remarks.

From the type species of the genus *Unidentia*, the Eastern Pacific Mexican species *U. angelvaldesi* Millen & Hermosillo, 2012, *U. nihonrossija* sp. n. differs substantially both morphologically and by colouration, details of the radular teeth, and presence of double proximal receptacles (*U. angelvaldesi* possesses a single receptaculum seminis). From the tropical Indo-West Pacific species *U. sandramillenae* sp. n. (see below), *U. nihonrossija* sp. n. differs both morphologically and according to the molecular phylogenetic analysis (Figs 1, 2).

#### Unidentia sandramillenae sp. n.

Taxon classificationAnimaliaNudibranchiaUnidentiidae

http://zoobank.org/88733F74-3948-47E5-8C25-EBFFDDACEC12

Fig. 43

##### Type material.

Holotype, ZMMU Op-617, 7 mm long (preserved), Bali, Tulamben, 02.02.2017, 5–10 m depth, stones, collector I Wayan Mudianta.

##### Type locality.

Bali Island, Indonesia.

##### Etymology.

In honour of prominent nudibranch researcher, Sandra Millen (The University of British Columbia), who for almost 50 years has conducted fine taxonomic studies, including separation of the new family, Unidentiidae, together with Alicia Hermosillo.

##### Diagnosis.

Eight to ten ceratal clusters, ground colour and cerata whitish with violet hue, purple middorsal line, rachidian tooth with up to eight denticles, central cusp bears small denticles only in anterior radula, two proximal seminal receptacles, no accessory penial gland, penis with hollow stylet.

##### Description.

*External morphology* (Fig. 43A–C). Body elongate 7 mm in length (preserved). Rhinophores similar in size to oral tentacles, smooth. Cerata finger-shaped to fusiform, placed on several distinctly elongate elevations with ceratal clusters along dorsal edges. Apices of cerata gradually pointed, with elongate cnidosac. Distinct notal edge absent. Digestive gland diverticulum fills significant volume of the cerata. Anal opening pleuroproctic towards acleioproctic position on right side below second large ceratal clusters. Reproductive openings lateral, below first anterior cluster of cerata.

*Colour* (Fig. 43A–D). General colour translucent white with purple thin middorsal line; oral tentacles covered in purple pigment nearly half its length; subapical purple rings on cerata.

*Jaws* (Fig. 43E). Jaws broadly triangular, masticatory borders with special elaborate long denticles placed in clusters.

*Radula* (Fig. 43H, I). Radula formula 25 × 0.1.0. Rachidian tooth with up to eight distinct denticles. Central cusp smooth, widened in the middle and distinctly narrowed towards the tip; no small denticles at the base in posterior and middle teeth; vestigial denticles may present on the cusp of a few anterior teeth (Fig. 43I). No lateral teeth.

*Reproductive system* (Fig. 43K). Diaulic. Ampulla very voluminous, folded twice (Fig. 43K, am). Prostate thick (Fig. 43K, pr). Vas deferens short (Fig. 43K, vd), penial sheath elongate, conical penis armed with hollow stylet (Fig. 43K). Double proximal receptaculum seminis (Fig. 43K, rsp).

##### Biology.

On stones and algae covered with athecate hydroids (Fig. 43C, D).

##### Distribution.

Currently Indonesia, but probably Indo-West Pacific.

##### Remarks.

*Unidentia sandramillenae* sp. n. differs considerably from the only other known species of the genus *Unidentia*, *U. angelvaldesi* Millen & Hermosillo, 2012, both externally and internally. Externally, *Unidentia sandramillenae* sp. n. has a whitish ground colour whereas the Mexican specimens of *U. angelvaldesi* often have a red-orange ground colour. Internally, *Unidentia sandramillenae* sp. n. differs from *U. angelvaldesi* in the shape of the central cusp of the rachidian teeth, the shape of the ampulla and the presence of a distinct second seminal reservoir. Furthermore, in the original description of *U. angelvaldesi* several distinct species from various regions of the Pacific were commingled. Because the holotype was originally from Mexico in the eastern Pacific, the species *U. angelvaldesi* should be restricted to specimens from the tropical eastern Pacific. There are no molecular data available for the Mexican *U. angelvaldesi*; however, according to our new data, a separate species of *Unidentia*, which clearly differs from the *Unidentia sandramillenae* sp. n. from Bali, is also present in Japan and described here as *U. nihonrossija* sp. n. (Figs 42, 44, red lines). Therefore, taking into consideration the considerable morphological differences between *U. angelvaldesi* and *Unidentia sandramillenae* sp. n. these species should also have a considerable molecular gap (Fig. 44, blue line), that should be proved in further studies. In addition, the clade *U. angelvaldesi* is distant from Flabellinopsidae fam. n. according to a recent publication (Goodheart et al. 2017).

Uncorrected p-distances of the mitochondrial barcode marker (COI) between *U. sandramillenae* sp. n. and *U. nihonrossija* sp. n. reach 14.2%. Uncorrected p-distances between *P. amica* sp. n. and *P. goddardi* reach 12.2%. In contrast, the distance values between *Unidentia* and *Pacifia* ranged between 18.3–20.7% supporting the decision to create the new genus *Pacifia*.

#### Family Samlidae fam. n.

Taxon classificationAnimaliaNudibranchiaSamlidae

http://zoobank.org/6366D651-3069-49A2-8B2B-140894687F1A

##### Diagnosis.

Body narrow. Notum discontinuous. Cerata in separate clusters, on elevations. Rhinophores perfoliated. Anus pleuroproctic under the reduced notal edge or mixed (pleuroproctic in higher acleioproctic position). Distinct oral glands present, commonly penetrate below anterior cerata. Radula formula 1.1.1. Rachidian teeth usually with strong cusp, only rarely compressed by adjacent lateral denticles. Lateral teeth denticulated narrow or with attenuated process basally. Distal and proximal receptaculum seminis or only proximal receptaculum. Vas deferens usually short, with indistinct prostate. External permanent penial collar absent. Penis in many cases conical, narrow, always internal and unarmed.

##### Genera included.

*Luisella* gen. n., *Samla* Bergh, 1900.

##### Remarks.

The genus *Samla* and related taxa were invariably considered as synonyms of the traditional genus *Flabellina* throughout all the recent history of nudibranch taxonomy (e.g., Gosliner and Griffiths 1981; McDonald 2009). Previously it was listed sometimes as valid (e.g., Burn 1964) but without detailed discussion. However, the Indo-Pacific tropical genus *Samla* differs morphologically from the Flabellinidae *s. str.* in the absence of compound stalks, often non-compressed cusp of the rachidian teeth, and also by having a short vas deferens. The present molecular phylogenetic analysis not only confirmed such disparate morphology from true Flabellinidae, but also revealed a very profound problem: species of the genus *Samla* and some related taxa are basal to the Aeolidacea as a whole and the major complex of aeolidacean families, including Aeolidiidae, Facelinidae, Tergipedidae, and Eubranchidae, rendering the Flabellinidae polyphyletic! Such topology with minor variations has appeared invariably on all trees obtained in the present analysis and was hinted at as soon as *Flabellina babai* was sequenced (Carmona et al. 2011). This result is of great importance because it clearly implies that 1) Understanding of the real phylogenetic and taxonomic patterns of the traditional “Flabellinidae” is possible only with inclusion of all major families of the nudibranch suborder Aeolidacea in the analysis, as is performed in the present study, and 2) The traditional “Flabellinidae” cannot be by any means maintained as single family, but must be separated into several smaller morphologically and molecularly consistent families (Fig. 2). Therefore, the genus *Samla* Bergh, 1900 (Bergh 1900b, 1908) is resurrected and the new family Samlidae is established here.

Apart from the taxa that belong to the genus *Samla*, which are clustered in a compact clade (Figs 1, 2, 45) the species *Flabellina babai* Schmekel, 1972 appears as a closely related clade. *Flabellina babai* differs considerably from *Samla* including the character of a distinct granulated prostate (Schmekel 1972; Schmekel and Portmann 1982), unique for the majority of the Paracoryphellidae, Coryphellidae and Flabellinidae. This feature is so far known only for the paracoryphellid genus *Chlamylla*, which is dramatically different from *Flabellina babai* externally. Therefore, a new genus for *F. babai*, *Luisella* gen. n., is proposed here (see below).

#### Luisella gen. n.

Taxon classificationAnimaliaNudibranchiaSamlidae

http://zoobank.org/16513FA9-F61E-4626-A46B-04970B4A6B73

Figs 2 , 45

##### Type species.

*Flabellina babai* Schmekel, 1972.

##### Etymology.

Named to honour an eminent expert of nudibranchs, Luise Schmekel, who named *Flabellina babai* and authored the well-recognised monograph *Opisthobranchia des Mittelmeeres.*

##### Diagnosis.

Body narrow. Notal ridge completely absent. Cerata in several distinct rows. Rhinophores perfoliated, shorter than oral tentacles. Anterior foot corners present. Anus mixed: pleuroproctic shifted towards dorsal acleioproctic position. Rachidian teeth with narrow compressed cusp and distinct denticles. Lateral teeth denticulated with attenuated process basally. Proximal tubular receptaculum seminis. Moderately long vas deferens widened distally, prostate distinct, granulated. No external penial collar. Penis bluntly conical.

##### Species included.

*Luisella babai* (Schmekel, 1972), comb. n. (original description in Schmekel 1972).

#### Samla

Taxon classificationAnimaliaNudibranchiaSamlidae

Bergh, 1900

Figs 45 , 46 , 47

##### Type species.

*Samla annuligera* Bergh, 1900

##### Diagnosis.

Body narrow. Notal ridge present, discontinuous. Cerata on low elevations. Single precardiac ceratal cluster. Rhinophores perfoliated, shorter than oral tentacles. Anterior foot corners present. Anus pleuroproctic. Rachidian teeth usually with relatively broad non-compressed cusp and distinct denticles, more rarely cusp compressed. Lateral teeth with attenuated process basally. Distal and proximal receptaculum seminis. Short vas deferens distally widens into non-granulated prostate. No external penial collar. Penis indistinct or conical.

##### Species included.

*Samla bicolor* (Kelaart, 1858) (= *Samla annuligera* Bergh, 1900) (Fig. 46) (original description in Kelaart 1858; Bergh 1900b; redescription in Gosliner and Willan 1991), *Samla bilas* (Gosliner & Willan, 1991), comb. n. (original description in Gosliner and Willan 1991), *Samla macassarana* (Bergh, 1905), comb. n. (original description in Bergh 1905), *Samla riwo* (Gosliner & Willan 1991), comb. n. (original description in Gosliner and Willan 1991), *Samla takashigei* sp. n. (Fig. 47). Two more species are tentatively assigned to the genus *Samla*, but their placement needs to be refined using molecular data: *Samla telja* (Marcus & Marcus, 1967), comb. n. (original description in Marcus and Marcus 1967) and *Samla rubropurpurata* (Gosliner & Willan, 1991), comb. n. (original description in Gosliner and Willan 1991).

#### Samla takashigei sp. n.

Taxon classificationAnimaliaNudibranchiaSamlidae

http://zoobank.org/A2B247EA-1BD3-4D38-9648-6F74AF5DE534

Fig. 47

##### Type material.

Holotype, ZMMU Op-530, 26 mm long (live), Japan, Osezaki, 11.09.2016, depth 10–15 m, coll. T.A. Korshunova, A.V. Martynov.

##### Type locality.

Osezaki, Japan.

##### Etymology.

Named in honour of Hiroshi Takashige (Tokyo), esteemed diver who is very fond of natural history and provided invaluable help during field collection in Osezaki, Japan.

##### Diagnosis.

Considerably reduced notal edge, forming several slightly elevated rows, background colour whitish with bluish hue, digestive gland in cerata light brownish, subapical parts of cerata orange-brownish, apical parts of cerata without white pigment, rachidian tooth with up to nine distinct denticles, slightly adpressed to central cusp, lateral teeth with up to eight denticles, penis conical.

##### Description.

*External morphology* (Fig. 47A–E). Body narrow. Foot and tail narrow, anterior foot corners long. Rhinophores ca. six times shorter than extremely long oral tentacles, annulated. Dorsal cerata finger-shaped to fusiform, forming eight slightly elevated rows along dorsal edges. Apices of cerata gradually pointed, with elongate cnidosac. Distinct notal edge remains mostly below cerata clusters. Digestive gland diverticulum fills significant volume of the cerata. Anal opening pleuroproctic on right side between first and second ceratal rows. Reproductive openings lateral, below first anterior cluster of cerata.

*Colour* (Fig. 47A). Background colour whitish with bluish hue. Digestive gland diverticula light brownish. Rhinophores background colour similar to body. Subapical parts of cerata brighter orange–brownish. Cerata covered with opaque white pigment throughout most of the length. Apical parts of cerata without opaque cap of white pigment.

*Jaws* (Fig. 47F, G). Masticatory process more than one-third as long as jaw body. Edge of masticatory processes bears approximately 31 sharp denticles that form main single row of denticles on body of masticatory processes.

*Radula* (Fig. 47H). Radula formula: 19 × 1.1.1 (26 mm). Rachidian tooth elongate-triangular with short narrow cusp of less than 1/3 of tooth length (Fig. 47H). Rachidian tooth bears seven to nine well-defined separated long lateral denticles slightly adpressed towards cusp. Cusp is compressed by adjacent first lateral denticles. Lateral teeth (Fig. 47H) broadly triangular with obtuse and very attenuated posteriorly outer process, bear between six and eight sharp denticles. Some denticles can be forked or even triple.

*Reproductive system* (Fig. 47I, J). Diaulic. Hermaphroditic duct leads to convoluted ampulla of about two whorls. Vas deferens long, S-shaped, no distinct prostate. Penial sheath is narrow. Penis is narrow conical. Oviduct connects through insemination duct into female gland complex. Vagina short and indistinct. Proximal receptaculum seminis oval, swollen. Distal receptaculum seminis present, small.

##### Ecology.

Shallow waters, stony habitats.

##### Distribution.

Pacific side of middle Japan (Honshu).

##### Remarks.

From all known species of the genus *Samla*, *S. takashigei* sp. n. differs by colour pattern and details of the radular teeth and these morphological differences are robustly supported by molecular data (Fig. 2). The three specimens examined demonstrate significant uniformity in the body shape and colour. Our specimens from Vietnam (Fig. 46) are consistent morphologically with the type species of the genus *Samla*, *S. bicolor* (Kelaart, 1858) and its recognised synonym *S. annuligera* Bergh, 1900, according to the original descriptions (Kelaart 1858; Bergh 1900b) and differ considerably from the Japanese *S. takashigei* sp. n. However, more species in the *S. bicolor* species complex are clearly present in the Indo-West Pacific according to the present phylogenetic analysis (Fig. 1); these will need attention in a further study.

Uncorrected p-distances of the mitochondrial barcode marker (COI) between *Luisella babai* and *Samla* species range between 19.0–21.0%, supporting the genus *Luisella* as a separate genus from *Samla* within the family Samlidae.

#### Family Apataidae fam. n.

Taxon classificationAnimaliaNudibranchiaApataidae

http://zoobank.org/A0C932C0-CA54-419B-98EB-24BADFF66741

##### Diagnosis.

Body narrow. Notum fully reduced. Cerata in separate rows, on elevations. Rhinophores perfoliated. Anus mixed, pleuroproctic in higher acleioproctic position. Distinct oral glands present. Radula formula 1.1.1. Rachidian teeth cusp compressed by adjacent lateral denticles. Lateral teeth smooth with attenuated process basally. Distal receptaculum seminis. Vas deferens moderately long, with indistinct prostate. External permanent penial collar absent. Penis conical, narrow, always internal unarmed.

##### Genera included.

*Apata* gen. n., ?*Tularia* Burn, 1966.

##### Remarks.

Another unexpected and novel result is the apparent phylogenetic relationship between Samlidae and a North Pacific species, *Coryphella pricei* MacFarland, 1966. In our analysis *C. pricei* appears either as sister to the Samlidae *s. str.* (i.e. *Samla* + *F. babai*) or as a separate clade basal to Samlidae, Eubranchidae, and Tergipedidae (Figs 1, 2). Morphologically *C. pricei* is also quite separate from any Flabellinidae *s. l.* since it possesses peculiar comb-shaped ceratal rows in combination with a relatively long and thick vas deferens and smooth lateral teeth. Thus, *C. pricei* deserves not only a new genus but also a family-level taxon. Despite its somewhat unstable position, a new genus and family are established for *C. pricei* in order to highlight obvious morphological and molecular differences from the Samlidae (*Apata* gen. n. and Apataidae, respectively).

*Tularia bractea* Burn, 1966 was described from southern Australia (Burn 1966) and possesses simple raised rows of cerata instead of clusters or stalks, a triserial radula with smooth lateral teeth, and a reproductive system without a supplementary gland at the penis and single distal receptaculum seminis. These characters are in agreement with the diagnosis of the family Apataidae, and we therefore included *Tularia* in this family until molecular data are available.

#### Apata gen. n.

Taxon classificationAnimaliaNudibranchiaApataidae

http://zoobank.org/839B3636-1C79-41E8-9ED4-B7362847781A

Figs 45 , 48

##### Type species.

*Coryphella pricei* MacFarland, 1966

##### Etymology.

From the Russian form "apata" of the Ancient Greek *Απατη*, a deity of deceit, in reference to some deceptive features of the new genus and family, which are highly similar (especially the radular teeth) to the family Flabellinidae, but some other characters (e.g., comb-like instead of pedunculate ceratal rows) and molecular phylogenetic data place the new taxon in a very separate position from true flabellinids, making the traditional family Flabellinidae remarkably polyphyletic.

##### Diagnosis.

Body narrow. Notal ridge completely absent. Cerata in several distinct rows. Rhinophores perfoliated, shorter than oral tentacles. Anterior foot corners present. Anus mixed: pleuroproctic shifted towards a dorsal acleioproctic position. Rachidian teeth with narrow compressed cusp and distinct denticles. Lateral teeth with attenuated process basally. Moderately long vas deferens widened distally, prostate indistinct. No external penial collar. Penis bluntly conical.

##### Species included.

*Apata pricei pricei* (MacFarland, 1966), comb. n. (original description in MacFarland 1966), *Apata pricei komandorica* subsp. n.

#### Apata pricei komandorica subsp. n.

Taxon classificationAnimaliaNudibranchiaApataidae

http://zoobank.org/81627421-C76B-4BBC-8081-86E67B0973F4

Fig. 48

##### Type material.

Holotype, ZMMU Op-533, 8 mm long (fixed), North West Pacific, Commander Islands, 13.08.2014, 55°12.2182'N 165°55.4992'E, depth 19.5 m, coll. N.P. Sanamyan. Two paratypes, ZMMU Op-599, 7 mm long, same locality and date as holotype.

##### Type locality.

North West Pacific, Commander Islands.

##### Etymology.

After the Commander Islands in the NW Pacific (after original Russian spelling Komandorskie Islands), type locality of the subspecies.

##### Diagnosis.

Completely reduced notal edge, comb-like ceratal rows, background colour whitish, digestive gland in cerata greenish at the base and brownish-reddish toward ceratal top, subapical parts of cerata with white pigment, apical parts of cerata without white pigment, rachidian tooth with up to seven distinct denticles, adpressed to central cusp, lateral teeth smooth, penis conical.

##### Description.

*External morphology* (Fig. 48A–D). Body narrow. Foot and tail narrow, anterior foot corners short. Rhinophores similar in length to oral tentacles, bear up to 15 annulations. Dorsal cerata finger-shaped to fusiform, forming up to ten comb-shaped rows on dorsal side. Apices of cerata gradually pointed, with elongate cnidosac. Distinct notal edge remains mostly below cerata clusters. Digestive gland diverticulum fills significant volume of the cerata. Anal opening almost acleioproctic (“pleuroproctic in higher acleioproctic position”) on right side between six and seven ceratal rows. Reproductive openings lateral, below first anterior cluster of cerata.

*Colour* (Fig. 48A, C). Background colour whitish and translucent. Digestive gland diverticula greenish at base and first half and light brownish-dark reddish toward top of cerata. Rhinophores oral tentacles, and subapical parts of cerata covered dorsally with white pigment. Apical parts of cerata translucent.

*Jaws* (Fig. 48E, F). Masticatory process more than one-third as long as jaw body. Edge of masticatory processes bears ca. 30 broad flattened denticles that form a main single row of denticles on the body of the masticatory processes.

*Radula* (Fig. 48G). Radula formula: 20 × 1.1.1 (8 mm). Rachidian tooth elongate-triangular with short narrow cusp of less than 1/3 of tooth length (Fig. 48G). Rachidian tooth bears between five and seven well-defined separated long lateral denticles considerably adpressed towards cusp. Cusp considerably adpressed by adjacent first lateral denticles. Lateral teeth (Fig. 48G) completely smooth, form long narrow curved plate with innermost pointed strong denticle.

*Reproductive system* (Fig. 48H). Diaulic. Hermaphroditic duct leads to compact ampulla. Vas deferens is long, looped, no distinct prostate, but distal part considerably enlarged. Penial sheath narrow. Penis narrow conical. Oviduct connects through insemination duct into female gland complex. Vagina short and indistinct. Proximal receptaculum seminis oval, swollen. Distal receptacle present, rounded, on a very long convoluted duct, which is expanded distally.

##### Ecology.

Intertidal to 20–30 m, stony and soft bottom.

##### Distribution.

NW Pacific, Commander Islands, Middle Kurile Islands.

##### Remarks.

The type locality of *Apata pricei* (MacFarland, 1966) is California. According to the present phylogenetic analysis at least two species can be clearly separated among “*Apata pricei*” in Californian waters (Fig. 1): both are molecularly distinct from our material from Commander Islands using the COI molecular marker, although to different degrees. One species is highly divergent at 12.98%, and the other one is closer but still different (1.83%) from the Commander Islands specimens. The Commander Islands specimens are therefore recognised as a separate subspecies *Apata pricei komandorica* subsp. n. Morphological differences of the new subspecies are the fewer number of ceratal rows (up to ten) compared to the original description of *A. pricei pricei* from California by MacFarland (1966) (up to at least 12 ceratal rows). We cannot definitively evaluate the taxonomic placement of A. cf. pricei (CAS 114776) according to the original description of MacFarland (1966) due to the lack of the morphlogical description for this specimen in Cella et al. (2016). Further study on the *Apata pricei* complex is necessary to clarify its species composition in the Eastern Pacific.

### Family *incertae sedis*

Species included (previously were included in Flabellinidae *sensu lato*). *Coryphella californica* Bergh, 1904 (original description in Bergh 1904), *Coryphella* (?) *pallida* A. E. Verrill, 1900 (original description in Verrill 1900), *Flabellina bulbosa* Ortea and Espinosa, 1998 (original description in Ortea and Espinosa 1998).

#### Kynaria gen. n.

Taxon classificationAnimaliaNudibranchiaApataidae

http://zoobank.org/EF57B240-60C9-4E97-BC92-48E42AEA3399

##### Type species.

*Coryphella cynara* Marcus & Marcus, 1967

##### Etymology.

From the Greek word *κινάρα* , which means “artichoke”, in reference to the Latinised name of the type species “*C.*” *cynara* which was originally named after the peculiar shape of its body which appeared when the animal folded its cerata, so the general outline of the body became similar to an artichoke.

##### Diagnosis.

Body narrow. Notum discontinuous. Cerata on low elevations, in several groups. Rhinophores perfoliated, shorter than oral tentacles. Anterior foot corners present. Anus mixed: pleuroproctic shifted towards dorsal acleioproctic position. Rachidian teeth with narrow non-compressed cusp and distinct denticles. Lateral teeth with attenuated process basally. Moderately long vas deferens with indistinct prostate. No external penial collar. Penis large, globular, tuberculated.

##### Species included.

*Kynaria cynara* (Marcus & Marcus, 1967), comb. n. (original description in Marcus and Marcus 1967).

##### Remarks.

*Kynaria cynara* possesses a very special large globular copulative apparatus which is unknown among species of traditional Flabellinidae (Marcus and Marcus 1967; Millen and Hermosillo 2007). In combination with the anus in a higher acleioproctic position this puts *Kynaria cynara* apart from any other families analysed here or genera of traditional Flabellinidae. *Kynaria cynara* also demonstrates peculiar swimming behaviour (Marcus and Marcus 1967). No molecular data is available for *Kynaria cynara*, and it is separated into a new genus *Kynaria* gen. n. based on morphological data; its family placement as *incertae sedis* remains until molecular data are available.

## Discussion

### A new aeolid tree

Aeolidacean nudibranchs are a large and diverse group which are characterised by a single cnidosac in the cerata (Martin et al. 2008, 2010). Aeolidacea is traditionally comprised of the following families: Aeolidiidae, Calmidae, Cumanotidae, Eubranchidae, Facelinidae, Fionidae, Flabellinidae, Notaeolidiidae, Pseudovermidae, Protaeolidiidae, and Tergipedidae (Odhner 1939; Thompson and Brown 1984; Rudman 1998). Recently, Protaeolidiidae has been moved to Facelinidae (Carmona et al. 2015), and most controversially, a large cluster of traditional families such as Calmidae, Eubranchidae, and Tergipedidae has been moved to the aberrant planktonic and previously monotypic family Fionidae because of its apparent molecular relationship and because of simple priority of the latter family (Cella et al. 2016). The present study reveals an extremely complicated phylogenetic and hence taxonomic pattern of the traditional family “Flabellinidae”, including triple polyphyly in relation to the other traditional families such as Aeolidiidae, Calmidae, Eubranchidae, Fionidae and Tergipedidae. Previously, paraphyly of “Flabellinidae” has been noted based on morphological analysis (Wägele and Willan 2000), but it was never tested on a broad taxon selection using molecular phylogenetic tools.

Despite the fact that there is an apparently monophyletic core of “flabellinid” families, including Coryphellidae, Flabellinidae *s. str.*, Flabellinopsidae and Paracoryphellidae, there are many examples when putative “flabellinid” species actually represent completely separate clades and families – Samlidae, Apataidae and Unidentiidae. Importantly, the latter three families are probably related to very different aeolid families, i.e., either to Aeolidiidae and Facelinidae or Calmidae, Eubranchidae, Fionidae, Tergipedidae. Thus, this case obviously suggests that there are only two plausible alternatives for classifying the aeolids after discovering polyphyly of the traditional “Flabellinidae”:

1) To unite ALL existing diverse aeolidacean taxa into a single family, by priority Aeolidiidae

2) To establish an adequate number of family-level groups to reflect phylogenetic and ontogenetic diversity.

The first alternative is simple and apparently rational, but will seriously obscure the extremely complicated phylogenetic patterns discovered here and ignore the astonishing morphological and molecular diversity of Aeolidacea. Furthermore, it will disregard any traditional aeolid family-level taxa and the well-established suborder Aeolidacea would also equal a family rank. Current practice in other invertebrate groups, e.g., echinoderm ophiuroids, confirms the plausibility of a differentiated taxonomic approach using an appropriate number of families in a broad phylogenomic study (O’Hara et al. 2017). Therefore, we have chosen the second alternative and fully consider phylogenetic patterns resulting in particular disparate morphologies by establishing as many minimally heterogeneous family- and genera-level groups as necessary.

Importantly, in the present study we have proposed morphological and molecular units which are as coherent as possible for such large groups of traditional “Flabellinidae”. For every family- and genus-level taxon, appropriate morphological diagnosis, including extensive illustrations, and molecular distances which show significant values between all newly separated genera and families are presented. This is not a traditional “splitting” approach since it employs two major objective characteristics: maximally homogenous morphology within a genus coupled with significant molecular distances (for the latter see also Tables 1 and 2). The range of possible modifications at family level is expected to be higher than at a genus level. One may argue that, in this respect, since Coryphellidae, Flabellinidae, Flabellinopsidae fam. n., and Paracoryphellidae likely have a common monophyletic ancestry, they could be united into a single family. However, common ancestry does not imply a particular taxonomic arrangement into any one family, for example. Each of the families demonstrates its own unique characteristics and evolutionary trends. For example, within Coryphellidae and Paracoryphellidae there are no genera with stalked or elevated ceratal rows, whereas in Flabellinidae this is a common trend. However, in Flabellinidae there are no taxa with a broad continuous notal edge, whereas without exception all Paracoryphellidae possess a well-defined notal edge. In turn, the family Flabellinopsidae fam. n. shows evident features of independence from Coryphellidae and Flabellinidae in the reduction of the plesiomorphic notal edge and so needs to be separated. Apart from phylogenetic arguments for the distinctness of this family (which is enough for many researchers in the present taxonomic paradigms), the family Flabellinopsidae, despite low node support has its own peculiar morphological characteristic, such as the presence of flap-like notal edges instead of stalked or elevated notal edges as in the family Flabellinidae *sensu stricto*. Additionally, there are no distinct oral glands in Flabellinopsidae, whereas true flabellinids, even those without elaborate stalks (like in the genera Carronella and Edmundsella) always possess oral glands. Furthermore, regarding the notal edge within the family Coryphellidae a peculiar evolutionary mosaic occurs, when several genera with a continuous notal edge are commonly more basal in relation to the genera with a reduced discontinuous notal edge (Fig. 2). This reflects the plesiomorphic retention of a well-defined notal edge and we cannot avoid separating such considerable diversity in a combination of plesiomorphic and apomorphic features into particular taxa. Instead, it is more natural to include groups of related (but still clearly separated genera, according to the morphological data and molecular distances: see Tables 1 and 2), with both a continuous and a discontinuous notal edge into the family Coryphellidae.

Thus, for the future of new taxa, it is more practical to separate taxa with a number of unique features at the family level instead of lumping them into an extremely heterogeneous (and large) grouping. With narrow morphological and molecularly separate taxa, there would be no need to compare potential new taxa to a large heterogeneous assemblage. The most crucial result of the present study showed that a considerable number of tropical taxa that were previously considered within the single genus *Flabellina* actually had nothing phylogenetically in common not only with Flabellinidae or Coryphellidae, but with a majority of the aeolidaceans and are in fact placed very basally (Fig. 2). This is one of the best arguments for the practical reliability of the “small coherent unit/group” approach in taxonomy since previously such “flabellinid” taxa were already separated into at least one separate genus, *Samla* (Bergh 1900b) using only morphological arguments, but such prophetic suggestions were completely discarded in a dismissive pan-*Flabellina* concept. Therefore, it is both reliable and logical to separate this taxon into the family Samlidae fam. n. since it is placed very far from any proper Flabellinidae phylogenetically (Fig. 2), using both molecular and morphological arguments. One more surprising result comes also from a temperate and subtropical taxon, “*Flabellina*” *pricei*, which was never considered as separate from *Flabellina* despite its distinct morphological characteristics. This is a remarkable case since “*Flabellina*” *pricei* is one of the most basal aeolidaceans elucidated so far, and phylogenetically also has nothing in common with Flabellinidae or any other related families. “*Flabellina*” *pricei* has a very special morphological characteristic which includes comb-like ceratal rows, unique for any Flabellinidae or Coryphellidae but occurring within Trinchesiidae, which is considerably different from “*Flabellina*” *pricei* both phylogenetically and morphologically. Thus, it is fully justified to separate the very distinct “*Flabellina*” *pricei* into a separate genus *Apata* gen. n. and family Apataidae fam. n.

One of the most reliable indications of the usefulness of the “small unit” approach is the Unidentiidae family story: when it was recently separated using only morphological data (Millen and Hermosillo 2012) this suggestion was subsequently discarded by placement of evidently different, uniserial, taxon with a reproductive system (including hollow stylet) very different from all flabellinids, within the genus *Flabellina* (Gosliner et al. 2015). In our present study it is consistently shown that Unidentiidae is in reality completely separate from any flabellinid taxon, and is placed basally to the family Facelinidae (Fig. 2).

Of course, we are dealing with living organisms which are comprised of numerous elements which may show some variation even within such small groups, and there is still some arbitrariness in naming, even if the smallest units for the genera have been utilised. However, an important step is presented here wherein complicated ontogenetic information (= morphology in broad sense) and phylogenetic molecular data are reflected in the classification; surely not without potential pitfalls, but this is among the first attempts of this kind in nudibranch taxonomy. The resulting taxonomic units are not simply theoretical, but of great practical use since within each family and genus, further hidden diversity it is expected to be found. This was confirmed while this paper was under review by the most recent notion about “cryptic diversity” within several coryphellid and flabellinid taxa (Furfaro et al. 2017). To describe such hidden diversity will now be much easier and more plausible within these smaller genera than within a single large, extremely heterogeneous, assemblage like *Flabellina* *sensu* Gosliner and Griffiths (1981), which included taxa which are scattered among many major aeolidacean families.

Furthermore, there is remarkable agreement in the biogeographic patterns of distribution with the results of the morphological and molecular phylogenetic analyses of the various families discussed above. For example, the family Paracoryphellidae is an exclusively cold-water group and inhabits only the Arctic and the coldest parts of the adjacent northern Atlantic and Pacific oceans strongly influenced by Arctic waters. There are no records of any paracoryphellids from subtropical or tropical regions. The members of the family Coryphellidae are instead predominantly temperate (boreal) species whose diversity declines in the subtropical waters and is largely unknown in the tropics. Flabellinidae *sensu stricto* in turn includes mostly tropical and subtropical representatives, and only two genera with few species, *Edmundsella* and *Carronella*, penetrate boreal waters under the influence of warm currents like the Gulf Stream, but never occur further north in the Arctic regions. The family Apataidae has a similar distribution as the family Coryphellidae. Finally, species from the families Samlidae fam. n. and Flabellinopsidae fam. n. are exclusively tropical and subtropical taxa, and have not been recorded even from relatively warm boreal waters. These biogeographical trends with disparate morphological and molecular data may therefore reflect the evolutionary pathways of each of these families and further contribute to support of the new classification.

### A new aeolid classification

Is it really necessary to propose a reclassification based on so many re-established and newly created taxa? As is shown in the present study, the traditional family Flabellinidae is split by numerous definitely non-flabellinid taxa such as Aeolidiidae, Glaucidae, Facelinidae *s. l.*, Tergipedidae, Eubranchidae, and others. If we want to preserve their traditional family ranks, we need to establish new family-level clades within the polyphyletic Flabellinidae accordingly. On the other hand, our topology under the logic of pan-unification (e.g., Cella et al. 2016) implies that we would have to consider almost all aeolidacean families as just a single family, by priority Aeolidiidae Gray, 1827 or Glaucidae Gray, 1827. This would result in the creation of a morphologically extremely heterogeneous assembly, which would be very complicated and practically unmanageable since the description of any new taxa would have to indicate to which particular group within this “super-grouping” a new taxon belongs. Furthermore, it would not be possible to provide any clear diagnosis of such unified groups, even with reservations and exceptions, because almost every character would have alternative states. The only possible diagnosis for such a putative “pan-aeolidacean” family could be the presence of a single cnidosac in the cerata. However, this character is not unique enough since in several non-aeolidaceans (e.g., *Embletonia, Hancockia*) cnidosac-related structures are present (Martin et al. 2009, 2010). Both theoretical and practical unreliability of such pan-unification suggestions is clearly demonstrated by a recent publication (Cella et al. 2016). In that publication a diagnosis for the family Fionidae in the widest sense was provided, and included among other characteristics, the presence of an anus, and the notion that “The penis usually has always has a distinct penial gland at the posterior end of the penis” [sic] (Cella et al. 2016: 12). However, such a diagnosis is clearly untenable since the genus *Calma*, uniquely among aeolidaceans, has no anus, several species of the genus *Cuthonella* have a penial gland which is inserted into the vas deferens and not to the penis (Martynov 1992, 2006b; Cella et al. 2016), and the genus *Fiona* does not have a penial gland at all. Such facts clearly contradict the diagnosis of Fionidae *sensu lato*, but for some reason they were overlooked in Cella et al. (2016), perhaps due to the emphasis the authors placed on uniting the diversity of the tergipedid-like nudibranchs under a single family name. Finally, their pan-unification suggestion ignores one of the main principles of taxonomy (to reveal the hierarchy of taxa) since it makes a family- and an order-level taxon equal and merges family-level taxa into one order-level taxon. For example, the genus *Calma* is related to the genus *Cuthonella* according to molecular data (Figs 1, 2) but dramatically differs from all known aeolidaceans by the absence of an anus in the adult state. Such a distinctive feature should not be obscured by taxonomically uniting the genus *Calma* with *Cuthonella* within a single family. The traditional family Calmidae, along with other related families, was therefore reinstated (Korshunova et al. 2017a). The present molecular analysis reveals a similar tree topology and confirms this recent taxonomic decision. For the genus *Cuthonella*, which has an anus and, also uniquely for the “Tergipedidae *s. l.*” a supplementary (or penial gland) inserted to the vas deferens instead of the penis, Miller (1977) suggested the subfamily Cuthonellinae; this was raised to family level. Furthermore, a recently described genus *Murmania* (Martynov, 2006b) is placed basally to *Calma* and *Cuthonella*, according to the molecular data (Figs 1, 2). However, according to morphological data this genus has even more plesiomorphic features, unknown within the traditional Tergipedidae, including an extreme number of the ceratal rows which do not form regular rows, and a posterior cleioproctic or pleuroproctic anus; the latter features fit clearly within the diagnosis of the families Coryphellidae and Aeolidiidae rather than to traditional “Tergipedidae”. One more very unusual feature of *Murmania* is a triangular fold inclined between the rhinophores. Such a structure is completely absent not only within the traditional family Tergipedidae, but also is not known within any other aeolidacean group and somewhat resembles the caruncle in arminacean nudibranchs. At the same time, *Murmania* possesses a clearly defined penial gland which is inserted to the penial sac, as in most of the traditional Tergipedidae (but not in the related *Cuthonella*). However, *Murmania* is considerably separated from the other Tergipedidae *s. l.* taxa on the tree (Figs 1, 2). Therefore, if we merge *Murmania* with *Cuthonella* or *Calma* within the same family we will conceal these considerable morphological discrepancies within the other Tergipedidae. Therefore, the genus *Murmania* is separated here as the basis of a new family Murmaniidae fam. n. (see Suppl. material 1: Data S1).

The remainder of the traditional Tergipedidae are also morphologically heterogeneous and do not form a compact group according to both Cella et al. (2016) and the present analysis (Figs 1, 2). For example, the genus *Cuthona* *s. str.* (with the inclusion of only the type species *C. nana* and two very closely related species, *C. hermitophila* and *C. divae*) is placed basally and separately from major taxa that previously were considered within the very large genus “*Cuthona*” (*sensu* Williams and Gosliner 1979). Two further clades on the molecular tree (Cella et al. 2016, present study) include the genus *Tergipes* and the family of the extremely aberrant planktonic nudibranch genus *Fiona*. Only after this consideration is the main complex of the traditional family “Tergipedidae” separated, but without Tergipedidae itself (Figs 1, 2). For this group the family-level name Trinchesiidae Nordsieck, 1972 is available (Nordsieck 1972; Korshunova et al. 2017a).

The genus *Abronica* is somewhat externally similar to Trinchesiidae, but according to molecular phylogenetic data it is stably placed basally to Eubranchidae, which all possess a triserial radula and a penial gland; therefore, the separate family Abronicidae fam. n. is proposed here (see Suppl. material 1: Data S1).

Another special case is the genus *Rubramoena* (proposed in Cella et al. 2016). Morphologically it is similar to the family Trinchesiidae. According to the present molecular phylogenetic analysis based on Bayesian Inference (BI) (Fig. 1) *Rubramoena* is related to the morphologically very dissimilar Tergipedidae and not to Trinchesiidae. However, *Rubramoena* holds an unstable position; on Maximum Likelihood (ML) analyses (not shown) it clustered together with the family Trinchesiidae instead of Tergipedidae. Its needs further investigation and we refrain from creating a separate family for *Rubramoena* at present.

The family Facelinidae also includes very diverse assemblages, but in contrast to the Flabellinidae and Tergipedidae *s. l.* numerous narrower facelinid genera were already established (e.g., Schmekel 1966, 1967; Edmunds 1970; Schmekel and Portmann 1982; Willan 1987; Gosliner and Behrens 1986; Gosliner 1991; Millen and Hamann 1992; Rudman 1998; Hirano 1999; Millen and Hermosillo 2012). We do not intend to revise the Facelinidae in this study and the facelinid genera list is considered tentative, but there is evidence that more family-level taxa within this family should either be resurrected (e.g., Babakinidae) or created as new. For the purpose of the present analysis it is enough that the complexity of Facelinidae and Aeolidiidae together with the traditional Tergipedidae deeply divide the traditional Flabellinidae, rendering it not only paraphyletic, but actually a profoundly polyphyletic ensemble.

While we have used molecular data for the majority of the aeolidacean families, some such as Cumanotidae, Pseudovermidae, and Notaeolidiidae were not included in the major tree (Figs 1, 2). Both Cumanotidae and Pseudovermidae are distinct morphologically and are maintained as separate families (see Suppl. material 1: Data S1). The family Notaeolidiidae is important since it is the only aeolidacean family with more than three rows of lateral teeth, a remarkably plesiomorphic feature within Aeolidacea (Wägele and Willan 2000). A few sequences of the single species available, *Notaeolidia depressa* Eliot, 1905 have been included, and in some trees it appears as a sister clade to the families Samlidae and Apataidae. Due to insufficient data it has not been included in the final tree in the present study, but it may indicate a basal position of Notaeolidiidae as well. We propose, therefore, the following family system for the Aeolidacea (see Suppl. material 1: Data S1). We avoid the superfamily rank due to the current phylogeny not being fully settled, but the order of appearance reflects the general evolutionary pattern. The major implication of the present study is that the only possibility of constructing a truly practical, and not just a theoretical, “integrative” taxonomy is with careful and balanced employment of both molecular and morphological data.

The proposed 24 families herein are unequal by number of genera, reflecting current phylogenetic hypotheses based on the states of knowledge and molecular sampling. The presence of monotypic families or families with just a few genera is not unusual in traditional classifications and should not be an argument against our approach. The main goal is not to create as many taxa as possible, but to reflect the extremely complicated evolutionary pattern in a classification with maximal consistency by analysing both morphological and molecular data.

### Patterns of aeolid evolution

According to the molecular and morphological data presented here (Figs 1, 2) the main trends of aeolidacean evolution were reduction and modification of the well-defined notal edge and reduction of the triserial radula into a uniserial one. The only aeolid family that preserved the ancestral multiserial radula (more than one lateral tooth per transversal row, as found in non-aeolidacean nudipleurans) is the endemic Antarctic Notaeolidiidae. The triserial radula is retained as a key character within the Apataidae, Coryphellidae, Cumanotidae, Eubranchidae, Flabellinidae, Flabellinopsidae, Paracoryphellidae, Pseudovermidae, and Samlidae, whereas all the other aeolid families have evolved a uniserial radula. The genus *Paracoryphella* is the only paracoryphellid genus that may also possess a second row of reduced lateral teeth (Fig. 8E, 9K). Remarkably, traditional flabellinids largely retain an exclusively triserial radula. Presence of a triserial radula also within the family Eubranchidae, which according to the morphological (external and reproductive features) and molecular data is related to the families with exclusively uniserial radula, Abronicidae, Murmaniidae, Cuthonellidae, Calmidae, Cuthonidae, Tergipedidae, and Trinchesiidae rather than to traditional flabellinids suggests that the common ancestor of most of the aeolid families, after splitting from Notaeolidiidae-like ancestral group, had a triserial radula. Because a well-defined, ample notal edge is invariably present in all members of the order Pleurobranchomorpha, which is now considered to be the sister clade to all nudibranchs (including Aeolidacea) according to both morphological and molecular data (Martynov 2011; Kano et al. 2016), it is plausible to suggest that the aeolid ancestor had a well-defined notal edge furnished with numerous cerata. Such morphology is most similar to the modern members of the family Paracoryphellidae. However, from a molecular phylogenetic perspective, the family Paracoryphellidae, though quite basal in our tree (Fig. 2), does not occupy the most basal position within the clade of the traditional flabellinids, where it is placed in the family Flabellinopsidae, which have a modified, discontinuous notal edge. Furthermore, the present molecular analysis reveals a remarkable mosaicism of families with a rather narrow body and discontinuous notal edge. For instance, the families Apataidae and Samlidae are the most basal aeolidacean families (except for Notaeolidiidae, not shown here) on the present tree, but possess a rather slender body with reduced notal edge.

Such patterns reveal the limits of the molecular phylogenetic analysis in inferring the ancestral state of morphological characters, if we strictly follow the recovered molecular pattern. Instead, a broadly ontogenetic understanding of the evolutionary process and resulting taxonomic classification implies formulating a model of ancestral ontogenetic cycle that suggest, among other features, a well-defined notal edge as a common characteristic of ancestral ontogeny of the Nudibranchia *s. str.* (= Cladobranchia), including Aeolidacea (Martynov 2011). At the same time, molecular phylogenetic analysis may contribute significantly in revealing numerous cases of secondary reduction of various characters, which are otherwise not obvious using other approaches. For example, our tree suggests several independent cases of the reduction of the notal edge (among the families Apataidae, Coryphellidae, Flabellinidae), as well as independent reduction of the triserial radula into a unserial one within the Aeolidiidae-clades and within the Eubranchidae-Tergipedidae clade. Molecular support for the reliability of the broadly ontogenetic consideration of the initial presence of a well-defined notal edge is the basal occurrence of genera with a continuous notal edge within various clades of the family Coryphellidae (i.e., *Gulenia*, *Himatina*, *Borealia*, *Itaxia*). A notable case is when molecular phylogenetic analysis revealed an unexpected independent earlier reduction of both the notal edge and a triserial radula in the family Abronicidae (Fig. 2). This family, with a narrow body and uniserial radula appeared basally to the Eubranchidae, a family with a triserial radula, which is itself basal to the large clade with a uniserial radula containing several families with a wide body (Cuthonellidae, Cuthonidae, Fionidae *s. str.* and in one case even with a rudimental notal edge as in Murmaniidae). Thus, while molecular phylogenetics may distinguish clades with particular morphological features, the direction of their general evolution can be inferred only using a model of an ancestral ontogenetic cycle.

Another clear trend that features in the evolution of Aeolidacea as a whole group is the transformation of a pleuroproctic anus (on the right lateral side of the body below the cerata) into cleioproctic (within the cerata of the right posterior digestive gland) and acleioproctic ones (between anterior and posterior major branches of the right digestive gland). Like the reduction of the notal edge, the transition of the anus from pleuroproctic to cleioproctic and acleioproctic positions has occurred independently within many morphologically and molecularly consistent clades of the Aeolidacea. For example, within the predominantly acleioproctic traditional tergipedids, the families Cuthonidae and Cuthonellidae may contain cleioproctic forms, the family Murmaniidae is normally exclusively cleioproctic with the presence of pleuroproctic-like conditions in some specimens of *Murmania antiqua* (Martynov, 2006). Within Aeolidiidae a pleuroproctic anus occurs in some clades, like *Aeolidia* and *Cerberilla* (Carmona et al. 2013; Korshunova et al. 2017c). Especially evident is how the plesiomorphic pleuroproctic position of the hindgut opening, typical for such unquestionably basal groups such as Doridoxidae, Arminidae, and Tritoniidae has transformed into an acleioproctic-like position within the family Flabellinidae *s. str.* In this family, some genera (like *Carronella*) which are more basally placed on the molecular tree possess a pleuroproctic anus, whereas some terminal clades (like *Calmella*) have the anus in a transitional position which is shifted towards the acleioproctic position (Fig. 2). Interestingly, as already noted for the reduction of the notal edge, the translocation of the anal opening has occurred mosaically – for instance the family Apataidae, very basal on the molecular tree, already does not possess a notal edge and shows the anus in a higher acleioproctic position. The latter set of characters suggests that a reduction of notal edge and anus transition occurred in the Apataidae very early on and independently from the other Aeolidacea.

Regarding internal characters, it is important to discuss some evolutionary patterns of the reproductive system. Within the Paracoryphellidae, Flabellinopsidae, and Flabellinidae a long to moderate prostatic vas deferens is dominant (Figs 7, 34), whereas among the species-rich family Coryphellidae (to which Paracoryphellidae is more basal (Fig. 2) most of the genera have a short to very short vas deferens, but one of the most basal coryphellid genera *Microchlamylla* remarkably preserves a plesiomorphic long vas deferens (Fig. 21). The group of families of the traditional tergipedids (Calmidae, Cuthonidae, Cuthonellidae, Eubranchidae, Tergipedidae *s. str.*, Trinchesiidae) are characterised by the presence of a supplementary gland, which can be inserted either to the vas deferens or to the penis; however, this character cannot be a universal synapomorphy used to unite this large clade, since the family Fionidae *s. str.* is fully devoid of this supplementary penial gland. The family Cuthonellidae is invariably characterised by the supplementary gland being inserted into the vas deferens instead of the penis, but because the type species of such a morphologically and molecularly disparate family as Eubranchidae (*Eubranchus tricolor*) also has supplementary gland inserted into vas deferens, this is likely a plesiomorphic state of this character, translocated into the penis independently in several different families such as Cuthonidae, Tergipedidae *s. str.*, and Trinchesiidae. The family Murmaniidae is a notable example of an earlier independent acquisition of the supplementary gland inserted to the penis. A clear independent case of transition from vas deferens- to penis-inserted supplementary gland is the family Eubranchidae since the type species of the genus *Eubranchus*, *E. tricolor*, has the vas deferens-inserted supplementary gland, whereas the majority of other eubranchid genera possess a penis-inserted supplementary gland (Edmunds and Kress 1969; Martynov 1998). Interestingly, the family Eubranchidae, which is very uniform because of the invariable presence of a triserial radula, and is also monophyletic according to the molecular data, has shown a remarkably quick evolution, in a group of genera, of a very peculiar penis-free copulative apparatus containing a set of special fragile stylets of such form and structure that are unknown in other nudibranchs (Martynov 1998, 2005). The supplementary gland has been reported for at least one species of the family Unidentiidae (Gosliner 2010; but needs to be confirmed in additional material) and reported for a few genera of the family Facelinidae. On the contrary, the family Aeolidiidae possesses quite a conservative and simple copulative apparatus. The penial stylet has appeared in several genera of the family Facelinidae and independently, in the family Tergipedidae *s. str.*, all species of the families Trinchesiidae, and in the majority of Eubranchidae.

Most of the family-level clades demonstrate conservatism and little to moderate morphological diversity concordant with the molecular data (Fig. 2), but may significantly differ in species number. For example, such families as Aeolidiidae, Coryphellidae, Cuthonellidae, Eubranchidae, Flabellinidae *s. str.*, Paracoryphellidae, Samlidae, and Trinchesiidae include considerable species diversity, whereas some others, Apataidae, Abronicidae, Calmidae, Cuthonidae, Fionidae *s. str.*, Murmaniidae, Tergipedidae *s. str.*, are morphologically and molecularly disparate from other families yet contain only few species. This may be influenced by several factors, including still undiscovered or not yet fully described hidden diversity within a given clade, produced by a rapid evolution during a quick transition to a fundamentally new niche (particularly relevant for the families Abronicidae, Cuthonidae, and Fionidae *s. str.*), that were responsible for a number of drastically different apomorphies (as in Calmidae, Fionidae *s. str.*, and Tergipedidae *s. str.*), and in some families may be also due to an extinction of most of the closely related genera and/or species. This is pertinent for the family Murmaniidae, which has a number of morphological archaisms such as a very broad body with numerous ceratal rows and rudiments of a notal edge, which is otherwise rare or unknown within its molecular tergipedid super-clade. Hidden species diversity, both molecular and morphological, has been discovered in almost all the families studied here, suggesting that further research in this direction is highly desirable.

## Conclusions

The main result of this molecular tree is that several well-separated clades consisting of various taxa that are currently included in the single traditional family Flabellinidae, and in the single huge genus *Flabellina*, have been recovered. Several clades have been robustly revealed as placed very distant from Flabellinidae *s. str.* and deeply divided by other aeolidacean families (Figs 1, 2). Thus, it is no longer possible to use the family Flabellinidae in the traditional sense any more. In many cases our molecular clades have coincided with families which were previously described using only morphological data. In several other cases, no appropriate family groupings were available, and to accommodate the robust molecular data several new families have been proposed (e.g., Apataidae fam. n., Samlidae fam. n.).

Another remarkable case of significant agreement between earlier morphological data and new molecular results is the family Unidentiidae, which was recently described using only morphological data by Millen and Hermosillo (2012), but found robust molecular support in our study. In some cases families have low statistical support (for example, Flabellinopsidae fam. n.), partly because only three taxa are included in the molecular analysis. However, this clade was revealed as a separate one both by BI and ML analyses. The low bootstraps on the branches joining the families are actually what demonstrate the need for these clades to be treated as separate families; the phylogenetic relationships with other families are not fully uncovered because they are deep in the evolutionary history of the group. Finally, Flabellinopsidae fam. n. has unique morphological features (including presence of special flap-like lobes of the notal edge, instead of stalks in proper Flabellinidae).

Our comprehensive multi-locus analysis of aeolid sea slugs suggests a new phylogenetic hypothesis with a polyphyletic classical Flabellinidae. To address relationships and disparity we propose a new family system for aeolids. Here the aeolidacean species are classified into at least 102 genera and 24 families, with possibly more families and genera not analysed herein. Our molecular tree is robust in large parts and suggests that the morphological character evolution within aeolids is even more complex than expected.

## Plates

**Figure 35.**
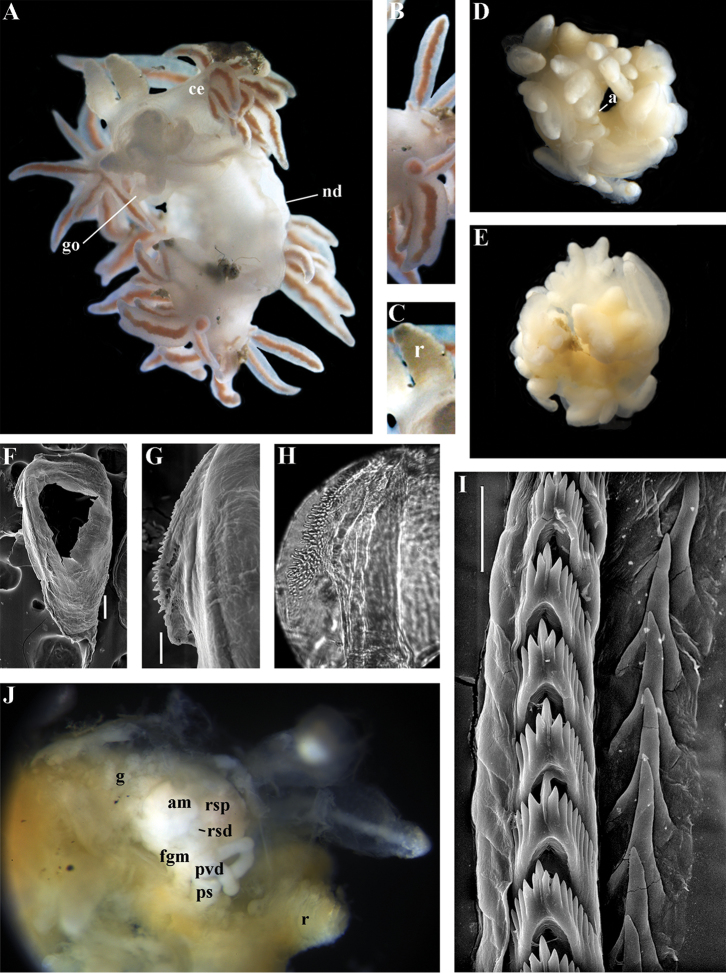
*Carronella enne* sp. n. NE Atlantic, off Ireland. ZMMU Op-526, specimen 5 mm (fixed) in length: **A** dorsal view (live); **B** details of cerata **C** rhinophore **D** dorsal view (fixed) **E** ventro-lateral (fixed) **F** jaw, SEM **G** details of masticatory process of jaw, SEM **H** details of masticatory process of jaw, light microscopy **I** radular teeth, posterior part, SEM **J** reproductive system, light microscopy. Abbreviations: **a** anus **am** ampulla **ce** ceratal elevations **fgm** female gland mass **g** gonad **go** genital opening **nd** discontinuous notal edge **ps** penial sheath **pvd** prostatic vas deferens **r** rhinophores **rsd** distal receptaculum seminis **rsp** proximal receptaculum seminis. Scale bars: **F** = 300 μm; **G, I** = 30 μm. Photos of living specimens by E. Schwabe, other photos and SEM images by A.V. Martynov.

**Figure 36.**
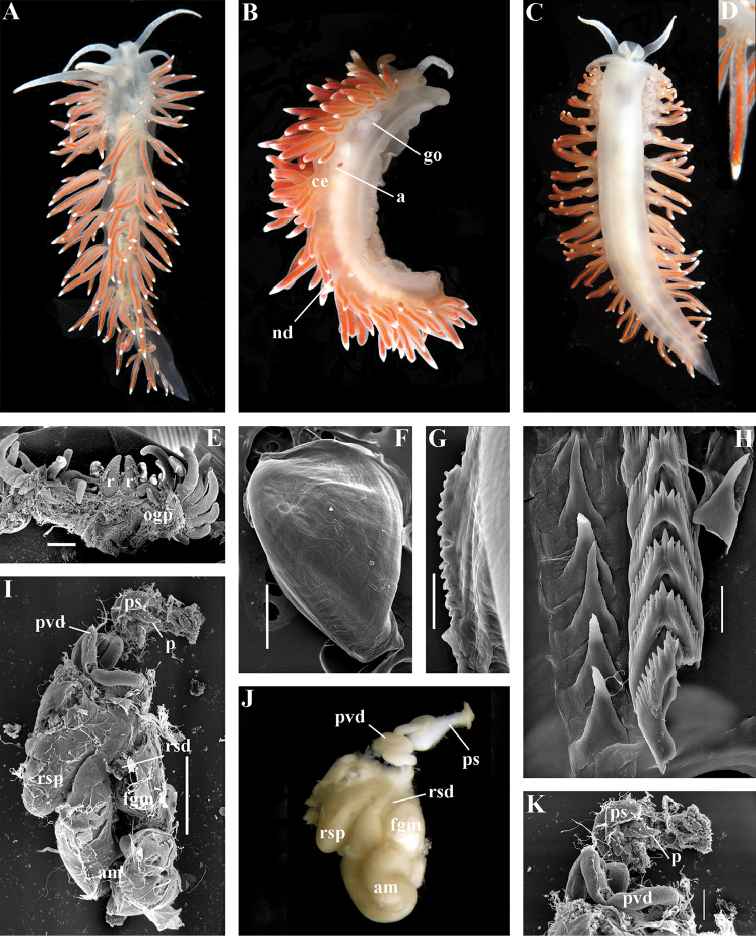
*Carronella pellucida* (Alder & Hancock, 1843), comb. n. Norwegian Sea, Gulen Dive Center. ZMMU Op-513, living specimen 18.5 mm in length: **A** dorsal view **B** lateral view **C** ventral view **D** details of cerata **E** dissected anterior part (pharynx removed) and rhinophores, SEM **F** jaw SEM **G** details of masticatory process of jaw, SEM **H** radular teeth, posterior part, SEM **I** reproductive system, SEM **J** reproductive system, light microscopy **K** dissected penial sheath and penis, SEM. Abbreviations: **a** anus **am** ampulla **ce** ceratal elevations **fgm** female gland mass **go** genital opening **nd** discontinuous notal edge **ogp** oral gland penetrating into basis of cerata **r** rhinophores **p** penis **ps** penial sheath **pvd** prostatic vas deferens **rsd** distal receptaculum seminis **rsp** proximal receptaculum seminis. Scale bars: **E** = 1 mm; **F, K** = 300 μm; **G** = 30 μm; **H** = 30 μm; **I** = 1 mm. Photos and SEM images by T.A. Korshunova, A.V. Martynov.

**Figure 37.**
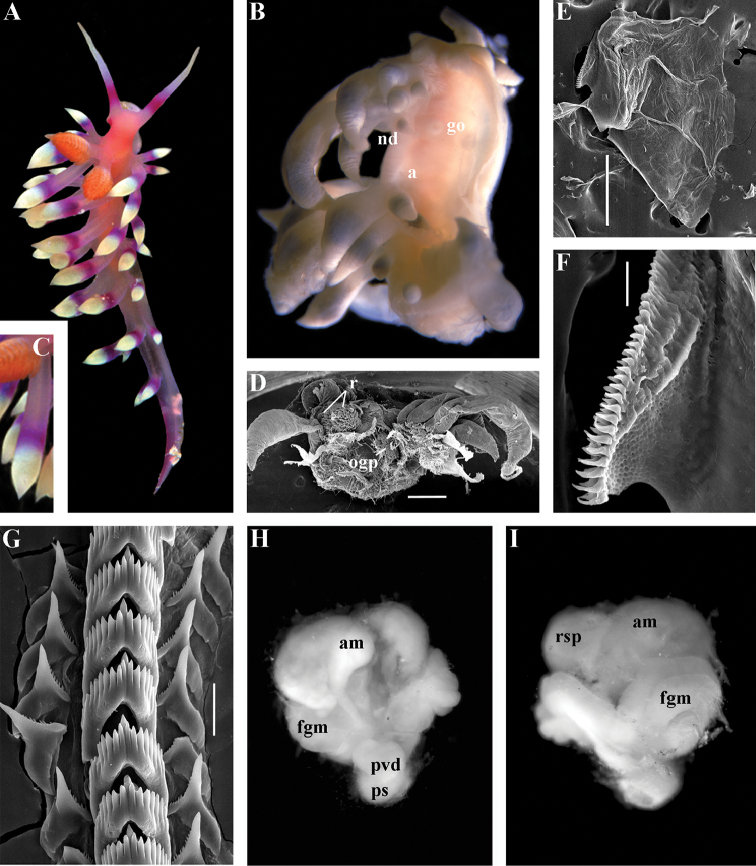
*Coryphellina exoptata* (Gosliner & Willan, 1991), comb. n. Vietnam, Nhatrang Bay. ZMMU Op-116, living specimen 9 mm in length: **A** dorsal view **B** ventral view (fixed) **C** details of cerata **D** dissected anterior part (pharynx removed) and rhinophores, SEM **E** jaw, SEM **F** details of masticatory process of jaw, SEM, scale bar = 30 μm **G** radular teeth, posterior part, SEM **H, I** reproductive system, light microscopy. Abbreviations: **a** anus **am** ampulla **fgm** female gland mass **go** genital opening **nd** discontinuous notal edge **ogp** oral gland penetrating into basis of cerata **ps** penial sheath **r** rhinophores **rsp** proximal receptaculum seminis **pvd** prostatic vas deferens. Scale bars: **D** = 1 mm; **E** = 300 μm; **F** = 30 μm. Photos of living specimens by O.V. Savinkin, other photos and SEM images by A.V. Martynov.

**Figure 38.**
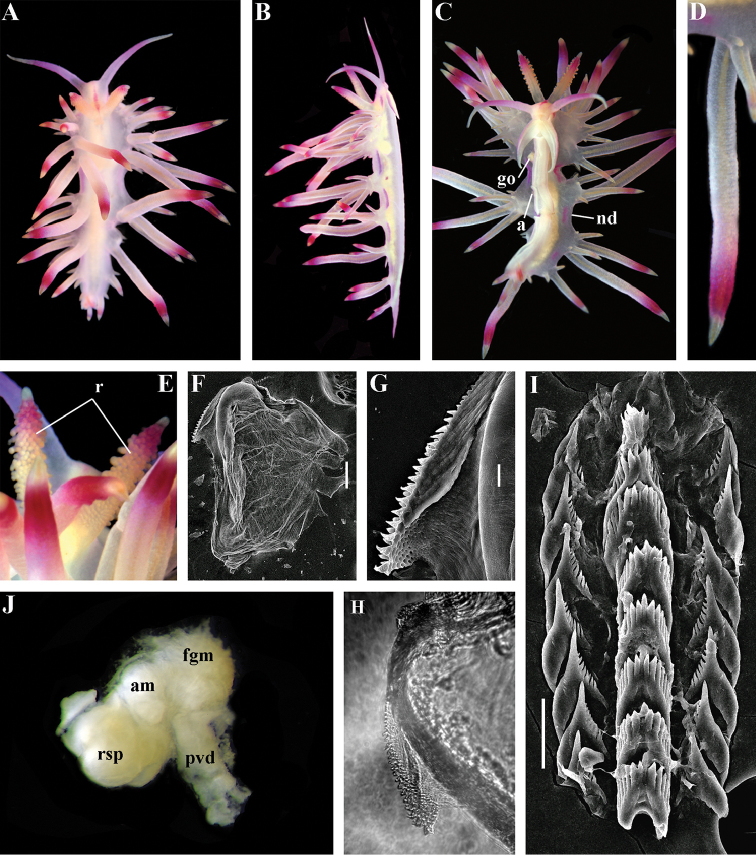
*Coryphellina lotos* sp. n. Japan, Pacific Honshu, Osezaki. ZMMU Op-515, living specimen 15.5 mm in length: **A** dorsal view **B** lateral view **C** ventral view **D** details of cerata **E** rhinophores, close up **F** jaw SEM **G** details of masticatory process of jaw, SEM **H** details of masticatory process of jaw, light microscopy **I** radular teeth, posterior part, SEM **J** reproductive system, light microscopy. Abbreviations: **a** anus **am** ampulla **fgm** female gland mass **go** genital opening **nd** discontinuous notal edge **pvd** prostatic vas deferens **r** rhinophores **rsp** proximal receptaculum seminis. Scale bars: **F** = 100 μm; **G** = 20 μm; **I** =50 μm. Photos and SEM images by T.A. Korshunova, A.V. Martynov.

**Figure 39.**
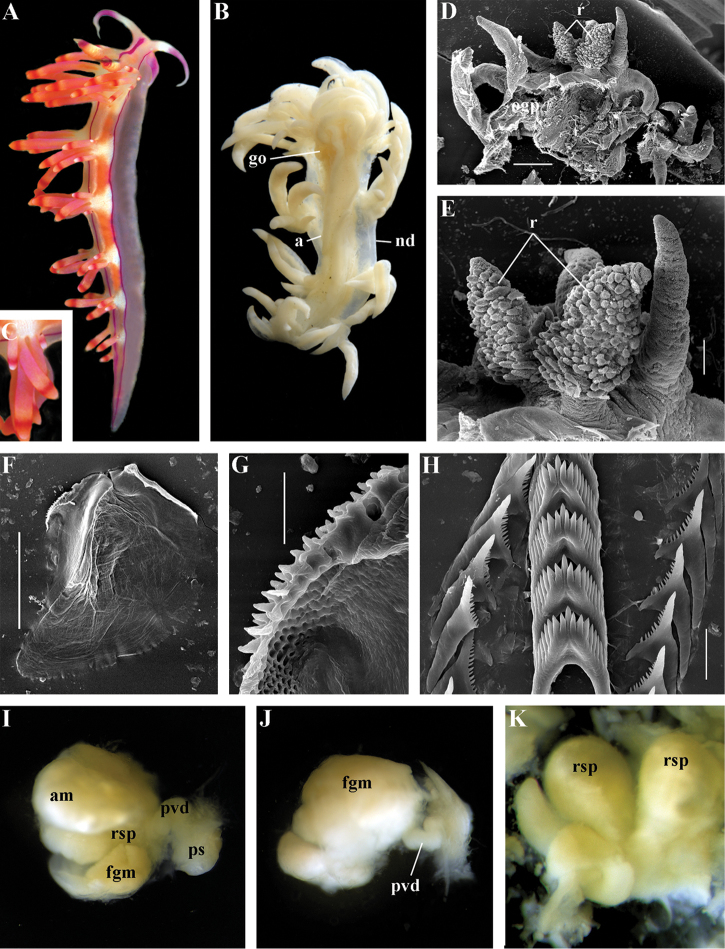
*Coryphellina rubrolineata* O’Donoghue, 1929. Vietnam, Nhatrang Bay. ZMMU Op-132, living specimen 15 mm in length: **A** dorsal view **B** ventral view (fixed) **C** details of cerata **D** dissected anterior part (pharynx removed) and rhinophores, SEM, scale bar 1 mm **E** rhinophores, close up, SEM **F** jaw, SEM **G** details of masticatory process of jaw, SEM **H** radular teeth, posterior part, SEM **I, J** reproductive system, light microscopy **K** bilobed proximal receptaculum seminis. Abbreviations: **a** anus **am** ampulla **fgm** female gland mass **go** genital opening **nd** discontinuous notal edge **ogp** oral gland penetrating into basis of cerata **ps** penial sheath **rsp** proximal receptaculum seminis **pvd** prostatic vas deferens **r** rhinophores. Scale bars: **D, F** = 300 μm; **G, H** = 30 μm. Photos of living specimens by O.V. Savinkin, other photos and SEM images by A.V. Martynov.

**Figure 40.**
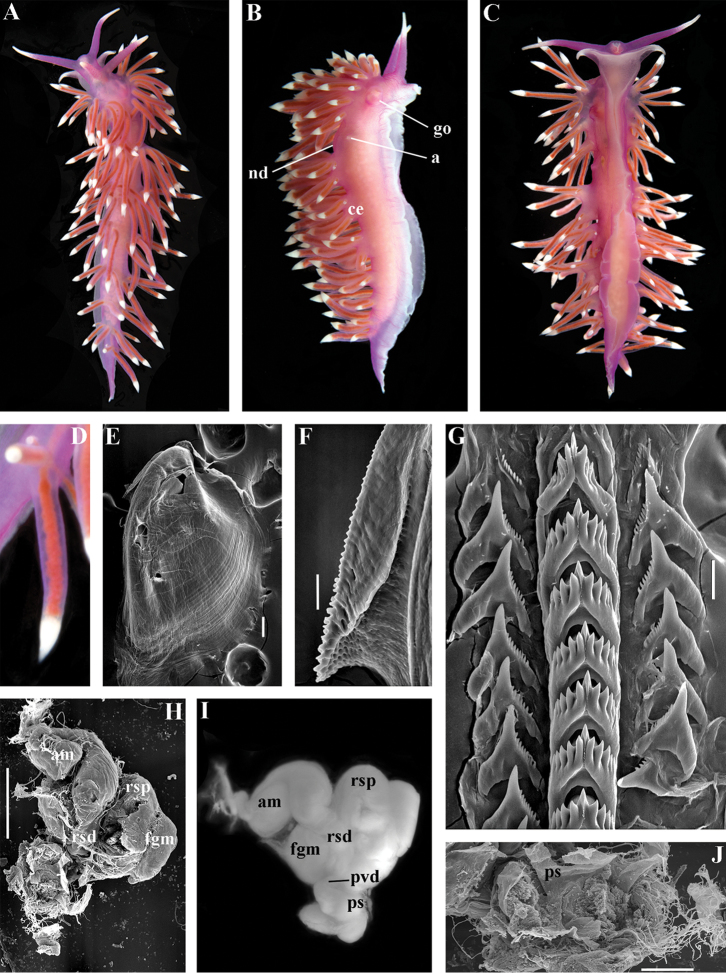
*Edmundsella pedata* (Montagu, 1815), comb. n. Norwegian Sea, Gulen Dive Center. ZMMU Op-540, living specimen 28 mm in length: **A** dorsal view **B** lateral view **C** ventral view **D** details of cerata **E** jaw, SEM **F** details of masticatory process of jaw, SEM **G** radular teeth, posterior part, SEM **H** reproductive system SEM **I** reproductive system, light microscopy **J** penial sheath dissected. Abbreviations: **a** anus **am** ampulla **ce** ceratal elevations **fgm** female gland mass **go** genital opening **nd** discontinuous notal edge**ps** penial sheath **pvd** prostatic vas deferens **rsd** distal receptaculum seminis **rsp** proximal receptaculum seminis. Scale bars: **E, H, J** = 100 μm; **F** = 20 μm; **G** = 50 μm. Photos and SEM images by T.A. Korshunova, A.V. Martynov.

**Figure 41.**
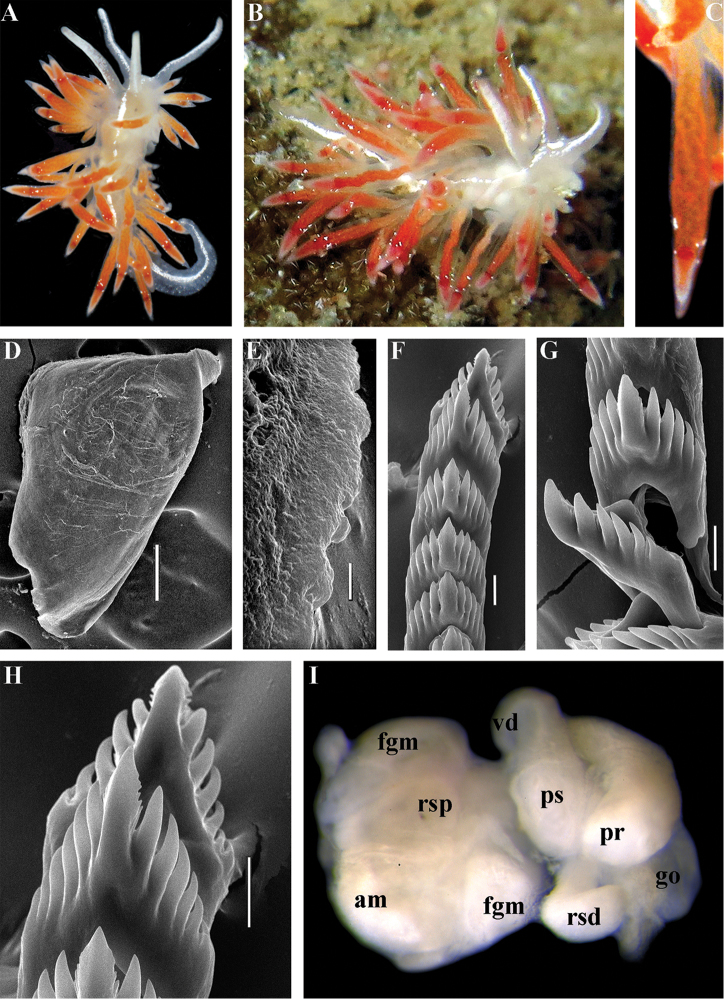
*Pacifia amica* sp. n., NE Pacific, Port Orchard, holotype ZMMU Op-614: **A** live, dorsal view, 6.5 mm in length **B** lateral view **C** details of cerata **D** jaws, lateral view **E** details of masticatory processes of jaw **F** posterior rows of radula, SEM **G** anterior rows of radula **H** details of posterior radular teeth **I** reproductive system in situ, light microscopy. Abbreviations: **am** ampulla **fgm** female gland mass **go** genital opening **pr** prostate **ps** penial sheath **rsd** distal receptaculum seminis **rsp** proximal receptaculum seminis **vd** vas deferens. Scale bars: **D** = 100 μm **E** =2 μm; **F, G, H** = 10 μm. Photos of living specimens by K. Fletcher, other photos and SEM images by A.V. Martynov.

**Figure 42.**
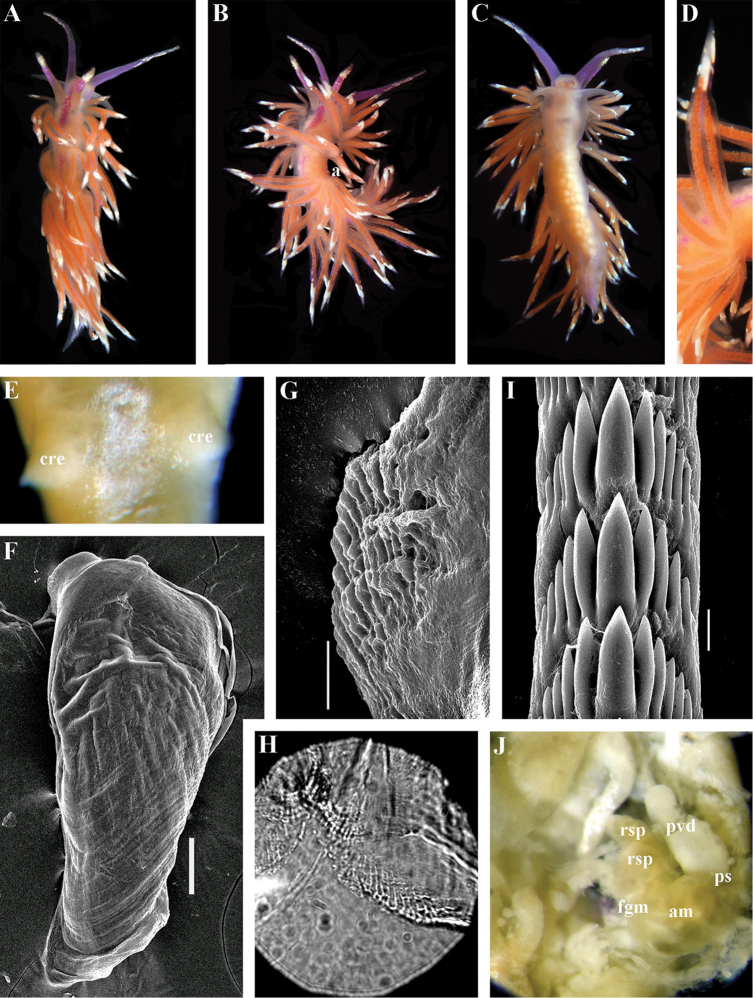
*Unidentia nihonrossija* sp. n. Japan, Pacific Honshu, Osezaki. ZMMU Op-517, specimen 4.5 mm in length (fixed): **A** dorsal view (live) **B** lateral view (live) **C** ventral view (live) **D** details of cerata (live) **E** particular elongate elevations at the base of cerata (fixed) **F** jaw, SEM **G** details of masticatory process of jaw, SEM **H** details of masticatory process of jaw, light microscopy **I** radular teeth, posterior part, SEM **J** reproductive system, light microscopy. Abbreviations: **a** anus **am** ampulla **cre** ceratal elevated rows **fgm** female gland mass **ps** penial sheath **pvd** prostatic vas deferens **rsp** proximal receptaculum seminis. Scale bars: **F** = 100 μm; **G, I** = 10 μm. Photos and SEM images by T.A. Korshunova, A.V. Martynov.

**Figure 43.**
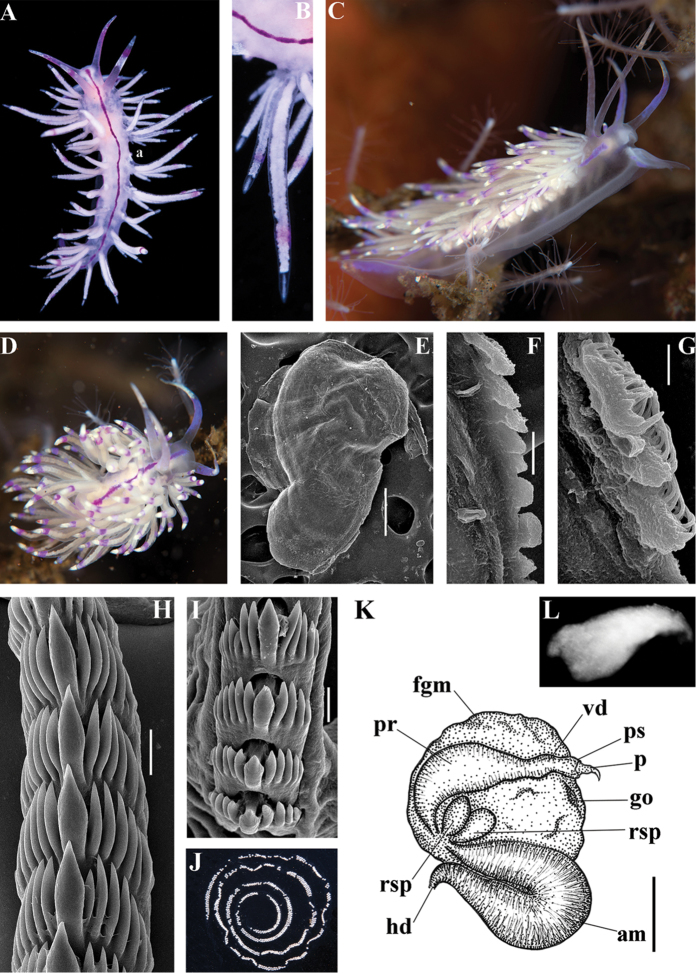
*Unidentia sandramillenae* sp. n. **A** holotype ZMMU Op-617, live, dorsal view , 7 mm in length (fixed) **B** same, details of cerata **C** uncollected specimen, lateral view **D** same, among athecate hydroids, possible prey **E** holotype, jaws, lateral view, SEM **F G** details of masticatory processes of jaw **H** posterior rows of radula, SEM **I** anterior rows of radula **J** egg mass **K** scheme of reproductive system **L** penis with hollow stylet, light microscopy. Abbreviations: **am** ampulla **fgm** female gland mass **go** genital opening **hd** hermaphoroditic duct **p** penis **pr** prostate **ps** penial sheath **vd** vas deferens **rsp** proximal receptaculum seminis Scale bars: **E** = 200 μm; **F** =10 μm; **G** = 5 μm; **H, I** = 20 μm; **K** = 0.5 mm. Photos of living specimens and eggs by B. Picton, other photos and SEM images by A.V. Martynov, drawings by T.A. Korshunova.

**Figure 44.**
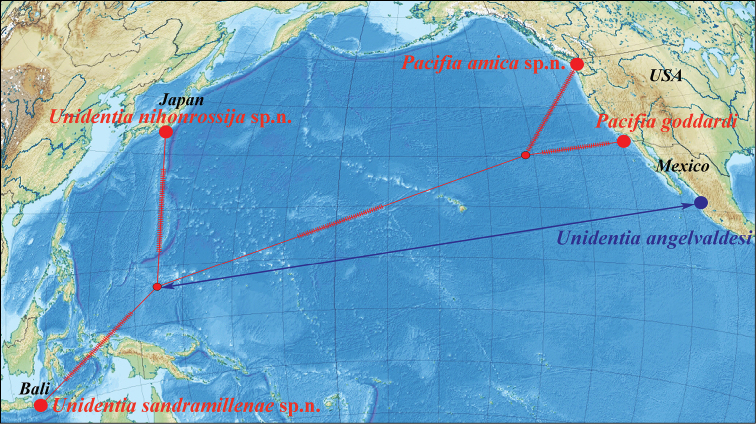
Haplotype network based on molecular data COI for the taxa of the family Unidentiidae (red lines) superimposed on the map of the Pacific Ocean. Predicted relationship between *Unidentia angelvaldesi* and other Unidentiidae is indicated by blue line.

**Figure 45.**
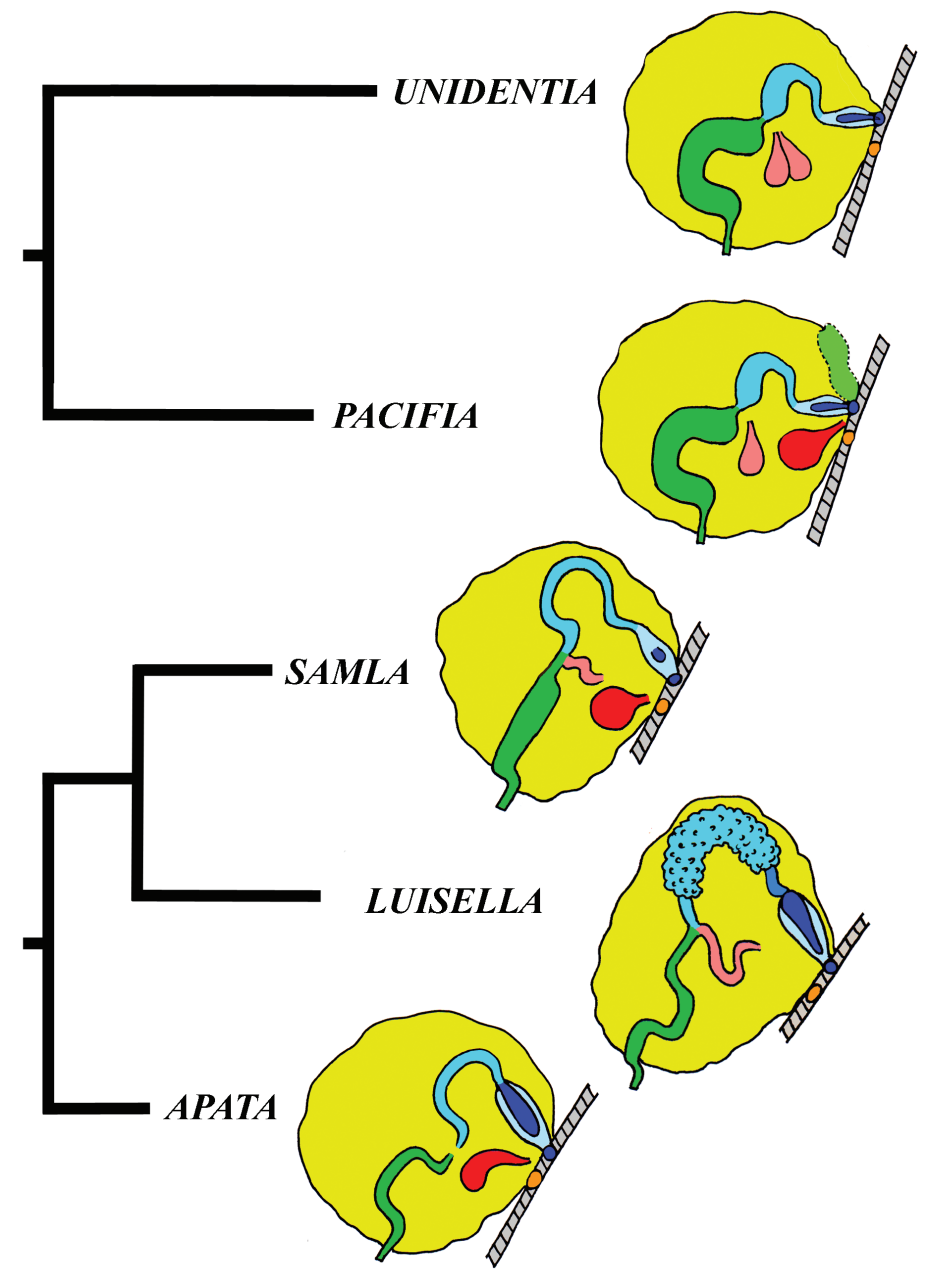
Schematic outline of the reproductive systems of the taxa of the families Unidentiidae, Samlidae fam. n. and Apataidae fam. n. integrated with molecular phylogenetic data. Colour indication of reproductive system characters: ampulla – green; body wall – gray; distal receptaculum seminis – red; female gland mass – yellow; female genital opening – orange; penis and male genital opening – dark blue; penial sheath – pale blue; prostate and prostatic vas deferens – turquoise; proximal receptaculum seminis – pink; supplementary gland – light green.

**Figure 46.**
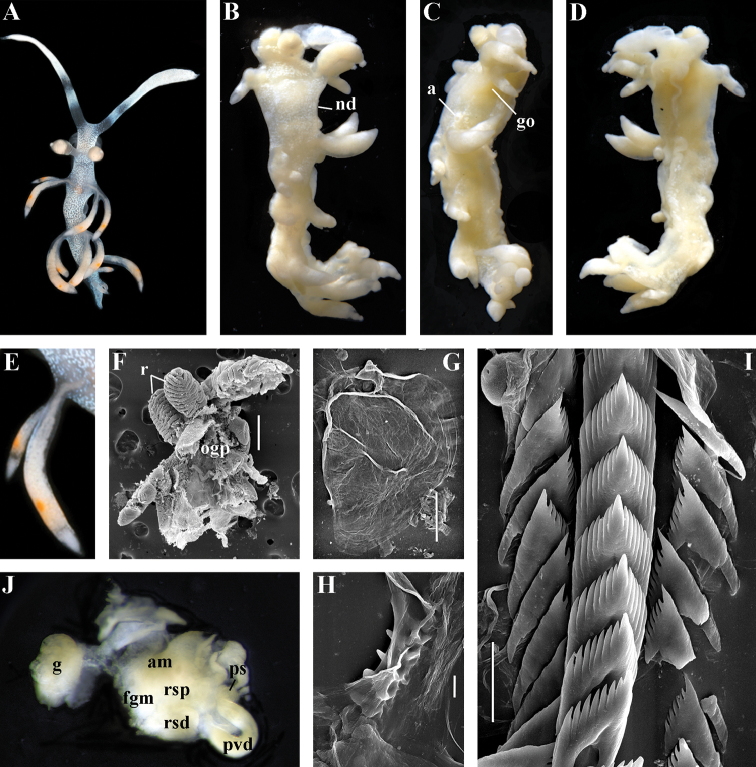
*Samla bicolor* (Kelaart, 1858). Vietnam, Nhatrang Bay. ZMMU Op-68, living specimen 5 mm in length: **A** dorsal view **B** dorsal view (fixed) **C** lateral view (fixed) **D** ventral view (fixed) **E** details of cerata **F** dissected anterior part (pharynx removed) and rhinophores, SEM **G** jaw, SEM **H** details of masticatory process of jaw, SEM **I** radular teeth, posterior part, SEM **J** reproductive system, light microscopy. Abbreviations: **a** anus **am** ampulla **fgm** female gland mass **g** gonad **go** genital opening **nd** discontinuous notal edge **ogp** oral gland penetrating into basis of cerata **ps** penial sheath **pvd** prostatic vas deferens **r** rhinophores **rsd** distal receptaculum seminis **rsp** proximal receptaculum seminis. Scale bars: **F** = 300 μm; **G** = 100 μm; **H** = 10 μm; **I** = 30 μm. Photos of living specimens by O.V. Savinkin, other photos and SEM images by A.V. Martynov.

**Figure 47.**
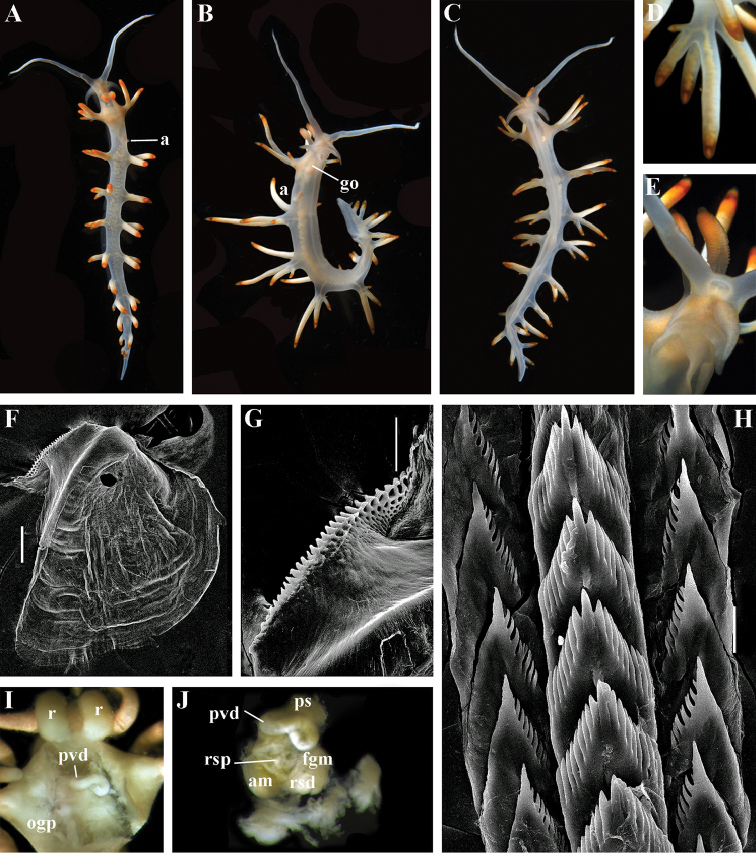
*Samla takashigei* sp. n. Japan, Pacific Honshu, Osezaki. ZMMU Op-530, living specimen 26 mm in length: **A** dorsal view **B** lateral view **C** ventral view **D** details of cerata **E** rhinophores **F** jaw, SEM **G** details of masticatory process of jaw, SEM **H** radular teeth, posterior part, SEM **I** dissected anterior part with reproductive system, light microscopy **J** reproductive system, light microscopy. Abbreviations: **a** anus **am** ampulla **fgm** female gland mass **go** genital opening **ogp** oral gland penetrating into basis of cerata **ps** penial sheath **pvd** prostatic vas deferens **r** rhinophores **rsd** distal receptaculum seminis **rsp** proximal receptaculum seminis. Scale bars: **F** = 100 μm; **G** = 50 μm; **H** = 20 μm. Photos and SEM images by T.A. Korshunova, A.V. Martynov.

**Figure 48.**
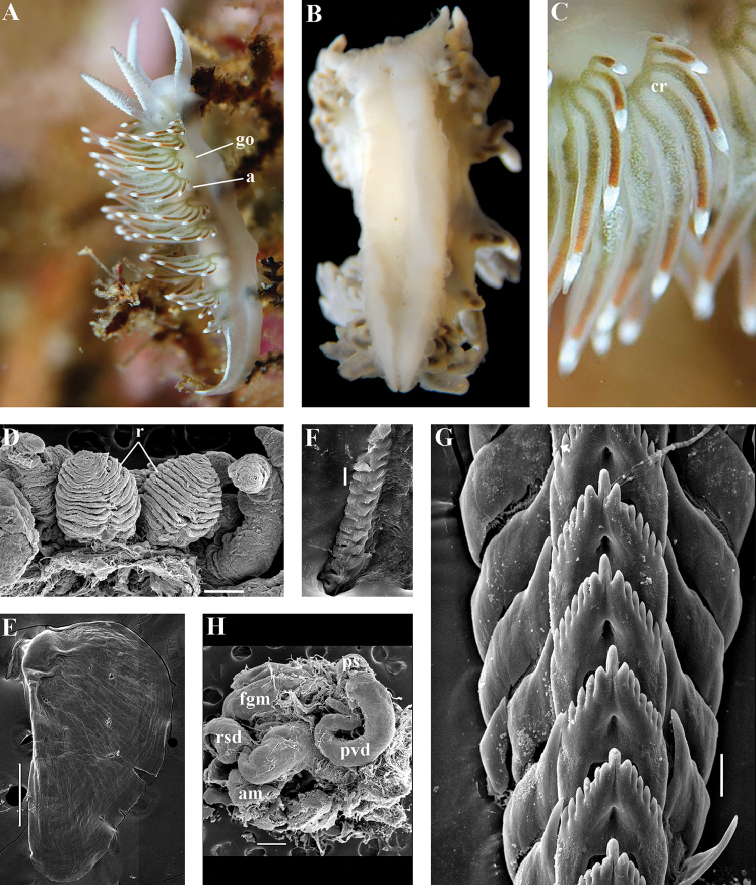
*Apata pricei komandorica* subsp. n. North West Pacific, Commander Islands. ZMMU Op-533, living specimen 8 mm in length: **A** dorso-lateral view **B** ventral view (fixed) **C** details of cerata **D** rhinophores, SEM **E** jaw, SEM **F** details of masticatory process of jaw, SEM **G** radular teeth, posterior part **H** reproductive system, SEM. Abbreviations: **a** anus **am** ampulla **cr** ceratal rows **fgm** female gland mass **go** genital opening **ps** penial sheath **pvd** prostatic vas deferens **r** rhinophores **rsd** distal receptaculum seminis. Scale bars: **D, E, H** = 300 μm; **F** = 10 μm; **G** = 30 μm. Photos of living specimens by N.P. Sanamyan other photos and SEM images by A.V. Martynov.

## Supplementary Material

XML Treatment for Paracoryphellidae

XML Treatment for Chlamylla

XML Treatment for Paracoryphella

XML Treatment for Paracoryphella ignicrystalla

XML Treatment for Polaria

XML Treatment for Ziminella

XML Treatment for Ziminella abyssa

XML Treatment for Ziminella circapolaris

XML Treatment for Baenopsis

XML Treatment for Baenopsis

XML Treatment for Flabellinopsis

XML Treatment for Coryphellidae

XML Treatment for Borealia

XML Treatment for Borealia sanamyanae

XML Treatment for Coryphella

XML Treatment for Fjordia

XML Treatment for Fjordia chriskaugei

XML Treatment for Fjordia lineata

XML Treatment for Gulenia

XML Treatment for Gulenia monicae

XML Treatment for Gulenia orjani

XML Treatment for Himatina

XML Treatment for Itaxia

XML Treatment for Microchlamylla

XML Treatment for Microchlamylla gracilis zfi

XML Treatment for Occidentella

XML Treatment for Orientella

XML Treatment for Coryphellidae

XML Treatment for Flabellinidae

XML Treatment for Calmella

XML Treatment for Carronella

XML Treatment for Carronella enne

XML Treatment for Coryphellina

XML Treatment for Coryphellina lotos

XML Treatment for Edmundsella

XML Treatment for Flabellina

XML Treatment for Paraflabellina

XML Treatment for Piseinotecus

XML Treatment for Flabellinidae

XML Treatment for Unidentiidae

XML Treatment for Pacifia

XML Treatment for Pacifia amica

XML Treatment for Unidentia

XML Treatment for Unidentia nihonrossija

XML Treatment for Unidentia sandramillenae

XML Treatment for Samlidae

XML Treatment for Luisella

XML Treatment for Samla

XML Treatment for Samla takashigei

XML Treatment for Apataidae

XML Treatment for Apata

XML Treatment for Apata pricei komandorica

XML Treatment for Kynaria

## Supplementary materials

Supplementary material 1 Data S1 Data type: Microsoft Word 97–2003 Document (.doc) Explanation note: Synopsis of the families of suborder Aeolidacea. This dataset is made available under the Open Database License (http://opendatacommons.org/licenses/odbl/1.0/). The Open Database License (ODbL) is a license agreement intended to allow users to freely share, modify, and use this Dataset while maintaining this same freedom for others, provided that the original source and author(s) are credited.Tatiana Korshunova, Alexander Martynov, Torkild Bakken, Jussi Evertsen, Karin Fletcher, I Wayan Mudianta, Hiroshi Saito, Kennet Lundin, Michael Schrödl, Bernard Picton

Supplementary material 2 Table S1 Data type: Microsoft Word 97–2003 Document (.doc) Explanation note: List of samples, localities, GenBank accession numbers, and voucher references. This dataset is made available under the Open Database License (http://opendatacommons.org/licenses/odbl/1.0/). The Open Database License (ODbL) is a license agreement intended to allow users to freely share, modify, and use this Dataset while maintaining this same freedom for others, provided that the original source and author(s) are credited.Tatiana Korshunova, Alexander Martynov, Torkild Bakken, Jussi Evertsen, Karin Fletcher, I Wayan Mudianta, Hiroshi Saito, Kennet Lundin, Michael Schrödl, Bernard Picton

Supplementary material 3 Table S2 Data type: Microsoft Word 97–2003 Document (.doc) Explanation note: Primer sequences. This dataset is made available under the Open Database License (http://opendatacommons.org/licenses/odbl/1.0/). The Open Database License (ODbL) is a license agreement intended to allow users to freely share, modify, and use this Dataset while maintaining this same freedom for others, provided that the original source and author(s) are credited.Tatiana Korshunova, Alexander Martynov, Torkild Bakken, Jussi Evertsen, Karin Fletcher, I Wayan Mudianta, Hiroshi Saito, Kennet Lundin, Michael Schrödl, Bernard Picton

Supplementary material 4 Table S3 Data type: Microsoft Excel–2003 Document (.xlsx) Explanation note: List of taxonomic changes to the traditional family Flabellinidae. This dataset is made available under the Open Database License (http://opendatacommons.org/licenses/odbl/1.0/). The Open Database License (ODbL) is a license agreement intended to allow users to freely share, modify, and use this Dataset while maintaining this same freedom for others, provided that the original source and author(s) are credited.Tatiana Korshunova, Alexander Martynov, Torkild Bakken, Jussi Evertsen, Karin Fletcher, I Wayan Mudianta, Hiroshi Saito, Kennet Lundin, Michael Schrödl, Bernard Picton
