# Corrigenda: Polyphyly of the traditional family Flabellinidae affects a major group of Nudibranchia: aeolidacean taxonomic reassessment with descriptions of several new families, genera, and species (Mollusca, Gastropoda). https://doi.org/10.3897/zookeys.717.21885

**DOI:** 10.3897/zookeys.725.23022

**Published:** 2017-12-29

**Authors:** Tatiana Korshunova, Alexander Martynov, Torkild Bakken, Jussi Evertsen, Karin Fletcher, I Wayan Mudianta, Hiroshi Saito, Kennet Lundin, Michael Schrödl, Bernard Picton

**Affiliations:** 1 Koltzov Institute of Developmental Biology, RAS, 26 Vavilova Str., 119334 Moscow, Russia; 2 Zoological Museum, Moscow State University, Bolshaya Nikitskaya Str. 6, 125009 Moscow, Russia; 3 NTNU University Museum, Norwegian University of Science and Technology, NO-7491 Trondheim, Norway; 4 Port Orchard, Washington 98366, USA; 5 Universitas Pendidikan Ganesha, Bali, 81116, Indonesia; 6 National Museum of Nature and Science, Amakubo 4-1-1, Tsukuba, Japan; 7 Gothenburg Natural History Museum, Box 7283, S-40235, Gothenburg, Sweden; 8 Gothenburg Global Biodiversity Centre, Box 461, S-40530, Gothenburg, Sweden; 9 Zoologische Staatssammlung München, Münchhausenstr. 21, D-81247 Munich, Germany; 10 Biozentrum Ludwig Maximilians University and GeoBio-Center LMU Munich, Germany; 11 National Museums Northern Ireland, Holywood, Northern Ireland, United Kingdom; 12 Queen’s University, Belfast, Northern Ireland, United Kingdom

In our recently published study the traditional Flabellinidae underwent a major revision and 17 new genera were proposed. The Abstract should read “17 new genera”.

Immediately after publication our attention was drawn to homonymy of three generic names: *Borealia*, *Occidentella*, and *Orientella* with *Borealia* Maksimova, 1977, an extinct trilobite, *Occidentella* Hoffmann, 1929, a junior synonym of *Onchidella*, a pulmonate slug, and *Orientella* Repina & Okuneva, 1969, another extinct trilobite. In a stroke of irony the two trilobite names were published in little known Russian journals, with Russian descriptions. To avoid homonymy, throughout the text, figures, and all supplementary materials replacement names for these three genera are as follows: *Borealea* Korshunova et al., nom. n., *Occidenthella* Korshunova et al., nom. n., and *Orienthella* Korshunova et al., nom. n. We are grateful to Jimmy Gaudin for alerting us to this unfortunate circumstance. Other corrections are as follows:

On p. 14 “…the same region our…” should be “…the same region as our…”

On p. 16 “…additional rows of of small…” should be “…additional rows of small…”

On p. 17 In the diagnosis of *Paracoryphella ignicrystalla* should be “…rachidian tooth with up to 15 denticles...”

On p. 18 “…cusp of nealy 1/3 of the tooth…” should be “…cusp of nearly 1/3 of the tooth…”

On p. 19 “…*Coryphella polaris* Voldochenko, 1946…” should be “…*Coryphella polaris* Volodchenko, 1946…”; “…(Figs 7, 10H, I)…” should be “…(Figs 7, 11H, I)…”; “…(Fig. 10G)…” should be “…(Fig. 11G)…”

On p. 26 Table 1. In fifth column: minimum uncorrected p-distances between *Coryphella verrucosa* and *Itaxia falklandica* should be 17.2%.

On p. 27 “…original description in Verril, 1880…” should be “…original description in Verrill, 1880…”; “…detailed redescripton in Kuzirian…” should be “…detailed redescription in Kuzirian…”

On p. 31 In the diagnosis of *Fjordia chriskaugei* should be “...orange-brown to reddish brown, sometimes blackish…”

On p. 33 In the diagnosis of *Fjordia lineata* should be: “…orange-brown to salmon, bright red, and dark brown, sometimes almost black...”

On p. 35 In the diagnosis of *Gulenia monicae* should be “...delineated from central cusp...”

On p. 37 In the diagnosis of *Gulenia orjani* should be “…delineated from central cusp...”

On p. 52 “…inclusion of *F. funeka* and *F. ishitana* is not…” should be “…inclusion of *F. funeka* and *F. ischitana* is not…”; “…and “*Piseinotecus*” *gabienerei* must be…” should be “…and “*Piseinotecus*” *gabienerei* must be…”

On p. 53 “…related to *Paraflabellina ishitana*…” should be “…related to *Paraflabellina ischitana*…”

On p. 54 “… (pleuroproctic in higher acleiproctic position)...” should be “…(pleuroproctic in higher acleioproctic position)…”

On p. 57 “…on distinct elonagate elevations…” should be “…on distinct elongate elevations...”; “… (pleuroproctic in higher cleiproctic position)…” should be “… (pleuroproctic in higher acleioproctic position)…”

On p. 58 In the diagnosis of *U. nihonrossija* should be “….apical parts of cerata without white pigment... rachidian tooth with up to seven denticles, central cusp without small denticles, double proximal receptaculum seminis…”.

On p. 66 “…due to the lack of the morphlogical description…” should be “…due to the lack of a morphological description…”

On p. 68 “…always posses oral glands…” should be “…always possess oral glands…”

On p. 74 “…reduction of the triserial radula into a unserial one…” should be “reduction of the triserial radula into a uniserial one…”

On p. 79 Figure 3. Should be *Chlamylla borealis borealis* Bergh, 1886

On p. 91 “(Cothouy, 1839)” should be “(Couthouy, 1838)”

Supplementary material 2. *Chlamylla atypica* should read *Chlamylla borealis borealis* Bergh, 1886. Localities for *Himatina trophina* (Bergh, 1890) GQ292023 and *Microchlamylla amabilis* (Hirano & Kuzirian, 1991) GQ292022 should be “WA, USA”.
