# Supplementary material for: Polyphyly of the traditional family Flabellinidae affects a major group of Nudibranchia: aeolidacean taxonomic reassessment with descriptions of several new families, genera, and species (Mollusca, Gastropoda)
Source: Zookeys. 2017 Nov 30;(717):1–139. doi: 10.3897/zookeys.717.21885 (PMC5784208; doi:10.3897/zookeys.717.21885)
Supplement: Supplementary material 1 — Data S1 [file zookeys-717-001-s001.doc]

Family Notaeolidiidae Eliot, 1910

Diagnosis. Body wide. Notal edge reduced, discontinuous. Cerata on lateral extensions, numerous per row. Ceratal rows branched. Rhinophores with folds and wrinkles. Anus pleuroproctic under notal edge. Radula formula 7-3.1.3-7. Rachidian teeth with short basis and strong cusp, not compressed by adjacent lateral denticles. Lateral teeth denticulated. Distal receptaculum seminis. Vas deferens with indistinct prostate. Massive external permanent penial collar present or absent. Penis external or internal, broad, unarmed. Supplementary (“penial”) gland absent.

Genera: *Notaeolidia* Eliot, 1905.

Family Samlidae **fam. n.**

http://zoobank.org/6366D651-3069-49A2-8B2B-140894687F1A

Diagnosis. Body narrow. Notal edges discontinuous or fully reduced. Cerata on low elevations, few per row. Ceratal rows branched. Rhinophores perfoliated. Anus pleuroproctic under the reduced notal edge or shifted towards acleioproctic position. Radula formula 1.1.1. Rachidian teeth usually with strong cusp, only rarely compressed by adjacent lateral denticles. Distal and proximal receptaculum seminis or only proximal receptaculum. Vas deferens usually short, with indistinct or distinct prostate. Massive external permanent penial collar absent. Penis internal, narrow, unarmed. Supplementary (“penial”) gland absent.

Genera: *Samla* Bergh, 1900, *Luisella* **gen. n.**

Remarks: see main text.

Family Apataidae **fam. n.**

http://zoobank.org/A0C932C0-CA54-419B-98EB-24BADFF66741

Diagnosis. Body narrow. Notum fully reduced. Cerata in separate rows, on elevations, numerous per row. Rhinophores perfoliated. Anus mixed (pleuroproctic in higher acleioproctic position). Radula formula 1.1.1. Rachidian teeth cusp compressed by adjacent lateral denticles. Lateral teeth smooth with attenuated process basally. Distal receptaculum seminis. Vas deferens moderately long, with indistinct prostate. Massive external permanent penial collar absent. Penis internal, narrow, unarmed. Supplementary (“penial”) gland absent.

Genera. *Apata* **gen. n.,** ?*Tularia* Burn, 1966

Remarks: see main text.

Family Pseudovermidae Thiele, 1931

Diagnosis. Body narrow, worm-shaped. Notum fully reduced. Cerata reduced in few separate rows. Rhinophores and oral tentacles absent. Anus acleioproctic. Radula formula 1.1.1. Rachidian teeth broad, pectinate, cusp not compressed by adjacent lateral denticles. Lateral teeth denticulated. Proximal receptaculum seminis. Vas deferens moderately long, with indistinct prostate. Massive external permanent penial collar absent. Penis internal, narrow, unarmed. Supplementary (“penial”) gland absent.

Genera. *Pseudovermis* Perejaslavtzeva, 1891.

Family Abronicidae **fam. n.**

http://zoobank.org/7BCC4848-E99E-4FA1-A61E-393A926BAE3D

Genera: *Abronica* Cella et al*.*, 2016

Diagnosis. Body narrow. Notal edges absent. Cerata non-elevated, few per row. Ceratal rows simple. Rhinophores smooth. Anus acleioproctic. Radula formula 0.1.0. Rachidian teeth usually with strong cusp not compressed by adjacent lateral denticles, elongated. Distal receptaculum seminis. Vas deferens usually short with indistinct prostate. Massive external permanent penial collar absent. Penis internal, narrow, armed. Supplementary (“penial”) gland present, inserts to penis.

Genus: *Abronica* Cella et al., 2016.

Remarks: This family apparently represents an independent case of the loss of the lateral teeth. Externally it is somewhat similar to Trinchesiidae, but according to molecular phylogenetic data the genus *Abronica* is placed basally to Eubranchidae, which all possess a triserial radula, and other family level taxa with a penial gland; therefore, the separate family Abronicidae **fam. n.** is proposed here.

Family Eubranchidae Odhner, 1934

Diagnosis. Body moderate to narrow. Notal edges fully reduced. Cerata non elevated, numerous to few per row. Ceratal rows branched or simple. Rhinophores smooth, rarely annulated or papillate. Anus acleioproctic. Radula formula 1.1.1. Rachidian teeth usually with small cusp, compressed or not compressed by adjacent lateral denticles. Lateral teeth thin, without denticles. Distal receptaculum seminis. Vas deferens usually short, with indistinct prostate. Massive external permanent penial collar absent. Penis internal, narrow, commonly armed, rarely unarmed. Special system of complex stylets present in several genera. Supplementary (“penial”) gland present, inserts to penis or rarely in vas deferens.

Genera: *Aenigmastyletus* Martynov, 1998, *Amphorina* Quatrefages, 1844, *Dunga* Eliot, 1902, *Eubranchus* Forbes, 1838, *Leostyletus* Martynov, 1998, *Nudibranchus* Martynov, 1998, *Produnga* Martynov, 1998.

Family Murmaniidae **fam. n.**

http://zoobank.org/07653D6A-337D-46E1-94D6-B6F35A8CF199

Diagnosis. Body broad. Notal edges present, continuous, reduced. Cerata non elevated, numerous per row. Ceratal rows branched. Rhinophores wrinkled. Special wedge-shaped structure between rhinophores. Anus cleioproctic or pleuroproctic. Radula formula 0.1.0. Rachidian teeth usually with strong cusp, not compressed by adjacent lateral denticles. Proximal receptaculum seminis. Vas deferens usually moderately long, with indistinct prostate. Massive external permanent penial collar absent. Penis internal, broad, unarmed. Supplementary (“penial”) gland present, inserts to penis.

Genera: *Murmania* Martynov, 2006, ?*Guyvalvoria* Vayssière, 1906.

Remarks: The genus *Murmania* is very distinct according to morphological characters in possessing a wide *Aeolidia*-like body with numerous ceratal rows but with a rudimentary notal edge and wedge-like insertion between rhinophores, similar to some *Janolus* species. The molecular data place *Murmania* basal to the Calmidae and Cuthonellidae clades. According to this consolidated morphological and molecular evidence, the new family Murmaniidae fam. nov. is therefore established herein.

Family Cuthonellidae Miller, 1971

Diagnosis. Body moderate to narrow. Notal edges fully reduced. Cerata non elevated, numerous per row. Ceratal rows branched or simple. Rhinophores smooth to wrinkled. Anus acleioproctic or cleioproctic. Radula formula 0.1.0. Rachidian teeth usually with strong cusp, not compressed by adjacent lateral denticles. Distal receptaculum seminis. Vas deferens usually moderate, with indistinct prostate. Massive external permanent penial collar absent. Penis internal, narrow, unarmed. Supplementary (“penial”) gland present, inserts to vas deferens.

Genera: *Cuthonella* Bergh, 1884, *Fiocuthona* Martynov, 1992.

Remarks: see main text.

Family Calmidae Iredale & O'Donoghue, 1923

Diagnosis. Body wide. Notal edges fully reduced. Cerata non elevated, numerous per row. Ceratal rows simple. Rhinophores smooth. Anus absent. Radula formula 0.1.0. Rachidian teeth without cusp and denticles. Distal receptaculum seminis. Vas deferens long, with indistinct prostate. Massive external permanent penial collar absent. Penis internal, narrow, unarmed. Supplementary (“penial”) gland present, inserts to vas deferens.

Genus: *Calma* Alder & Hancock, 1855.

Family Cuthonidae Odhner, 1934

Diagnosis. Body wide. Notal edges fully reduced. Cerata non elevated, numerous per row. Ceratal rows branched. Rhinophores smooth. Anus acleioproctic or cleioproctic. Radula formula 0.1.0. Rachidian teeth with strong cusp not compressed by adjacent lateral denticles. Proximal and distal receptaculum seminis. Vas deferens short, with indistinct prostate. Massive external permanent penial collar absent. Penis internal, narrow, unarmed. Supplementary (“penial”) gland present, inserts to penis.

Genera: *Cuthona* Alder & Hancock, 1855.

Remarks: see main text.

Family Tergipedidae Bergh, 1889, restricted

Diagnosis. Body narrow. Notal edges fully reduced. Cerata non elevated, single per row. Ceratal rows simple. Rhinophores smooth. Anus acleioproctic. Radula formula 0.1.0. Rachidian teeth with strong cusp not compressed by adjacent lateral denticles. Distal receptaculum seminis. Vas deferens short, with indistinct prostate. Massive external permanent penial collar absent. Penis internal, narrow, armed. Supplementary (“penial”) gland present, inserts to penis.

Genera: *Tergipes* Forsskål, 1777.

Remarks: see main text.

Family Fionidae Gray, 1857

Diagnosis. Body narrow. Notal edges present. Cerata non-elevated, numerous per row. Ceratal rows branched. Membranaceous fold on ceratal edge. Rhinophores smooth. Anus acleioproctic. Radula formula 0.1.0. Rachidian teeth usually with strong cusp not compressed by adjacent lateral denticles. Distal receptaculum seminis. Vas deferens extremely long, with indistinct prostate. Massive external permanent penial collar absent. Penis internal, extremely long, narrow, unarmed. Supplementary (“penial”) gland absent.

Genera: *Fiona* Alder & Hancock, 1853, ?*Tergiposacca* Cella et al., 2016.

Family Trinchesiidae Nordsieck, 1972

Diagnosis. Body moderate to narrow. Notal edges absent. Cerata non-elevated, numerous to few per row. Ceratal rows simple. Rhinophores smooth, rarely annulated. Anus acleioproctic. Radula formula 0.1.0. Rachidian teeth usually with strong cusp not compressed by adjacent lateral denticles, often small arc-shaped. Distal receptaculum seminis. Vas deferens usually short, with indistinct prostate. Massive external permanent penial collar absent. Penis internal, narrow, armed. Supplementary (“penial”) gland present, inserts to penis.

Genera: *Catriona* Winckworth, 1941, *Diaphoreolis* Iredale & O'Donoghue, 1923, *Phestilla* Bergh, 1874, *Tenellia* Costa, 1866, *Trinchesia* Ihering, 1879, *Zelentia* Korshunova, Martynov & Picton, 2017

Remarks: see main text.

Family Facelinidae Bergh, 1889

Diagnosis. Body moderate to narrow. Notum fully reduced. Cerata without distinct elevations, numerous per row. Ceratal rows simple, arched or branched. Rhinophores smooth, annulated, perfoliated or papillated. Anus commonly cleioproctic, rarely acleioproctic or pleuroproctic. Radula formula 0.1.0. Rachidian teeth with non-compressed cusp. Distal and proximal receptaculum seminis or only proximal receptaculum. Vas deferens moderately long, with distinct or indistinct prostate. Massive external permanent penial collar absent. Adjacent penial gland absent or present. Penis internal, narrow or broad, armed or unarmed.

Genera: *Adfacelina* Millen & Hermosillo, 2012, *Algarvia* Garcia-Gomez & Cervera, 1990, *Amanda* Macnae, 1954, *Anetarca* Gosliner, 1991, *Antonietta* Schmekel, 1966, *Austraeolis* Burn, 1962, *Bajaeolis* Gosliner & Behrens, 1986, *Burnaia* Miller, 2001, *Caloria* Trinchese, 1888, *Cratena* Bergh, 1864, *Echinopsole* Macnae, 1954, *Emarcusia* Roller, 1972, *Facelina* Alder & Hancock, 1855, *Facelinopsis* Pruvot-Fol, 1954, *Dicata* Schmekel, 1967, *Dondice* Marcus, 1958, *Godiva* Macnae, 1954, *Favorinus* Gray, 1850, *Hermissenda* Bergh, 1879, *Hermosita* Gosliner & Behrens, 1986, *Herviella* Baba, 1949, *Jason* Miller, 1974, *Learchis* Bergh, 1896, *Moridilla* Bergh, 1888, *Nanuca* Marcus, 1957, *Noumeaella* Risbec, 1937, *Palisa* Edmunds, 1964, *Pauleo* Millen & Hamann, 1992, *Phidiana* Gray, 1850, *Phyllodesmium* Ehrenberg, 1831, *Pruvotfolia* Tardy, 1969, *Pteraeolidia* Bergh, 1875, *Sakuraeolis* Baba, 1965, *Setoeolis* Baba & Hamatani, 1965.

Family Unidentiidae Millen & Hermosillo, 2012

Diagnosis. Body moderate to narrow. Notum fully reduced. Cerata non-elevated, numerous per row. Ceratal rows simple. Rhinophores smooth. Anus cleioproctic. Radula formula 0.1.0. Rachidian teeth with non-compressed cusp. Distal and proximal receptaculum seminis or only proximal receptaculum (single or double). Vas deferens moderately long, with distinct prostate. Massive external permanent penial collar absent. Adjacent penial gland absent or present. Penis internal, armed or unarmed.

Genus: *Pacifia* **gen. n.**, *Unidentia* Millen & Hermosillo, 2012.

Family Glaucidae Gray, 1827

Diagnosis. Body broad. Notum fully reduced. Cerata on extremely enlarged stalks, numerous per row. Ceratal rows simple and arched. Rhinophores smooth. Anus cleioproctic. Radula formula 0.1.0. Rachidian teeth with non-compressed cusp. Distal and proximal receptaculum seminis or only proximal receptaculum. Vas deferens moderately long, with indistinct prostate. Massive external permanent penial collar absent. Adjacent penial gland absent. Penis internal, broad, armed.

Genus: *Glaucus* Forster, 1777.

Family Pleurolidiidae Burn, 1966

Diagnosis. Body narrow. Notum present, narrow. Cerata non-elevated, numerous per row. Ceratal rows branched. Rhinophores smooth. Anus pleuroproctic. Radula formula 0.1.0. Rachidian teeth arc-shaped. Distal receptaculum seminis. Vas deferens moderately long, with indistinct prostate. Massive external permanent penial collar absent. Adjacent penial gland absent. Penis internal, narrow, unarmed.

Genera: *Pleurolidia* Burn, 1966, *Protaeolidiella* Baba, 1955.

Family Babakinidae Roller, 1973

Diagnosis. Body moderate to narrow. Notum partly reduced. Cerata non-elevated, numerous per row. Ceratal rows arched or branched. Rhinophores perfoliated. Anus pleuroproctic. Radula formula 0.1.0. Rachidian teeth with compressed cusp. Distal and proximal receptaculum seminis. Vas deferens moderately long, with distinct or indistinct prostate. Massive external permanent penial collar absent. Adjacent penial gland present. Penis internal, narrow, unarmed.

Genus: *Babakina* Roller, 1973.

Family Aeolidiidae Gray, 1827

Diagnosis. Body broad to narrow. Notum absent in majority of genera. Cerata non-elevated, numerous per row. Rhinophores smooth, perfoliated or papillated. Ceratal rows simple or arched. Anus commonly cleioproctic, rarely pleuroproctic. Radula formula 0.1.0. Rachidian teeth arc-shaped. Vas deferens moderately long, with indistinct prostate. Massive external permanent penial collar absent. Adjacent penial gland absent. Penis internal, narrow or broad, unarmed.

Genera: *Aeolidia* Cuvier, 1798, *Aeolidiella* Bergh, 1867, *Anteaeolidiella* M. C. Miller, 2001, *Baeolidia* Bergh, 1888, *Berghia* Trinchese, 1877, *Bulbaeolidia* Carmona, Pola, Gosliner & Cervera, 2013, *Cerberilla* Bergh, 1873, *Limenandra* Haefelfinger & Stamm, 1958, *Spurilla* Bergh, 1864, *Zeusia* Korshunova, Zimina & Martynov, 2017.

Family Cumanotidae Odhner, 1907

Diagnosis. Body moderate. Notal edge absent. Cerata on elevations, numerous per row. Ceratal rows branched. Rhinophores smooth. Anus pleuroproctic. Radula formula 1.1.1. Rachidian teeth usually with compressed cusp by adjacent lateral denticles. Lateral teeth narrow or with attenuated process basally, usually denticulated, rarely smooth. Distal receptaculum seminis. Vas deferens long, with distinct or indistinct prostate. Special grasping organ in female part of reproductive system with numerous stylets. Penis internal, narrow, enrolled, armed or unarmed. Supplementary (“penial”) gland absent.

Genera: *Cumanotus* Odhner, 1907.

Family Paracoryphellidae Miller, 1971, reinstated

Diagnosis. Body wide. Notal edge continuous. Cerata non-elevated, numerous per row. Ceratal rows branched. Rhinophores smooth to wrinkled. Anus pleuroproctic under the notal edge. Radula formula 1.1.1. Rachidian teeth with long basis and strong cusp, not compressed by adjacent lateral denticles. Lateral teeth narrow or rarely with attenuated process basally, usually denticulated. Commonly only single distal receptaculum seminis present. Vas deferens with distinct or indistinct prostate. Massive external permanent penial collar present or absent. Penis external or internal, narrow, unarmed. Supplementary (“penial”) gland absent.

Genera: *Chlamylla* Bergh, 1886, *Paracoryphella* Miller, 1971, *Polaria* **gen. n.**, *Ziminella* **gen. n.**

Remarks: see main text.

Family Coryphellidae Bergh, 1889, reinstated

Diagnosis. Body wide or narrow. Notal edge continuous or discontinuous. Cerata non elevated, numerous per row. Ceratal rows branched. Rhinophores smooth, wrinkled, rarely annulated or perfoliated. Anus pleuroproctic. Radula formula 1.1.1. Rachidian teeth with long basis and strong cusp, usually not compressed by adjacent lateral denticles. Lateral teeth

narrow or with attenuated process basally, always denticulated. Vas deferens with indistinct prostate. Massive external permanent penial collar absent. Penis internal, commonly broad, unarmed. Supplementary (“penial”) gland absent.

Genera: Genera: *Borealia* **gen. n.**, *Coryphella* Gray, 1850, *Fjordia* **gen. n.**, *Gulenia* **gen. n.***, Himatina* Thiele, 1931, *Itaxia* **gen. n.**, *Microchlamylla* **gen. n.**, *Occidentella* **gen. n.**, *Orientella* **gen. n.**

Remarks: see main text.

Family Flabellinopsidae **fam. n.**

<http://zoobank.org/>F3E7E3B7-E77F-484A-B629-FFDDA9FDCD59

Diagnosis. Body relatively wide. Notal edge discontinuous. Cerata on broad extensions, numerous per row. Ceratal rows branched. Rhinophores perfoliated or granulated. Anus pleuroproctic. Radula formula 1.1.1. Rachidian teeth usually with cusp compressed by adjacent lateral denticles. Lateral teeth narrow or with attenuated process basally, denticulated or smooth. Distal receptaculum seminis. Vas deferens long, with distinct or indistinct prostate. Massive external permanent penial collar absent. Penis internal, unarmed. Supplementary (“penial”) gland absent.

Genera: *Flabellinopsis* MacFarland, 1966; *Baenopsis* **gen. n.**

Remarks: see main text.

Family Flabellinidae Bergh, 1889, restricted

Diagnosis. Body narrow. Notal edge discontinuous or fully reduced. Cerata on elevations or stalks, numerous to few per row. Ceratal rows simple. Rhinophores smooth, annulated, or papillated. Anus pleuroproctic or shifted towards acleioproctic. Radula formula 1.1.1. Rachidian teeth with long basis and small cusp compressed by adjacent lateral denticles. Lateral teeth with attenuated process basally, usually denticulated, rarely smooth. Vas deferens with indistinct prostate. Massive external permanent penial collar absent. Penis internal, commonly narrow, unarmed. Supplementary (“penial”) gland absent.

Genera: *Calmella* Eliot, 1910, *Carronella* **gen. n.**, *Coryphellina* O’Donoghue, 1929, *Edmundsella* **gen. n.**, *Flabellina* Gray, 1833 in Griffith et Pidgeon, 1833-1834, *Paraflabellina* **gen. n.**

Remarks: see main text.
