# Supplementary material for: Polyphyly of the traditional family Flabellinidae affects a major group of Nudibranchia: aeolidacean taxonomic reassessment with descriptions of several new families, genera, and species (Mollusca, Gastropoda)
Source: Zookeys. 2017 Nov 30;(717):1–139. doi: 10.3897/zookeys.717.21885 (PMC5784208; doi:10.3897/zookeys.717.21885)
Supplement: Supplementary material 2 — Table S1 [file zookeys-717-001-s002.doc]

| **Table S1.**  **List of samples, localities, GenBank**  **accession numbers and voucher references** | | | | | | |
| --- | --- | --- | --- | --- | --- | --- |
| **Species name** | **Voucher** | **Locality** | **COI** | **16S** | **H3** | **28S** |
| *Abronica abronia* (MacFarland, 1966) | CAS181319 | California | KY128919 | KY128716 | KY128508 | - |
| *Aeolidia papillosa* (Linnaeus, 1761) | ZMMU:Op-558 | Russia,  Barents Sea | KX758258 | KX758253 | KX758262 | - |
| *Aeolidia loui* Kienberger, Carmona, Pola, Padula, Gosliner & Cervera, 2016 | CAS173369 | California | KY128974 | KY128766 | KY128561 | - |
| *Aeolidiella alderi* (Cocks, 1852) | ZSMMol20012341 | Italy | HQ616766 | HQ616729 | HQ616795 | - |
| *Amphorina odhneri* (Derjugin & Gurjanova, 1926) | ZMMU:Op-484 | Russia,  Barents Sea | **MF523318** | **MF523396** | **MF523244** | **MF523472** |
| *Apata pricei komandorica* subsp. n. | ZMMU:Op-533 | Russia, Commander Ids | **MF523386** | **MF523413** | **MF523311** | - |
| *Apata* cf*. pricei* (MacFarland, 1966) | CAS114776 | California | KY129060 | KY128851 | KY128645 | - |
| *Apata pricei pricei* (MacFarland, 1966) | CAS181322 | California | KY129052 | KY128843 | KY128637 | - |
| *Chlamylla atypica* (Bergh, 1899) | ZMMU:Op-487 | Russia,  Kara Sea | **MF523321** | **MF523395** | **MF523246** | **MF523471** |
| *Chlamylla intermedia* (Bergh, 1899) | ZMMU:Op-488 | Russia,  White Sea | **MF523322** | **MF523392** | - | **MF523468** |
| *Chlamylla intermedia* (Bergh, 1899) | ZMMU:Op-489 | Russia,  White Sea | **MF523323** | **MF523393** | - | **MF523469** |
| *Chlamylla intermedia* (Bergh, 1899) | ZMMU:Op-493 | Russia,  White Sea | **MF523324** | **MF523415** | **MF523247** | - |
| *Chlamylla intermedia* (Bergh, 1899) | ZMMU:Op-494 | Russia,  Laptev Sea | **MF523325** | **MF523418** | **MF523248** | **MF523492** |
| *Chlamylla intermedia* (Bergh, 1899) | ZMMU:Op-495 | Russia,  Laptev Sea | **MF523326** | **MF523447** | **MF523249** | **MF523514** |
| *Chlamylla intermedia* (Bergh, 1899) | ZMMU:Op-480 | Russia,  White Sea | **MF523327** | **MF523450** | **MF523250** | **MF523518** |
| *Chlamylla intermedia* (Bergh, 1899) | ZMMU:Op-481 | Russia,  Laptev Sea | **MF523328** | **MF523451** | **MF523251** | **MF523519** |
| *Chlamylla intermedia* (Bergh, 1899) | ZMMU:Op-496 | Russia,  Laptev Sea | **MF523329** | **MF523452** | **MF523252** | **MF523520** |
| *Chlamylla intermedia* (Bergh, 1899) | ZMMU:Op-497 | Russia,  Laptev Sea | **MF523330** | **MF523453** | **MF523253** | - |
| *Babakina anadoni* (Ortea, 1979) | MNCN15.05/46704 | Spain | HQ616767 | HQ616730 | HQ616796 | - |
| *Babakina indopacifica* Gosliner, Gonzalez-Duarte & Cervera, 2007 | CASIZ177458 | Philippines | HM162754 | HM162678 | HM162587 | - |
| *Baenopsis baetica* (Garcia-Gomez, 1984) | MNCN15.05/53699 | Spain | HQ616755 | HQ616718 | HQ616784 | - |
| *Bonisa nakaza* Gosliner, 1981 | CASIZ176146 | South Africa | HM162746 | HM162670 | HM162579 | - |
| *Borealia nobilis* (Verrill, 1880) | ZMMU:Op-510 | Russia,  White Sea | **MF523347** | **MF523411** | **MF523271** | **MF523487** |
| *Borealia nobilis* (Verrill, 1880) | ZMMU:Op-511 | Russia,  White Sea | **MF523348** | - | **MF523272** | **MF523516** |
| *Borealia nobilis* (Verrill, 1880) | NTNU-VM:66923 | Norway,  Svalbard | - | **MG452650** | **MG452568** | - |
| *Borealia nobilis* (Verrill, 1880) | NTNU-VM:66922 | Norway,  Svalbard | - | **MG452652** | **MG452570** | - |
| *Borealia sanamyanae* sp. n. | ZMMU:Op-518 | Russia,  Matua Island | - | **MF523461** | **MF523298** | **MF523527** |
| *Calma glaucoides* (Alder & Hancock, 1854) | CPIC00562 | - | JQ699567 | JQ699477 | JQ699388 | - |
| *Calmella cavolini* (Vérany, 1846) | ZMMU:Op-485 | France,  Banyuls-sur-Mer | MF523319 | MF523448 | MF523245 | MF523515 |
| *Calmella cavolini* (Vérany, 1846) | MNCN15.05/53688 | Italy | HQ616772 | HQ616737 | - | - |
| *Calmella gaditana*  Cervera, García-Gómez & García, 1987 | MCNCN/ADN51998 | France | JX087557 | JX087491 | JX087627 | - |
| *Calmella gaditana* Cervera, García-Gómez & García, 1987 | MNCN15.05/53704 | Spain | HQ616759 | HQ616722 | HQ616788 | - |
| *Calmella confusa* Gonzalez-Duarte, Cervera & Poddubetskaia, 2008 | MCNCN/ADN51999 | France | JX087556 | JX087490 | JX087626 | - |
| *Carronella enne* sp. n. | ZMMU:Op-526 | East Atlantic | **MF523382** | **MF523439** | **MF523307** | **MF523506** |
| *Carronella pellucida* (Alder & Hancock, 1843) | ZMMU:Op-514 | Norway,  Gulen | **MF523349** | **MF523434** | **MF523273** | **MF523501** |
| *Carronella pellucida* (Alder & Hancock, 1843) | ZMMU:Op-513 | Norway,  Gulen | **MF523350** | **MF523435** | **MF523274** | **MF523502** |
| *Carronella pellucida* (Alder & Hancock, 1843) | NTNU-VM:65478 | Norway,  Frøya | - | **MG452639** | **MG452556** | - |
| *Carronella pellucida* (Alder & Hancock, 1843) | NTNU-VM:68824 | Norway,  Gulen | **MG452617** | **MG452667** | **MG452586** | - |
| *Carronella pellucida* (Alder & Hancock, 1843) | NTNU-VM:68823 | Norway,  Gulen | **MG452618** | **MG452668** | **MG452587** | - |
| *Catriona aurantia* (Alder & Hancock, 1842) | ZMMU:Op-545 | Norway,  Gulen | KY985467 | **MF523458** | - | **MF523524** |
| *Coryphella pseudoverrucosa* Martynov, Sanamyan & Korshunova, 2015 | ZMMU:Op-527 | Russia,  Kamchatka | **MF523351** | - | **MF523275** | **MF523517** |
| *Coryphella pseudoverrucosa* Martynov, Sanamyan & Korshunova, 2015 | ZMMU:Op-528 | Russia,  Kamchatka | **MF523352** | **MF523454** | **MF523276** | **MF523521** |
| *Coryphella pseudoverrucosa* Martynov, Sanamyan & Korshunova, 2015 | ZMMU:Op-529 | Russia,  Kamchatka | **MF523353** | **MF523456** | **MF523277** | **MF523522** |
| *Coryphella verrucosa* (Sars M., 1829) | ZMMU:Op-520 | Russia,  White Sea | **MF523374** | **MF523412** | **MF523299** | **MF523488** |
| *Coryphella verrucosa* (Sars M., 1829) | ZMMU:Op-521 | Russia,  Barents Sea | **MF523375** | **MF523421** | **MF523300** | **MF523494** |
| *Coryphella verrucosa* (Sars M., 1829) | CAS183939 | Maine | KY129065 | KY128856 | KY128650 | - |
| *Coryphella verrucosa* (Sars M., 1829) | PPM197 | Canada | KF643409 | - | - | - |
| *Coryphella verrucosa* (Sars M., 1829) | NTNU-VM:62691 | Norway,  Frøya | HM425522 | **MG452636** | **MG452553** | - |
| *Coryphella verrucosa* (Sars M., 1829) | NTNU-VM:62688 | Norway,  Frøya | HM425523 | **MG452637** | **MG452554** | - |
| *Coryphella verrucosa* (Sars M., 1829) | NTNU-VM:62693 | Norway,  Moss | - | **MG452638** | **MG452555** | - |
| *Coryphella verrucosa* (Sars M., 1829) | NTNU-VM:68808 | Norway,  Gulen | **MG452614** | **MG452664** | **MG452583** | - |
| *Coryphella verrucosa* (Sars M., 1829) | NTNU-VM:68810 | Norway,  Gulen | **MG452615** | **MG452665** | **MG452584** | - |
| *Coryphella verrucosa* (Sars M., 1829) | NTNU-VM:68809 | Norway,  Gulen | **MG452616** | **MG452666** | **MG452585** | - |
| *Coryphella verrucosa* (Sars M., 1829) | NTNU-VM:68826 | Norway,  Gulen | **MG452623** | **MG452673** | **MG452592** | - |
| *Coryphella verrucosa* (Sars M., 1829) | NTNU-VM:68825 | Norway,  Gulen | **MG452624** | **MG452674** | **MG452593** | - |
| *Coryphella verrucosa* (Sars M., 1829) | NTNU-VM:68827 | Norway,  Gulen | **MG452625** | **MG452675** | **MG452594** | - |
| *Coryphellina arveloi* (Ortea & Espinosa, 1998) | CAS179418 | Sao Tome and Principe | KY129048 | KY128839 | KY128633 | - |
| *Coryphellina arveloi* (Ortea & Espinosa, 1998) | CAS179419 | Sao Tome and Principe | KY129049 | KY128840 | KY128634 | - |
| *Coryphellina lotos* sp. n. | ZMMU:Op-515 | Japan,  Izu Peninsula | **MF523387** | **MF523462** | **MF523312** | **MF523528** |
| *Coryphellina exoptata* (Gosliner & Willan, 1991) | CAS178322 | Malaysia | KY129053 | KY128844 | KY128638 | - |
| *Coryphellina exoptata* (Gosliner & Willan, 1991) | ZMMU Op-116 | Vietnam,  Nha Trang | **MF523380** | **MF523438** | **MF523305** | **MF523505** |
| *Coryphellina rubrolineata* (O'Donoghue, 1929) | CAS177287 | Philippines | KY129061 | KY128852 | KY128646 | - |
| *Coryphellina rubrolineata* (O'Donoghue, 1929) | ZMMU:Op-132 | Vietnam | **MF523381** | **MF523437** | **MF523306** | **MF523504** |
| *Cuthona nana* (Alder & Hancock, 1842) | ZMMU:Op-522 | Russia,  Barents Sea | **MF523376** | **MF523397** | **MF523301** | **MF523473** |
| *Cuthonella cocoachroma* (Williams & Gosliner, 1979) | CAS181307b | California | KY128927 | KY128722 | KY128515 | - |
| *Cuthonella concinna* (Alder & Hancock, 1843) | ZMMU:Op-523 | Russia,  White Sea | **MF523377** | **MF523459** | **MF523302** | **MF523525** |
| *Cuthonella soboli* Martynov, 1992 | ZMMU:Op-524 | Russia,  Japan Sea | **MF523378** | **MF523457** | **MF523303** | **MF523523** |
| *Diaphoreolis lagunae* (O'Donoghue, 1926) | CAS179465a | California | KY128956 | KY128749 | KY128543 | - |
| *Diaphoreolis viridis* (Forbes, 1840) | ZMMU:Op-537 | Russia,  White Sea | MG266028 | MG266026 | MG266029 | MG266027 |
| *Edmundsella*  *albomaculata* (Pola, Carmona, Calado & Cervera, 2014) | MNCN:15.05/60115 | Cape Verde | KJ721521 | - | KJ721523 | - |
| *Edmundsella*  *albomaculata* ( Pola, Carmona, Calado & Cervera, 2014) | MNCN:15.05/69896 | Cape Verde, Sao Vicente Island | KJ721522 | KJ721520 | KJ721524 | - |
| *Edmundsella pedata* (Montagu, 1815) | MT09669 | North Sea | KR084950 | - | - | - |
| *Edmundsella pedata* (Montagu, 1815) | MNCN15.05/53702 | Spain,  Malaga | HQ616758 | HQ616721 | HQ616787 | - |
| *Edmundsella pedata* (Montagu, 1815) | NTNU-VM:65502 | Norway,  Hambåra | HM425421 | - | **MG452564** | - |
| *Edmundsella pedata* (Montagu, 1815) | NTNU-VM: 65498 | Norway,  Ørland | **MG452603** | **MG452648** | **MG452566** | - |
| *Edmundsella pedata* (Montagu, 1815) | NTNU-VM: 68819 | Norway,  Gulen | **MG452607** | **MG452657** | **MG452576** | - |
| *Eubranchus pallidus* (Alder & Hancock, 1842) | GNM9094 | Scotland | KY129030 | KY128821 | KY128616 | - |
| *Eubranchus tricolor* Forbes, 1838 | ZMMU:Op-525 | Norway,  Gulen | **MF523379** | **MF523399** | **MF523304** | **MF523475** |
| *Facelina bostoniensis* (Couthouy, 1838) | CAS184184 | New Hampshire | KY129046 | KY128837 | KY128632 | - |
| *Favorinus branchialis* (Rathke, 1806) | MNCN15.05/53695 | Spain | HQ616761 | HQ616724 | HQ616790 | - |
| *Favorinus* sp. | CASIZ 181353 | Marshall Islands | JX220463 | JX220495 | JX220431 | - |
| *Fiona pinnata* (Eschscholtz, 1831) | MCNCN/ADN51997 | Morocco | JX087558 | JX087492 | JX087628 | - |
| *Fjordia browni*  (Picton, 1980) | ZMMU:Op-413 | Norway,  Gulen | **MF523333** | **MF523423** | **MF523257** | **MF523496** |
| *Fjordia browni* (Picton, 1980) | NTNU-VM:65462 | Norway, Sållåtneset | HM425442 | **MG452647** | **MG452565** | - |
| *Fjordia browni* (Picton, 1980) | NTNU-VM:65465 | Ireland | HM425458 | **MG452649** | **MG452567** | - |
| *Fjordia browni* (Picton, 1980) | NTNU-VM: 68801 | Norway,  Gulen | **MG452619** | **MG452669** | **MG452588** | - |
| *Fjordia browni* (Picton, 1980) | NTNU-VM: 68800 | Norway,  Gulen | **MG452620** | **MG452670** | **MG452589** | - |
| *Fjordia browni* (Picton, 1980) | NTNU-VM:68802 | Norway,  Gulen | **MG452621** | **MG452671** | **MG452590** | - |
| *Fjordia browni* (Picton, 1980) | NTNU-VM:68803 | Norway,  Gulen | **MG452622** | **MG452672** | **MG452591** | - |
| *Fjordia browni* (Picton, 1980) | NTNU-VM:68822 | Norway,  Gulen | **MG452629** | **MG452679** | **MG452598** | - |
| *Fjordia browni* (Picton, 1980) | NTNU-VM:68821 | Norway,  Gulen | **MG452630** | **MG452680** | **MG452599** | - |
| *Fjordia chriskaugei*  sp. n. | NTNU-VM:65505 | Norway,  Frøya | **HM425396** | **MG452641** | **MG452558** | - |
| *Fjordia chriskaugei* sp. n. | NTNU-VM:65509 | Norway, Drøbaksundet | HM425416 | **MG452643** | **MG452560** | - |
| *Fjordia chriskaugei*  sp. n. | NTNU-VM:65508 | Norway, Drøbaksundet | HM425417 | **MG452644** | **MG452561** | - |
| *Fjordia chriskaugei*  sp. n. | NTNU-VM:65506 | Norway,  Hambåra | HM425418 | **MG452645** | **MG452562** | - |
| *Fjordia chriskaugei*  sp. n. | NTNU-VM:67131 | Norway,  Gulen | **MG452606** | **MG452656** | **MG452575** | - |
| *Fjordia chriskaugei*  sp. n. | NTNU-VM:68806 | Norway,  Gulen | **MG452610** | **MG452660** | **MG452579** | - |
| *Fjordia chriskaugei*  sp. n. | NTNU-VM:68807 | Norway,  Gulen | **MG452611** | **MG452661** | **MG452580** | - |
| *Fjordia chriskaugei*  sp. n. | NTNU-VM:68805 | Norway,  Gulen | **MG452612** | **MG452662** | **MG452581** | - |
| *Fjordia chriskaugei*  sp. n. | NTNU-VM:68804 | Norway,  Gulen | **MG452613** | **MG452663** | **MG452582** | - |
| *Fjordia chriskaugei*  sp. n. | ZMMU:Op-542 | Norway,  Gulen | **MF523355** | **MF523405** | **MF523279** | **MF523481** |
| *Fjordia chriskaugei*  sp. n. | ZMMU: Op-477 | Norway,  Gulen | **MF523356** | **MF523406** | **MF523280** | **MF523482** |
| *Fjordia chriskaugei*  sp. n. | ZMMU: Op-499 | Norway,  Gulen | **MF523357** | **MF523417** | **MF523281** | **MF523491** |
| *Fjordia lineata* (Lovén, 1846) | NTNU-VM:65504 | Norway,  Frøya | HM425389 | **MG452640** | **MG452557** | - |
| *Fjordia lineata* (Lovén, 1846) | NTNU-VM:68815 | Norway,  Gulen | **MG452608** | **MG452658** | **MG452577** | - |
| *Fjordia lineata* (Lovén, 1846) | ZMMU:Op-506 | Norway,  Gulen | **MF523343** | **MF523402** | **MF523267** | **MF523478** |
| *Fjordia lineata* (Lovén, 1846) | ZMMU:Op-507 | Norway,  Gulen | **MF523344** | **MF523403** | **MF523268** | **MF523479** |
| *Fjordia lineata* (Lovén, 1846) | ZMMU:Op-508 | Norway,  Gulen | **MF523345** | **MF523404** | **MF523269** | **MF523480** |
| *Fjordia lineata* (Lovén, 1846) | NTNU-VM: 72483 | Sweden,  Oslo Fjord | **MF523346** | **MF523422** | **MF523270** | **MF523495** |
| *Flabellina affinis* (Gmelin, 1791) | - | Spain, Mediterranean Sea | AF249783 | - | - | - |
| *Flabellina affinis* (Gmelin, 1791) | - | Murcia,  SE Spain | AY345055 | - | - | - |
| *Flabellina affinis* (Gmelin, 1791) | MNCN15.05/53696 | Spain | HQ616753 | HQ616716 | HQ616782 | - |
| *Flabellinopsis iodinea* (Cooper, 1863) | CAS181313a | California | KY129056 | KY128847 | KY128641 | - |
| *Flabellinopsis iodinea* (Cooper, 1863) | CAS181313b | California | KY129057 | KY128848 | KY128642 | - |
| *Flabellinopsis iodinea* (Cooper, 1863) | CAS181313c | California | KY129058 | KY128849 | KY128643 | - |
| *Gulenia borealis* (Odhner, 1922) | GNM9417 | Norway | **MG452631** | **MG452682** | **MG452600** | **MG452635** |
| *Gulenia borealis* (Odhner, 1922) | MT09694 | North Sea | KR084727 | - | - | - |
| *Gulenia borealis* (Odhner, 1922) | MT09704 | North Sea | KR084774 | - | - | - |
| *Gulenia borealis* (Odhner, 1922) | MT09705 | North Sea | KR084492 | - | - | - |
| *Gulenia borealis* (Odhner, 1922) | MT09706 | North Sea | KR084560 | - | - | - |
| *Gulenia monicae* sp. n. | ZMMU:Op-466 | Norway,  Gulen | **MF523369** | **MF523408** | **MF523293** | **MF523484** |
| *Gulenia monicae* sp. n. | ZMMU:Op-411 | Norway,  Gulen | **MF523370** | **MF523410** | **MF523294** | **MF523486** |
| *Gulenia monicae* sp. n. | ZMMU:Op-475 | Norway,  Gulen | **MF523371** | **MF523425** | **MF523295** | **MF523498** |
| *Gulenia monicae* sp. n. | ZMMU:Op-476 | Norway,  Gulen | **MF523372** | **MF523429** | **MF523296** | **MF523500** |
| *Gulenia monicae* sp. n. | ZMMU:Op-408 | Norway,  Gulen | **MF523373** | **MF523441** | **MF523297** | **MF523508** |
| *Gulenia monicae* sp. n. | NTNU-VM:68816 | Norway,  Gulen | **MG452609** | **MG452659** | **MG452578** | - |
| *Gulenia orjani*  sp. n. | NTNU-VM: 72482 | Norway,  Gulen | **MF523358** | **MF523407** | **MF523282** | **MF523483** |
| *Gulenia orjani*  sp. n. | ZMMU:Op-409 | Norway,  Gulen | **MF523359** | **MF523409** | **MF523283** | **MF523485** |
| *Gulenia orjani* sp. n. | ZMMU:Op-538 | Norway,  Gulen | **MF523360** | **MF523416** | **MF523284** | **MF523490** |
| *Gulenia orjani* sp. n. | ZMMU:Op-467 | Norway,  Gulen | **MF523361** | **MF523424** | **MF523285** | **MF523497** |
| *Gulenia orjani* sp. n. | ZMMU:Op-468 | Norway,  Gulen | **MF523362** | **MF523426** | **MF523286** | **MF523499** |
| *Gulenia orjani* sp. n. | ZMMU:Op-473 | Norway,  Gulen | **MF523363** | **MF523427** | **MF523287** | - |
| *Gulenia orjani* sp. n. | ZMMU:Op-469 | Norway,  Gulen | **MF523364** | **MF523428** | **MF523288** | - |
| *Gulenia orjani* sp. n. | ZMMU:Op-474 | Norway,  Gulen | **MF523365** | **MF523430** | **MF523289** | - |
| *Gulenia orjani* sp. n. | ZMMU:Op-470 | Norway,  Gulen | **MF523366** | **MF523431** | **MF523290** | - |
| *Gulenia orjani* sp. n. | ZMMU:Op-471 | Norway,  Gulen | **MF523367** | **MF523432** | **MF523291** | - |
| *Gulenia orjani* sp. n. | ZMMU:Op-472 | Norway,  Gulen | **MF523368** | **MF523433** | **MF523292** | - |
| *Gulenia orjani* sp. n. | NTNU-VM:65507 | Norway,  Hambåra | HM425419 | **MG452646** | **MG452563** | - |
| *Gulenia orjani*  sp. n. | NTNU-VM:67090 | Norway,  Gulen | - | **MG452654** | **MG452572** | - |
| *Gulenia orjani*  sp. n. | NTNU-VM:67091 | Norway,  Gulen | **MG452604** | **MG452655** | **MG452573** | - |
| *Himatina trophina* (Bergh, 1890) | - | Ross Sea | GQ292023 | - | - | - |
| *Himatina trophina* (Bergh, 1890) | ZMMU:Op-531 | Russia,  Kamchatka | **MF523388** | **MF523440** | **MF523313** | **MF523507** |
| *Himatina trophina* (Bergh, 1890) | ZMMU:Op-532 | Russia,  Kamchatka | **MF523389** | **MF523460** | **MF523314** | **MF523526** |
| *Himatina trophina* (Bergh, 1890) | LACM172497 | - | JQ699573 | JQ699486 | JQ699398 | - |
| *Itaxia falklandica* (Eliot, 1907) | ZSM Mol-20070592 | Chile | **MF523334** | **MF523467** | **MF523258** | **MF523530** |
| *Janolus longidentatus* Gosliner, 1981 | CASIZ176320 | South Africa | HM162749 | HM162673 | HM162582 | - |
| *Luisella babai* (Schmekel, 1972) | MNCN15.05/53698 | Spain | HQ616783 | HQ616754 | HQ616717 | - |
| *Microchlamylla amabilis* (Hirano & Kuzirian, 1991) | - | Ross Sea | GQ292022 | - | - | - |
| *Microchlamylla gracilis*  *gracilis* (Alder & Hancock, 1844) | ZMMU:Op-500 | Russia,  Barents Sea | **MF523335** | **MF523420** | **MF523259** | **MF523493** |
| *Microchlamylla gracilis* (Alder & Hancock, 1844) | ZMMU:Op-501 | Russia,  Franz Josef Land | **MF523336** | **MF523442** | **MF523260** | **MF523509** |
| *Microchlamylla gracilis* (Alder & Hancock, 1844) | ZMMU:Op-502 | Norway,  Gulen | **MF523337** | **MF523443** | **MF523261** | **MF523510** |
| *Microchlamylla gracilis* (Alder & Hancock, 1844) | ZMMU:Op-503 | Norway,  Gulen | **MF523338** | **MF523444** | **MF523262** | **MF523511** |
| *Microchlamylla gracilis* (Alder & Hancock, 1844) | ZMMU:Op-504 | Russia,  Barents Sea | **MF523339** | **MF523445** | **MF523263** | **MF523512** |
| *Microchlamylla gracilis* (Alder & Hancock, 1844) | ZMMU:Op-505 | Russia,  Barents Sea | **MF523340** | **MF523446** | **MF523264** | **MF523513** |
| *Microchlamylla gracilis* (Alder & Hancock, 1844) | NTNU-VM:65480 | Norway, Drøbaksundet | HM425415 | **MG452642** | **MG452559** | - |
| *Microchlamylla gracilis* (Alder & Hancock, 1844) | NTNU-VM:67140 | Norway, Trondheim | **MG452605** | - | **MG452574** | - |
| *Microchlamylla gracilis* (Alder & Hancock, 1844) | NTNU-VM:68812 | Norway,  Gulen | **MG452626** | **MG452676** | **MG452595** | - |
| *Microchlamylla gracilis* (Alder & Hancock, 1844) | NTNU-VM:68813 | Norway,  Gulen | **MG452627** | **MG452677** | **MG452596** | - |
| *Microchlamylla gracilis* (Alder & Hancock, 1844) | NTNU-VM:68811 | Norway,  Gulen | **MG452628** | **MG452678** | **MG452597** | - |
| *Microchlamylla gracilis* (Alder & Hancock, 1844) | CAS183938 | Maine | KY129055 | KY128846 | KY128640 | - |
| *Murmania antiqua* Martynov, 2006 | ZMMU:Op-399 | Russia,  Kara Sea | **MF523390** | **MF523394** | **MF523315** | **MF523470** |
| *Occidentella athadona* (Bergh, 1875) | ZMMU:Op-498 | Russia,  Kamchatka | **MF523332** | **MF523414** | **MF523256** | **MF523489** |
| *Occidentella athadona* (Bergh, 1875) | - | South Korea | KT724949 | - | - | - |
| *Orientella trilineata* (O'Donoghue, 1921) | CAS179466 | California | KY129064 | KY128855 | KY128649 | - |
| *Pacifia goddardi* Gosliner, 2010 | CAS182590 | California | KY129063 | KY128854 | KY128648 | - |
| *Pacifia amica* gen. et sp. n. | ZMMU:Op-614 | USA | **MG452633** | **-** | **MG452602** | - |
| *Paracoryphella islandica* (Odhner, 1937) | ZMMU:Op-534 | Russia,  Barents Sea | MF523391 | MF523398 | MF523316 | MF523474 |
| *Paraflabellina ischitana* (Hirano & Thompson, 1990) | MNCN15.05/53700 | Morocco | HQ616756 | HQ616719 | HQ616785 | - |
| *Paraflabellina ischitana* (Hirano & Thompson, 1990) | MNCN15.05/53697 | Spain | HQ616757 | HQ616720 | HQ616786 | - |
| *Paraflabellina ischitana* (Hirano & Thompson, 1990) | MNCN15.05/53701 | Spain | - | HQ616745 | HQ616808 | - |
| *Paraflabellina funeka* (Gosliner & Griffiths, 1981) | CAS176374 | South Africa | KY129054 | KY128845 | KY128639 | - |
| *Paraflabellina gabinierei* (Vicente, 1975) | MNCN/ADN52000 | Spain | - | JX087495 | JX087631 | - |
| *Paraflabellina gabinierei* (Vicente, 1975) | MNCN/ADN52001 | Spain | - | JX087496 | JX087632 | - |
| *Polaria polaris* (Volodchenko, 1946) | ZMMU:Op-519  clone1 | Russia,  Laptev Sea | **MF523331** | **MF523401** | **MF523254** | **MF523477** |
| *Polaria polaris* (Volodchenko, 1946) | ZMMU:Op-519  clone2 | Russia,  Laptev Sea | - | **MF523455** | **MF523255** | - |
| *“Piseinotecus”* sp. | CASIZ177740 | Philippines | HM162694 | HM162604 | HM162510 | - |
| *Phestilla lugubris* (Bergh, 1870) | CAS177437 | Philippines | KY129075 | KY128866 | KY128660 | - |
| *Phestilla melanobrachia* Bergh, 1874 | CAS177299 | Philippines | KY129079 | KY128870 | KY128664 | - |
| *Protaeolidiella atra* Baba, 1955 | NSMT-Mo78852 | Japan | KP143675 | KP143671 | KP143679 | - |
| *Rubramoena amoena* (Alder & Hancock, 1845) | GNM9098 | Great Britain | KY128904 | KY128696 | KY128491 | - |
| *Rubramoena rubescens* (Picton & Brown, 1978) | GNM9102 | Great Britain | KY128916 | KY128710 | KY128503 | - |
| *Sakuraeolis enosimensis* (Baba, 1930) | CASIZ178876 | California | HM162758 | HM162682 | HM162591 | - |
| *Samla bicolor* (Kelaart, 1858) | ZMMU:Op-68 | Vietnam,  Nha Trang | **MF523383** | **MF523436** | **MF523308** | **MF523503** |
| *Samla* sp. (Kelaart, 1858) | CAS177345 | Philippines | KY129050 | KY128841 | KY128635 | - |
| *Samla bilas* (Gosliner & Willan, 1991) | CAS177355 | Philippines | KY129051 | KY128842 | KY128636 | - |
| *Samla macassarana* (Bergh, 1905) | CAS181283 | Philippines | KY129059 | KY128850 | KY128644 | - |
| *Samla takashigei* sp. n. | ZMMU:Op-530 | Japan, Izu Peninsula | **MF523384** | **MF523463** | **MF523309** | **MF523529** |
| *Spurilla neapolitana* (Delle Chiaje, 1841) | CASIZ175756 | Portugal | HQ616764 | HQ616727 | HQ616793 | - |
| *Tenellia adspersa* (Nordmann, 1845) | CAS184191 | New Hampshire | KY129085 | KY128876 | KY128668 | - |
| *Tergipes tergipes* (Forsskål in Niebuhr, 1775) | WS3463 | Barents Sea | KY129090 | KY128881 | KY128673 | - |
| *Tergiposacca longicerata* Tergiposacca longicerata Cella, Carmona, Ekimova, Chichvarkhin, Schepetov & Gosliner, 2016 | CAS177605 | Philippines | KY129086 | KY128877 | KY128669 | - |
| *Trinchesia caerulea* (Montagu, 1804) | ZMMU:Op-622 | Norway,  Gulen | MG266024 | MG266022 | MG266025 | MG266023 |
| *Tritonia challengeriana* Bergh, 1884 | CASIZ171177 | Bouvet Island | HM162718 | HM162643 | HM162550 | - |
| *Tritonia plebeia* Johnston, 1828 | ZMMU:Op-572 | Norway | KX788134 | KX788122 | - | KX788132 |
| *Zelentia fulgens* (MacFarland, 1966) | CAS185194 | California | KY128952 | KY128747 | KY128540 | - |
| *Zelentia ninel* Korshunova, Martynov & Picton, 2017 | ZMMU:Op-509 | Russia,  Barents Sea | KY952178 | **MF523400** | **MF523242** | **MF523476** |
| *Unidentia sandramillenae* sp. n. | ZMMU:Op-617 | Bali | **MG452632** | **MG452683** | **MG452601** | - |
| *Unidentia nihonrossija* sp. n. | ZMMU:Op-517 | Japan,  Izu Peninsula | **MF523385** | **MF523464** | **MF523310** | - |
| *Ziminella abyssa* sp. n. | ZSM Mol-20100647 | Japan Sea | **MF523341** | **MF523465** | **MF523265** | - |
| *Ziminella abyssa* sp. n. | ZSM Mol-20100644 | Japan Sea | **MF523342** | **MF523466** | **MF523266** | - |
| *Ziminella circapolaris* sp. n. | ZMMU:Op-482 | Russia,  Franz Josef Land | **MF523354** | **MF523419** | **MF523278** | - |
| *Ziminella circapolaris* sp. n. | NTNU-VM:66918 | Norway, | - | **MG452653** | **MG452571** | - |
| *Ziminella salmonacea* (Couthouy, 1838) | CAS183927 | Maine | KY129062 | KY128853 | KY128647 | - |
| *Ziminella salmonacea* (Couthouy, 1838) | NTNU-VM:66921 | Norway,  Svalbard | HM425478 | **MG452651** | **MG452569** | - |

All new sequences are highlighted in bold.
