# Supplementary material for: Polyphyly of the traditional family Flabellinidae affects a major group of Nudibranchia: aeolidacean taxonomic reassessment with descriptions of several new families, genera, and species (Mollusca, Gastropoda)
Source: Zookeys. 2017 Nov 30;(717):1–139. doi: 10.3897/zookeys.717.21885 (PMC5784208; doi:10.3897/zookeys.717.21885)
Supplement: Supplementary material 3 — Table S2 [file zookeys-717-001-s003.doc]

Table S2. Primer sequences.

| **Name** | **5′→3′** | **References** |
| --- | --- | --- |
| LCO 1490 | GGTCAACAAATCATAAAGATATTGG | Folmer *et al*., 1994* |
| HCO 2198 | TAAACTTCAGGGTGACCAAAAAATCA | Folmer *et al*., 1994 |
| 16S arL | CGCCTGTTTAACAAAAACAT | Palumbi *et al*. 2002 |
| 16S R | CCGRTYTGAACTCAGCTCACG | Puslednik & Serb, 2008 |
| H3 AF | ATGGCTCGTACCAAGCAGACGG | Colgan *et al*., 1998 |
| H3 AR | ATATCCTTGGGCATGATGGTGAC | Colgan *et al*., 1998 |
| 28S C1 | ACCCGCTGAATTTAAGCAT | Dayrat et al. 2001 |
| 28S C2 | TGAACTCTCTCTTCAAAGTTCTTTTC | Lê et al, 1993 |

* References see in the main text
